# Beyond Monomers: Discovery of Bioactive Naphthoquinone Heterodimers and Perenniporides from *Pyrenochaetopsis* Species

**DOI:** 10.1021/acs.jnatprod.5c01209

**Published:** 2025-12-02

**Authors:** Reema A. Al-Qiam, Manuel Rangel-Grimaldo, Isabel M. Chauvin, Elizabeth N. Kaweesa, Manead Khin, Huzefa A. Raja, Jimmy Orjala, Joanna E. Burdette, Cedric J. Pearce, Nicholas H. Oberlies

**Affiliations:** † Department of Chemistry and Biochemistry, 14616 University of North Carolina at Greensboro, Greensboro, North Carolina 27402, United States; ‡ Instituto de Química, Universidad Nacional Autónoma de México, Ciudad de México 04510, México; § Department of Pharmaceutical Sciences, 14681 University of Illinois at Chicago, Chicago, Illinois 60607, United States; ∥ 331581 Mycosynthetix, Inc., Hillsborough, North Carolina 27278, United States

## Abstract

Naphthoquinones can be found in Nature in various formats, both as standalone molecules and as building blocks for larger structures. A study of the fungus, *Pyrenochaetopsis* sp. (strain MSX63699), led to the isolation of naphthoquinone analogues (**1**-**21**), including both monomers and heterodimers and seven new compounds—three kirschsteinin analogues (**11**-**13**) and four perenniporides (**18**-**21**). The structures of these were elucidated through analysis of data from 1D and 2D NMR, HRESIMS, and ECD experiments, and several compounds displayed cytotoxic activity against melanoma (MDA-MB-435) and ovarian (OVCAR3) cancer cell lines, with structure–activity trends suggesting key roles for specific substituents in enhancing bioactivity. Notably, compounds **3**-**6**, **9**, **11**, **15**, and **19–20** had IC_50_ values that ranged from about 1 to 5 μM against both cell lines. Overall, this work expands the chemical diversity of fungal naphthoquinones by identifying new heterodimeric and perenniporide-type analogues and underscores the untapped biosynthetic potential of the *Pyrenochaetopsis* species.

## Graphical abstract

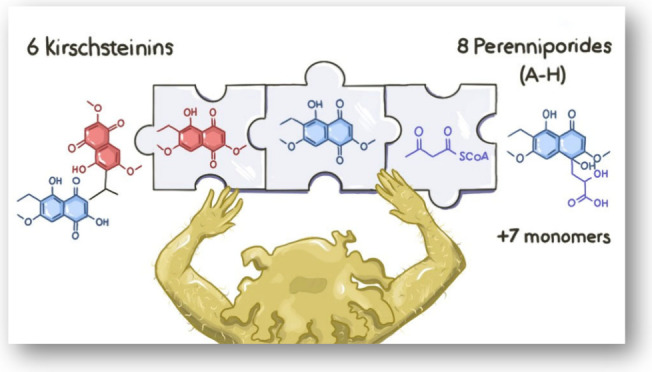

According to the American Cancer Society, in 2025 there will be over 2 million new cancer diagnoses in the United States, with an estimated 10 million deaths from the disease worldwide.^1^ By exploring unique compounds from natural sources,^2,3^ particularly fungi,^4,5^ we aim to expand the arsenal of anticancer agents from undiscovered fungi from the Mycosynthetix library.^6,7^

Naphthoquinones are a large class of secondary metabolites, with over 2000 members being reported from across the spectrum of life, including ∼400 members from fungi.^8–10^ They are characterized by a fused aromatic ring and a quinonoid structure and are known for their bioactivity in antibacterial, anti-inflammatory, and cytotoxicity assays, likely due to their ability to generate reactive oxygen species (ROS) and induce oxidative stress within cells.^10^ These compounds often feature fused aromatic rings, diverse substituents, and can form complex dimeric^11,12^ or trimeric structures,^13^ which in turn may contribute to their biological activities.^5^ Fungi seem to produce these naphthoquinones in response to communication with other biological systems,^14,15^ which can lead to structural modifications, such as glycosylation, hydroxylation, or methylation.^10,16^

Within this framework, heterodimeric and perenniporide-type naphthoquinones occupy a particularly intriguing niche.^17–19^ These metabolites exemplify how fungi can couple distinct naphthoquinone units or integrate additional aromatic scaffolds to create unprecedented molecular architectures.^20^ Such structural hybridization often enhances chemical stability, redox potential, and bioactivity profiles, underscoring their significance in both natural product chemistry and drug discovery.^9,15^ Consequently, exploring the biosynthetic diversity of these rare subclasses offers insight into fungal metabolic plasticity and may provide promising leads for further development.

## Results and Discussion

As part of a collaborative project to uncover cytotoxic metabolites from Nature,^21^ an extract of a solid phase culture of the Ascomycete, *Pyrenochaetopsis* sp. (strain MSX63699), was prioritized for further investigation for three reasons. Most importantly, it displayed cytotoxic activity against the human cancer cell lines MDA-MB-435 (melanoma) and OVCAR3 (ovarian), where less than 40% survival was observed when the extract was tested at 20 μg/mL. Furthermore, it passed dereplication against a database of >700 fungal metabolites, indicating that the extract did not contain well-known mycotoxins.^22,23^ Finally, this fungal lineage was first reported in 2010 ^24^ and has only received limited investigation for bioactive compounds, where derivatives of decalins^25–28^ and naphthoquinones^12^ have been reported. The fungus was a prolific producer of bioactive metabolites, as 21 naphthoquinone analogues were isolated (Figure S1) and characterized (*vide infra*). Members of this research team have studied cytotoxic fungal metabolites for over two decades, and this is only our second report of naphthoquinone analogues,^12^ and previously, 16 bioactive compounds, excluding mycotoxins, were the most that we had described from a single fungus.^29^ 
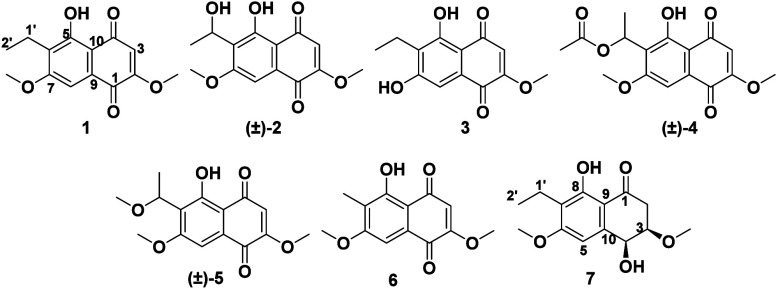


### Monomeric Naphthoquinones

There were seven monomeric naphthoquinones, which were identified based on favorable comparisons of their NMR data to those in the literature. Six of these were 1,4-naphthoquinones (where juglone is the most well-known example),^30,31^ specifically: 6-ethyl-2,7-dimethoxyjuglone (**1**),^32,33^ 6-(1-hydroxyethyl)-2,7-dimethoxyjuglone (**2**),^34^ 6-ethyl-7-hydroxy-2-methoxyjuglone (**3**),^34^ 6-[1-(acetyloxy)­ethyl]-2,7-dimethoxyjuglone (**4**),^12,34^ (1′-methoxy)-6-ethyl-2,7-dimethoxyjuglone (**5**),^12^ and misakimycin (**6**), which could also be noted as 6-methyl-2,7-dimethoxyjuglone (Figures S2–S7).^35^ Alternatively, compound **7** was the hydroxynaphthoquinone, botryosphaerone E (**7**) (Figure S8).^12^ The absolute configurations of **2**, **4**, **5**, and **7** were evaluated by ECD (Figure S9). Compounds **2**, **4**, and **5** were racemic, consistent with literature reports on these molecules.^12,34^ The Cotton effects for **7** matched the data from our previous report, indicating a configuration of 3*R*,4*S*.^12^ The cytotoxicity of **1**-**7** is discussed below in relation to the other molecules that were isolated. While structurally simple, there is continued interest in the advancement of monomeric naphthoquinones as anticancer leads, as evidenced by a recent review that summarized 197 analogues generated over the past decade.^36^ Monomeric 1,4-naphthoquinones are produced by many microorganisms, probably due to their diverse biological activities, including mechanisms such as topoisomerase inhibition,^37^ ROS-mediated oxidative stress,^38^ and DNA damage.^39^ They are also essential in vitamin K synthesis and the electron transfer pathway.^10,40^ Compound **1** was isolated in a high yield (∼95 mg), and as discussed below, represented a key building block in several of the heterodimeric (i.e., **8**-**12**) and perenniporide (i.e., **14**-**21**) structures.
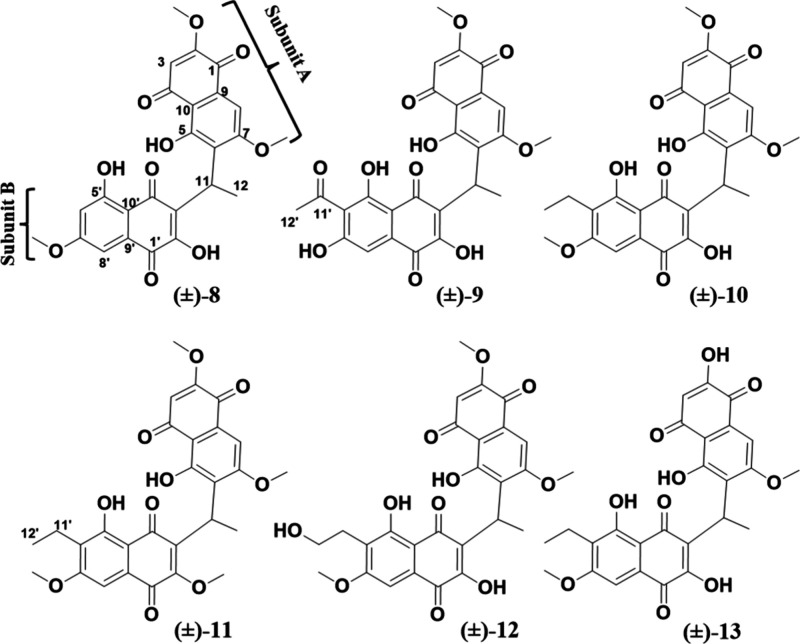


### Heterodimeric Naphthoquinones (Kirschsteinins)

There have only been five heterodimeric naphthoquinones reported from fungi, including kirschsteinin (**9**),^34^ deacetylkirschsteinin,^18^ kirschsteinin B (**10**) and C (**8**),^12^ and neofusnaphthoquinone B^11^ (Figure S10). In total, this fungus produced six heterodimeric naphthoquinones, likely biosynthesized via a head-to-tail coupling process (Figure S11), with three of them (**11**-**13**) being new to the literature. The NMR data for the known compounds [(kirschsteinin C (**8**),^12^ kirschsteinin (**9**),^34^ and kirschsteinin B (**10**);^12^ Figures S12–S14] agreed with the literature. Interestingly, for five of these compounds (**8**-**12**), subunit A was identical and likely derived from compound **1** (Figure S11). ECD data indicated that **8**-**13** were racemic (Figure S15). Previously, X-ray crystallography data of a heavy atom analogue of **8** also showed the epimeric nature of the C-11 position for this class of molecules.^12^ Their cytotoxicity are discussed in relation to the other isolated compounds below.

Compound **11** was isolated as an optically inactive orange powder, and its molecular formula was C_28_H_26_O_10_ based on HRESIMS data, yielding an index of hydrogen deficiency of 16 (Figure S16). The 1D and 2D NMR spectra revealed paired signals, indicative of the presence of two naphthoquinone subunits, an observation that was consistent with the structural features observed in other heterodimeric naphthoquinones (e.g., **8**-**10**).^12,34^ Compared to **10**, compound **11** showed one extra methoxy group in subunit B, while the subunit A signals matched those of the known compounds (Figure S11) and were further confirmed by HSQC and HMBC experiments (Figures 1, S17–S20, and Table 1). Briefly, HMBC correlations from H-11 (δ_H_ 4.96) and 5-OH (δ_H_ 12.83) to C-5 (δ_C_ 161.5), from H-3 (δ_H_ 6.05) and 2-OCH_3_ (δ_H_ 3.93) to C-2 (δ_C_ 160.6), and from H-11 and 7-OCH_3_ (δ_H_ 3.99) to C-7 (δ_C_ 163.0) confirmed the structure of subunit A. HMBC correlations from both H-11 and 2′-OCH_3_ (δ_H_ 3.89) to C-2′ (δ_C_ 158.5) indicated the position of the new methoxy at C-2′. Also, the ethyl moiety was confirmed at C-6′ based on correlations from both H-11′ (δ_H_ 2.76, q) and 5′–OH (δ_H_ 12.65) to C-5′ (δ_C_160.6) and from H-11′ to C-7′ (δ_C_ 162.3). Compound **11** was ascribed the trivial name kirschsteinin D.

**Figure 1:**
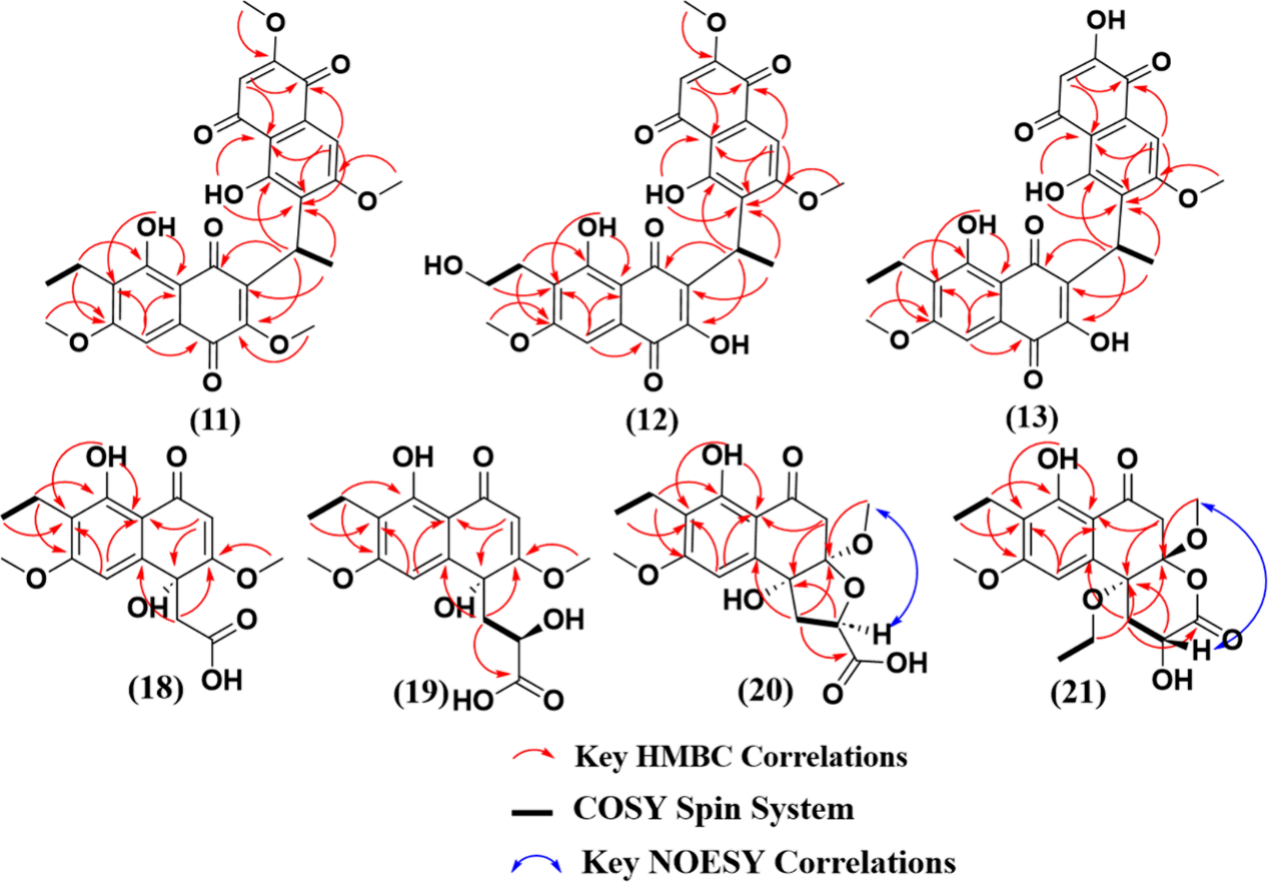
Spin systems (from COSY data), key HMBC, and key NOESY correlations for the kirschsteinin analogues (i.e., **11**-**13**) and the perenniporide-related naphthoquinones (i.e.,**18**-**21**).

**Table 1:** ^1^H and ^13^C NMR Data for Compounds **11**-**13**

	11^a^		12^b^		13^c^	
position	δ_C_, type	δ_H_, mult. (*J* in Hz)	δ_C_, type	δ_H_, mult. (*J* in Hz)	δ_C_, type	δ_H_, mult. (*J* in Hz)
1	180.8, C		179.7, C		179.6, C	
2	160.6, C		160.6, C		160.6, C	
3	109.6, CH	6.05, s	109.6, CH	6.01, s	109.3, CH	6.69, s
4	190.0, C		190.1, C		190.1, C	
5	161.5, C		161.3, C		161.0, C	
6	127.1, C		127.3, C		127.4, C	
7	163.0, C		162.8, C		162.8, C	
8	103.5, CH	7.28, s	103.5, CH	7.23, s	102.4, CH	7.30, s
9	130.3, C		130.4, C		128.8, C	
10	109.0, C		109.1, C		109.5, C	
11	28.9, CH	4.96, q (7.4)	28.1, CH	4.95, q (7.4)	28.0, CH	4.33, q (7.4)
12	18.1, CH_3_	1.72, d (7.4)	17.3, CH_3_	1.68, d (7.4)	17.3, CH_3_	1.40, d (7.4)
2-OCH_3_	56.7, CH_3_	3.93, s	56.6, CH_3_	3.87, s	-	-
7-OCH_3_	56.2, CH_3_	3.99, s	56.4, CH_3_	3.94, s	56.5, CH_3_	3.92, s
5-OH	-	12.83, s	-	12.83, s	-	12.33, s
1′	181.6, C		181.5, C		181.6, C	
2′	158.5, C		154.0, C		153.8, C	
3′	137.9, C		124.1, C		124.0, C	
4′	190.1, C		190.4, C		190.3, C	
5′	160.7, C		160.9, C		160.6, C	
6′	127.0, C		123.2, C		127.9, C	
7′	162.3, C		162.3 C		161.7, C	
8′	102.2, CH	7.18, s	102.4, CH	7.21, s	103.5, CH	7.29, s
9′	130.5, C		128.7, C		130.4, C	
10′	109.4, C		109.5, C		109.0, C	
11′	16.4, CH_2_	2.76, q (7.5)	26.7, CH_2_	3.01, t (6.5)	16.5, CH_2_	2.75, q (7.5)
12′	13.1, CH_3_	1.14, t (7.5)	62.1, CH_2_	3.80, t (6.5)	13.0, CH_3_	1.12, t (7.5)
2′-OCH_3_	60.9, CH_3_	3.89, s	-	-	-	-
7′-OCH_3_	56.5, CH_3_	3.99, s	56.7, CH_3_	3.93, s	56.2, CH_3_	4.00, s
5′–OH	-	12.65, s	-	12.84, s	-	12.24, s
2′–OH	-	-	-	7.64, bs	-	-

a 500 and 125 MHz, respectively, in CDCl_3_.

b 400 and 100 MHz, respectively, in CDCl_3,_

c 700 and 175 MHz, respectively, in CDCl_3._

Compound **12** was obtained as an optically inactive orange powder, and its molecular formula was C_27_H_24_O_11_ based on the HRESIMS data, signifying 16 degrees of unsaturation (Figure S21). Similar to **11**, subunit A was based on compound **1**, as noted by HMBC and HSQC data (Figures 1 and S22–S25), and methoxy signals were evident at positions 2-OCH_3_ (δ_H/C_ 3.87/56.6), 7-OCH_3_ (δ_H/C_ 3.94/56.4), and 7′-OCH_3_ (δ_H/C_ 3.93/56.7). In **12**, C-2′ (δ_C_ 154.0) was hydroxylated based on an HMBC correlation from H-11 (δ_H_ 4.94) to C-2′; the chemical shift at this position was nearly identical to those for compounds **8**-**10** (δ_C‑2′_ 153.8–154.0). In the ^1^H NMR and COSY data (Figures 1, S23 and Table 1), a difference between **10** and **12** was noted in the ethyl moiety at C-6′ (δ_C_ 123.2), suggesting an oxygenated methylene group at C-12′ (δ_C_ 62.1) in the latter. This was confirmed by COSY data and HMBC correlations from H-11′ (δ_H/C_ 3.01/26.7) to C-5′ (δ_C_ 160.9) and C-7′ (δ_C_ 162.3), and from H-12′ (δ_H_ 3.80) to C-6′ (δ_C_ 123.2) (Figure 1). Compound **12** was ascribed to the trivial name kirschsteinin E.

Similar to **11** and **12**, compound **13** was also an optically inactive orange powder and had a molecular formula of C_26_H_22_O_10_ and 16 degrees of unsaturation (Figure S26). There were many similarities in the ^1^H and ^13^C NMR spectra between compounds **10** and **13** (Figures S27–S30). For instance, the signals ascribed to subunit B were nearly identical. Examples include HMBC correlations to assign the position of the ethyl moiety via signals from H-11′ (δ_H_ 2.75) to both C-7′ (δ_C_ 161.7) and C-5′ (δ_C_ 160.6), signals from 7′-OCH_3_ (δ_H_ 4.00) to C-7′ to assign the methoxy at C-7′, and signals from H-11 (δ_H_ 4.33) to C-2′ (δ_C_ 153.8) to assign the 2′–OH. HMBC correlations from H-11 (δ_H_ 4.33), H-8 (δ_H_ 7.30), and 7-OCH_3_ (δ_H_ 3.92) to C-7 (δ_C_ 162.8) indicated the position of the second methoxy at C-7, and those from H-11 and 5-OH (δ_H_ 12.33) to C-5 (δ_C_ 161.0) indicated the position of the hydroxy at C-5. These data showed that subunits A and B were identical. Interestingly, that monomeric building block was not observed in the fungal extract, and this could explain the low isolated yield of **13** (i.e., <1 mg) relative to **8**-**12**. Compound **13** was ascribed to the trivial name kirschsteinin F.
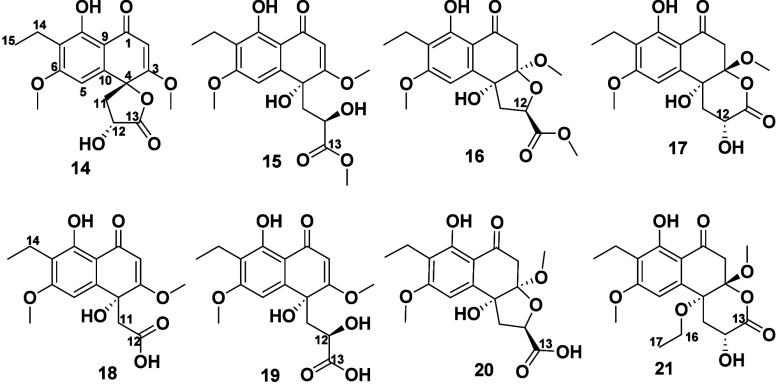


### Perenniporides (A-H)

The third subclass of naphthoquinones were a series of perenniporides (**14–21**), which are a rare class of compounds that were previously isolated from the fungi *Perenniporia* sp.^41^ and *Guignardia laricina* (now called *Neofusicoccum laricinum*).^32^ Four of these were identified as perenniporides A-D (**14**-**17**), and their NMR spectroscopy data (Figures S31–S34) and absolute configurations via ECD (Figure S35) were both in good agreement with the literature.^41^ The absolute configuration of **14–16** were also confirmed via total synthesis.^17^ Previously, those compounds were tested as antifungals, and only perenniporide A (**14**) showed an inhibitory effect against five tested pathogens.^41^ Their cytotoxicity are discussed in relation to the other isolated compounds below.

Compound **18** had a molecular formula of C_16_H_18_O_7_ (8 degrees of unsaturation) based on analysis of HRESIMS data (Figure S36). In the ^1^H and ^13^C NMR spectra, there were clear similarities between compounds **18** and **15** in terms of the ethyl group attached to C-7 (δ_C_ 119.0), the two methoxy groups at 6-OCH_3_ (δ_H/C_ 3.90/56.4) and 3-OCH_3_ (δ_H/C_ 3.96/57.1), two sp^2^ protons at C-2 (δ_H/C_ 5.65/101.6) and C-5 (δ_H/C_ 6.95/101.5), an oxygenated quaternary carbon C-4 (δ_C_ 71.3), and diastereotopic methylene protons at C-11 (δ_H/C_ 3.09 and 3.30/47.2) (Figure S37). HSQC and HMBC correlations were important to further elucidate the structure (Table 2 and Figures S38–S40). Correlations from H_2_-11 to C-3 (δ_C_ 176.7), C-4 (δ_C_ 71.3), C-10 (δ_C_ 145.5), and C-12 (δ_C_ 170.6) confirmed that the carbon chain at position 4 is one carbon shorter (compared to **15**) and terminated with a carboxylic acid (δ_C‑12_ 170.6). The absolute configuration of **18** was deduced by a comparison of the experimental and simulated electronic circular dichroism (ECD) spectra generated by the time-dependent density functional theory (TDDFT) method with the CAM-B3LYP/Def2-TZVP level of theory, demonstrating concordance between the experimental and calculated spectra for the 4*R* configuration (Figure 2A). Compound **18** was ascribed the trivial name perenniporide E.

**Figure 2:**
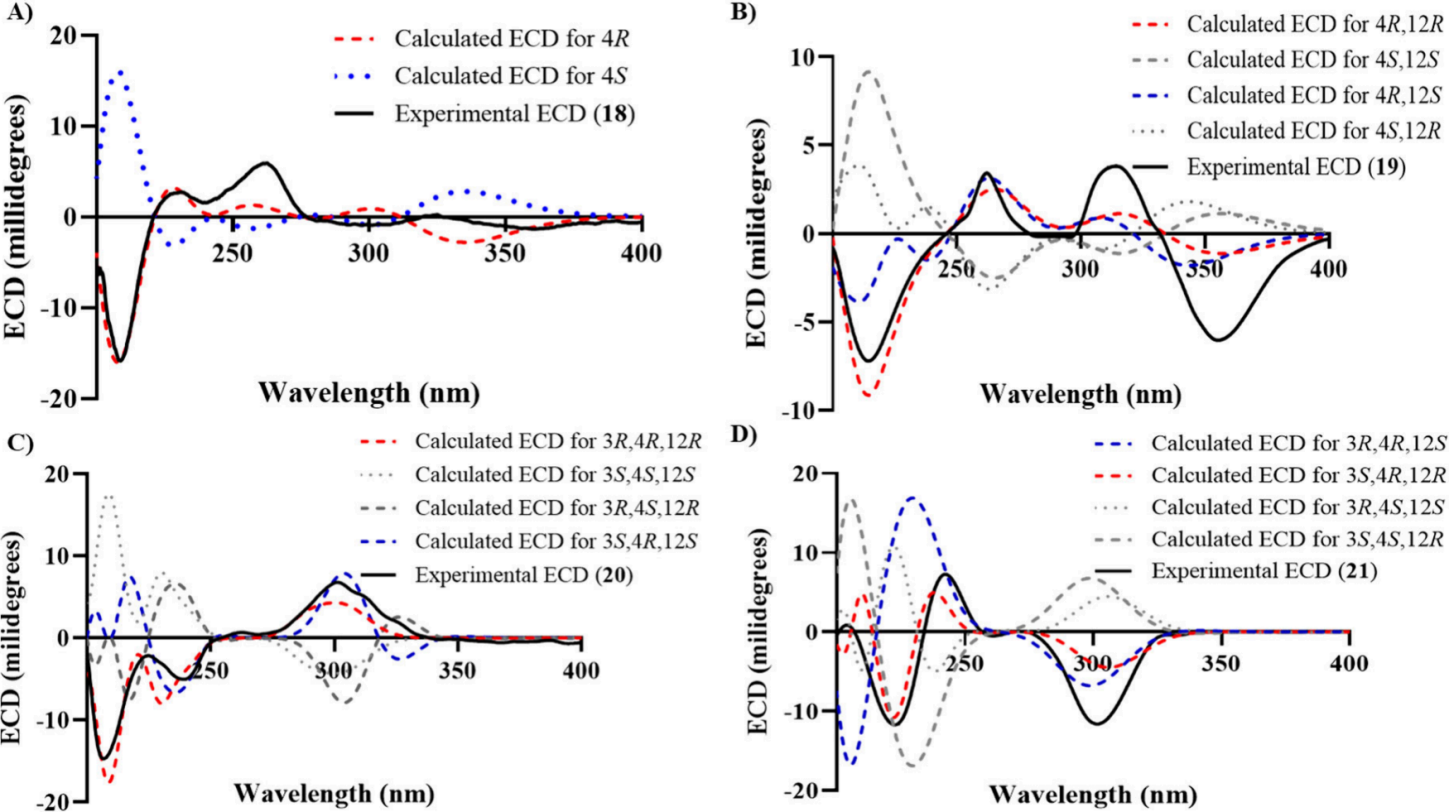
Comparison of the calculated vs experimental ECD spectra in MeOH for compounds **18** (**A**), **19** (**B**), **20** (**C**), and **21** (**D**).

**Table 2:** ^1^H and ^13^C NMR Data for Compounds **18**-**21**

	18^a^		19^b^		20^c^		21^c^	
position	δ_C_, type	δ_H_, mult. (*J* in Hz)	δ_C_, type	δ_H_, mult. (*J* in Hz)	δ_C_, type	δ_H_, mult. (*J* in Hz)	δ_C_, type	δ_H_, mult. (*J* in Hz)
1	191.3, C		191.6, C		197.4, C		197.7, C	
2	101.6, CH	5.65, s	101.5, CH	5.58, s	40.3, CH_2_	3.02, d (16.7)	40.9, CH_2_	3.08, d (16.0)
						3.19, d (16.7)		3.25, d (16.0)
3	176.7, C		177.9, C		104.9, C		105.1, C	
4	71.3, C		79.3, C		78.9, C		78.2, C	
5	101.5, CH	6.95, s	103.7, CH	6.79, s	102.3, CH	6.78, s	102.2, CH	6.73, s
6	163.2, C		164.9, C		164.1, C		163.7, C	
7	119.0, C		119.8, C		120.2, C		119.5, C	
8	161.1, C		161.7, C		161.1, C		160.7, C	
9	109.4, C		106.1, C		109.6, C		109.5, C	
10	145.5, C		146.4, C		141.7, C		142.4, C	
11	47.2, CH_2_	3.09, d (14.8)	43.6, CH_2_	2.76, dd (14.4, 12.5)	42.9, CH_2_	2.78, dd (12.7, 8.2)	43.2, CH_2_	2.75, dd (13.0, 4.8)
		3.30, d (14.8)		2.87, dd (14.4, 6.0)		2.91, dd (12.7, 8.2)		2.89, dd (13.0, 8.9)
12	170.6, C		75.6, CH	4.50, dd (12.5, 6.0)	73.7, CH	4.28, t (8.2)	61.4, CH	4.68, dd (8.9, 4.8)
13	-		171.8, C		174.9, C		171.2, C	
14	16.3, CH_2_	2.64, q (7.7)	16.4, CH_2_	2.57, q (7.7)	15.8, CH_2_	2.66, q (7.4)	15.6, CH_2_	2.63, q (7.4)
15	13.8, CH_3_	1.06, t (7.7)	13.5, CH_3_	1.01, t (7.7)	13.1, CH_3_	1.08, t (7.4)	13.0, CH_3_	1.04, t (7.4)
16	-	-	-	-	-	-	74.4, CH_2_	3.92, q (7.1)
17	-	-	-	-	-	-	13.8, CH_3_	1.05, t (7.1)
3-OCH_3_	57.1, CH_3_	3.96, s	56.5, CH_3_	3.88, s	50.3, CH_3_	3.55, s	50.0, CH_3_	3.49, s
6-OCH_3_	56.4, CH_3_	3.90, s	56.3, CH_3_	3.85, s	55.9, CH_3_	3.95, s	55.8, CH_3_	3.90, s
4-OH	-	8.11, s	-	-	-	-	-	-
8-OH		13.34, s	-	-	-	12.46, s	-	12.49, s

a 500 and 125 MHz, respectively, in Acetone-*d*_*6*_.

b 500 and 125 MHz, respectively, in CD_3_OD,

c 500 and 125 MHz, respectively, in CDCl_3._

Compound **19** was isolated as an optically active amorphous white powder with a molecular formula of C_17_H_20_O_8_ (8 degrees of unsaturation) based on HRESIMS data (Figure S41). 1D (Table 2 and Figure S42) and 2D (Figures S43–S45) NMR data analysis showed similarities between **19** and **15**, with only one methoxy missing in **19**. ^13^C NMR data suggested the replacement of the methyl ester moiety with a carboxylic acid (Figure S46) [δ_C‑13_ 175.3 for **15** vs 171.8 for **19**]. Key HMBC correlations from H-11 (δ_H_ 2.76/2.87) to C-10 (δ_C_ 146.4), C-3 (δ_C_ 177.9), and C-13 (δ_C_ 171.8), confirmed the C-4 side chain and indicated the placement of the carboxylic acid, similar to **15**. The positions of the methoxy groups were confirmed by HMBC correlations from 6-OCH_3_ (δ_H_ 3.85) and H-14 (δ_H_ 2.57) to C-6 (δ_C_ 164.9) and from 3-OCH_3_ (δ_H_ 3.88), H-2 (δ_H_ 5.58), and H-11 to C-3 (δ_C_ 177.9). The absolute configuration of **19** was deduced by comparison of the experimental and calculated ECD spectra for the four possible stereoisomers, where concordance was observed for the 4*R*,12*R* configuration (Figure 2B). Compound **19** was ascribed the trivial name perenniporide F.

Compound **20** was isolated as an optically active amorphous powder with a molecular formula of C_17_H_20_O_8_ (8 degrees of unsaturation) based on HRESIMS data (Figure S47). The ^1^H NMR and HSQC data (Table 2 and Figures S48–S50) indicated one aromatic proton (δ_H‑5_ 6.78), two methoxy signals (δ_H_ 3.55 and 3.95), an ethyl group (δ_H‑15_ 1.08 for CH_3_, and δ_H‑14_ 2.66 for CH_2_), two diastereotopic methylene groups (δ_H‑2_ 3.02/3.19 and δ_H‑11_ 2.78/2.91), and one oxygenated methine (δ_H‑12_ 4.28). Analysis of 1D and 2D NMR data confirmed similarities between **16** and **20**, with the key difference being two methoxy groups in **20**, compared to three in **16**. Analysis of HMBC correlations (Figure S51) positioned the two methoxy groups at C-3 (δ_C_ 104.9) and C-6 (δ_C_ 164.1), thereby indicating a carboxylic acid at C-13 (δ_C_ 174.9). The NOESY correlation (Figure S52) between H-12 and the 3-OCH_3_ group indicated their positioning on the same face of the 5-membered ring, enabling their relative configuration assignment as *rel-3R,12R/3S,12S*. Thus, it was possible to reduce the number of calculated conformers to four (i.e., **20A**-**D** in Figure S53). Comparing the calculated vs experimental ECD for the four possibilities indicated the absolute configuration of 3*R*,4*R*,12*R* for **20** (Figure 2C). This was also confirmed by comparing the calculated and experimental optical rotation data (Tables S1 and S2 summarize the ECD, optical rotation, and NOESY data). Compound **20** was ascribed to the trivial name perenniporide G.

Compound **21** was isolated as an optically active amorphous powder with a molecular formula of C_19_H_24_O_8_ (8 degrees of unsaturation) based on HRESIMS data (Figure S54). The NMR data (Table 2 and Figures S55–S58) revealed that **21** was similar to **17**, except that **21** had an additional ethyl moiety (δ_C/H_ 13.8/1.05 for CH_3_, and 74.4/3.92 for CH_2_). The HMBC correlations from H-5 (δ_H_ 6.73), H-2 (δ_H_ 3.08, 3.25), and H-16 (δ_H_ 3.92) to C-4 (δ_C_ 78.2) confirmed the connection of the ethyl group to C-4. Similar to **20**, a NOESY correlation (Figure S59) between H-12 and the 3-OCH_3_ group narrowed the number of possible conformers for ECD calculations (i.e., four instead of eight; **21A**-**D**, Figure S60). Calculation of the ECD (Figure 2D) and optical rotation data (Tables S3 and S4) indicated the absolute configuration of **21** as 3*S*,4*R*,12*R*. Compound **21** was ascribed to the trivial name perenniporide H.

An obvious question is whether compound **19** could result from acid-induced demethylation of **15** during the HPLC purification process,^42^ with a similar concern for compound **20** relative to **16**. To address this, we prepared compound **16** in CDCl_3_ containing 0.1% formic acid and monitored its stability by NMR (Figure S61). Compound **16** remained stable, with no detectable side products, for up to 48 h, suggesting that the carboxylic acids (i.e., **19** and **20**) are not artifacts. During isolation and analytical characterization, all members of the perenniporide series except **19** exhibited good stability, even at low pH, extended storage, and/or in MeOH solutions. For instance, during the time frame of this project (>2 years), we did not observe notable degradation of any of the perenniporides, apart from **19** (Figure S63).

Previously, Feng et al. proposed the biosynthesis of perenniporides A-D,^41^ which we have expanded upon for perenniporides A-H (**14**-**21**; Figure S62), suggesting that all of them could be biosynthesized from **1** and an additional acetoacetyl CoA unit. Although no specific biosynthetic enzymes have been identified for the formation of the perenniporides, the proposed pathway is inferred from the well-established biosynthesis of the naphthoquinone starter unit (i.e., compound **1**).^20,43,44^ This precursor is likely generated by a polyketide synthase, followed by oxidative coupling and tailoring reactions, such as hydroxylation and methylation. These steps, commonly mediated by fungal oxidases and P450 enzymes, could account for the structural diversity observed in the perenniporide series.^43,44^ Thus, compound **19** could result from the oxidation of the first intermediate and is proposed to be an intermediate of the other perenniporides (i.e., **14**-**17**, and **20**-**21**). Methylation of **19** could yield **15**, while dehydration could generate **14**. Alternatively, cyclization could happen either to generate a 5-membered ring (**16** and **20**) or a 6-membered ring (**17** and **21**). In the course of this study, **19** was isolated in the smallest quantity among the perenniporides. Interestingly, when left in solution for a few days (CDCl_3_), it gradually converted into compound **14** (Figure S63), suggesting that mild conditions are sufficient to induce cyclization. This observation supports the notion that **19** may serve as a key intermediate for several perenniporides. Regardless, the absolute configurations of compounds **14**–**21** were consistent, indicating that they likely originate from a common precursor.

#### Cytotoxicity of compounds **1–21**

The cytotoxic activities of compounds **1**–**21** were evaluated against the human cancer cell lines MDA-MB-435 (melanoma) and OVCAR3 (ovarian) (Table 3, Figure 3 and further summarized in Table S30). All of the monomeric naphthoquinones were active in both cell lines (∼2–8 μM). However, compound **7** was the least active, likely due to the reduction of both the carbonyl at C-1 and the Δ2^(3)^ double bond to a hydroxynaphthoquinone. This modification likely decreases the electrophilicity at the quinone center, attenuating redox cycling and ROS generation, which are mechanisms known to underlie naphthoquinone cytotoxicity.

**Table 3:** Cytotoxic Activity of Compounds 1–21 against Ovarian (OVCAR3) and Breast (MDA-MB-435) Cancer Cell Lines

	IC_50_ ^a^ values (μM) ± Std. Dev.	
Compound	OVCAR3	MDA-MB-435
**1**	2.3 ± 1.0	10.2 ± 0.2
**2**	6.4 ± 1.6	8.0 ± 1.6
**3**	3.5 ± 0.2	1.4 ± 0.1
**4**	3.8 ± 0.7	2.5 ± 0.7
**5**	4.7 ± 0.9	0.6 ± 0.1
**6**	2.1 ± 0.7	4.0 ± 0.1
**7**	23.6 ± 0.2	22.3 ± 1.4
**8**	12.4 ± 0.1	13.3 ± 0.7
**9**	2.1 ± 0.8	5.1 ± 0.2
**10**	8.2 ± 0.1	8.7 ± 1.0
**11**	3.4 ± 0.1	1.4 ± 0.4
**12**	5.6 ± 0.0	9.1 ± 1.3
**13**	>25	>25
**14**	>25	>25
**15**	3.3 ± 0.8	11.0 ± 0.3
**16**	6.8 ± 1.3	1.5 ± 0.2
**17**	4.3 ± 0.8	6.5 ± 2.5
**18**	6.0 ± 0.7	12.7 ± 1.1
**19**	2.3 ± 0.0	9.0 ± 0.9
**20**	2.0 ± 0.0	7.2 ± 1.0
**21**	7.2 ± 1.3	8.7 ± 0.7
**taxol** ^b^ **(nM)**	0.1 ± 0.0	0.1 ± 0.0

a IC_50_ values were determined as the concentration required to inhibit growth to 50% of the control with a 72-h incubation at 37 °C; standard deviations are also reported.

b Positive control: taxol (paclitaxel).

**Figure 3:**
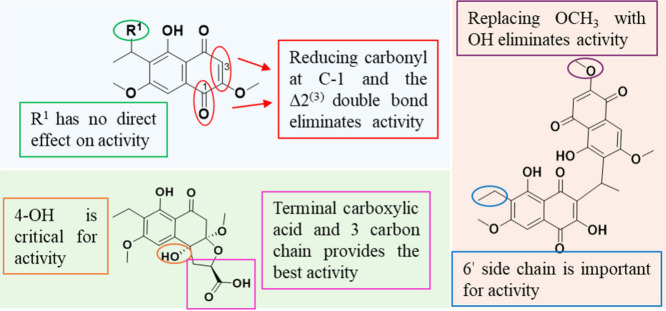
Summary of the structure activity relationships for naphthoquinones (blue box), kirschsteinins (orange box), and perenniporides (green box). Additional observations are summarized in Table S30.

Within the heterodimeric analogues (**8**-**13**), all except **13** retained cytotoxic activity, suggesting that the methoxy at position 2 was important for activity. Also, compounds **9**, **11**, and **12** were the most potent, suggesting that the substituents at subunit B, particularly those at C-6′, served to improve cytotoxic activity. Enhanced activity in these analogues may result from electron-donating methoxy or hydroxy groups at C-2 and C-6′, which could facilitate redox cycling or stabilize semiquinone intermediates. In contrast, **13** was inactive despite structural similarity to **10**. Its inactivity, coupled with its low yield, suggests the low stability of **13** mainly due to the formation of ortho-quinones that might increase off-target effects.^45^

Regarding the perenniporides (**14**-**21**), most analogues were active, and **19** and **20** were the most potent, suggesting that the size of the side chain at position 4 and the carboxylic acid may enhance cytotoxicity, possibly by increasing polarity and improving interactions with cellular targets or membrane transport. In contrast, **14**, which lacks a substituent at this position, was inactive, underscoring the importance of the hydrogen-bonding capacity or chain length for bioactivity.

## Conclusion

In summary, studies of *Pyrenochaetopsis* sp. (strain MSX63699) led to the isolation of 21 structurally diverse naphthoquinone analogues, including seven monomers, six heterodimers, and eight perenniporide-related compounds. Among these, seven were identified as new natural products—three kirschsteinins (**11**-**13**) and four perenniporides (**18**-**21**). These findings expand the structural landscape of fungal naphthoquinones, particularly the rare heterodimeric and perenniporide subclasses. Cytotoxicity assays revealed that several of these compounds exhibited activity against melanoma and ovarian cancer cell lines, with structure–activity relationship insights highlighting functional groups critical for activity, especially within the heterodimeric and perenniporide series. Our understanding of the biosynthesis of the perenniporides may have been enhanced by the discovery of **19**, which could serve as a common precursor for the other members of this relatively new series of compounds. Altogether, this study reinforces the importance of *Pyrenochaetopsis* sp. as a prolific source of bioactive natural products. Studies to further capitalize on the vast structural diversity afforded by this fungus are ongoing, particularly with respect to the tricyclic perenniporides, such as **16**, which was isolated in a high enough yield to be used as a starting material for semisynthesis.

## Experimental Section

### General Experimental Procedures

ECD data were obtained using a Jasco J-815 CD Spectrometer using MeOH as solvent and a scanning speed of 200 nm/min with continuous scanning; bandwidth was 1 nm. UV and Optical rotation spectra were acquired on a Varian Cary 100 Bio UV–Vis spectrophotometer and a Rudolph Research Autopol III polarimeter (Rudolph Research Analytical), respectively. NMR data were collected using either a JEOL ECS-400 NMR spectrometer equipped with a high sensitivity JEOL Royal probe operating at 400 MHz for ^1^H and 100 MHz for ^13^C or a JEOL ECA-500 NMR spectrometer operating at 500 MHz for ^1^H and 125 MHz for ^13^C, or an Agilent 700 MHz NMR spectrometer (Agilent Technologies), equipped with a cryoprobe, operating at 700 MHz for ^1^H and 175 MHz for ^13^C. Residual solvent signals were utilized for referencing, CDCl_3_ [δ_H_/δ_C_ 7.260/77.160], CD_3_OD [δ_H_ */δ*_C_ 3.310/49.000], Acetone-*d*_*6*_ [δ_H_ */δ*_C_ 2.050/206.260], and DMSO-*d*_*6*_ [δ_H_/δ_C_ 2.503/39.520]. HRMS experiments utilized either a Thermo LTQ Orbitrap XL mass spectrometer or a Thermo Q Exactive Plus (Thermo Fisher Scientific); both instruments were equipped with an electrospray ionization source. UPLC was carried out on a Waters Acquity system, with data collected and analyzed using Thermo Xcalibur software. HPLC was carried out using a Shimadzu-2050 HPLC system equipped with LC-20AR pumps, an SPD-M40 photodiode array detector (PDA) and an FRC-10A fraction collector with data collected and analyzed using LabSolutions software (version 5.127). Flash column chromatography was carried out with a Teledyne ISCO Combiflash Rf connected to both an evaporative light scattering detector and a PDA detector with UV set at 200–400 nm.

### Fungal Strain Identification

Strain MSX63699 was isolated from leaf litter on July 3, 1992, and is maintained at the Mycosynthetix culture collection in Hillsborough, North Carolina. It was identified using molecular data from the internal transcribed spacer (ITS) rDNA region since morphological characters were unavailable. Protocols for DNA extraction, PCR amplification, and Sanger sequencing have been outlined previously.^46^ Based on the NCBI GenBank BLAST search using the rRNA/ITS RefSeq database,^47^ the closest matches (≥97–98%) were sequences of the coelomycetous Dothideomycetes fungal genus *Pyrenochaetopsis*.^48,49^ All type species from *Pyrenochaetopsis* were downloaded from GenBank directly to the program Seaview v.5.05.^50^ Sequences were aligned using MUSCLE.^51^ Ambiguous regions were delimited and removed from the multiple sequence alignment using ClipKIT.^52,53^ Phylogenetic analysis were performed using IQTree^54^ in the program PhyloSuite.^55,56^ Prior to running the IQTree analysis, ModelFinder,^57^ was implemented in PhyloSuite. Based on Akaike information criterion (AIC) ModelFinder chose TIM2e+G4 as the most likely model. IQTree analysis was then run using 5000 ultrafast bootstraps.^58^ An additional bootstrap analysis using 1000 replicates was also performed using PhyML^59,60^ analysis in Seaview. Bayesian Inference phylogenies were inferred using MrBayes v3.2.7a^61^ under TIM2e+G4 in PhyloSuite (2 parallel runs, 10,000,0000 generations), in which the initial 25% of sampled data were discarded as burn-in. Results of the IQTree maximum likelihood analysis placed strain MSX63699 within the genus *Pyrenochaetopsis.* The strain was identical to strain MSX63693, which was reported by our group previously as *Pyrenochaetopsis* and was also shown to produce cytotoxic naphthoquinone analogues.^12^ Based on the results of the maximum likelihood phylogeny, we identified strain MSX63699 as *Pyrenochaetopsis* sp. (Pyrenochaetopsidaceae, Pleosporales, and Ascomycota) (Figure S64). The sequence data were deposited in GenBank (accession no: ITS: PV962929).

### Extraction and Isolation

The fungus was grown on 10 g of autoclaved rice in 250 mL Erlenmeyer flasks (n = 6). After approximately 3 weeks of growth at rt, 60 mL of 1:1 CHCl_3_–MeOH were added. The sample was chopped with a spatula and shaken for ∼16 h at ∼100 rpm at rt. The six samples were combined and filtered in vacuo, and the remaining residue was washed with MeOH. To the filtrate were added 90 mL of CHCl_3_ and 150 mL of H_2_O were added. The biphasic solution was stirred for 30 min and then transferred to a separatory funnel. The organic (bottom) layer was drawn off into a round-bottom flask, which was evaporated to dryness. The dried organic extract was then reconstituted in 100 mL of hexanes and 100 mL of 1:1 MeCN-MeOH, and the biphasic solution was shaken vigorously and then transferred to a separatory funnel. The bottom layer was drawn off into a round-bottom flask and evaporated to dryness under vacuum. The solid marc from the initial extraction was then sonicated with acetone (∼500 mL), filtered, dried, and then the two-extraction steps were repeated to ensure the full extraction of the naphthoquinones from the rice media, as reported recently for a similar class of compounds.^62,63^

The resulting defatted extracts (900 mg) and acetone wash (1.1 g) were dissolved in CHCl_3_, mixed together, and adsorbed onto Celite 545, prior to purification via flash chromatography using a RediSep silica column (12g) and a gradient solvent system of hexane–CHCl_3_–MeOH at a flow rate of 30 mL/min over 40 min; 70 column volumes were collected and pooled into five fractions, in which naphthoquinones were observed in fractions 1–4. Fraction 1 from flash chromatography was subjected to preparative RP-HPLC using a Gemini C_18_ column with a gradient system of 40:60 to 90:10 MeCN–H_2_O (acidified with 0.1% formic acid) over 30 min at a flow rate of 20.0 mL/min to yield seven subfractions. Fraction 1 yielded compounds **1** (95.6 mg), **2** (5.4 mg), **3** (3.5 mg), **4** (16.5 mg), **5** (1.5 mg), **6** (5.5 mg), and **7** (3.3). Fraction 2 from flash chromatography was purified via RP-HPLC using the same method to yield compounds **8** (6.3 mg), **9** (3.7 mg), **10** (3.5 mg), and **13** (0.7 mg). Fraction 3 was subjected to a preparative RP-HPLC using a Gemini column with a gradient system of 30:70 to 50:50 of MeCN–H_2_O (acidified with 0.1% formic acid) over 20 min, then to 80:20 over an additional 10 min, at a flow rate of 20.0 mL/min to generate compounds **11** (3.1 mg), **12** (2.9 mg), **14** (110.5 mg), **15** (11.0 mg), **16** (80.3 mg), **17** (11.0 mg), **19** (1.3 mg), **20** (10.2 mg), and **21** (7.7 mg). Finally, fraction 4 from flash chromatography yielded **18** (2.2 mg), effectively using the same method and conditions used for fraction 3. Table S5 shows the SMILES, InChI, and InChIKey descriptors of compounds **1**-**21**.

#### Kirschsteinin D (**11**)

Orange solid. [α]_D_^20^ −0.1 (c 0.13, MeOH); UV (MeOH) λ_max_ (log ε) 223 (3.5), 266 (3.5), 308 (3.4) nm, 427 (3.1); ^1^H and ^13^C NMR, Table 1; HRESIMS *m*/*z* 523.1594 [M + H]^+^ (calcd for C_28_H_27_O_10_, [M + H]^+^= 523.1598).

#### Kirschsteinin E (**12**)

Orange solid. [α]_D_^20^ 0.0 (c 0.13, MeOH); UV (MeOH) λ_max_ (log ε) 225 (3.5), 265 (3.5), 315 (3.4) nm, 419 (3.1); ^1^H and ^13^C NMR, Table 1; HRESIMS *m*/*z* 525.1391 [M + H]^+^ (calcd for C_27_H_25_O_11_, [M + H]^+^= 525.1391).

#### Kirschsteinin F (**13**)

Orange solid. [α]_D_^20^ −0.3 (c 0.13, MeOH); UV (MeOH) λ_max_ (log ε) 223 (3.5), 266 (3.5), 308 (3.4) nm, 451 (3.1); ^1^H and ^13^C NMR, Table 1; HRESIMS *m*/*z* 495.1288 [M + H]^+^ (calcd for C_26_H_23_O_10_, [M + H]^+^= 495.1286).

#### Perenniporide E (**18**)

Pale yellow powder. [α]_D_^20^ +31.0 (c 0.1, MeOH); UV (MeOH) λ_max_ (log ε) 210 (1.8), 260 (1.9), 299 (0.1) nm;^1^H and ^13^C NMR, Table 2; HRESIMS *m*/*z* 323.1120 [M + H]^+^ (calcd for C_16_H_19_O_7_, [M + H]^+^= 323.1125).

#### Perenniporide F (**19**)

white amorphous powder. [α]_D_^20^ −16.6 (c 0.1, MeOH); UV (MeOH) λ_max_ (log ε) 210 (3.1), 256 (1.8), 335 (2.56) nm;^1^H and ^13^C NMR, Table 2; HRESIMS *m*/*z* 353.1234 [M + H]^+^ (calcd for C_17_H_21_O_8_, [M+ H]^+^= 353.1231).

#### Perenniporide G (**20**)

Pale yellow, amorphous powder. [α]_D_^20^ −23.5 (c 0.1, MeOH); UV (MeOH) λ_max_ (log ε) 222 (+1.5), 340 (+4.7), nm;^1^H and ^13^C NMR, Table 2; HRESIMS *m*/*z* 353.1226 [M + H]^+^ (calcd for C_17_H_21_O_8_, [M+ H]^+^= 353.1231).

#### Perenniporide H (**21**)

Pale yellow powder. [α]_D_^20^ −56.8 (c 0.1, MeOH); UV (MeOH) λ_max_ (log ε) 225 (3.5), 240 (4.0), 290 (2.5), 335 (0.8) nm;^1^H and ^13^C NMR, Table 2; HRESIMS *m*/*z* 381.1533 [M + H]^+^ (calcd for C_19_H_25_O_8_, [M + H]^+^= 381.1544).

### Computational Methods

3D models of compound structures (i.e., **18**-**21**) were built and minimized using Spartan’10 software (Wave function Inc., CA, USA). Conformational analysis was performed using the MMFF94 (Merck Molecular Force Field) force field with the Monte Carlo search protocol. The resulting conformers under the 5 kcal/mol cutoff were checked for duplicates, and redundant conformers sharing equivalent energies were removed. Then, geometry and frequency calculations were performed on the selected conformers using the density functional theory (DFT) method at the B3LYP/6–31G­(d) level of theory in the gas phase. Then, conformer optimizations were carried out in methanol using the IEFPCM (Integral Equation Formalism Polarizable Continuum Model) solvation model at the B3LYP/6–311+G­(2d,p) level of theory. The time-dependent DFT (TDDFT) method at the CAM-B3LYP/Def2-TZVP level of theory in methanol was employed for ECD calculations.^64^ The calculated excitation energy (nm) and rotatory strength (R) in dipole velocity (R_vel_) and dipole length (R_len_) forms were used to simulate ECD curves using SpecDis 1.71 Software^65^ with a half-bandwidth from 0.2 to 0.3 eV and a hypsochromic UV correction of 10 to 15 nm. Optical rotations were calculated using the B3LYP/6–31+G­(d,p) spectrometer with the IEFPCM solvation model in methanol at 589.3 nm. All calculations were performed employing the Gaussian’16 program package (Gaussian, Inc.).^66^ Energies and Boltzmann distribution, along with XYZ coordinates of all the calculated conformers, are shown in Tables S6–S29. The final ECD spectra were generated by Boltzmann-weighted averaging of the individual conformers based on their relative Gibbs free energies. The conformers selected for comparison exhibited Cotton effects that were consistent with the experimental spectra, reinforcing the proposed configurations. The optical rotation, NMR coupling constants, and NOE correlations provided high confidence in the assignments.

### Cytotoxicity Assay

Compounds **1**-**21** were evaluated for cytotoxicity using methods described previously^6^ against human melanoma cancer cells MDA-MB-435 and human ovarian cancer cells OVCAR3, both of which were purchased from the American Type Culture Collection (Manassas, VA). The cell lines were propagated at 37 °C in 5% CO_2_ in RPMI 1640 medium supplemented with fetal bovine serum (10%), penicillin (100 units/mL), and streptomycin (100 μg/mL). Cells in the long phase growth were harvested by trypsinization followed by two washings to remove all traces of enzyme. A total of 5,000 cells were seeded per well of a 96-well clear, flat-bottom plate (Microtest 96, Falcon) and incubated overnight (37 °C in 5% CO_2_). Samples dissolved in DMSO were then diluted and added to the appropriate wells. The cells were incubated in the presence of a test substance in three technical replicates for 72 h at 37 °C and evaluated for viability with a commercial absorbance assay (CellTiter-Blue Cell Viability Assay, Promega Corp) that measured viable cells. IC_50_ values were measured using nonlinear regression analysis in GraphPad prism software. The values are representative of three biological replicates that are expressed in μM relative to the solvent (DMSO) control; taxol (paclitaxel) was used as a positive control.

## Supplementary Material



## Data Availability

The NMR data for compounds 1–21 were deposited in the NP-MRD (https://np-mrd.org/) under accession numbers: NP0138212, NP0351624, NP0351625, NP0351626, NP0351627, NP0022925, NP0351628, NP0351629, NP0351630, NP0351631, NP0351632, NP0351633, NP0351634, NP0010917, NP0010918, NP0010919, NP0010920, NP0351635, NP0351636, NP0351637, NP0351638. The sequence data for the fungus, *Pyrenochaetopsis* sp. (strain MSX63699), were deposited in GenBank (ITS: PV962929).

## Notes

Purity of **1**–**21** via UPLC; HRESIMS spectra and 1D (^1^H, ^13^C) and 2D (COSY, HMBC, HSQC, and NOESY) NMR data for the new compounds (i.e., **11**–**13**, **18**–**21**); 1D NMR and ECD spectra for the known compounds (i.e., **1**–**10**, **14**–**17**); energies, Boltzmann distributions, and XYZ coordinates for the calculated conformers for **18**–**21**; phylogram of the most likely tree for strain MSX63699; proposed biosynthesis of perenniporides A-H (i.e., **14**–**21**); summary of key structure–activity relationship trends and SMILES, InChI, and InChIKey descriptors for **1**–**21** (PDF)
