# Supplementary material for: Beyond Monomers: Discovery of Bioactive Naphthoquinone Heterodimers and Perenniporides from Pyrenochaetopsis Species
Source: J Nat Prod. 2025 Dec 2;88(12):2968–77. doi: 10.1021/acs.jnatprod.5c01209 (PMC12751106; doi:10.1021/acs.jnatprod.5c01209)
Supplement: Supplementary file 1 [file np5c01209_si_001.pdf]

Supporting Information

Beyond Monomers: Discovery of Bioactive Naphthoquinone Heterodimers and Perenniporides from *Pyrenochaetopsis* sp.

Reema A. Al-Qiam,[1] Manuel Rangel-Grimaldo,[1,2] Isabel M. Chauvin,[3] Elizabeth N. Kaweesa,[3] Manead Khin,[3] Huzefa A. Raja,[1] Jimmy Orjala,[3] Joanna E. Burdette,[3] Cedric J. Pearce,[4] and Nicholas H. Oberlies[1,]*

1 Department of Chemistry and Biochemistry, University of North Carolina at Greensboro, Greensboro, North Carolina 27402, United States.

2 Instituto de Química, Universidad Nacional Autónoma de México, Ciudad de México 04510, México.

3 Department of Pharmaceutical Sciences, University of Illinois at Chicago, Chicago, Illinois 60607, United States.

4 Mycosynthetix, Inc., Hillsborough, North Carolina 27278, United States.

*Correspondence: Nicholas H. Oberlies (n_oberli@uncg.edu).

Table of Content:

Table of Content: 2

Figure S1: UPLC chromatograms of compounds 1–21 (PDA detection), demonstrating >97% purity. All data were acquired via a Waters UPLC system with a BEH C_{18} (1.7 μm 2.1 × 50 mm) column and a MeCN–H_2O (0.1% formic acid) gradient that increases linearly from 15:85 to 100:00 of MeCN over 10 mins. Purity shown using full PDA range .... 6 &7

Figure S2: ^{1}H and ^{13}C NMR spectra for compound 1 ($CDCl_3$, 400 and 100 MHz, respectively). 8

Figure S3: ^{1}H and ^{13}C NMR spectra for compound 2 ($CDCl_3$, 400 and 100 MHz, respectively). 9

Figure S4: ^{1}H and ^{13}C NMR spectra for compound 3 ($CDCl_3$, 400 and 100 MHz, respectively). 10

Figure S5: ^{1}H and ^{13}C NMR spectra for compound 4 ($CDCl_3$, 400 and 100 MHz, respectively). 11

Figure S6: ^{1}H and ^{13}C NMR spectra for compound 5 ($CDCl_3$, 400 and 100 MHz, respectively). 12

Figure S7: ^{1}H and ^{13}C NMR spectra for compound 6 ($CDCl_3$, 400 and 100 MHz, respectively). 13

Figure S8: ^{1}H and ^{13}C NMR spectra for compound 7 ($CDCl_3$, 400 and 100 MHz, respectively). 14

Figure S9: ECD spectra for compounds 2, 4, 5, and 7 in CH_3OH at a concentration of 0.04, 0.10, 0.02, and 0.02 mg/mL, respectively. 15

Figure S10: Structures of the five heterodimeric naphthoquinones that have been described from fungi, including the names of the fungal strain. 16

Figure S11: Monomeric building units of compounds 8-13. All except 13 have the same subunit A (which is also compound 1, isolated as the major compound). Their diversity is mainly in subunit B (mainly positions 2' and 6'). Compound 13 could be assembled from two identical monomers that are not observed from the extract, and this might explain why it has the lowest yield (only 0.7 mg). 17

Figure S12: ^{1}H and ^{13}C NMR spectra for compound 8 ($CDCl_3$, 400 and 100 MHz, respectively). 18

Figure S13: ^{1}H and ^{13}C NMR spectra for compound 9 ($CDCl_3$, 400 and 100 MHz, respectively). 19

Figure S14: ^{1}H and ^{13}C NMR spectra for compound 10 ($CDCl_3$, 400 and 100 MHz, respectively). 20

Figure S15: ECD spectra for compounds 8-13 in CH_3OH at a concentration of 0.10-0.14 mg/mL. 21

Figure S16: HRESIMS data for compound 11 22

Figure S17: ^{1}H and ^{13}C NMR spectra for compound 11 ($CDCl_3$, 500 and 125 MHz, respectively) 23

Figure S18: COSY spectrum for compound 11 ($CDCl_3$, 500 MHz). 24

Figure S19: Edited HSQC spectrum of compound 11 ($CDCl_3$, 500 MHz). 25

Figure S20: HMBC spectrum of compound 11 ($CDCl_3$, 500 MHz) 26

Figure S21: HRESIMS data for compound 12. 27

Figure S22: ^{1}H and ^{13}C NMR spectra for compound 12 ($CDCl_3$, 400 and 100 MHz, respectively). 28

Figure S23: COSY spectrum for compound 12 ($CDCl_3$, 400 MHz). 29

Figure S24: Edited HSQC spectrum of compound 12 ($CDCl_3$, 400 MHz). 30

Figure S25: HMBC spectrum of compound 12 ($CDCl_3$, 400 MHz). 31

Figure S26: HRESIMS data for compound 13. 32

Figure S27: ^{1}H and ^{13}C NMR spectra for compound 13 ($CDCl_3$, 700 and 175 MHz, respectively). 33

Figure S28: COSY spectrum for compound 13 ($CDCl_3$, 700 MHz). 34

Figure S29: Edited HSQC spectrum of compound 13 ($CDCl_3$, 700 MHz). 35

Figure S30: HMBC spectrum of compound 13 ($CDCl_3$, 700 MHz). 36

Figure S31: ^{1}H and ^{13}C NMR spectra for perenniporide A (14) (Acetone-d_6, 400 and 100 MHz, respectively)........37

Figure S32: ^{1}H and ^{13}C NMR spectra for perenniporide B (15) (DMSO-d_6, 400 and 100 MHz, respectively)........38

Figure S33: ^{1}H and ^{13}C NMR spectra for perenniporide C (16) ($CDCl_3$, 400 and 100 MHz, respectively). 39

Figure S34: ^{1}H and ^{13}C NMR spectra for perenniporide D (17) (DMSO-d_6, 400 and 100 MHz, respectively). 40

Figure S35: ECD spectra for compounds 14-17 in CH_3OH at a concentration of 0.01, 0.02, 0.02, and 0.01 mg/mL, respectively. 41

Figure S36: HRESIMS data for compound 18. 42

Figure S37: ^{1}H and ^{13}C NMR spectra for perenniporide E (18) (Acetone-d_6, 500 and 125 MHz, respectively)........43

Figure S38: COSY spectrum for perenniporide E (18) (Acetone-d_6, 500 MHz). 44

Figure S39: Edited HSQC spectrum for perenniporide E (18) (Acetone-d_6, 500 MHz)........45

Figure S40: HMBC spectrum of perenniporide E (18) (Acetone-d_6, 500 MHz)........46

Figure S41: HRESIMS data for compound 19. 47

Figure S42: ^{1}H and ^{13}C NMR spectra for perenniporide F (19) (CD_3OD, 500 and 125 MHz, respectively). 48

Figure S43: COSY spectrum for perenniporide F (19) (CD_3OD, 500 MHz). 49

Figure S44: Edited HSQC spectrum for perenniporide F (19) (CD_3OD, 500 MHz). 50

Figure S45: HMBC spectrum of perenniporide F (19) (CD_3OD, 500 MHz). 51

Figure S46: Stacked ^{13}C spectra NMR of 19 (top) and 15 (bottom), (CD_3OD, 125 MHz). 52

Figure S47: HRESIMS data for compound 20. 53

Figure S48: ^{1}H and ^{13}C NMR spectra for perenniporide G (20) ($CDCl_3$, 500 and 125 MHz, respectively). 54

Figure S49: COSY spectrum for perenniporide G (20) ($CDCl_3$, 500 MHz). 55

Figure S50: Edited HSQC spectrum for perenniporide G (20) ($CDCl_3$, 500 MHz)........56

Figure S51: HMBC spectrum of perenniporide G (20) ($CDCl_3$, 500 MHz)........57

Figure S52: NOESY spectrum for perenniporide G (20) ($CDCl_3$, 500 MHz)........58

Figure S53: Four possible conformers of 20 (A-D; left). Minimized structure of 20A, showing the key NOESY correlation (blue arrow) and the distance between 3-OCH_3 and H-12, as they are on the same face (right). 59

Table S1: Calculated vs. experimental optical rotation of compound 20. .... **60**

Table S2: Summary of absolute configuration determination (ECD_{calc}, OR_{calc} and $NOESY_{exp}$) of compound 20. .... **60**

Figure S54: HRESIMS data for compound 21. .... **61**

Figure S55: ^{1}H and ^{13}C NMR spectra for perenniporide H (21) ($CDCl_3$, 500 and 125 MHz, respectively). **62**

Figure S56: COSY spectrum for perenniporide H (21) ($CDCl_3$, 500 MHz). **63**

Figure S57: Edited HSQC spectrum for perenniporide H (21) ($CDCl_3$, 500 MHz) **64**

Figure S58: HMBC spectrum of perenniporide H (21) ($CDCl_3$, 500 MHz) **65**

Figure S59: NOESY spectrum for perenniporide H (21) ($CDCl_3$, 500 MHz) **66**

Figure S60: Four possible conformers of 21 (A-D; left). Minimized structure of 21B, showing the key NOESY correlation (blue arrow) and the distance between 3-OCH_3 and H-12, as they are on the same face (right). **67**

Table S3: Calculated vs. experimental optical rotation of compound 21. .... **68**

Table S4:Summary of absolute configuration determination (ECD_{calc}, OR_{calc} and $NOESY_{exp}$) of compound 21. .... **68**

Figure S61: Stacked ^{1}H NMR spectra of 16 incubated with 0.1% formic acid after 3, 6, 24, and 48 hours ($CDCl_3$, 400 MHz) **69**

Figure S62: Proposed biosynthesis of perenniporides A-H (*i.e.,* compounds 14-21). **70**

Figure S63: Chromatographic separation of compound 19 after one week in an NMR tube in $CDCl_3$, indicating partial conversion to compound 14 (top). Stacked ^{1}H NMR spectra of 19 before and after purification ($CDCl_3$, 400 MHz) (bottom). **71**

Figure S64: Phylogram of the most likely tree (-lnL = 1627.382) from an IQ-TREE analysis v2.2.0 of 26 taxa based on ITS data (547 bp) under Numbers refer to UFBoot support values ≥ 90% based on 5000 replicates followed by PHYML bootstrap values. For IQ tree nodes ≥ 95% are considered strongly supported, while nodes ≥70% are considered significant for PHYML bootstrap analysis. Thickened branches indicate significant Bayesian posterior probabilities ≥ 95%. Bayesian analysis was calculated using Mr. Bayes v. 3.2 under TIM2e+G4 for ITS region. The MSX63699 strain is highlighted in bold. A 30 days old culture on malt extract agar is shown on the right. Scale bar indicates the expected number of nucleotide substitutions per site. **72**

Table S5:SMILES, InChI, and InChIkey descriptors of 1-21. .... **73 & 74**

Table S6: Energies and Boltzmann distribution of compound 18 (4*R*) **75**

Table S7: XYZ coordinates of compound 18 (4*R*). **75-78**

Table S8: Energies and Boltzmann distribution of compound 18 (4*S*). **78**

Table S9: XYZ coordinates of compound 18 (4*S*) **79-82**

Table S10: Energies and Boltzmann distribution of compound 19 (4*R*,12*R*). **82**

Table S11: XYZ coordinates of compound 19 (4*R*, 12*R*). **83-87**

Table S12: Energies and Boltzmann distribution of compound 19 (4*R*,12*S*). **88**

Table S13: XYZ coordinates of compound 19 (4*R*,12*S*). **89-94**

Table S14: Energies and Boltzmann distribution of compound 20 (3*R*,4*R*,12*R*). **94**
Table S15: XYZ coordinates of compound 20 (3*R*,4*R*,12*R*). **94**
Table S16: Energies and Boltzmann distribution of compound 20 (3*R*,4*R*,12*S*) **95**
Table S17: XYZ coordinates of compound 20 (3*R*,4*R*,12*S*) **95**
Table S18: Energies and Boltzmann distribution of compound 20 (3*R*,4*S*,12*R*) **96**
Table S19: XYZ coordinates of compound 20 (3*R*,4*S*,12*R*) **96-98**
Table S20: Energies and Boltzmann distribution of compound 20 (3*R*,4*S*,12*S*). **99**
Table S21:XYZ coordinates of compound 20 (3*R*,4*S*,12*S*). **99-101**
Table S22: Energies and Boltzmann distribution of compound 21 (3*R*,4*R*,12*R*). **102**
Table S23: XYZ coordinates of compound 21 (3*R*,4*R*,12*R*). **102-104**
Table S24: Energies and Boltzmann distribution of compound 21 (3*R*,4*R*,12*S*) **104**
Table S25: XYZ coordinates of compound 21 (3*R*,4*R*,12*S*) **104-106**
Table S26: Energies and Boltzmann distribution of compound 21 (3*R*,4*S*,12*R*) **106**
Table S27: XYZ coordinates of compound 21 (3*R*,4*S*,12*R*) **107-108**
Table S28: Energies and Boltzmann distribution of compound 21 (3*R*,4*S*,12*S*). **109**
Table S29: XYZ coordinates of compound 21 (3*R*,4*S*,12*S*). **109-112**
Table S30: Summary of key structure-activity relationship trends for compounds 1–21. .... **113**

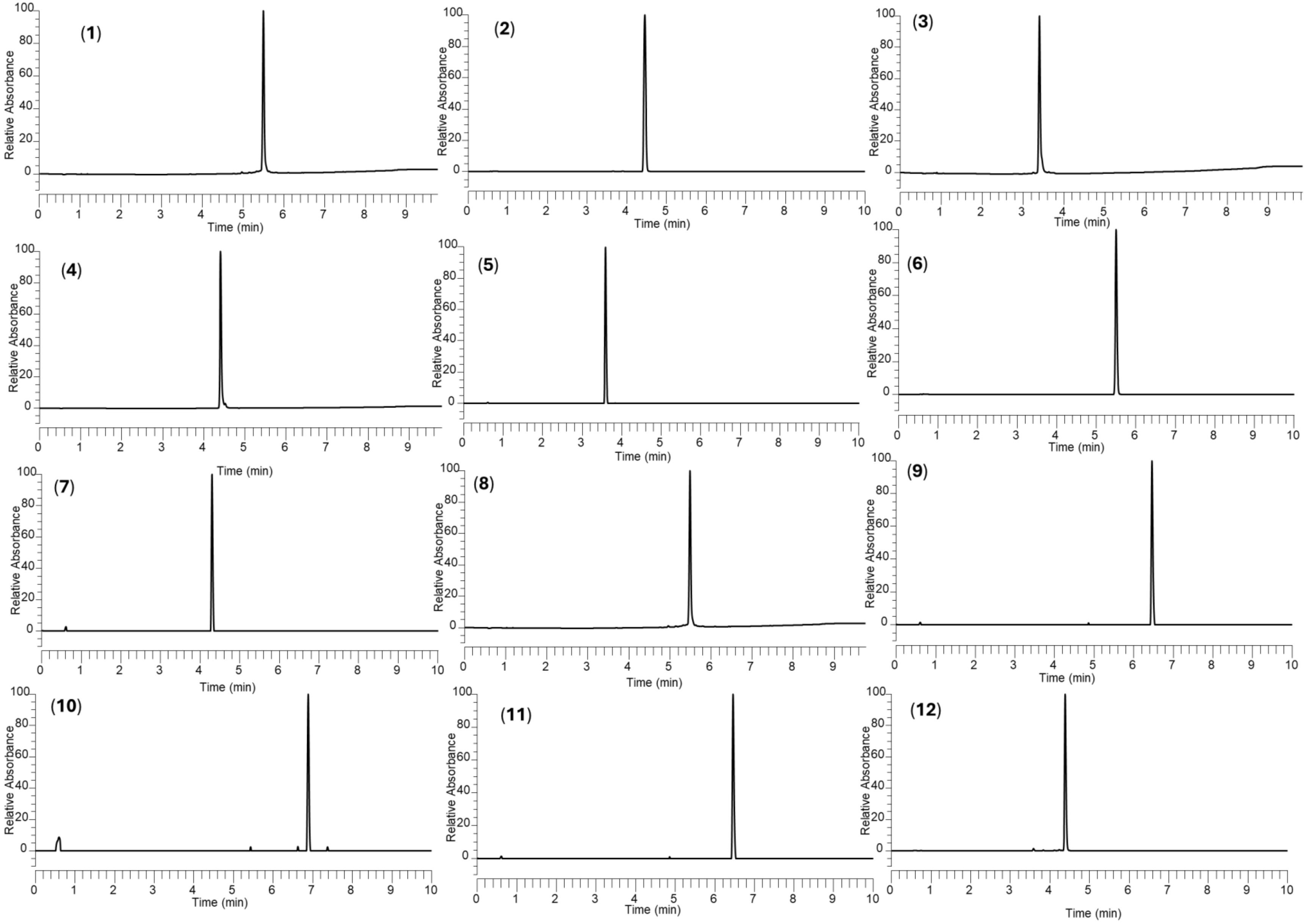
(1)
(2)
(3)
(4)
(5)
(6)
(7)
(8)
(9)
(10)
(11)
(12)
Relative Absorbance
Time (min)
0 20 40 60 80 100
0 1 2 3 4 5 6 7 8 9 10

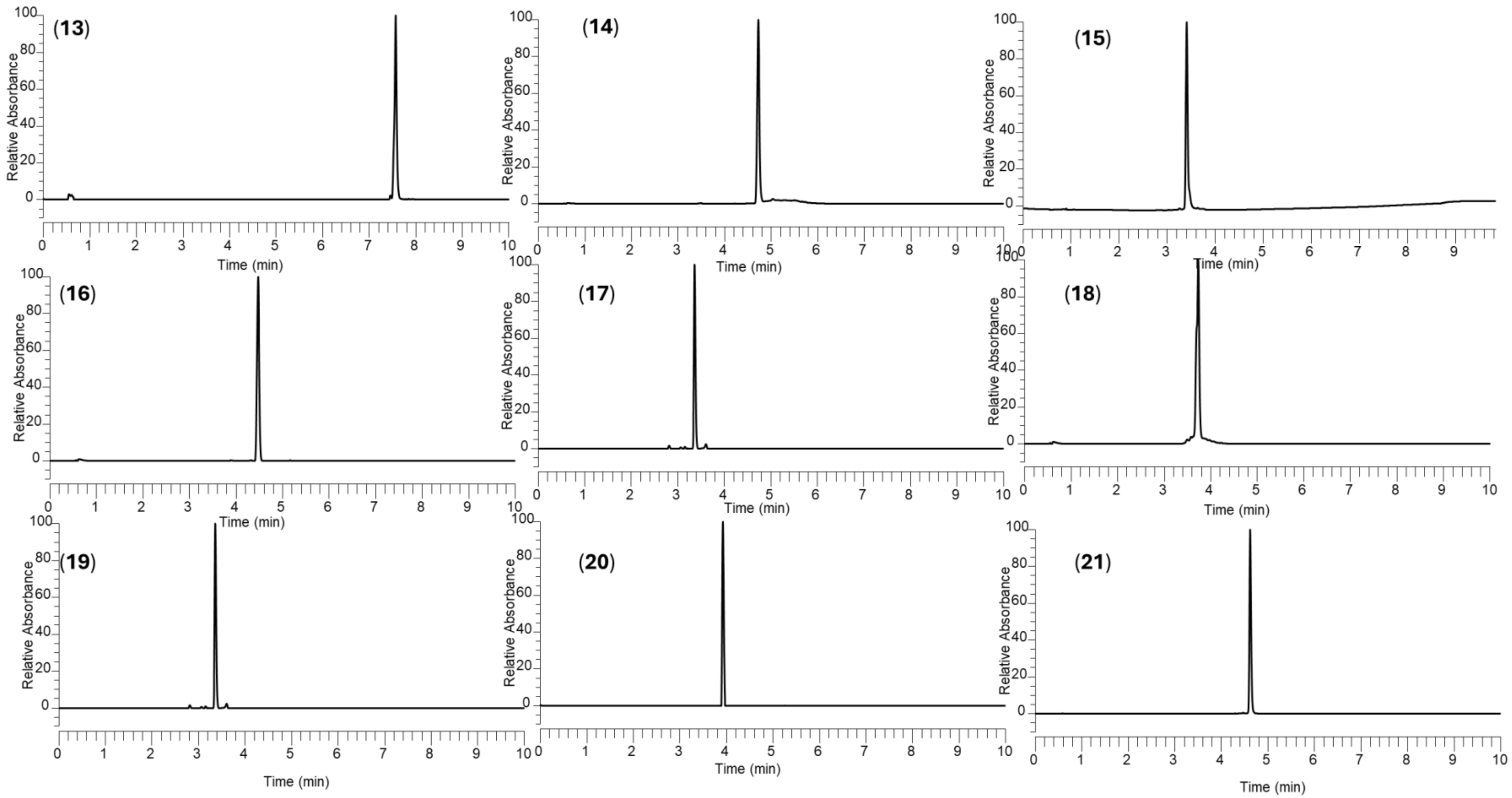

Figure S1: UPLC chromatograms of compounds **1**–**21** (PDA detection), demonstrating >97% purity. All data were acquired via a Waters UPLC system with a BEH C_{18} (1.7 μm 2.1 × 50 mm) column and a MeCN–H_2O (0.1% formic acid) gradient that increases linearly from 15:85 to 100:00 of MeCN over 10 mins. Purity shown using full PDA range.

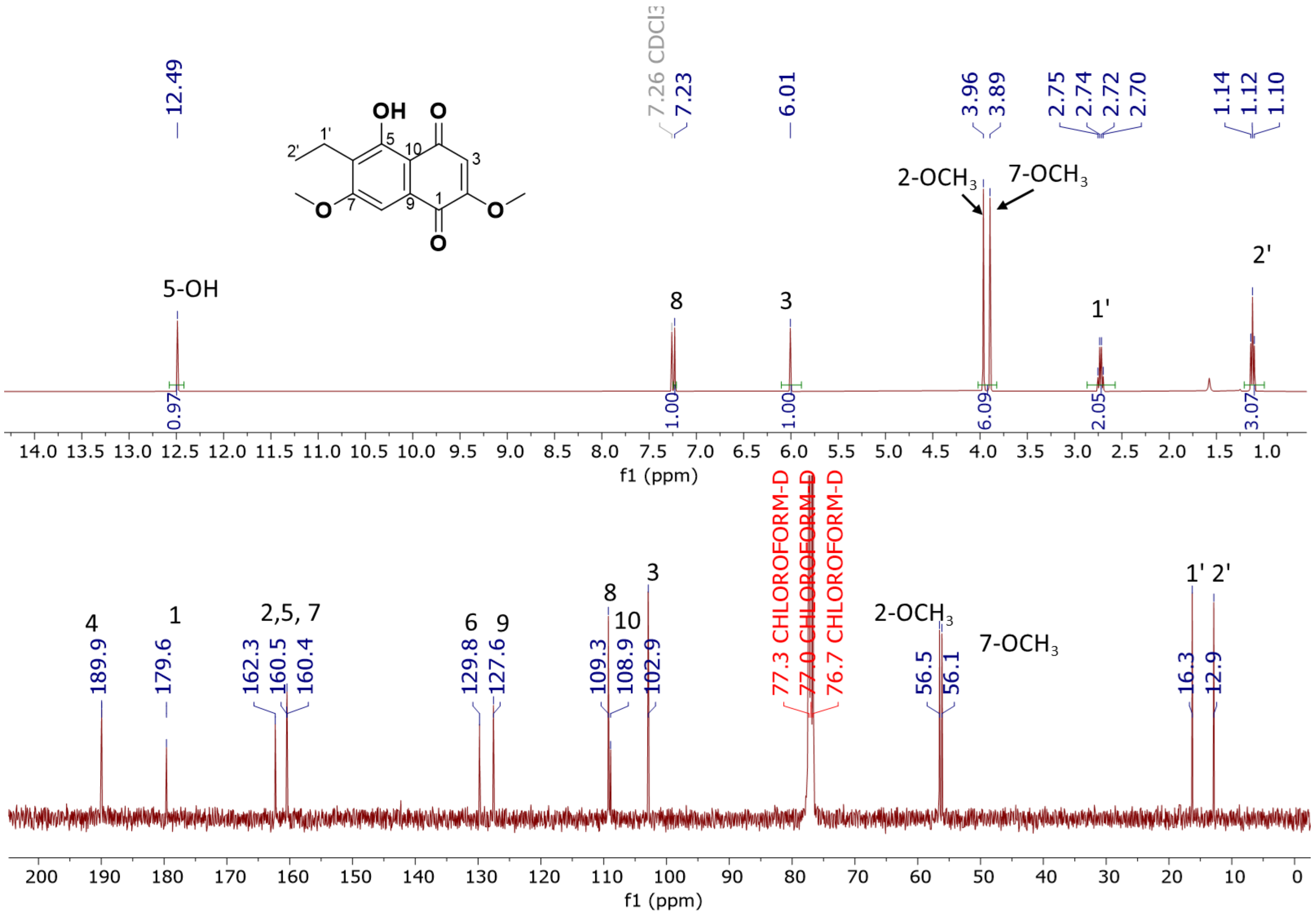

Figure S2: ^{1}H and ^{13}C NMR spectra for compound **1** ($CDCl_3$, 400 and 100 MHz, respectively).

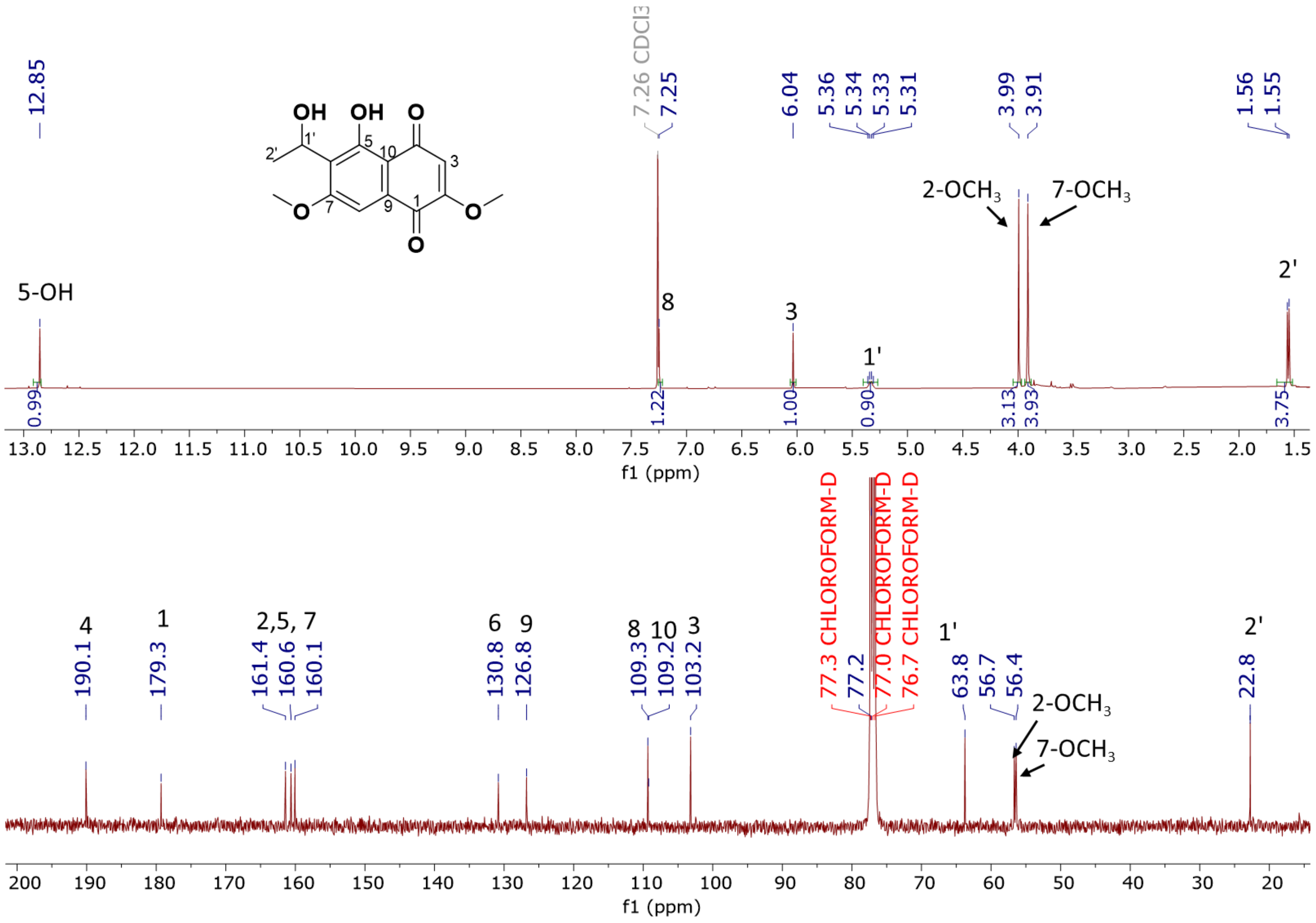

Figure S3: ^{1}H and ^{13}C NMR spectra for compound **2** ($CDCl_3$, 400 and 100 MHz, respectively).

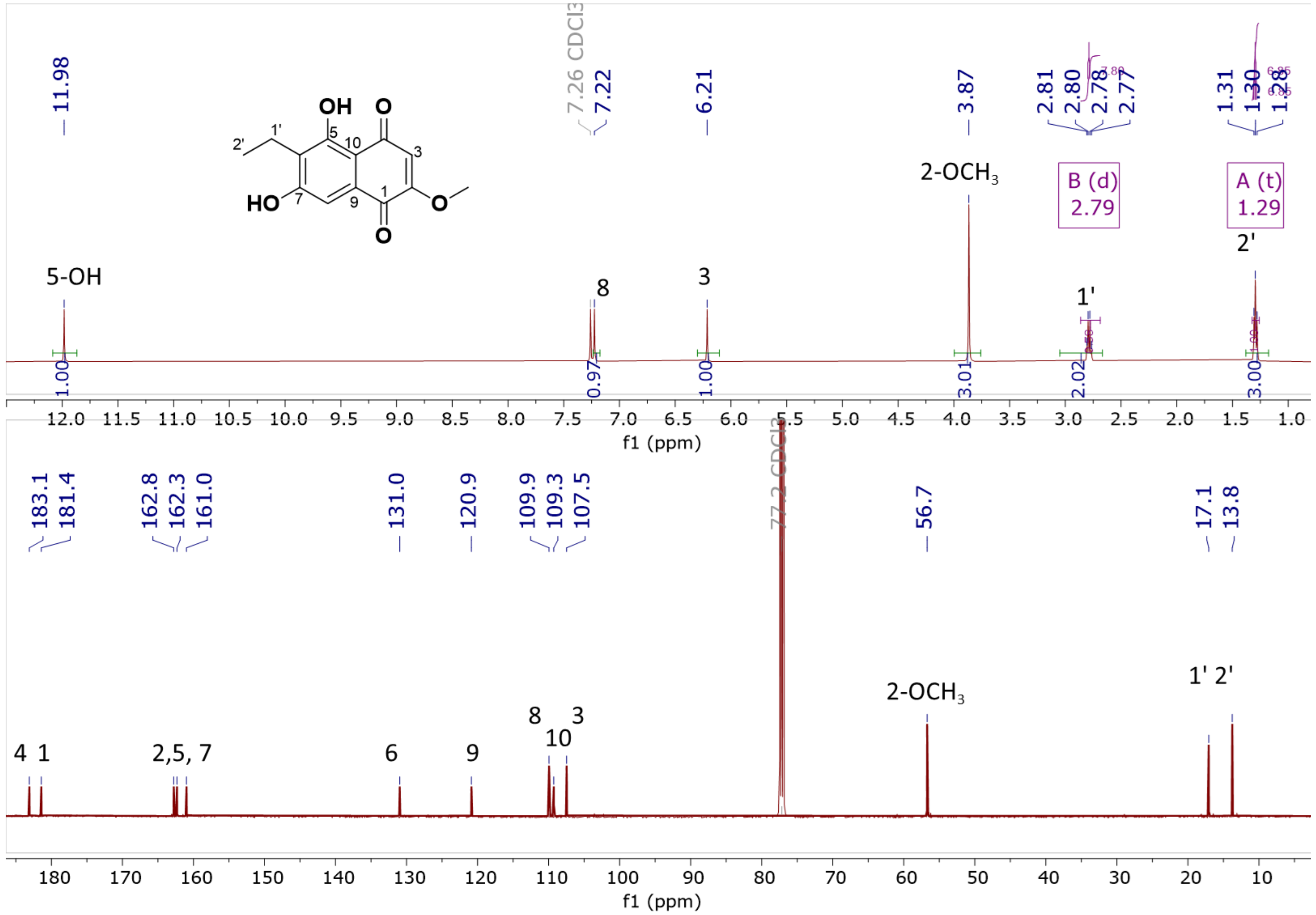

Figure S4: ^{1}H and ^{13}C NMR spectra for compound **3** ($CDCl_3$, 400 and 100 MHz, respectively).

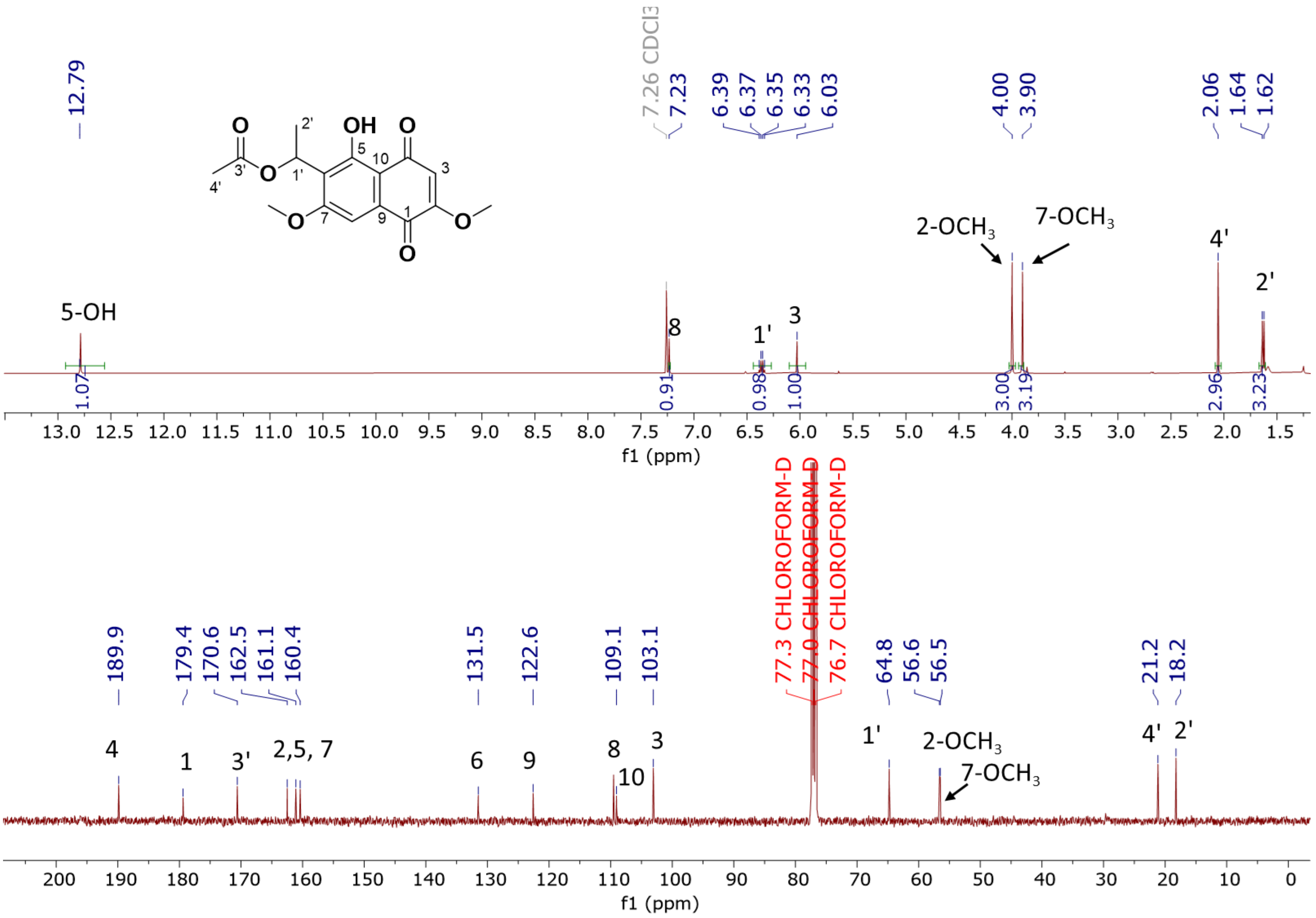

Figure S5: ^{1}H and ^{13}C NMR spectra for compound **4** ($CDCl_3$, 400 and 100 MHz, respectively).

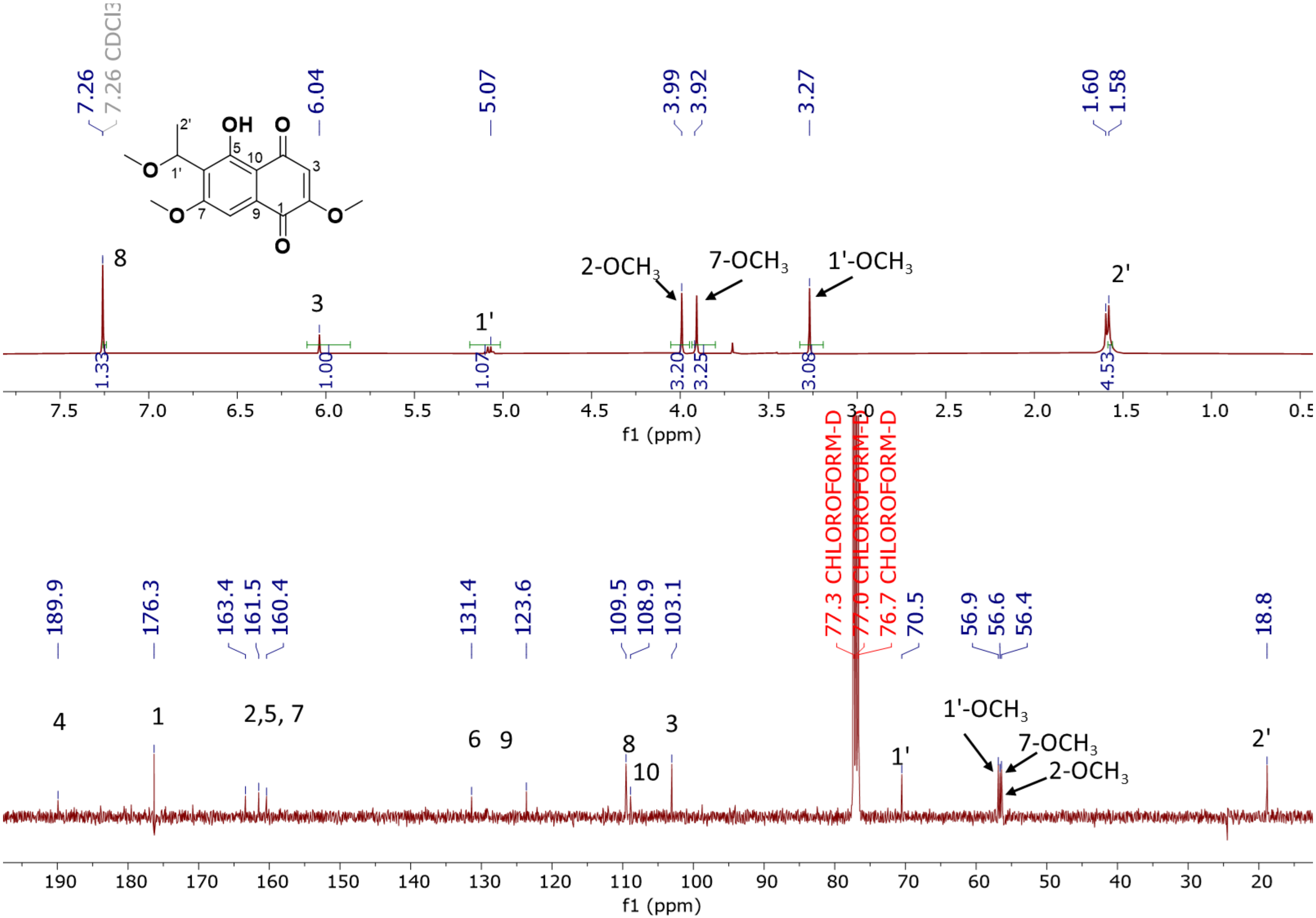

Figure S6: ^{1}H and ^{13}C NMR spectra for compound **5** ($CDCl_3$, 400 and 100 MHz, respectively).

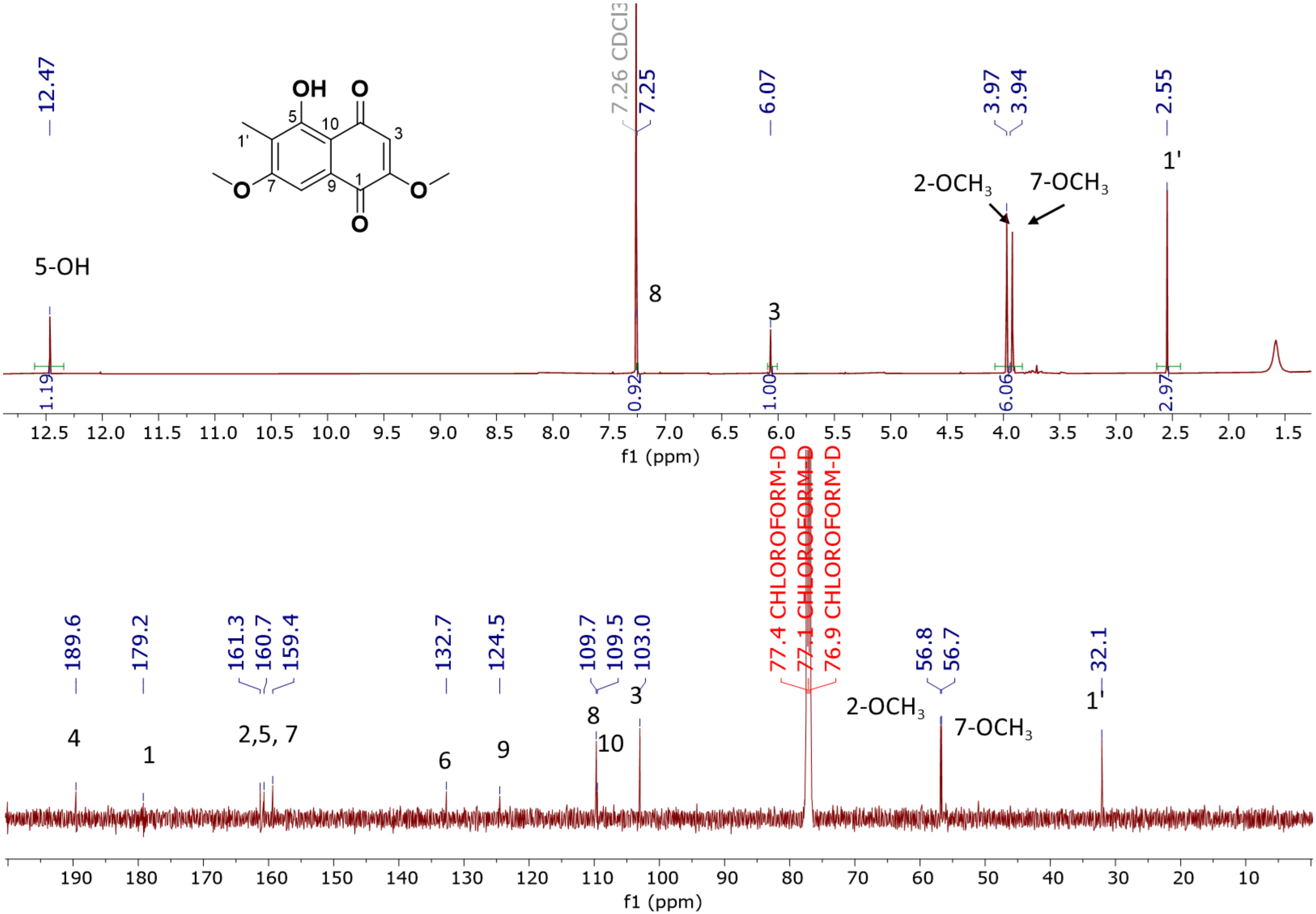

Figure S7: ^{1}H and ^{13}C NMR spectra for compound **6** ($CDCl_3$, 400 and 100 MHz, respectively).

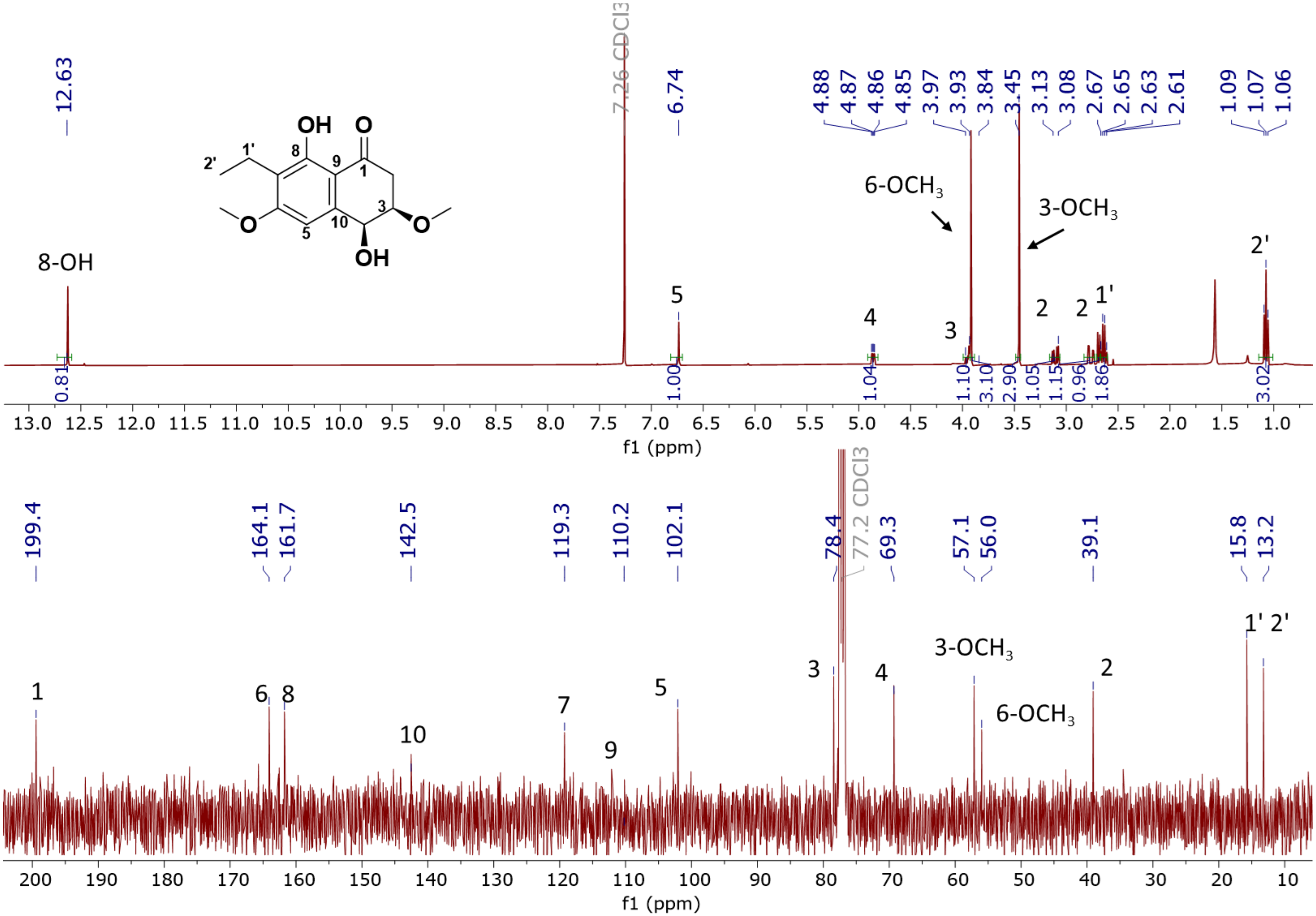

Figure S8: ^{1}H and ^{13}C NMR spectra for compound **7** ($CDCl_3$, 400 and 100 MHz, respectively).

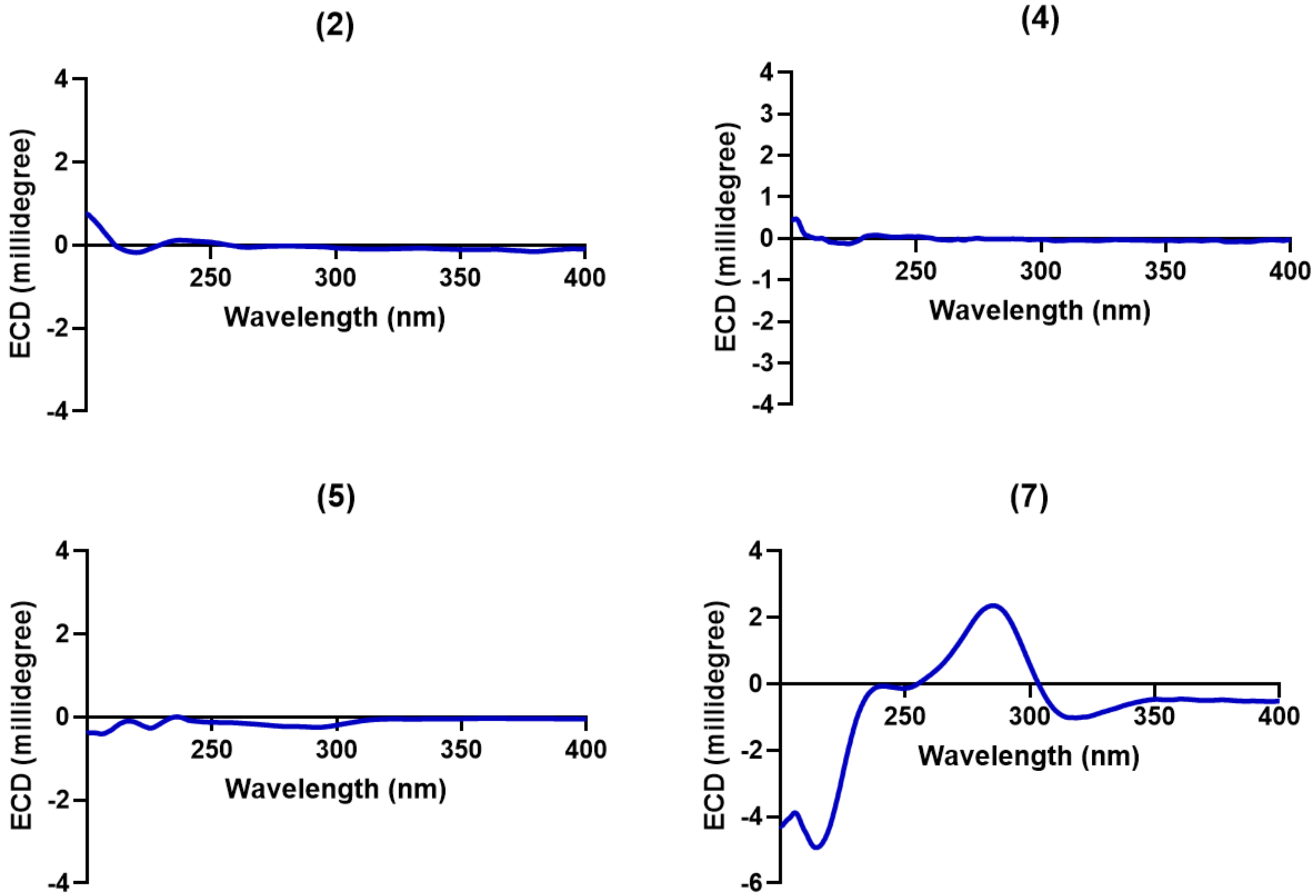

Figure S9: ECD spectra for compounds **2**, **4**, **5**, and **7** in CH_3OH at a concentration of 0.04, 0.10, 0.02, and 0.02 mg/mL, respectively.

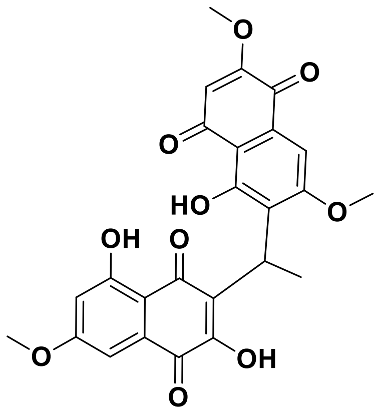

Kirschsteinin C (8)

Pyrenochaetopsis sp.

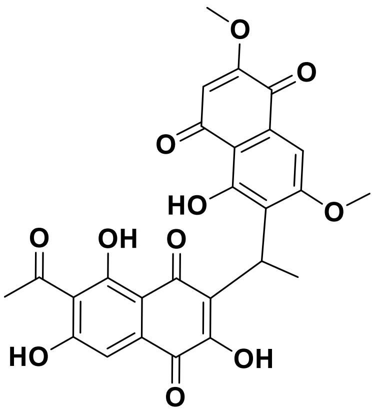

Kirschsteinin (9)

Kirschsteiniothelia sp.
Phaeosphaeria sp.
and *Pyrenochaetopsis* sp.

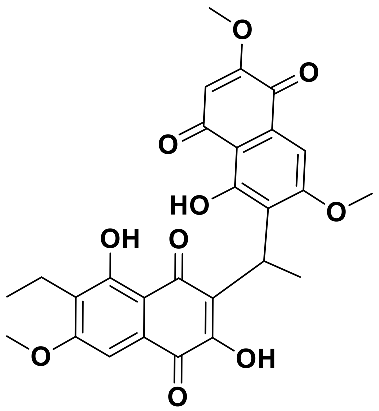

Kirschsteinin B / Neofusnaphthoquinone A (10)

Neofusicoccum australe

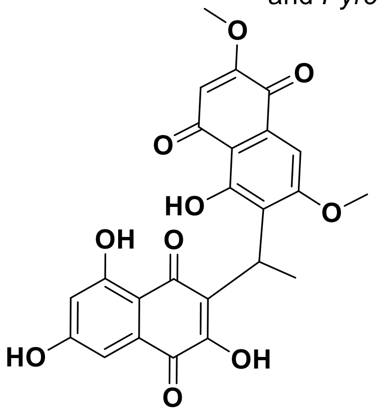

Deacetylkirschsteinin

Phaeosphaeria sp.

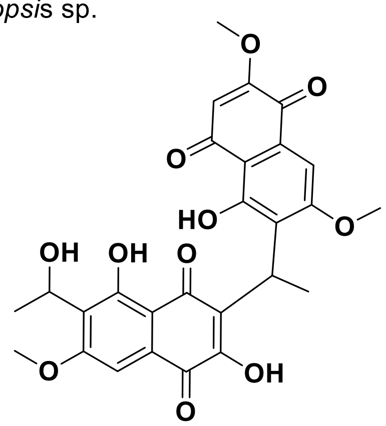

Neofusnaphthoquinone B

Neofusicoccum australe

Figure S10: Structures of the five heterodimeric naphthoquinones that have been described from fungi, including the names of the fungal strain.

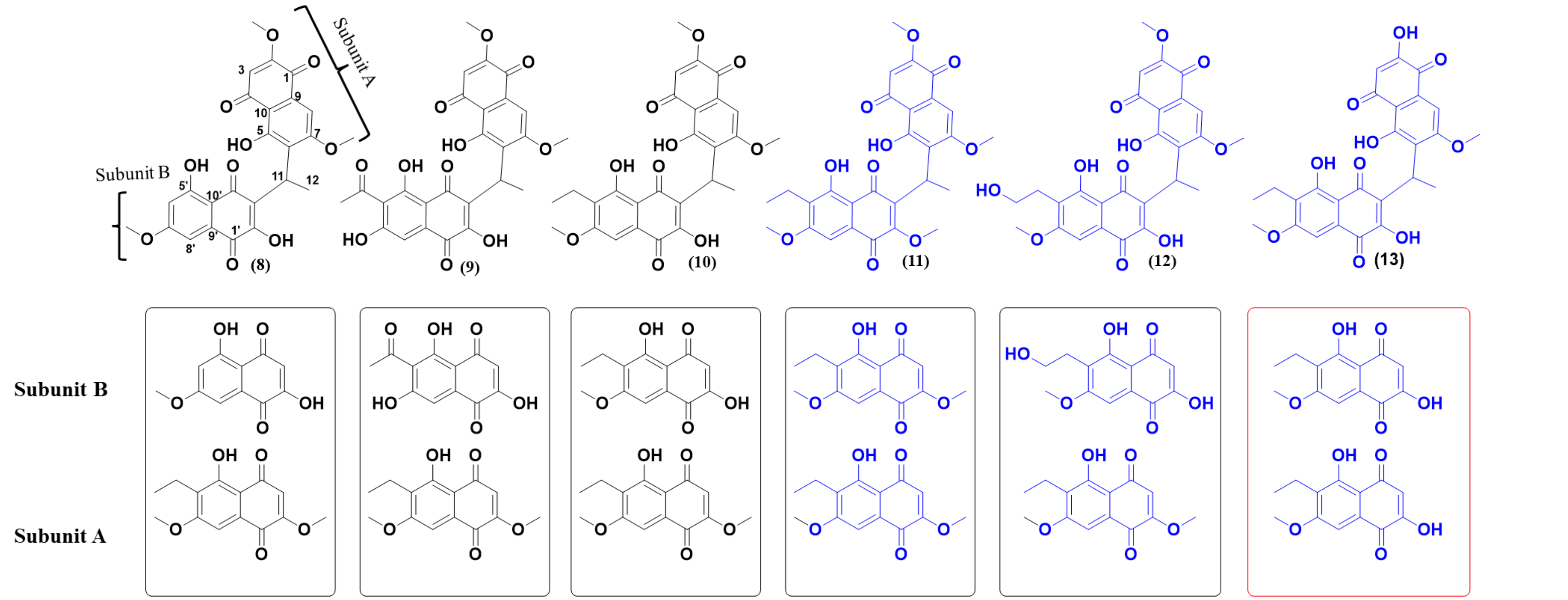

Figure S11: Monomeric building units of compounds **8**-**13**. All except **13** have the same subunit A (which is also compound **1,** isolated as the major compound). Their diversity is mainly in subunit B (mainly positions 2' and 6'). Compound **13** could be assembled from two identical monomers that are not observed from the extract, and this might explain why it has the lowest yield (only 0.7 mg).

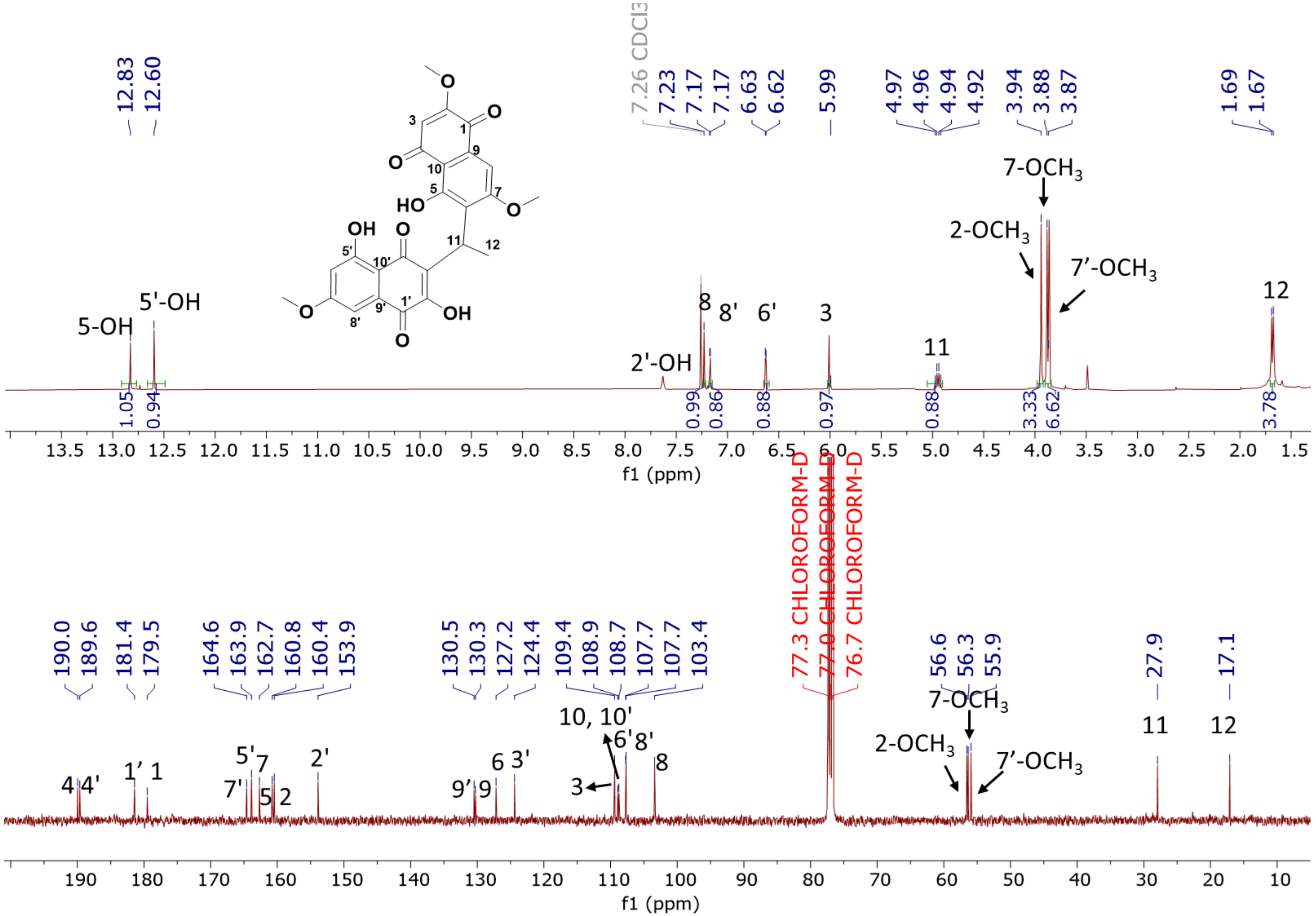

Figure S12: ^{1}H and ^{13}C NMR spectra for compound **8** ($CDCl_3$, 400 and 100 MHz, respectively).

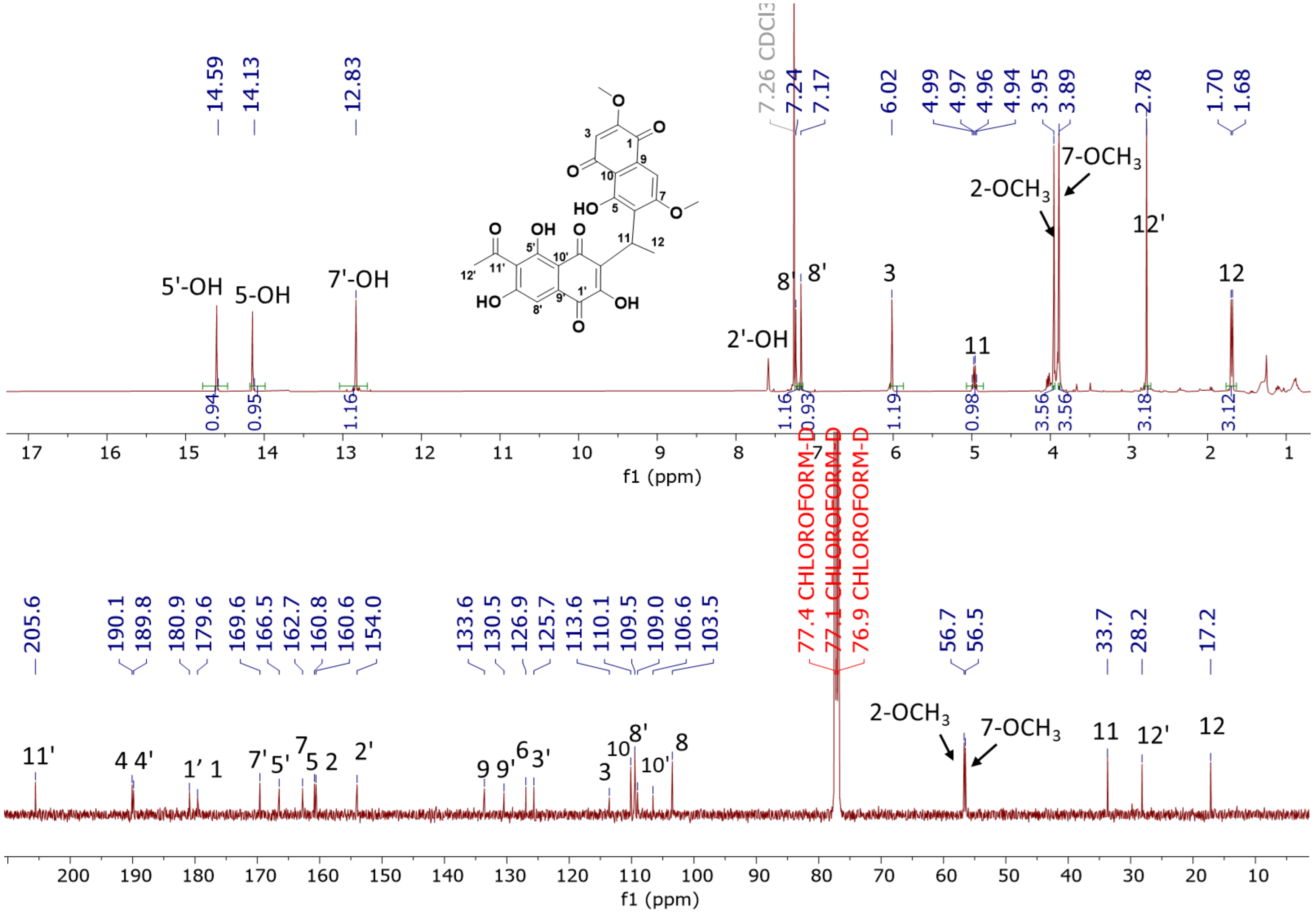

Figure S13: ^{1}H and ^{13}C NMR spectra for compound **9** ($CDCl_3$, 400 and 100 MHz, respectively).

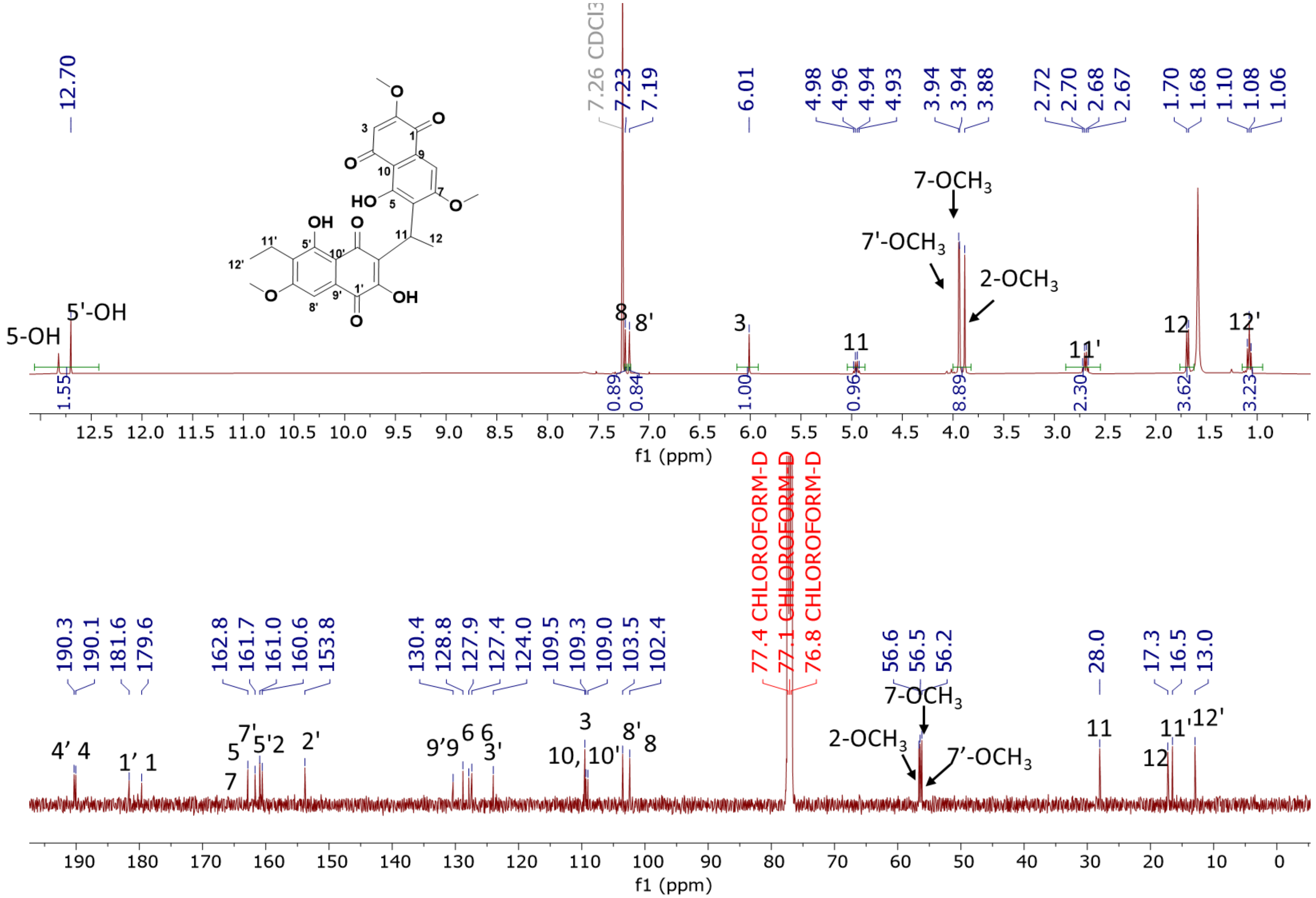

Figure S14: ^{1}H and ^{13}C NMR spectra for compound **10** ($CDCl_3$, 400 and 100 MHz, respectively).

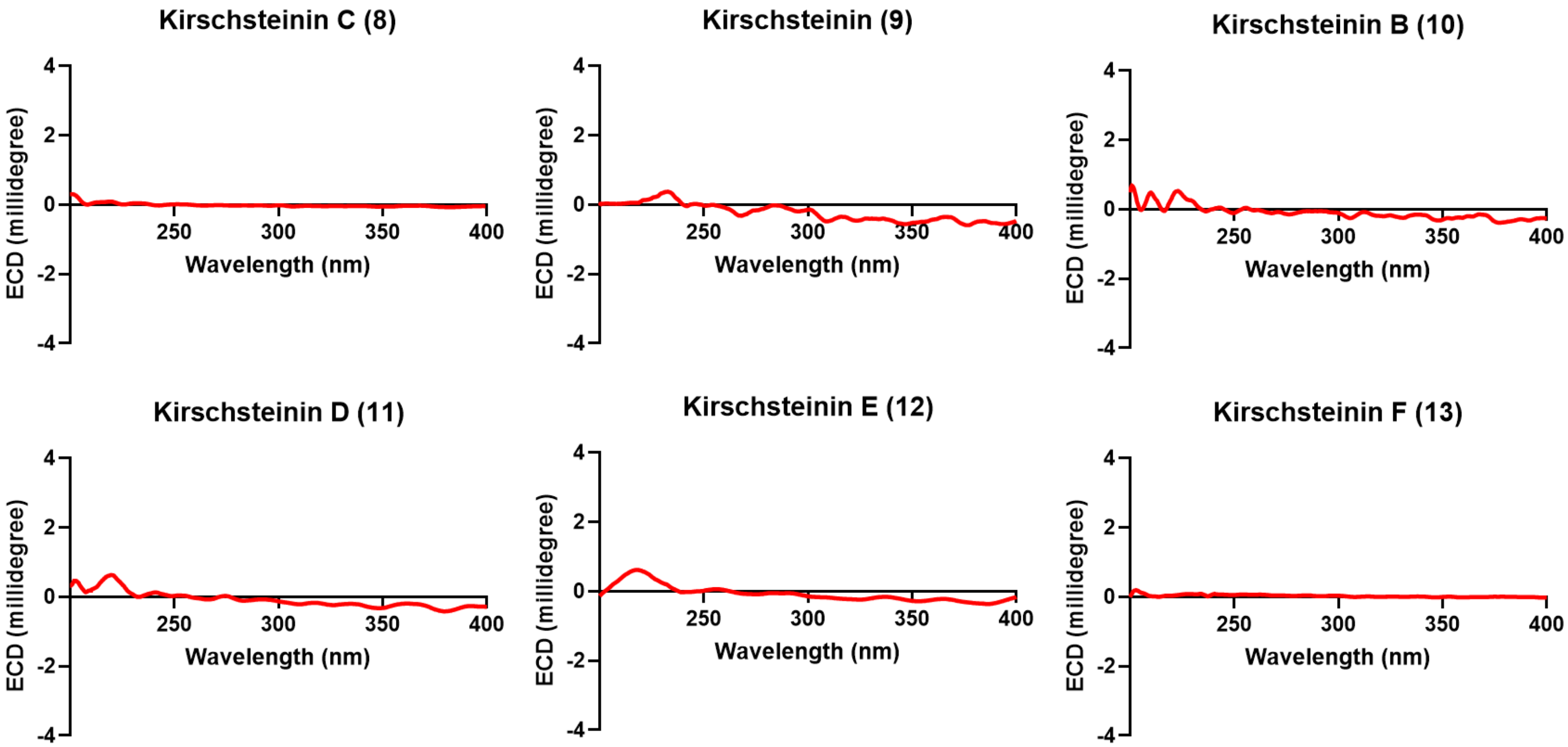

Figure S15: ECD spectra for compounds **8-13** in CH_3OH at a concentration of 0.10-0.14 mg/mL.

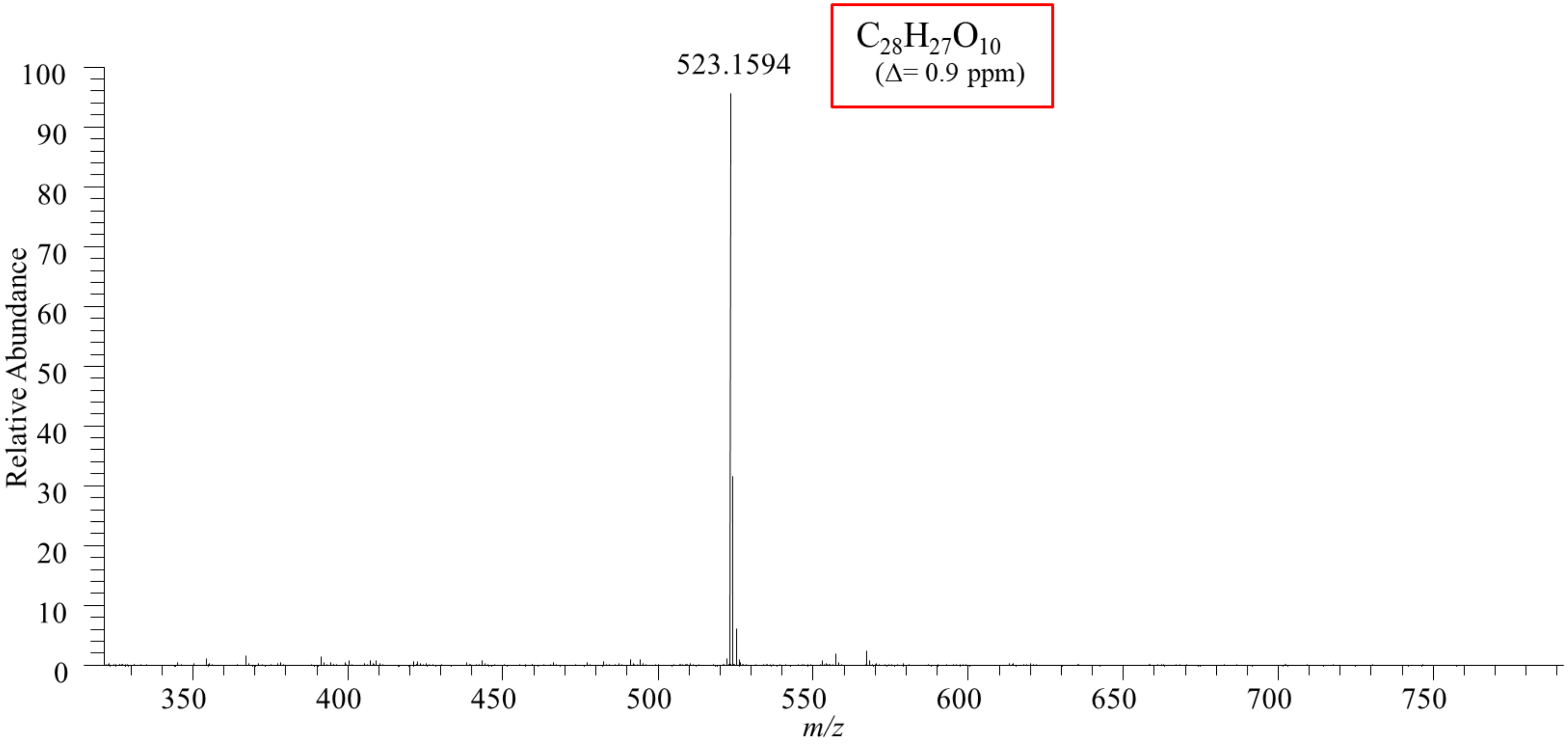

Figure S16: HRESIMS data for compound **11**.

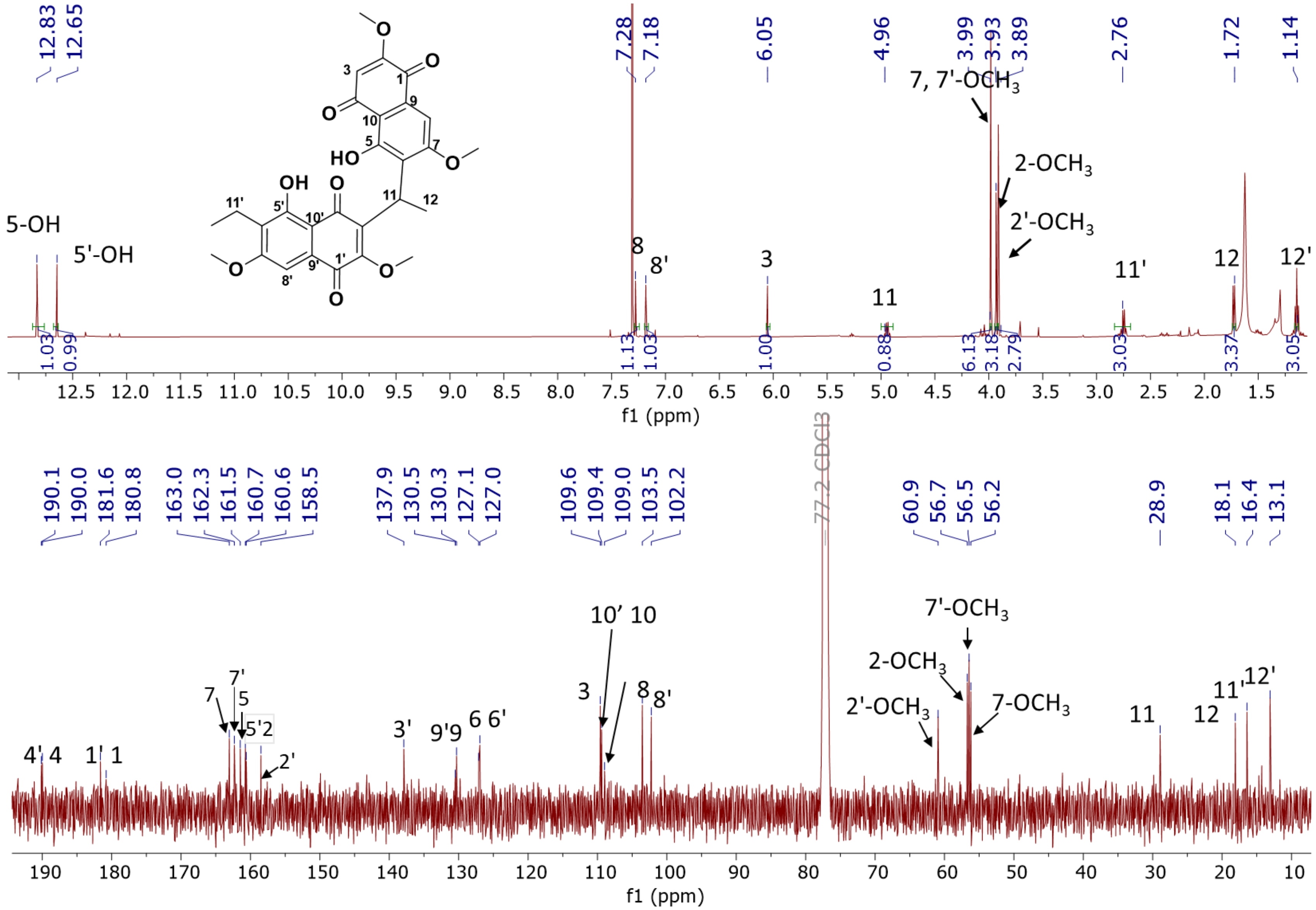

Figure S17: ^{1}H and ^{13}C NMR spectra for compound **11** ($CDCl_3$, 500 and 125 MHz, respectively).

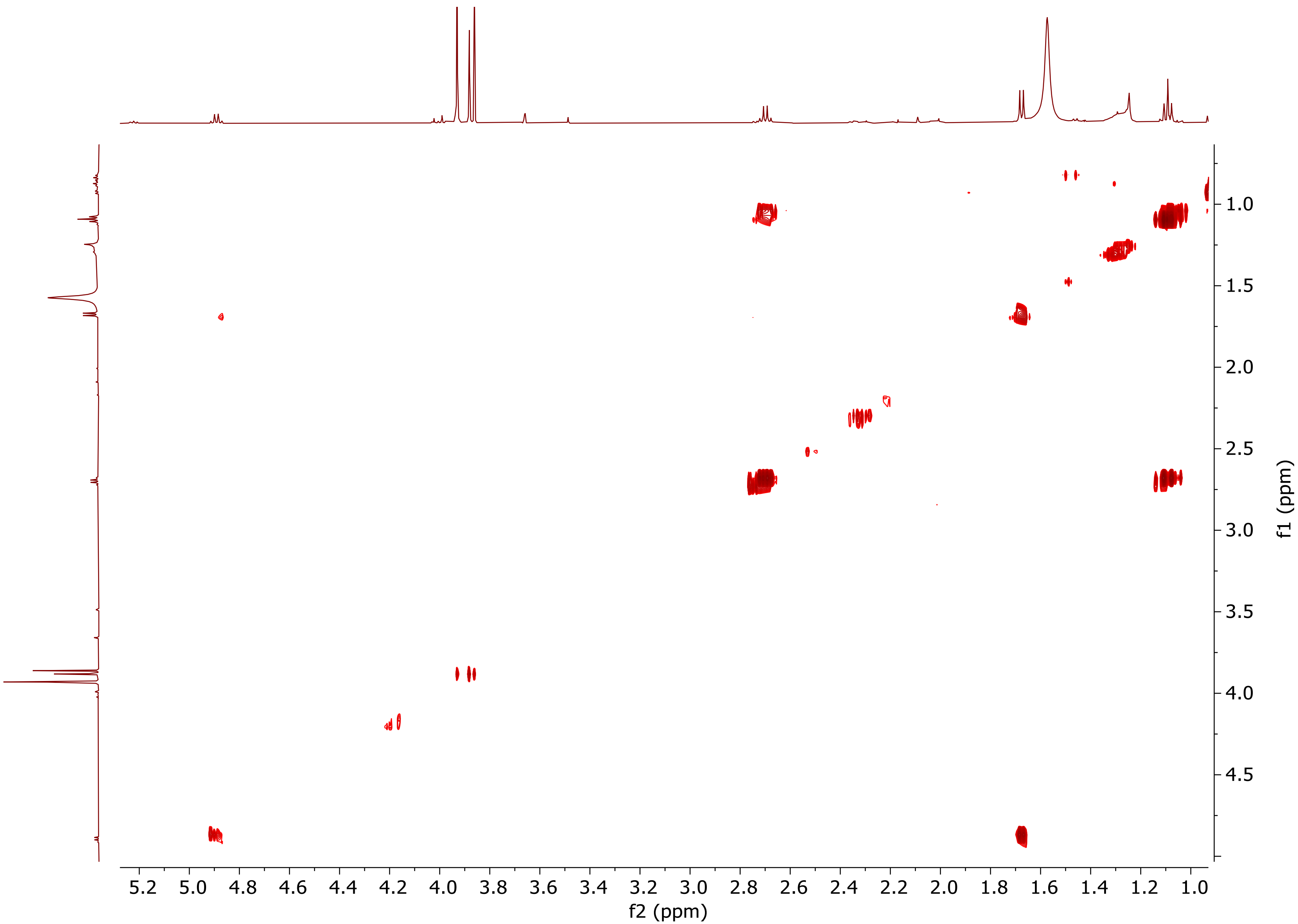

Figure S18: COSY spectrum for compound **11** ($CDCl_3$, 500 MHz).

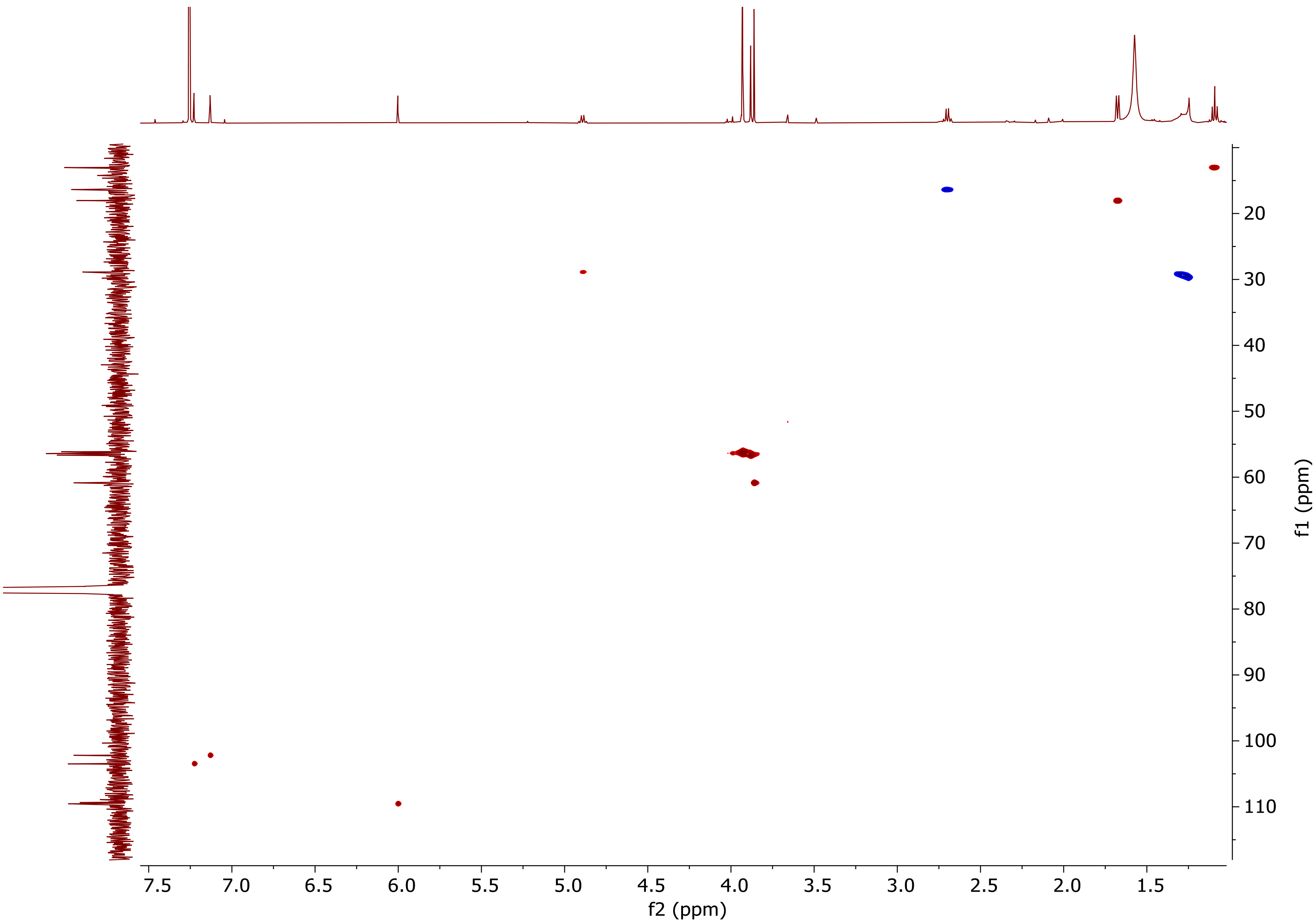

Figure S19: Edited HSQC spectrum of compound **11** ($CDCl_3$, 500 MHz).

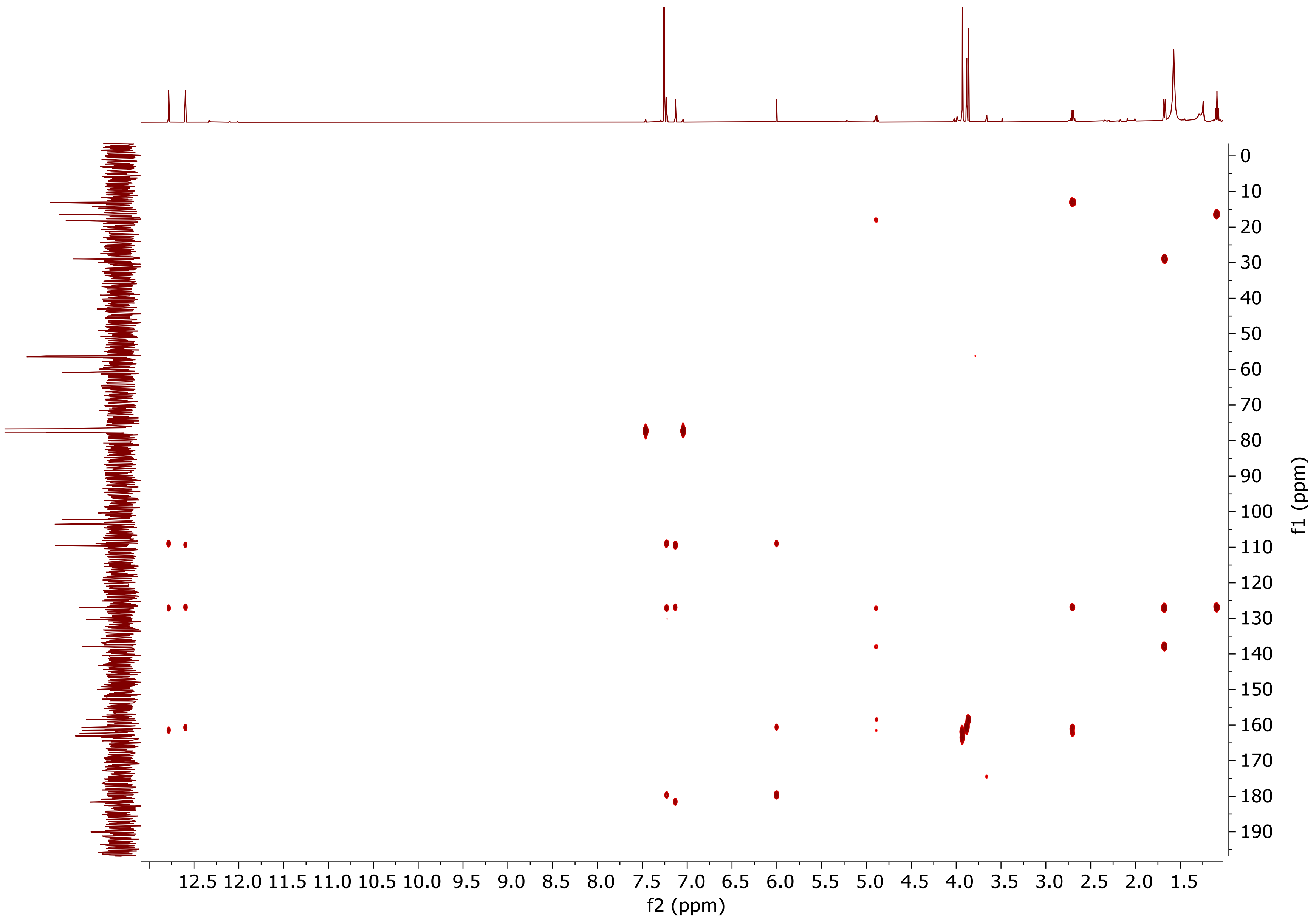

Figure S20: HMBC spectrum of compound **11** ($CDCl_3$, 500 MHz).

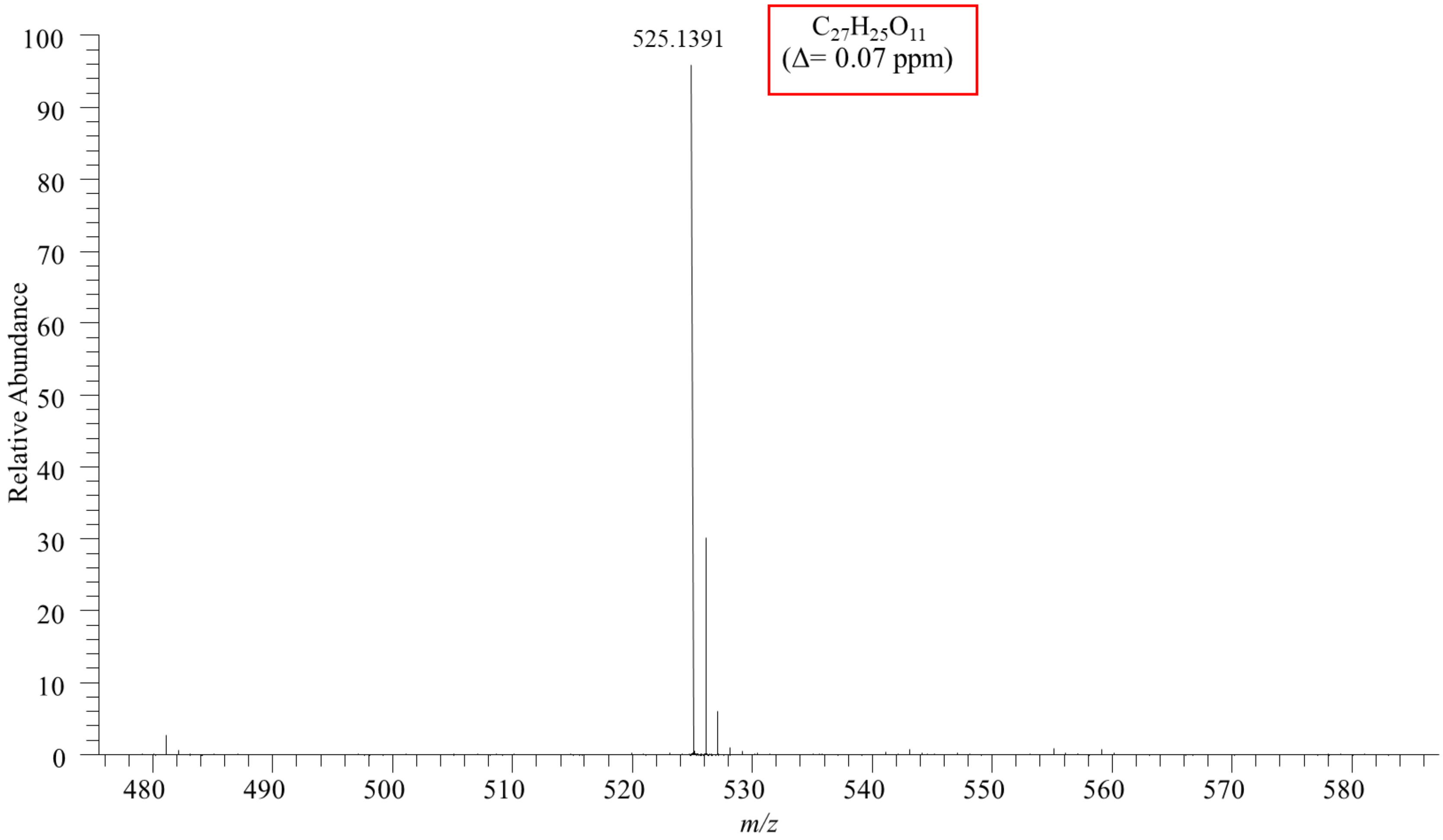

Figure S21: HRESIMS data for compound **12**.

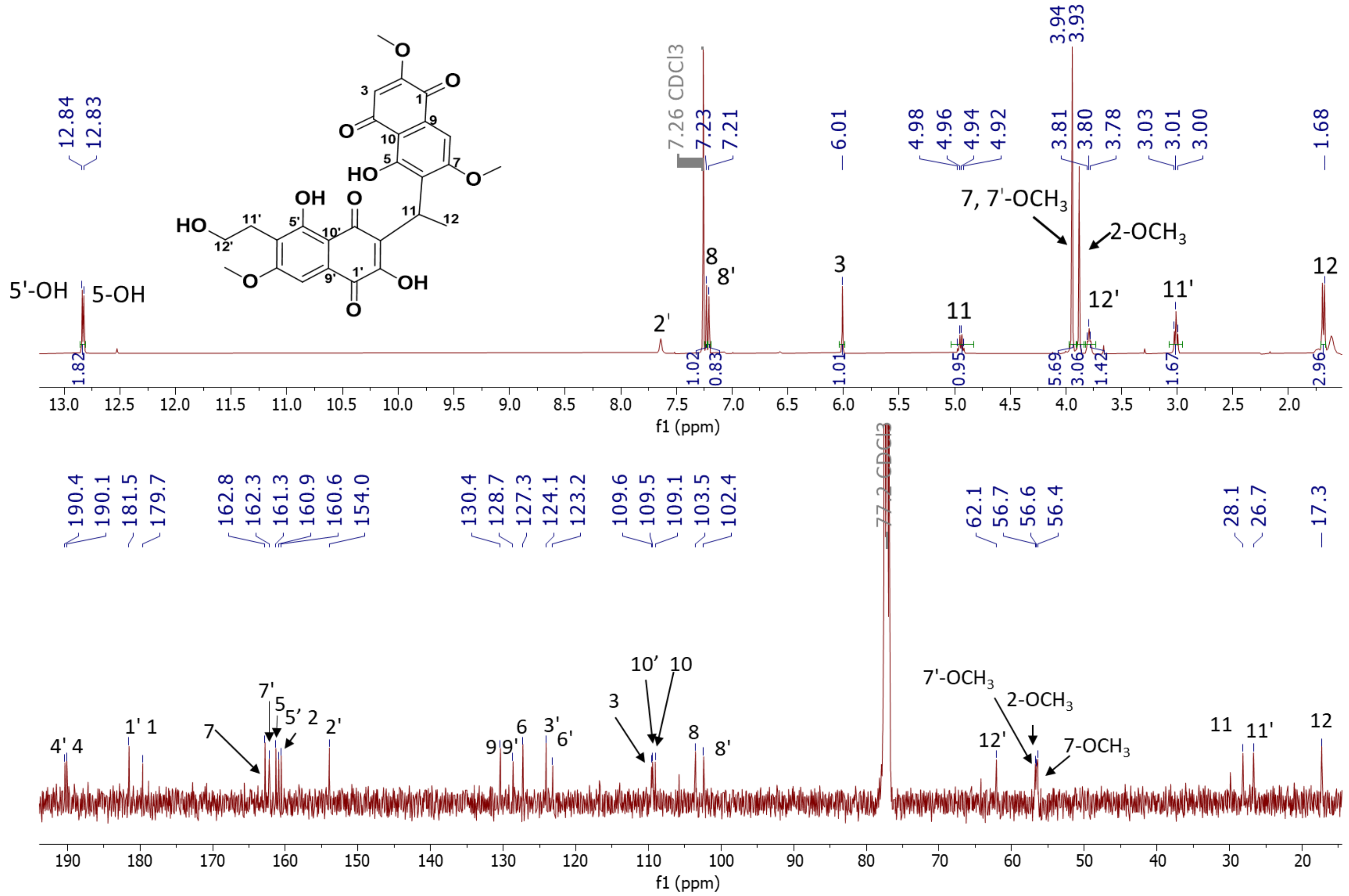

Figure S22: ^{1}H and ^{13}C NMR spectra for compound **12** ($CDCl_3$, 400 and 100 MHz, respectively).

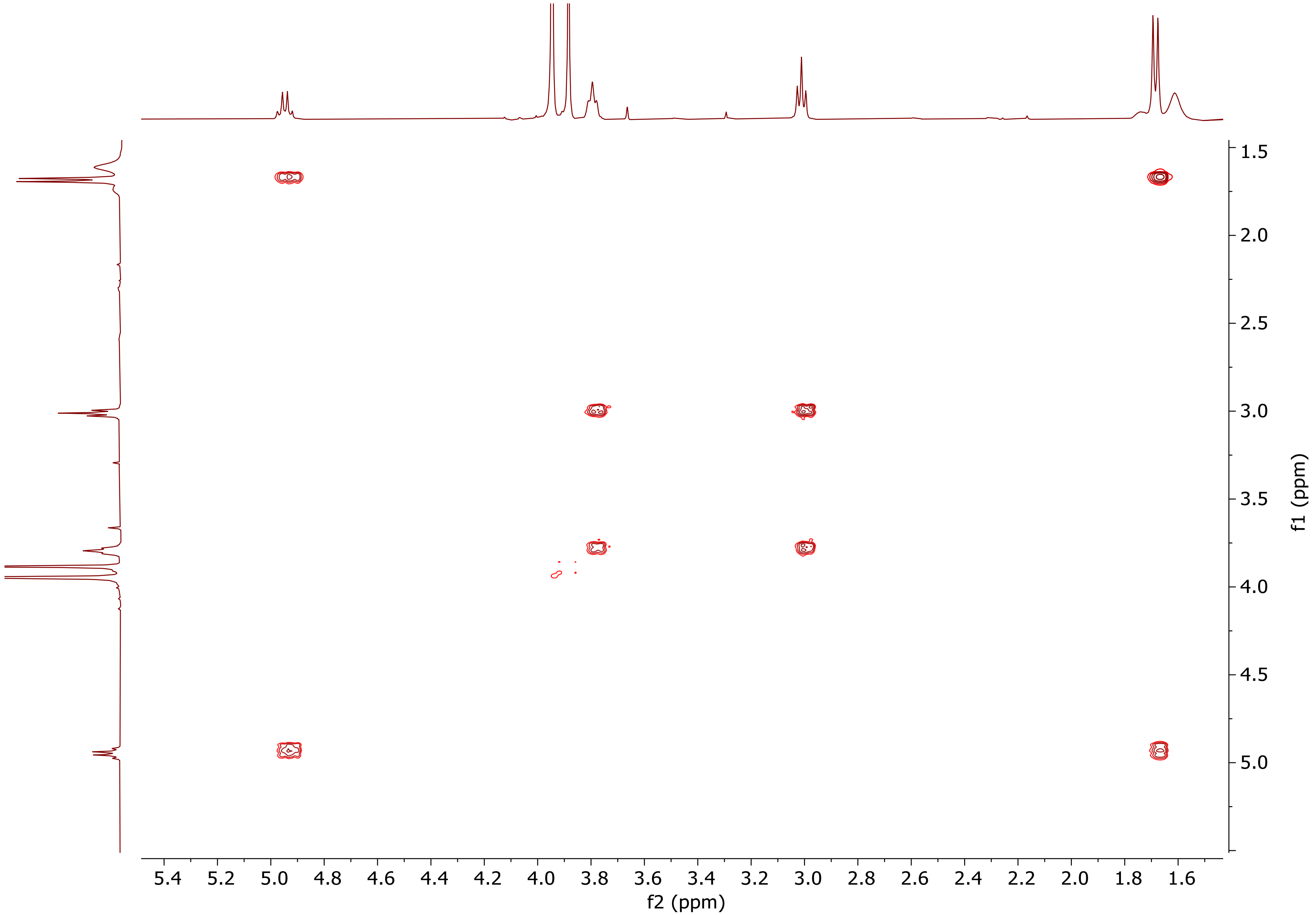

Figure S23: COSY spectrum for compound **12** ($CDCl_3$, 400 MHz).

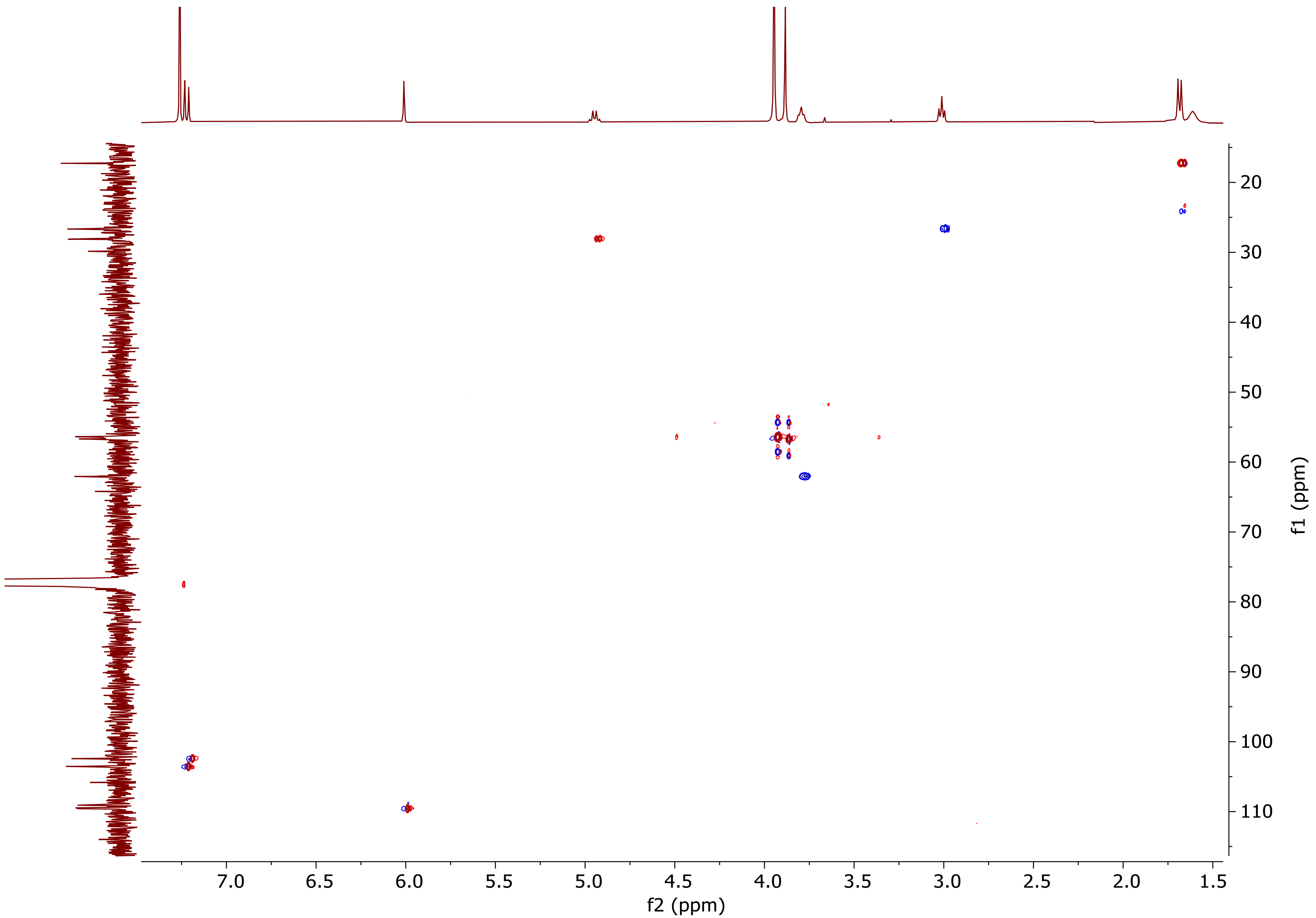

Figure S24: Edited HSQC spectrum of compound **12** ($CDCl_3$, 400 MHz).

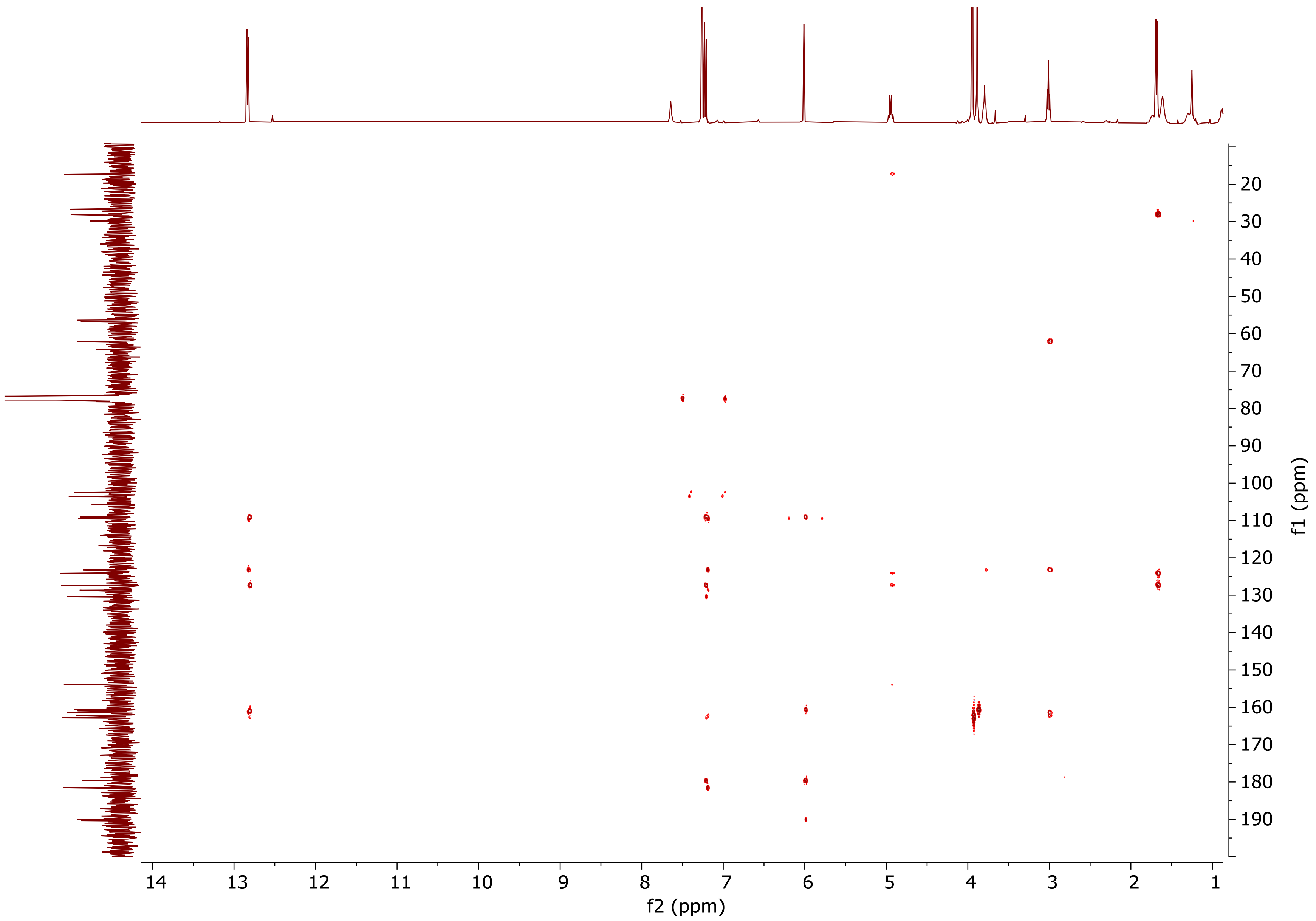

Figure S25: HMBC spectrum of compound **12** ($CDCl_3$, 400 MHz).

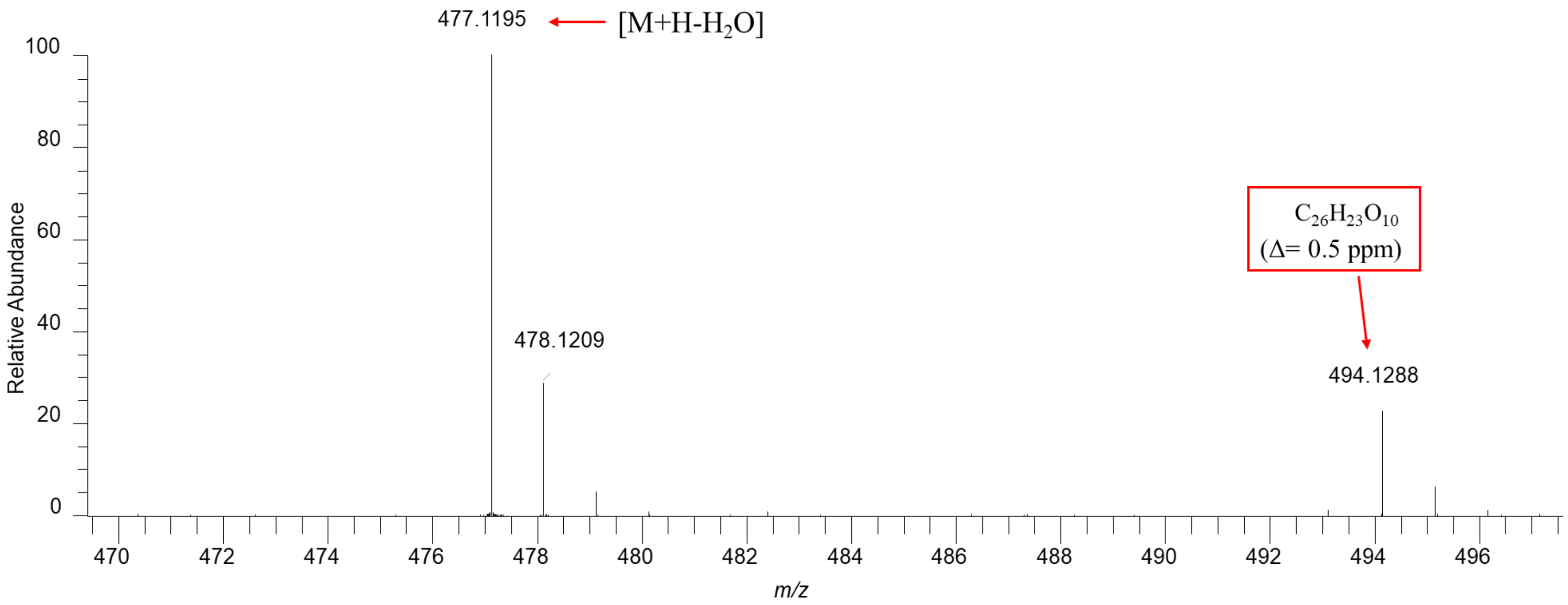

Figure S26: HRESIMS data for compound **13**.

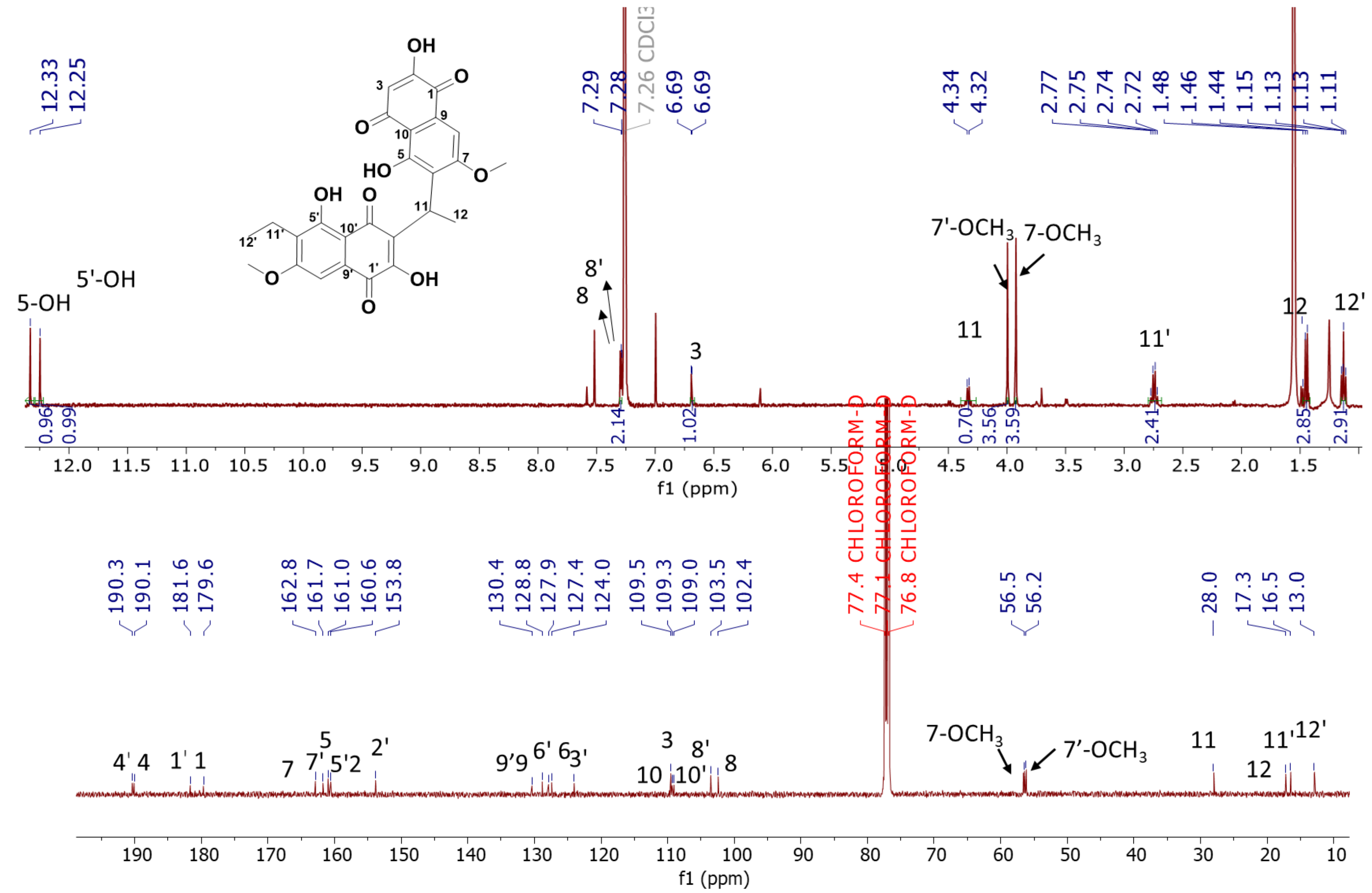

Figure S27: ^{1}H and ^{13}C NMR spectra for compound **13** ($CDCl_3$, 700 and 175 MHz, respectively).

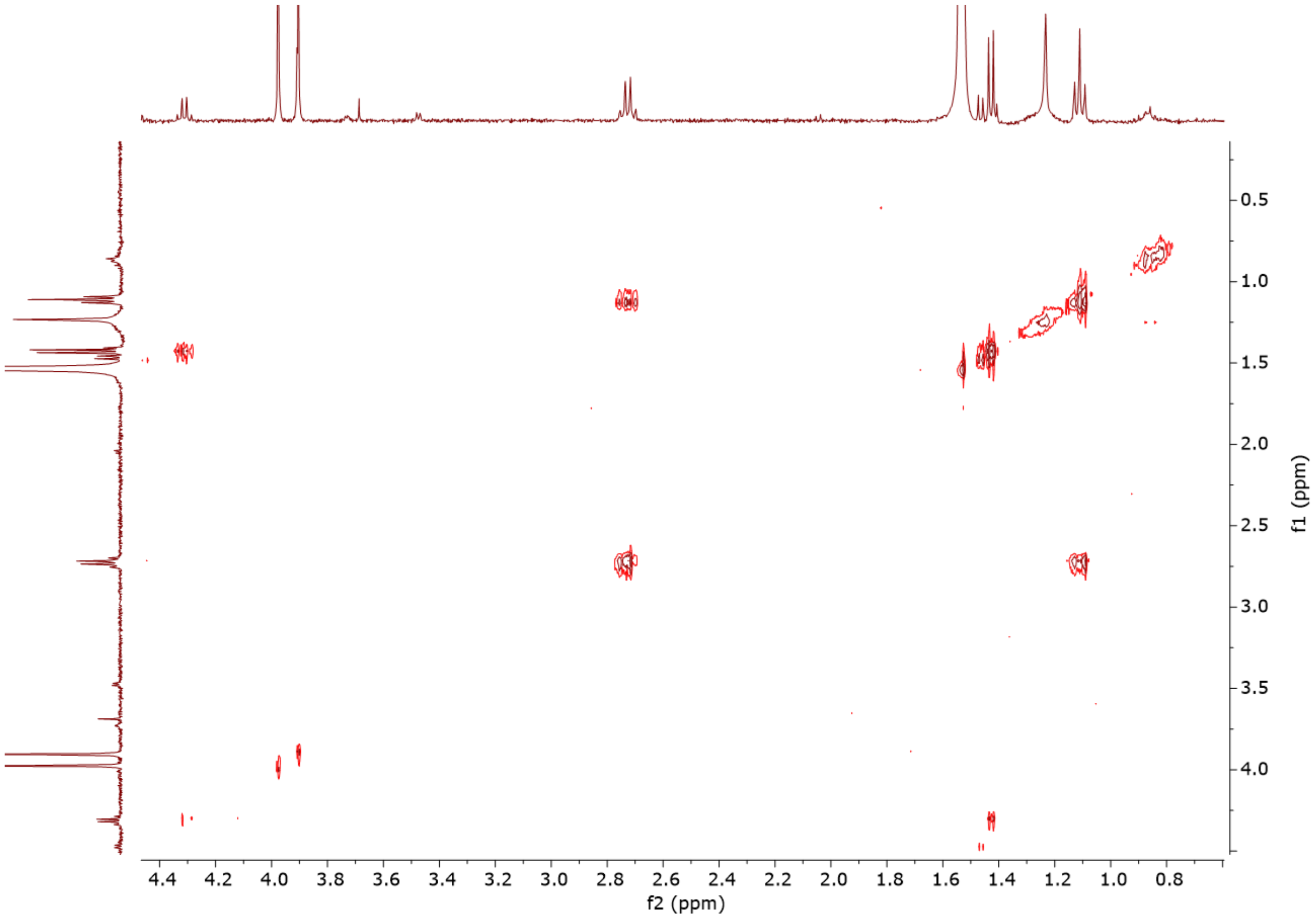

Figure S28: COSY spectrum for compound **13** ($CDCl_3$, 700 MHz).

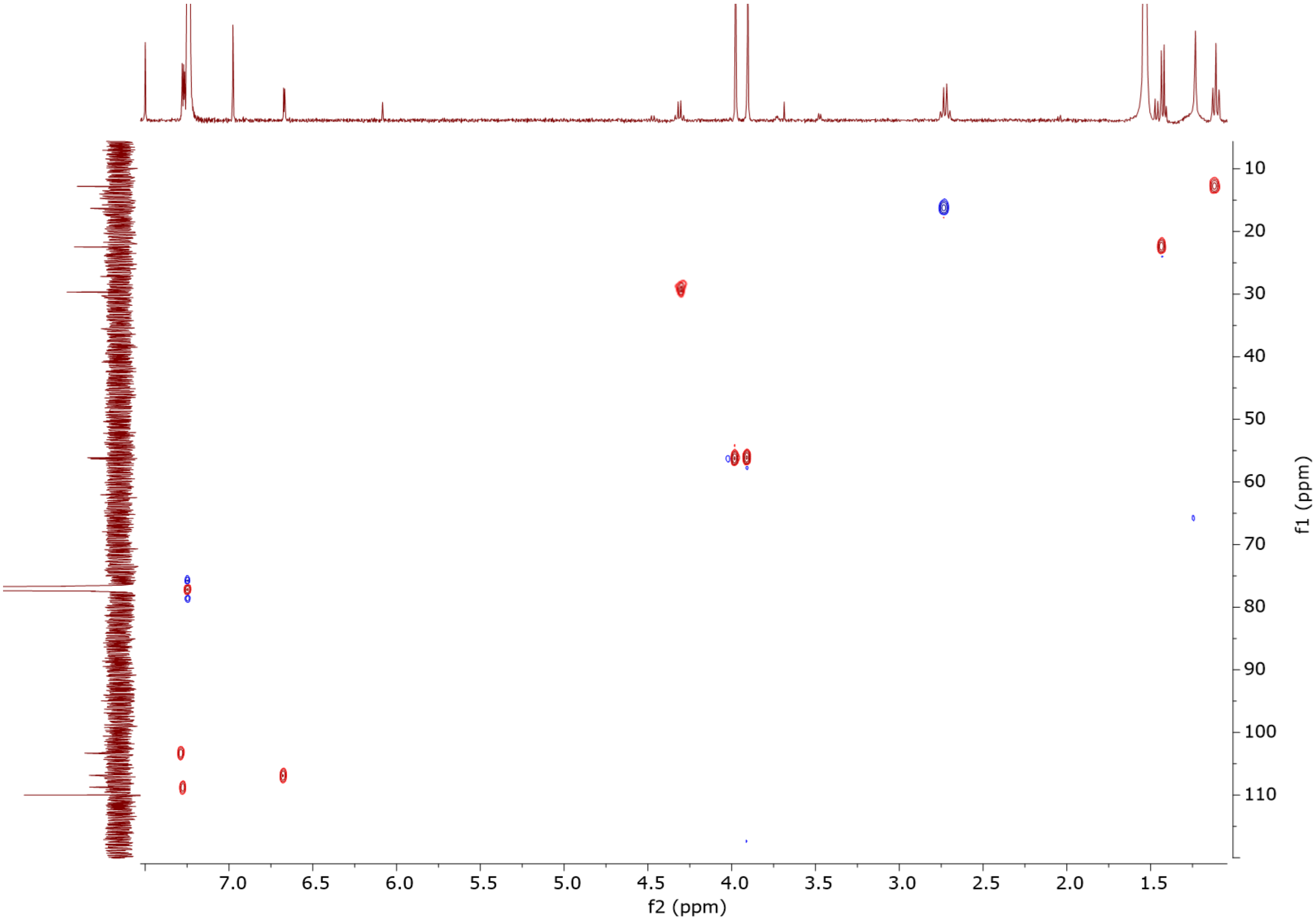

Figure S29: Edited HSQC spectrum of compound **13** ($CDCl_3$, 700 MHz).

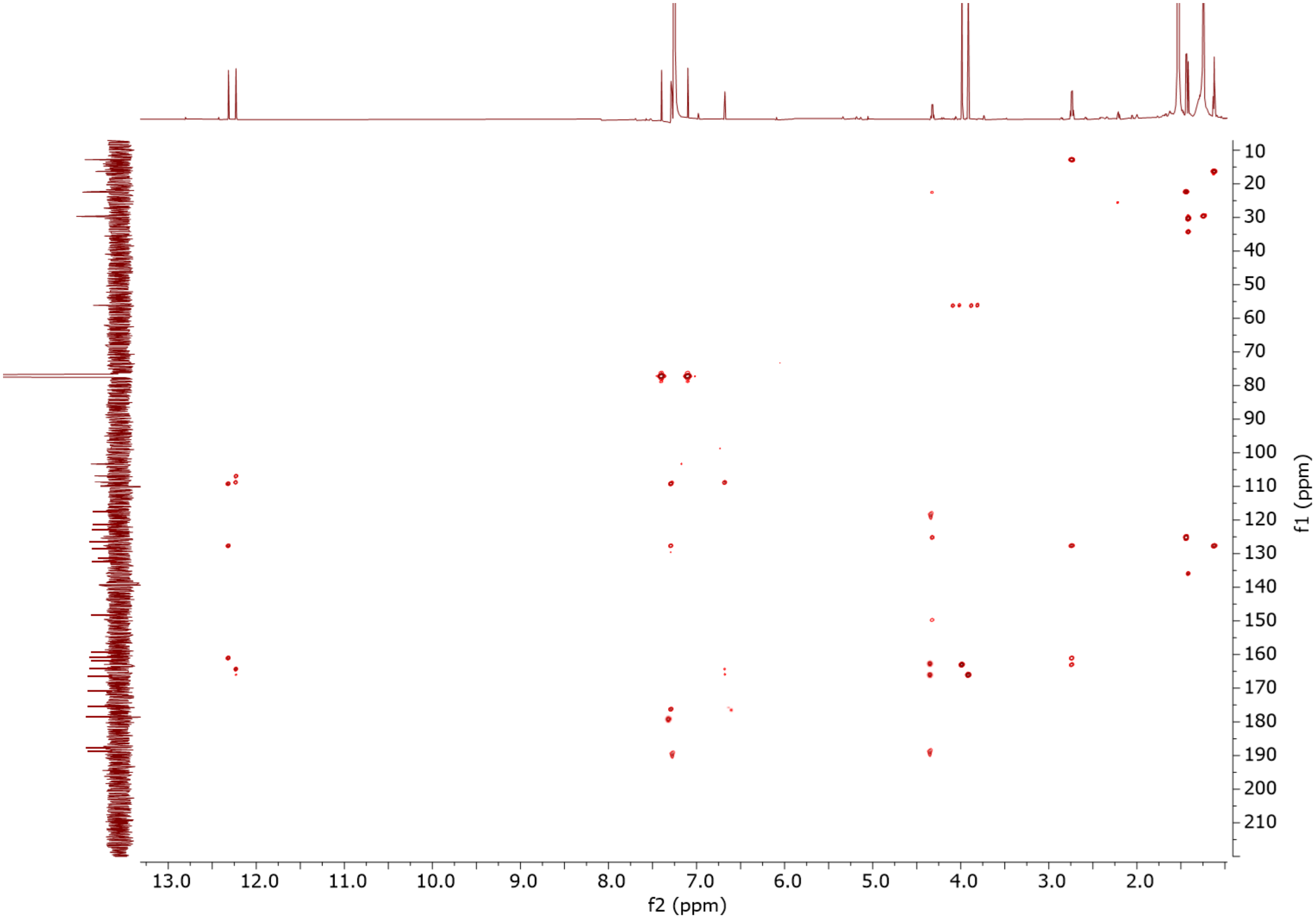

Figure S30: HMBC spectrum of compound **13** ($CDCl_3$, 700 MHz).

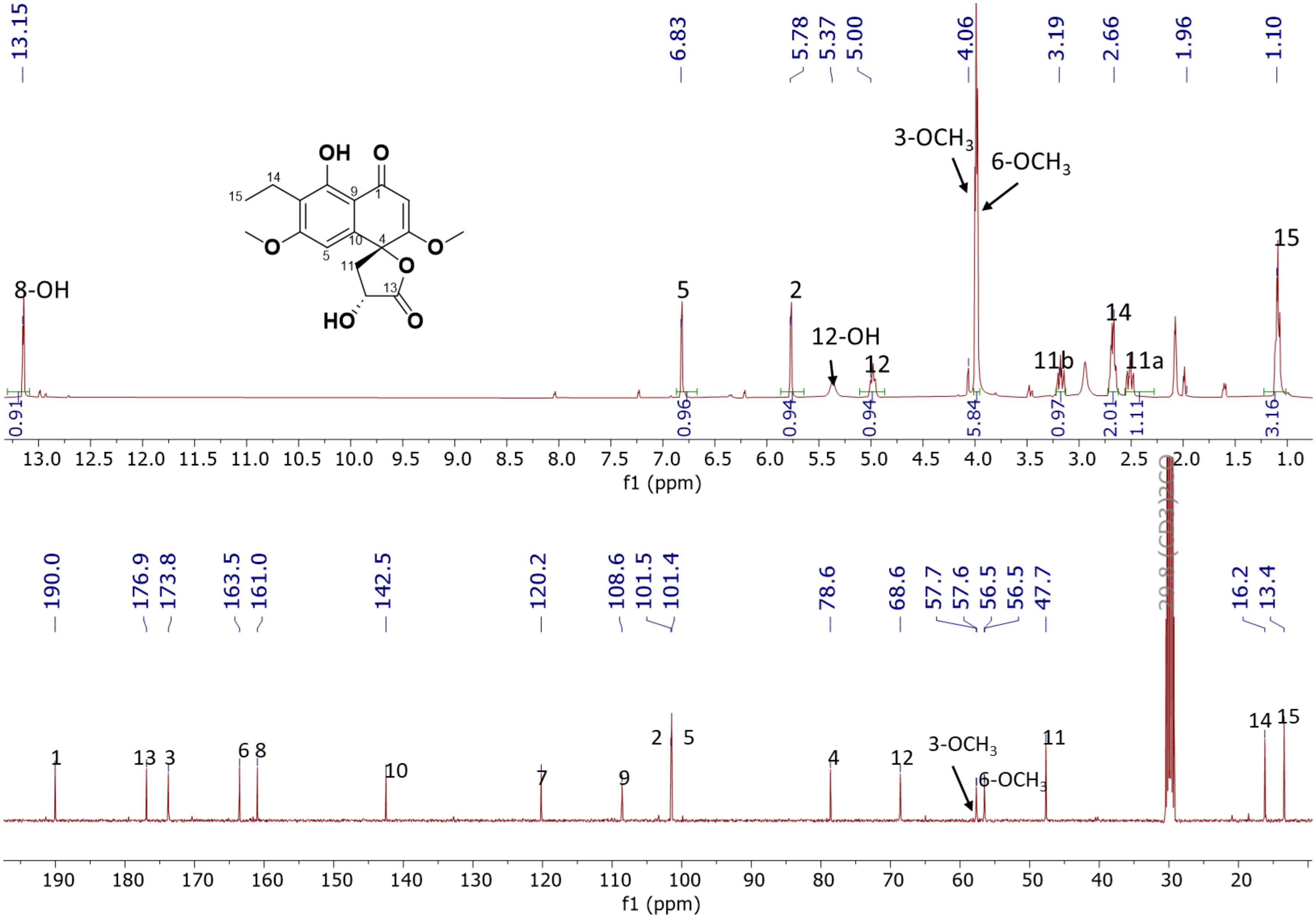

Figure S31: ^{1}H and ^{13}C NMR spectra for perenniporide A (**14**) (Acetone-d_6, 400 and 100 MHz, respectively).

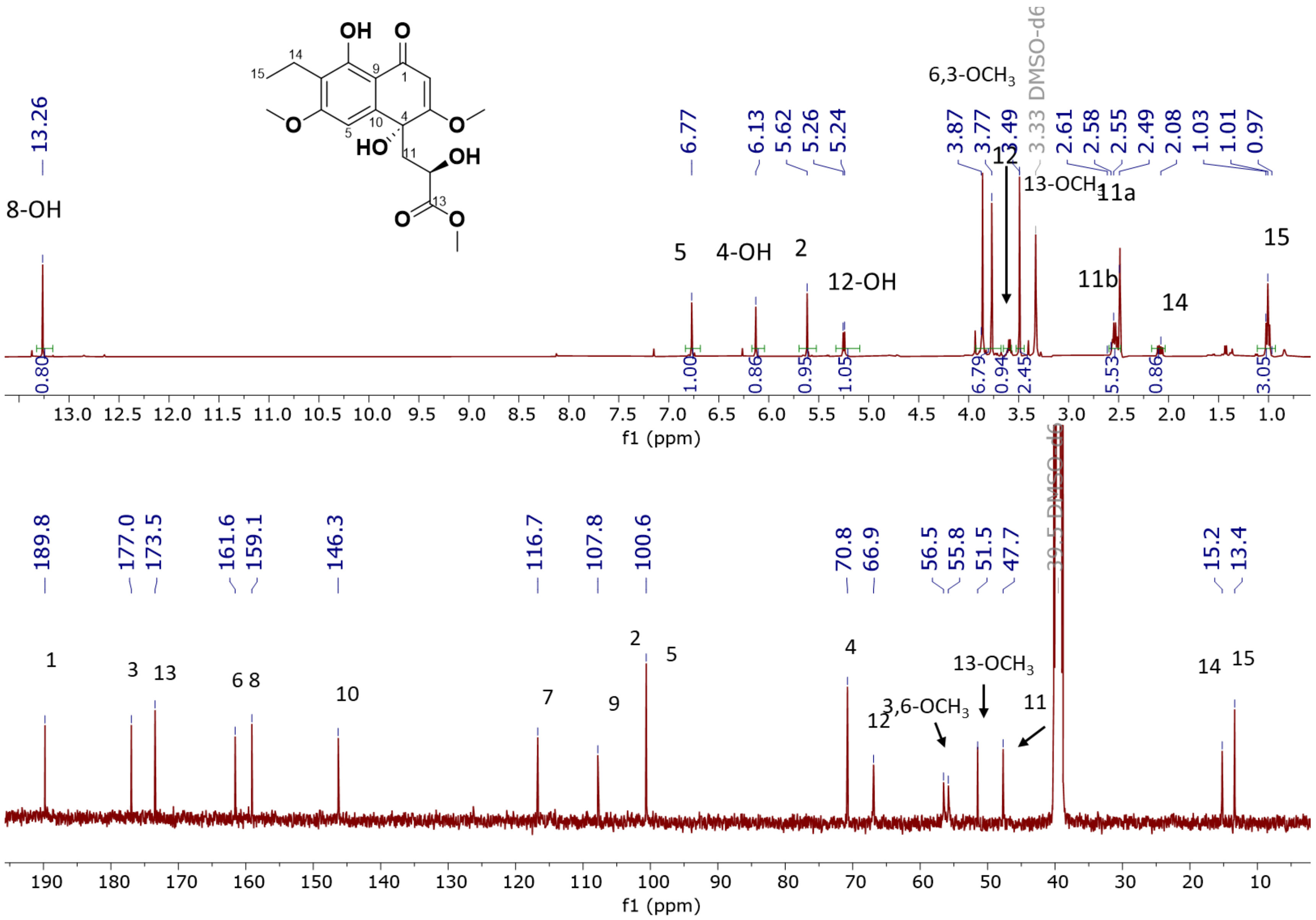

Figure S32: ^{1}H and ^{13}C NMR spectra for perenniporide B (**15**) (DMSO-d_6, 400 and 100 MHz, respectively).

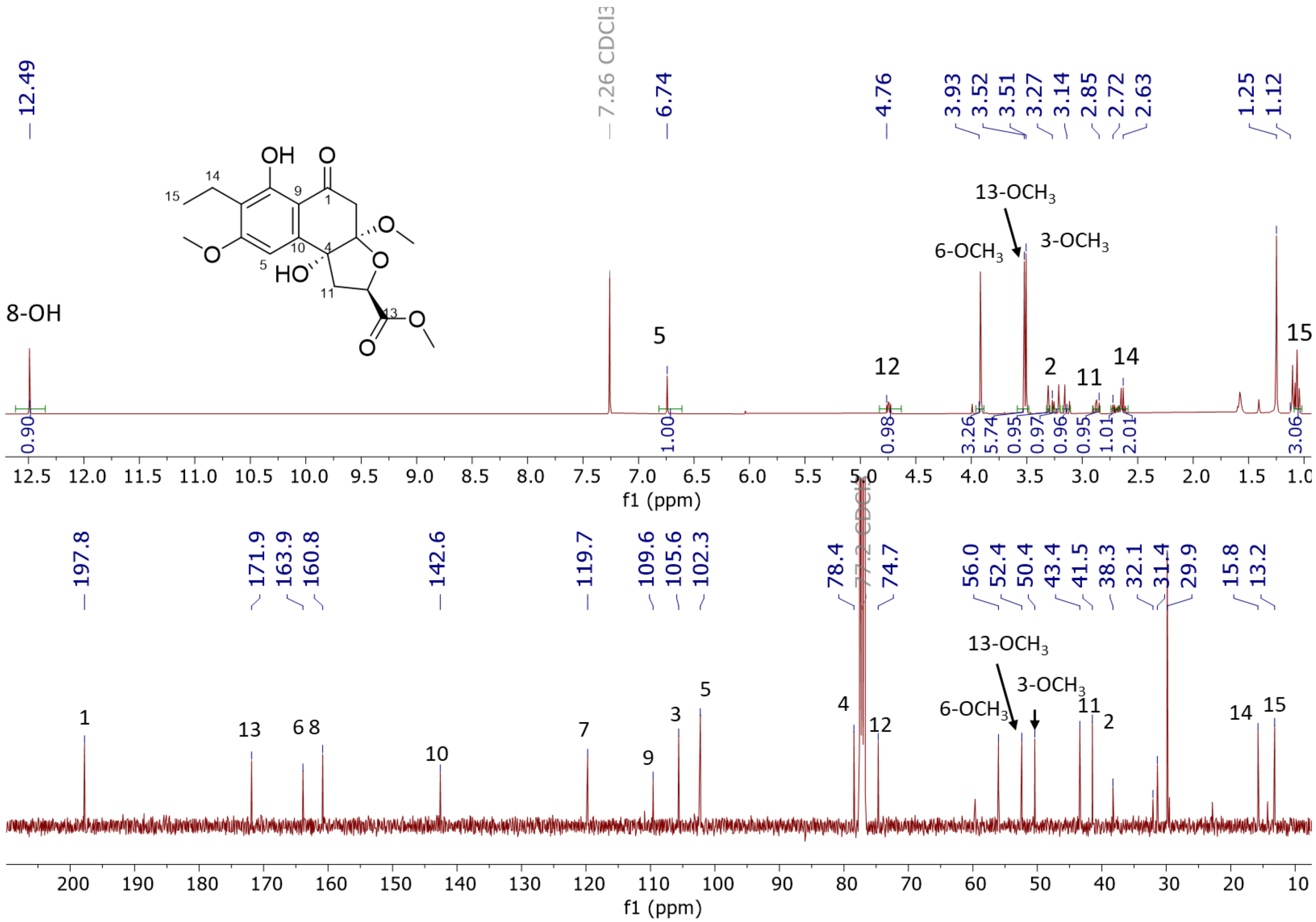

Figure S33: ^{1}H and ^{13}C NMR spectra for perenniporide C (**16**) ($CDCl_3$, 400 and 100 MHz, respectively).

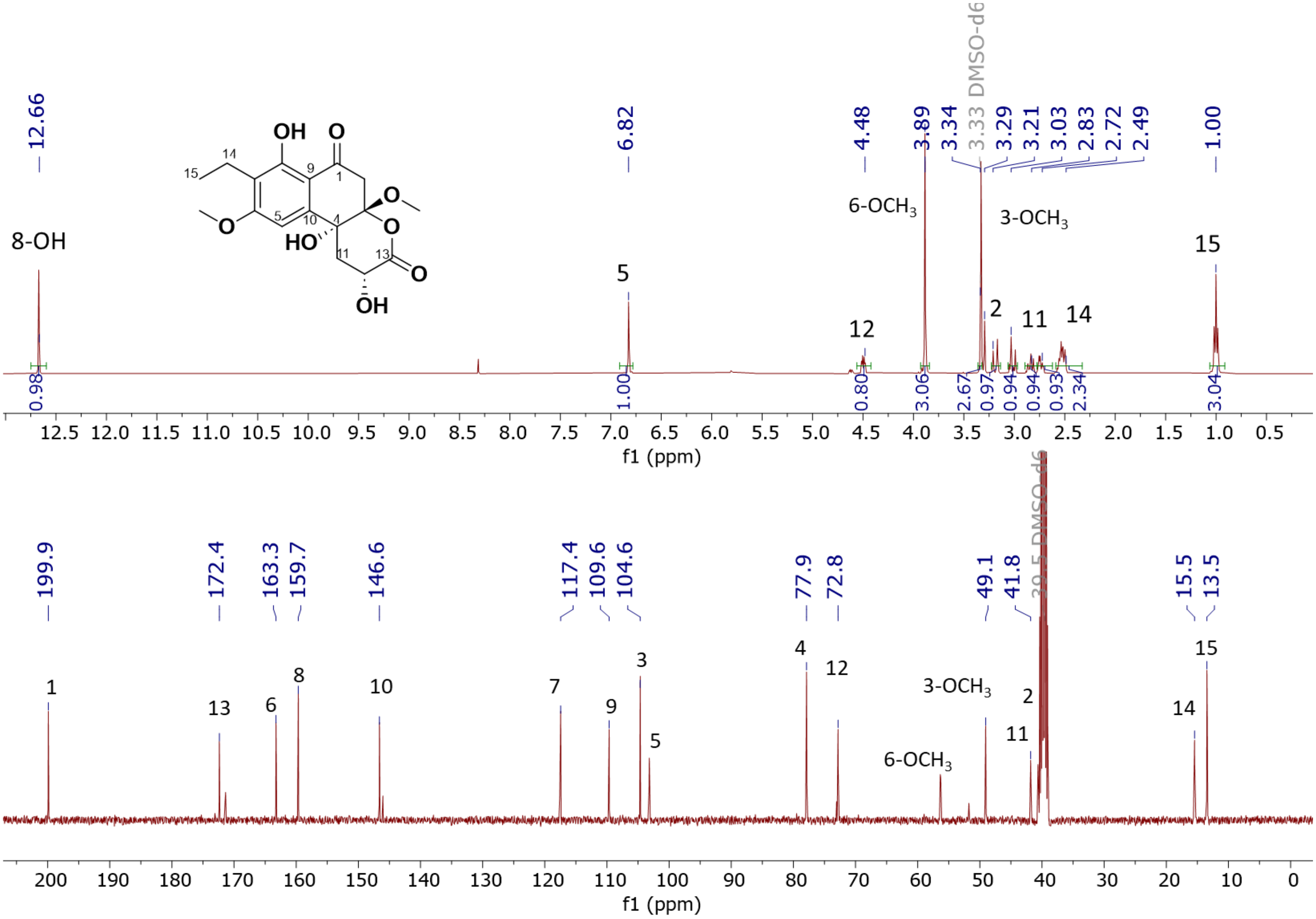

Figure S34: ^{1}H and ^{13}C NMR spectra for perenniporide D (**17**) (DMSO-d_6, 400 and 100 MHz, respectively).

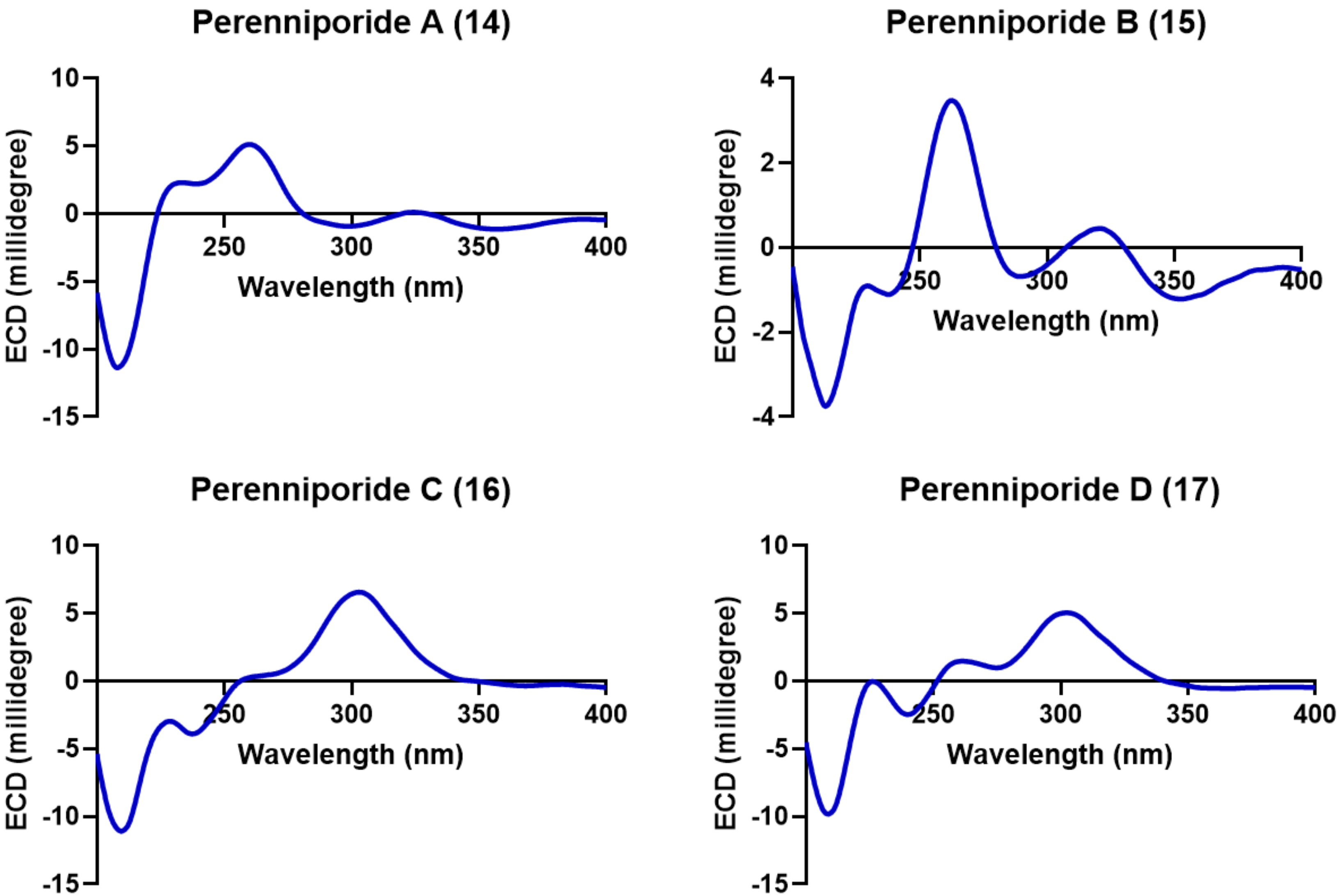

Figure S35: ECD spectra for compounds **14-17** in CH_3OH at a concentration of 0.01, 0.02, 0.02, and 0.01 mg/mL, respectively.

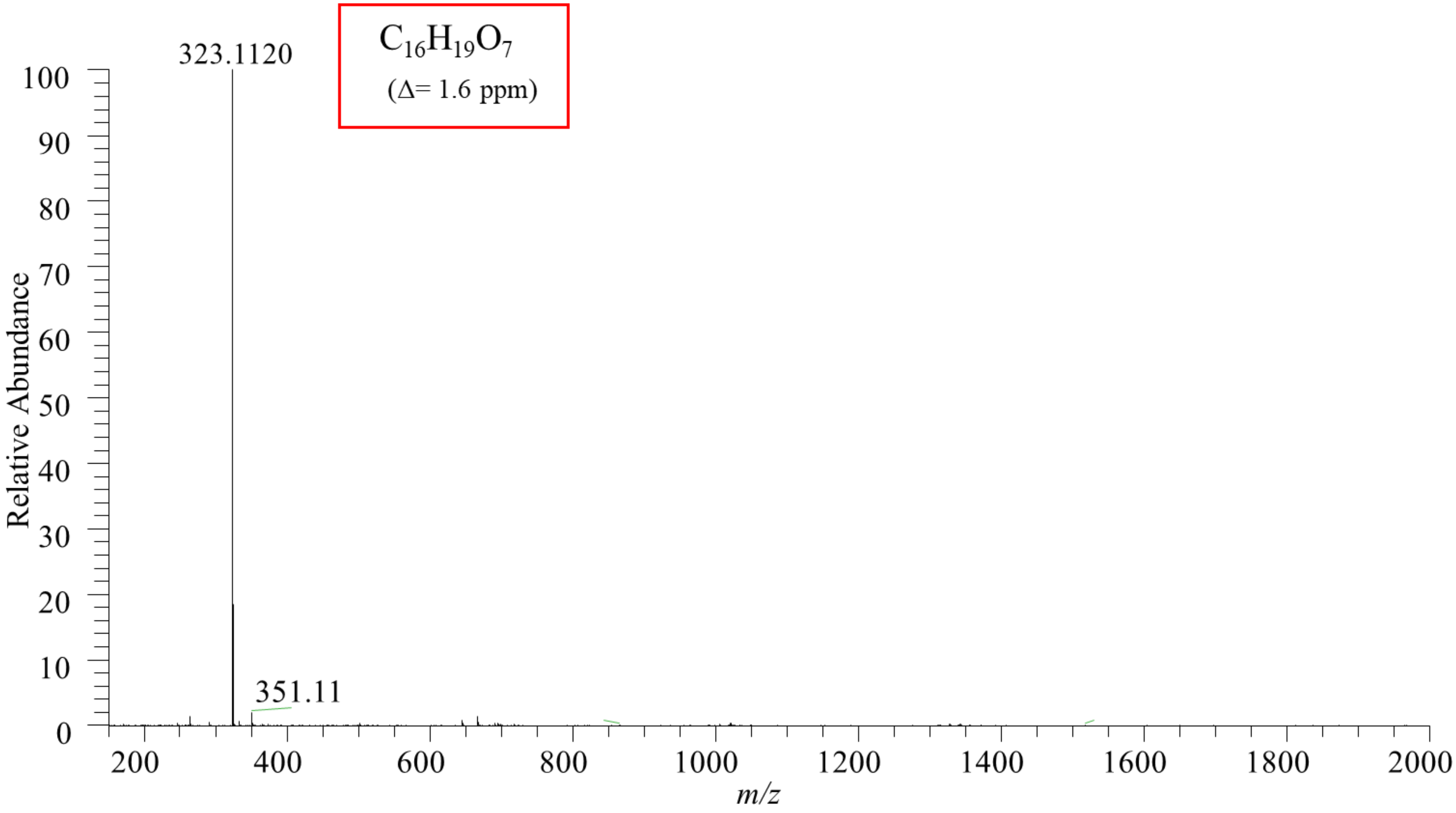

Figure S36: HRESIMS data for compound **18**.

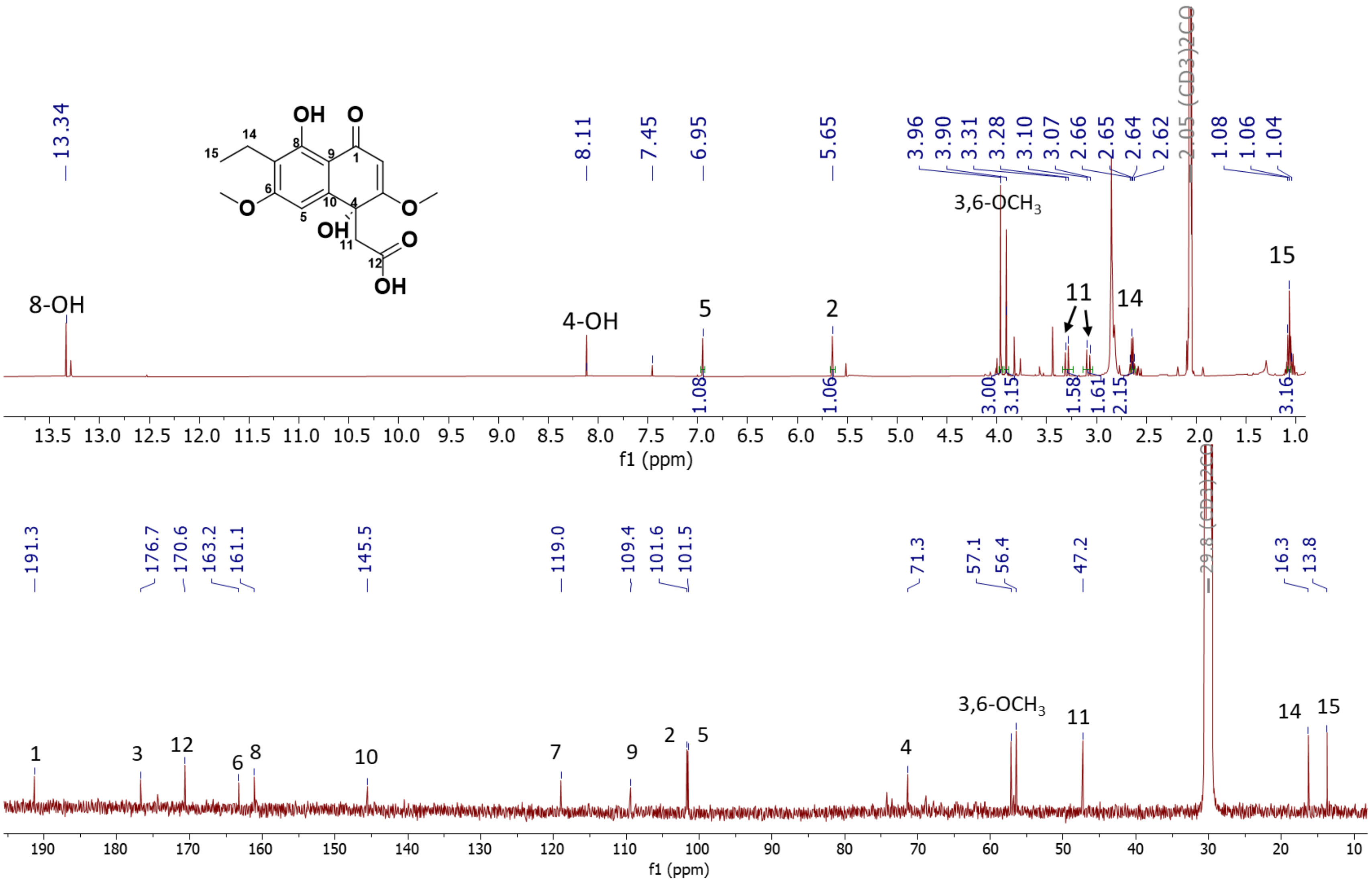

Figure S37: ^{1}H and ^{13}C NMR spectra for perenniporide E (**18**) (Acetone-*d*$_6$, 500 and 125 MHz, respectively).

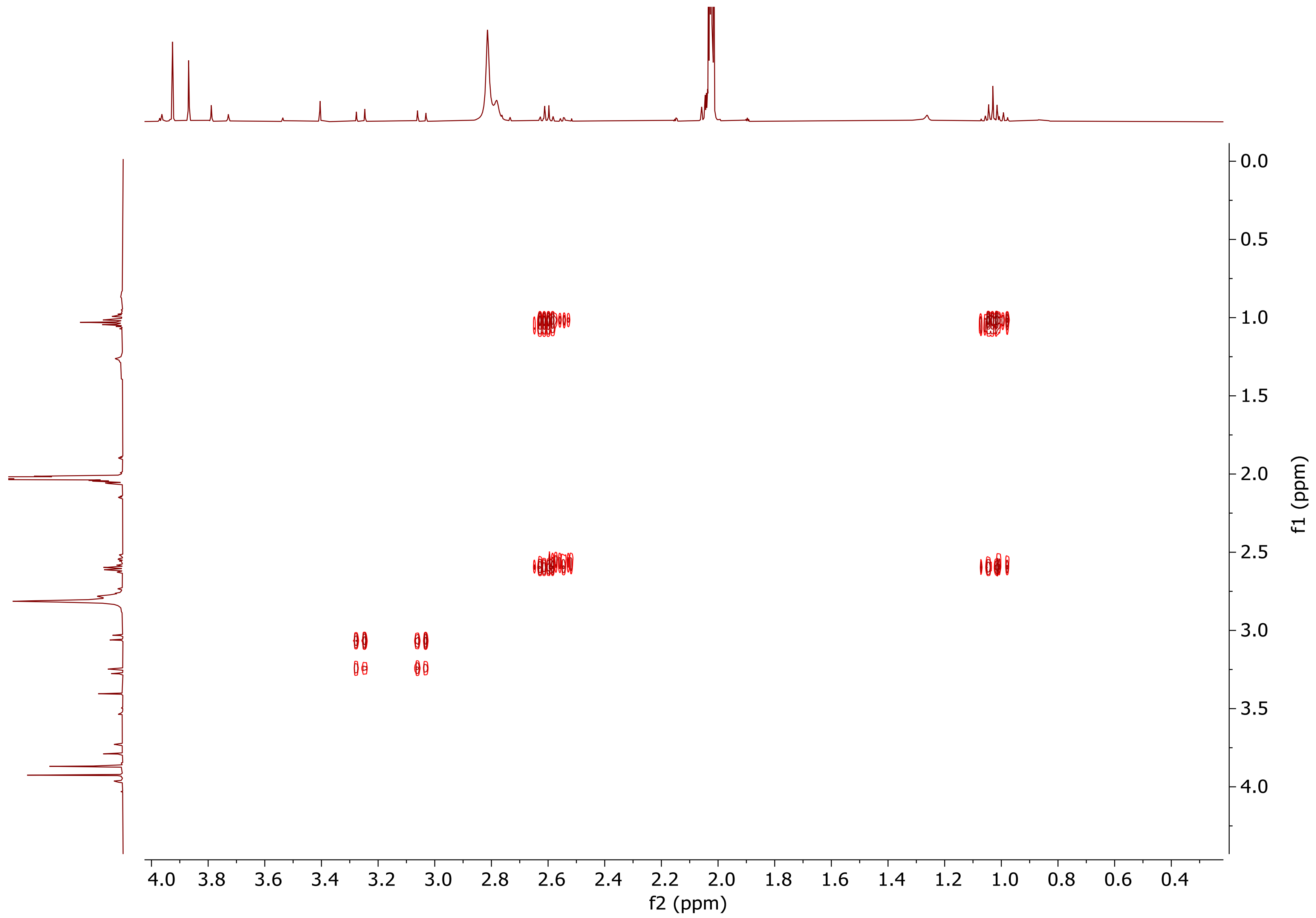

Figure S38: COSY spectrum for perenniporide E (**18)** (Acetone-d_6, 500 MHz).

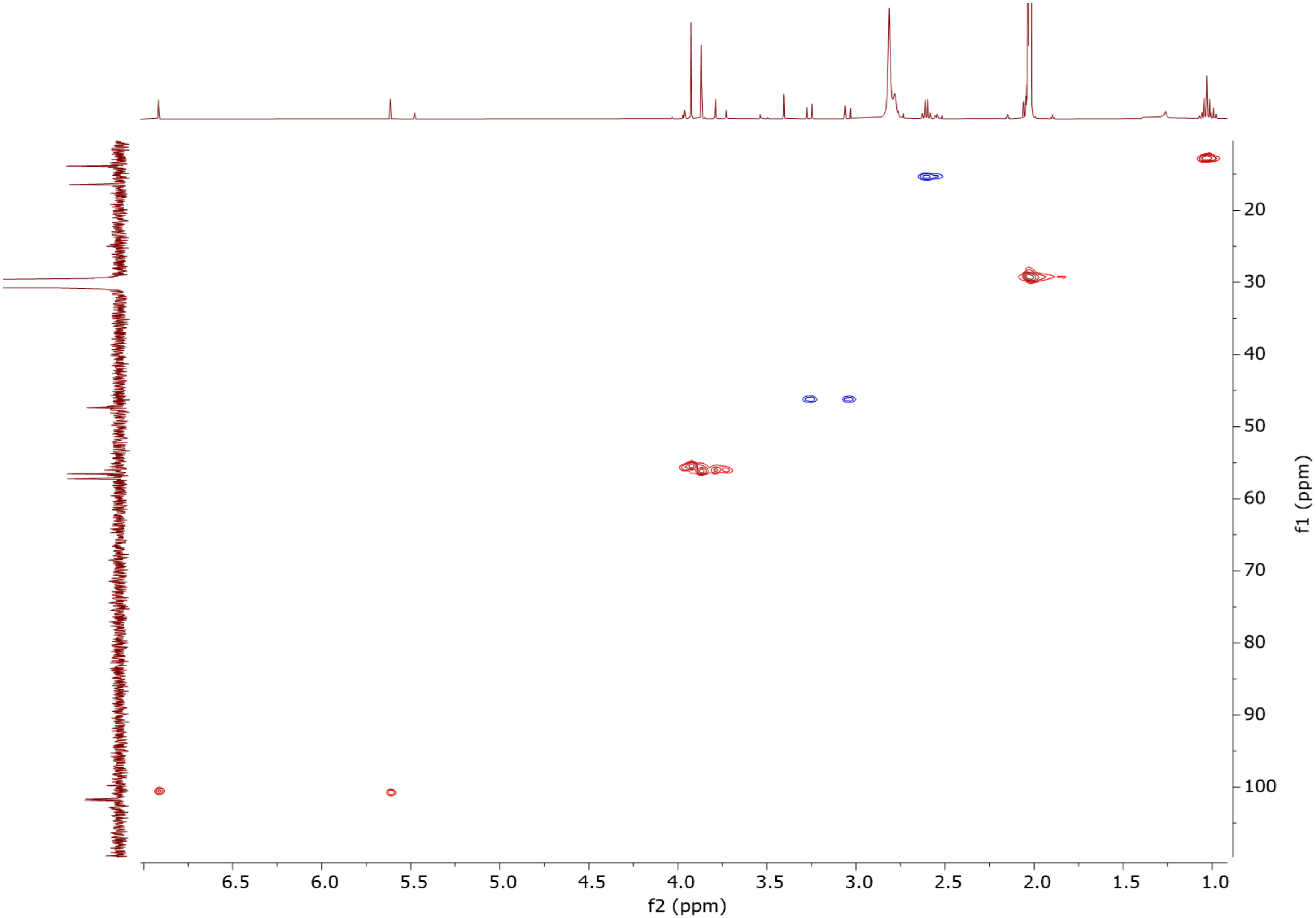

Figure S39: Edited HSQC spectrum for perenniporide E (**18)** (Acetone-d_6, 500 MHz).

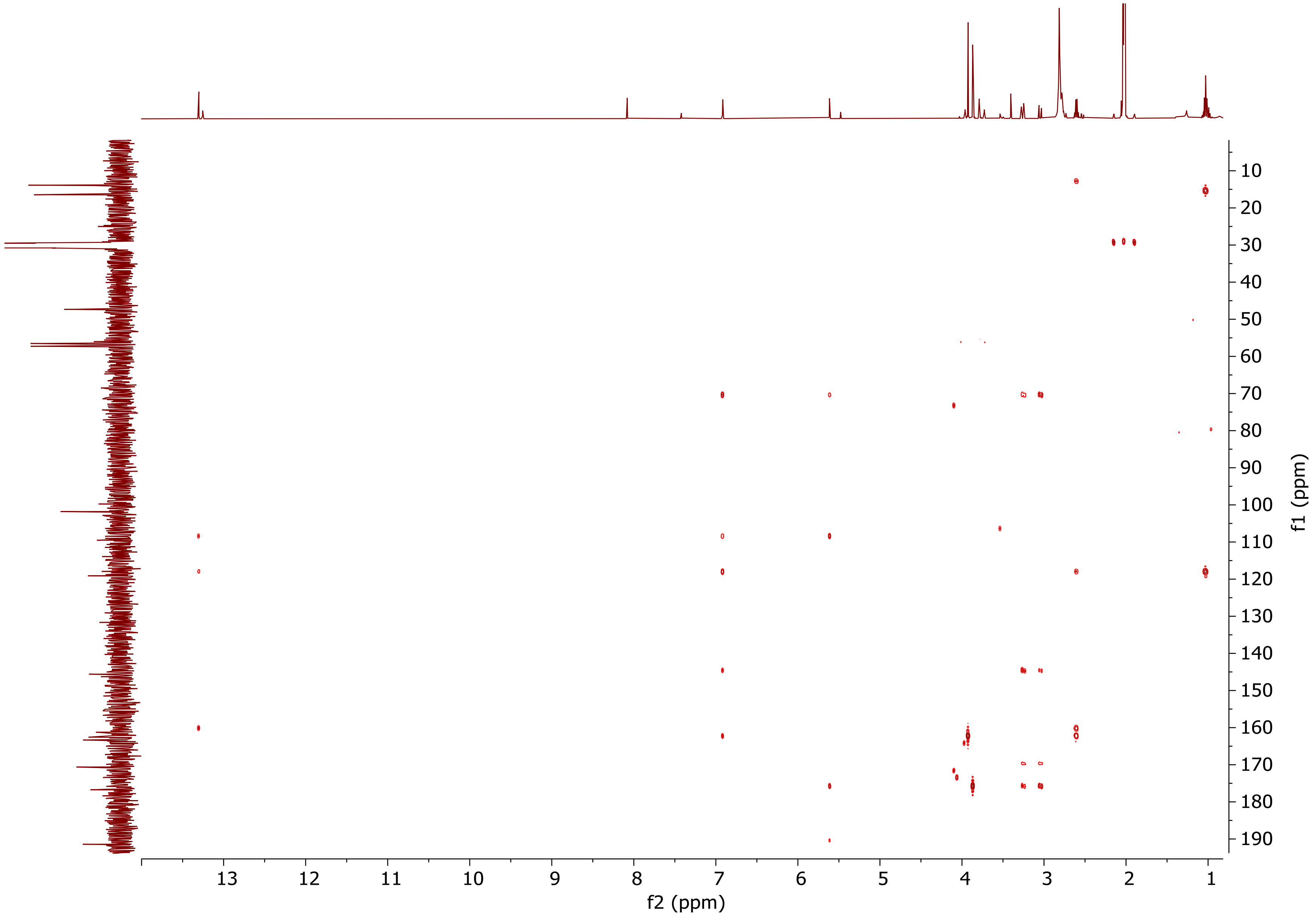

Figure S40: HMBC spectrum of perenniporide E (**18**) (Acetone-d_6, 500 MHz).

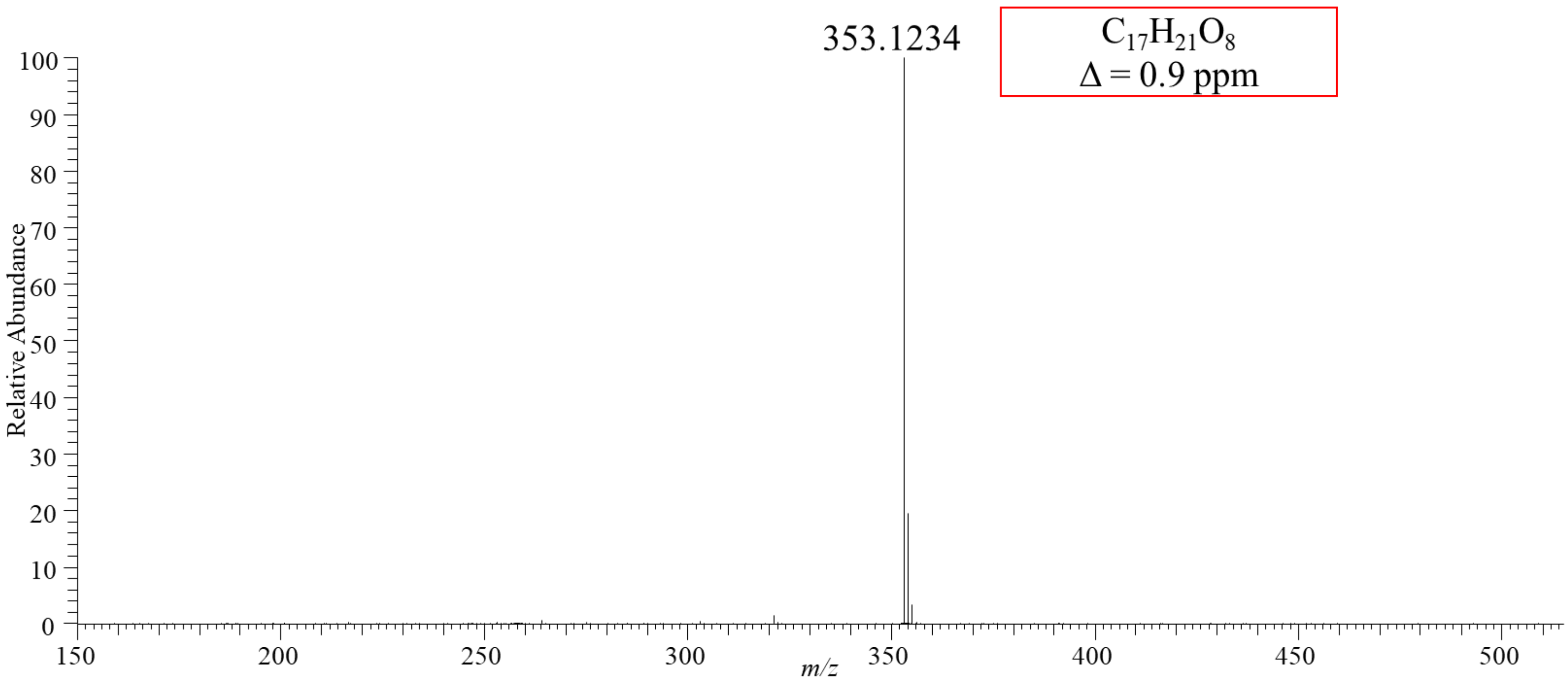

Figure S41: HRESIMS data for compound **19**.

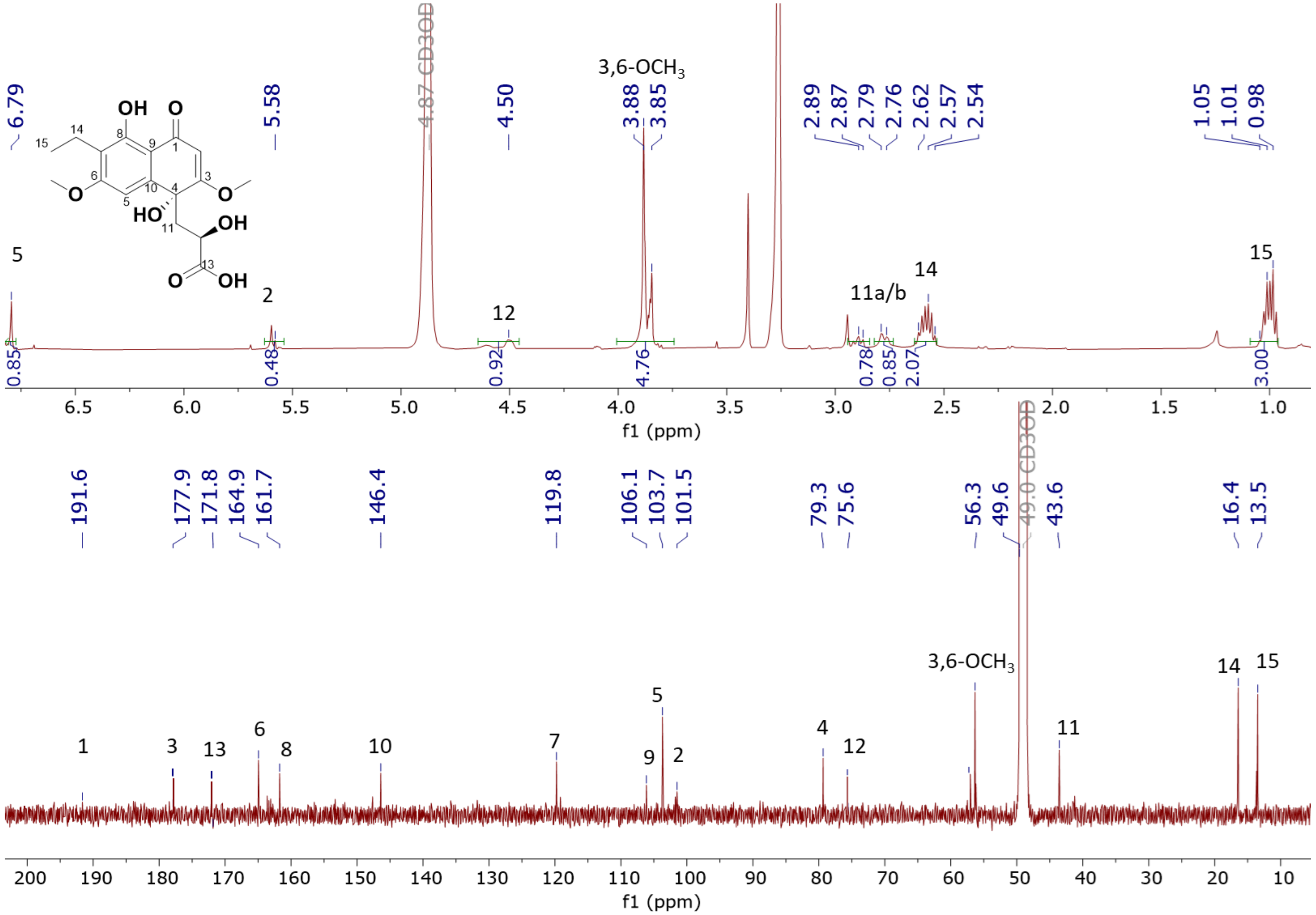

Figure S42: ^{1}H and ^{13}C NMR spectra for perenniporide F (**19)** (CD3OD, 500 and 125 MHz, respectively).

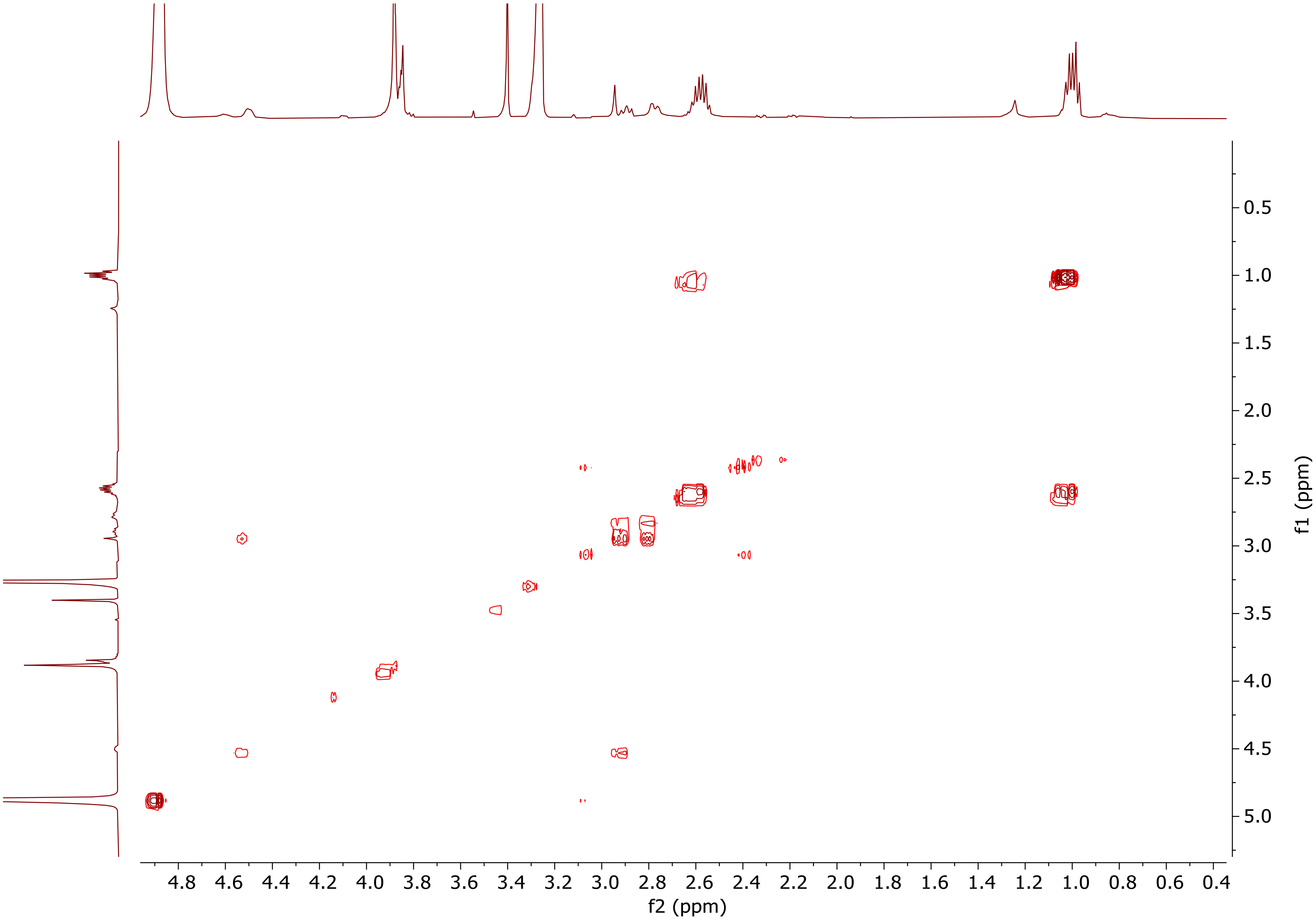

Figure S43:COSY spectrum for perenniporide F (**19**) (CD_3OD, 500 MHz).

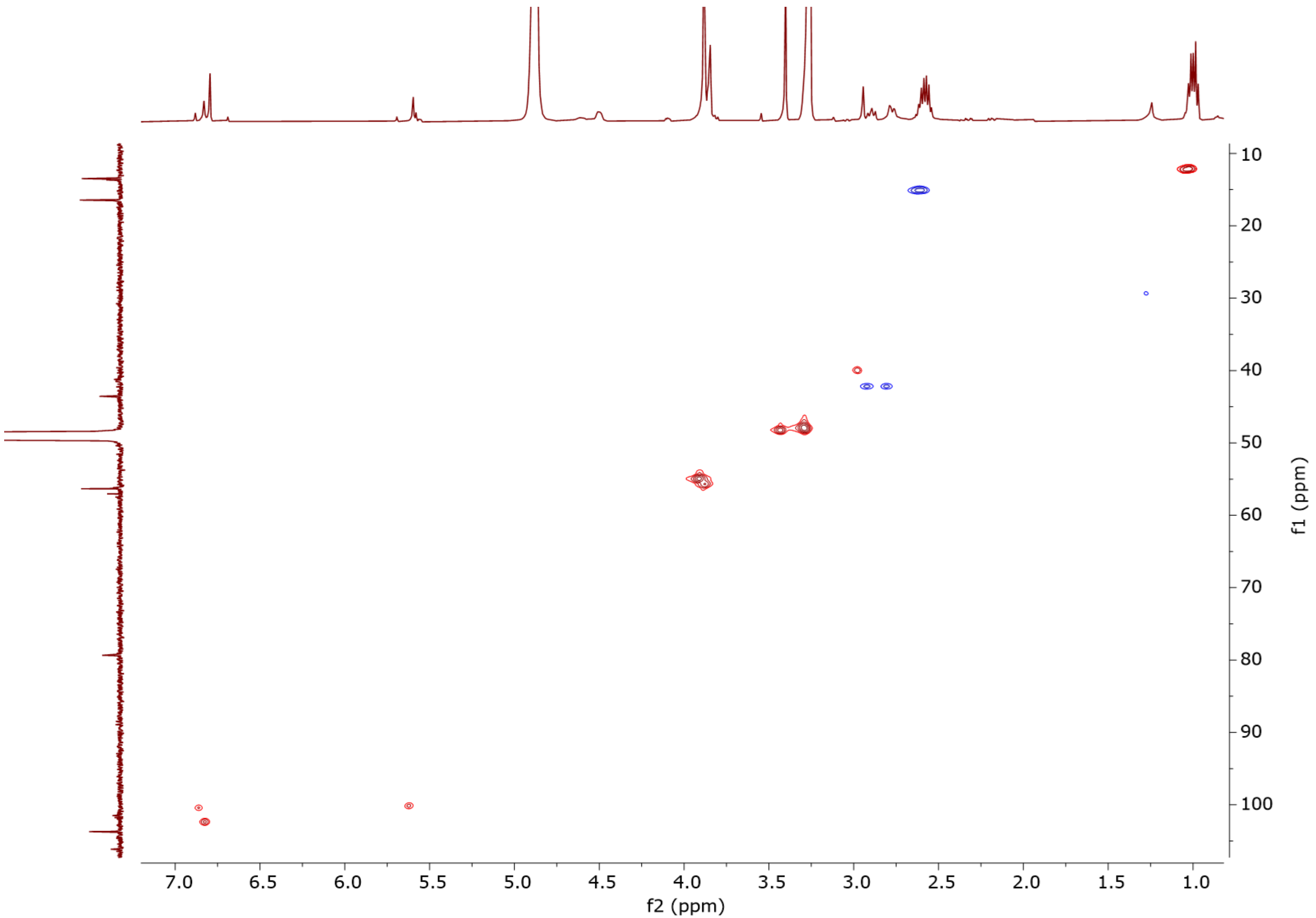

Figure S44: Edited HSQC spectrum for perenniporide F (**19**) (CD_3OD, 500 MHz).

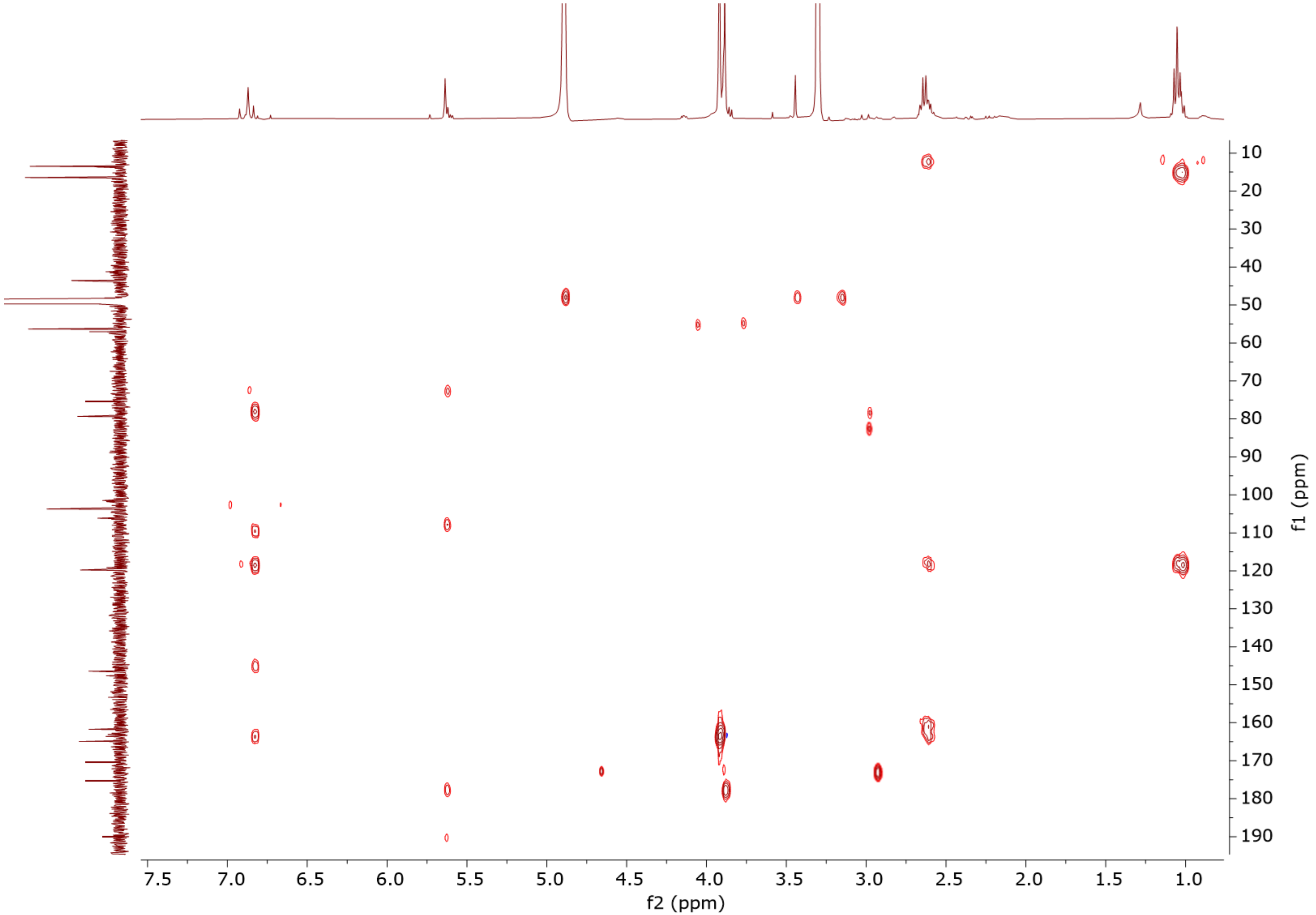

Figure S45: HMBC spectrum of perenniporide F (**19**) (CD_3OD, 500 MHz).

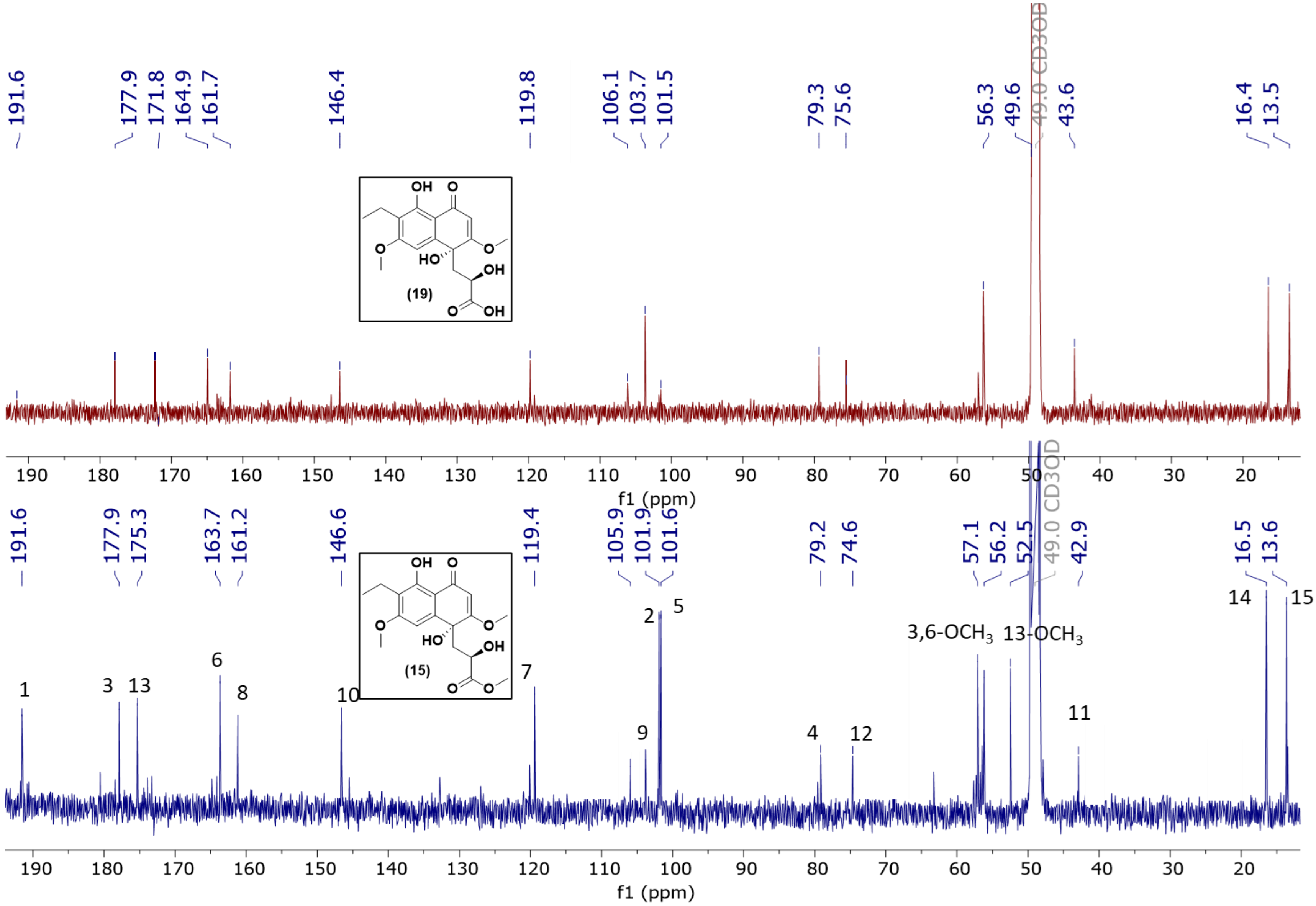

Figure S46: Stacked ^{13}C spectra NMR of **19** (top) and **15** (bottom), (CD_3OD, 125 MHz).

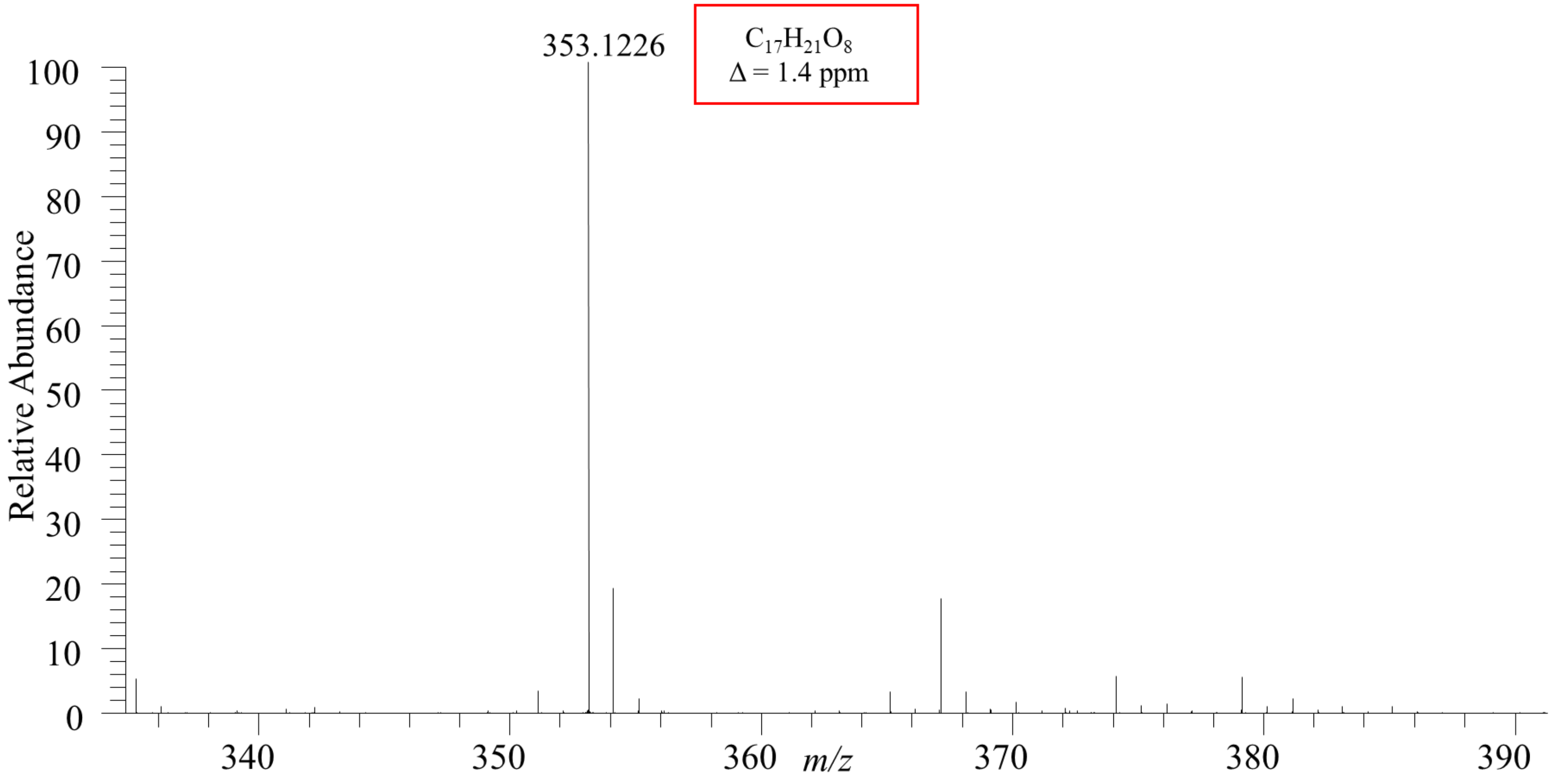

Figure S47: HRESIMS data for compound **20**.

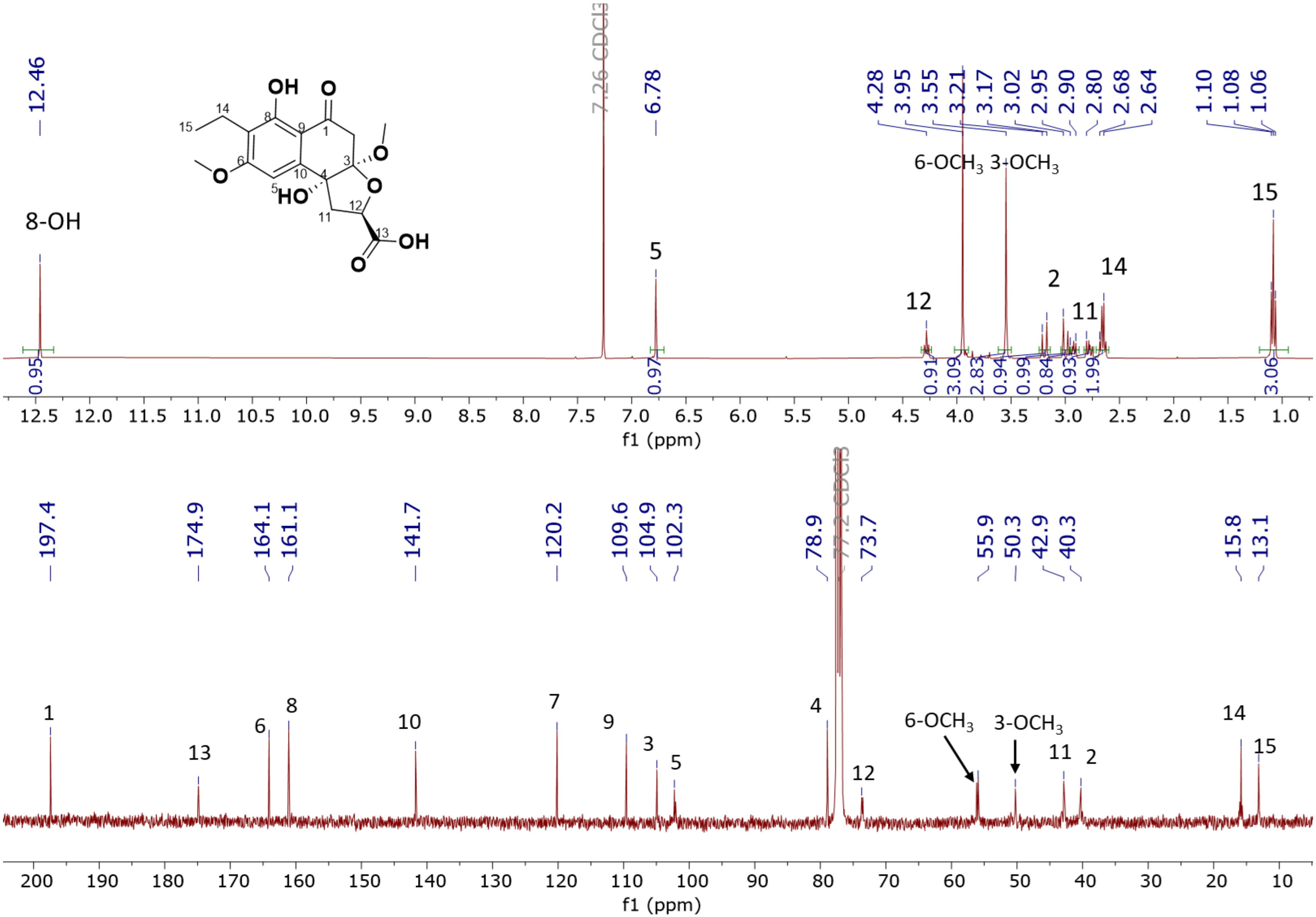

Figure S48: ^{1}H and ^{13}C NMR spectra for perenniporide G (**20**) ($CDCl_3$, 500 and 125 MHz, respectively).

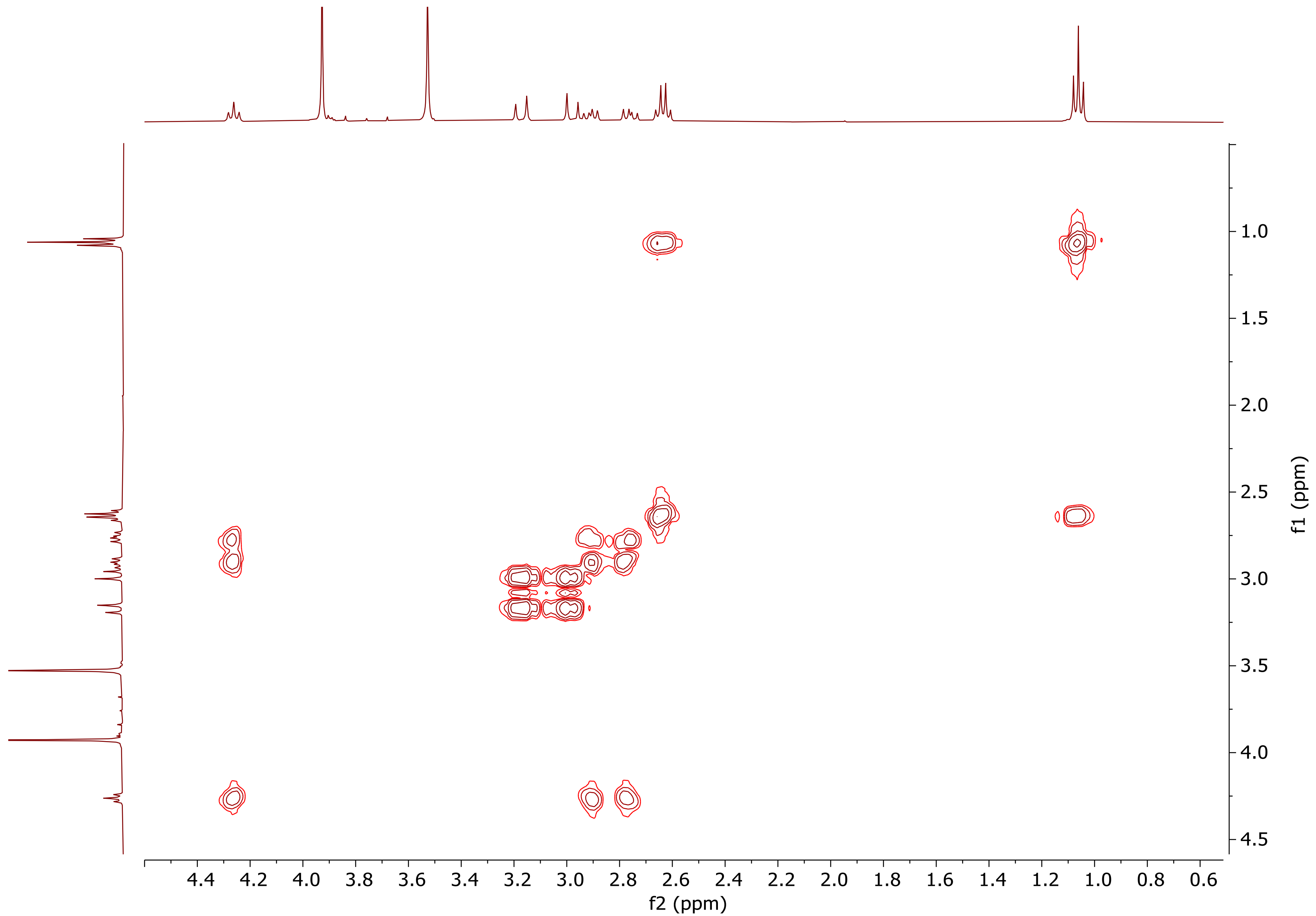

Figure S49: COSY spectrum for perenniporide G (**20)** ($CDCl_3$, 500 MHz).

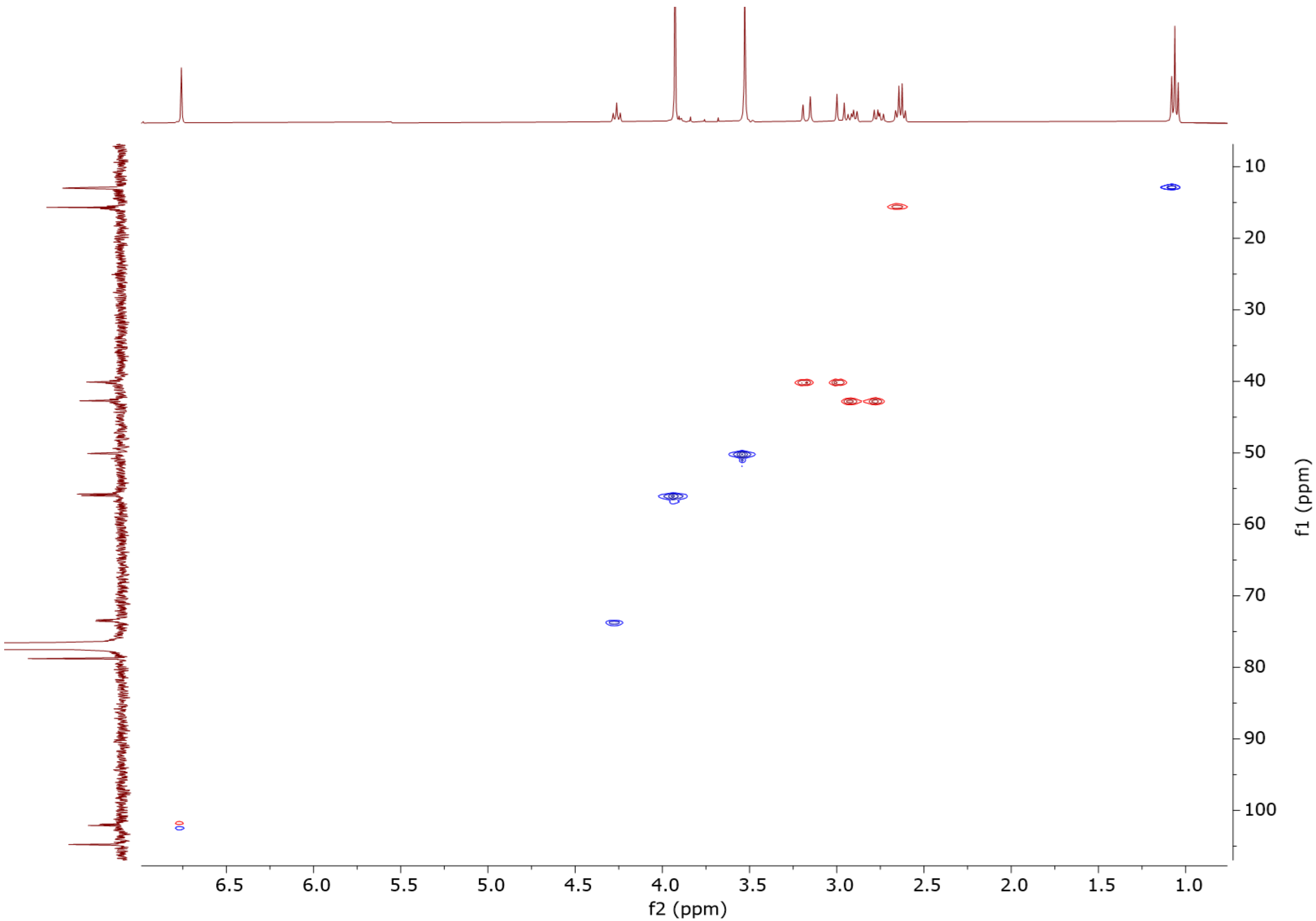

Figure S50: Edited HSQC spectrum for perenniporide G (**20**) ($CDCl_3$, 500 MHz).

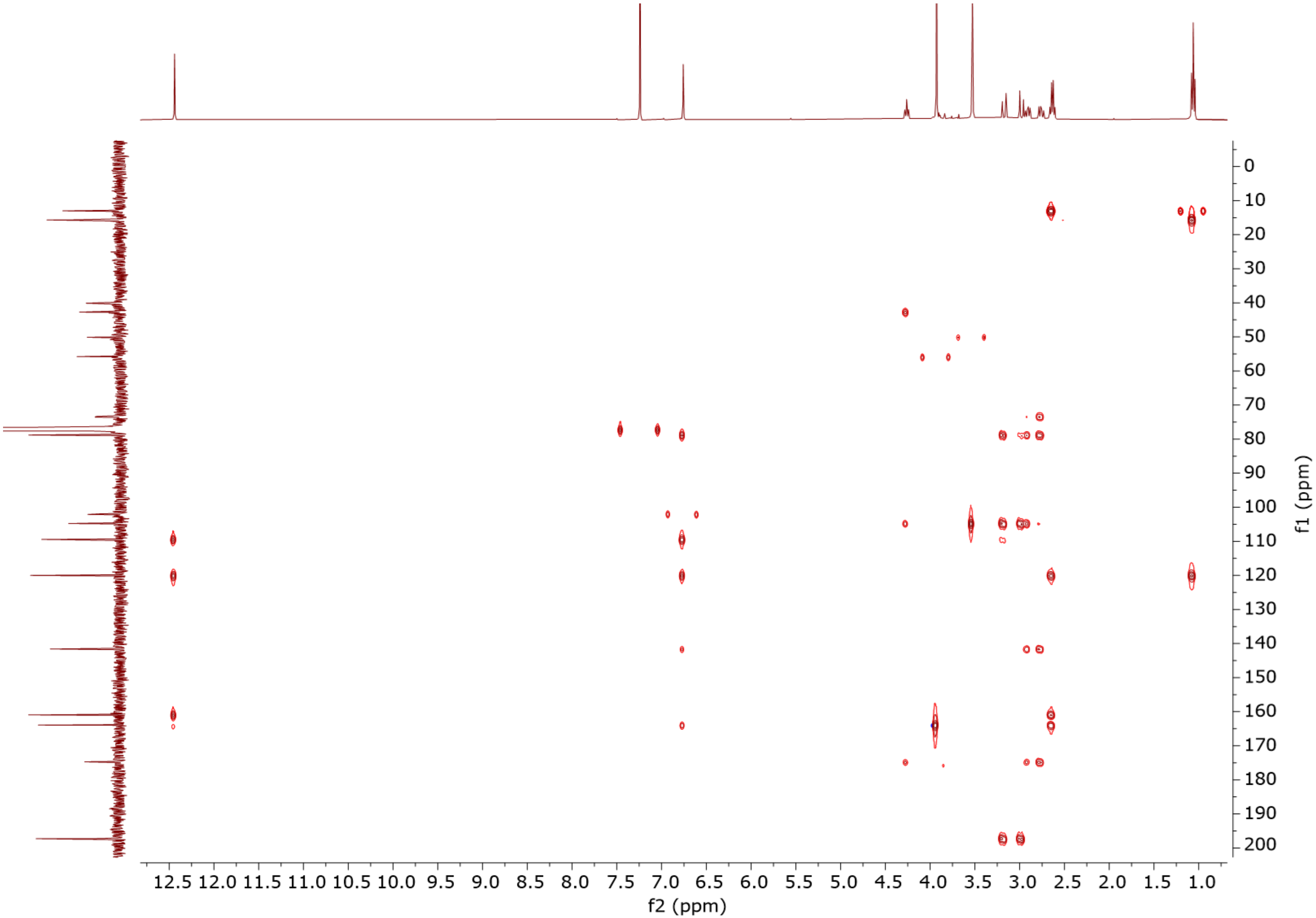

Figure S51: HMBC spectrum of perenniporide G (**20**) ($CDCl_3$, 500 MHz).

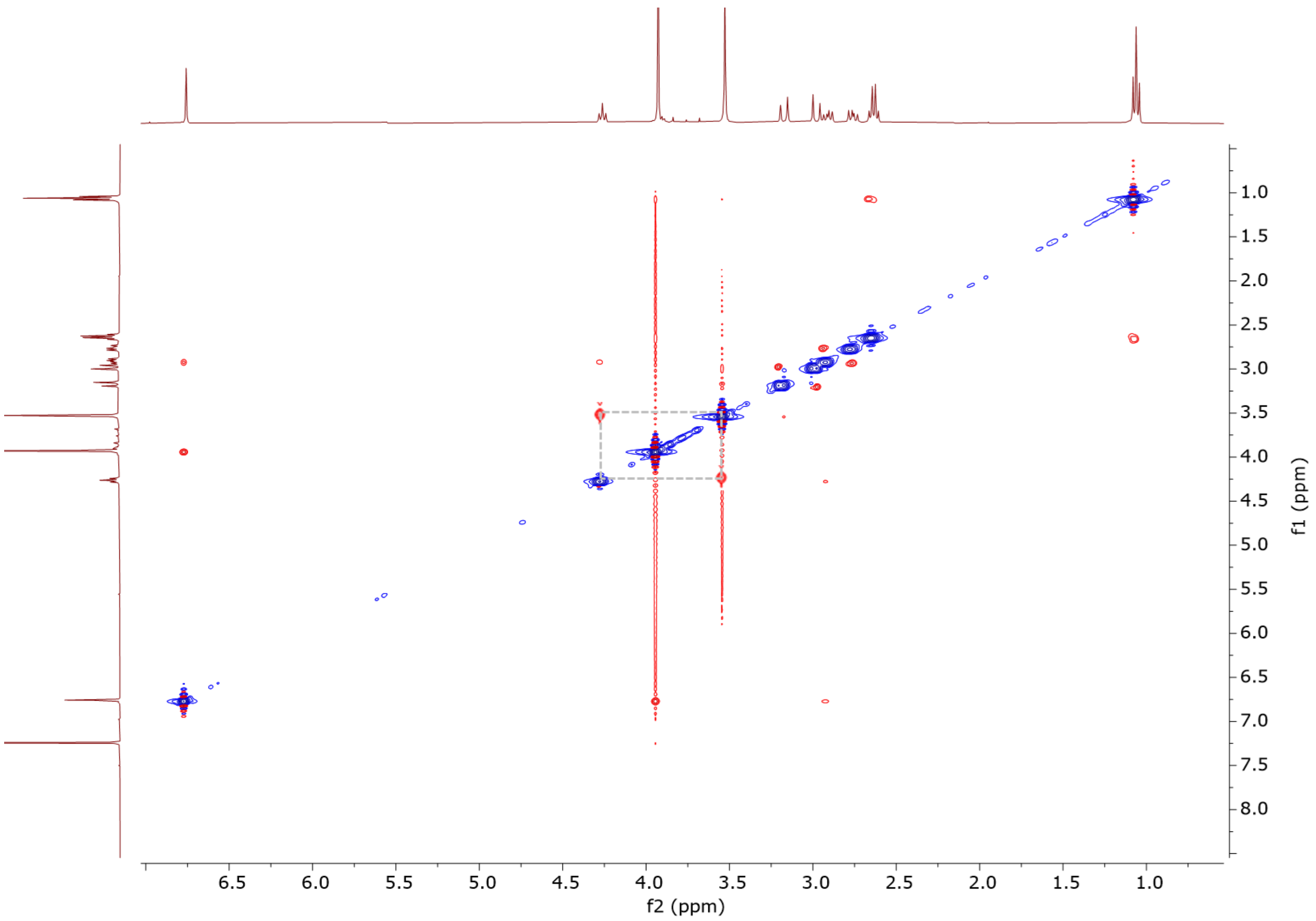

Figure S52:NOESY spectrum for perenniporide G (**20)** ($CDCl_3$, 500 MHz).

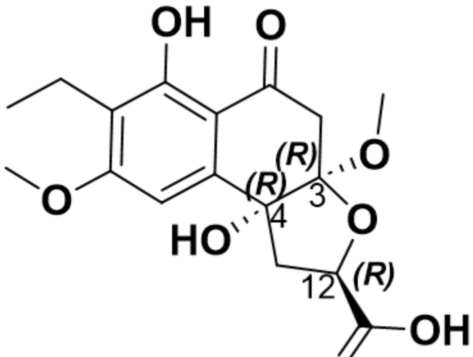

20A

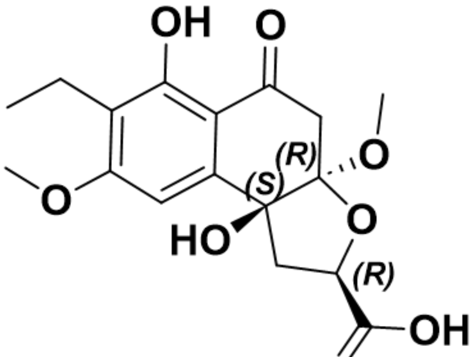

20B

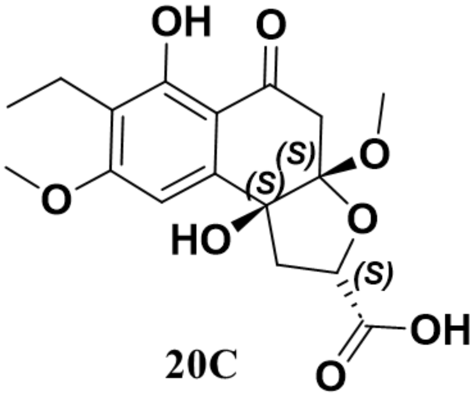

20C

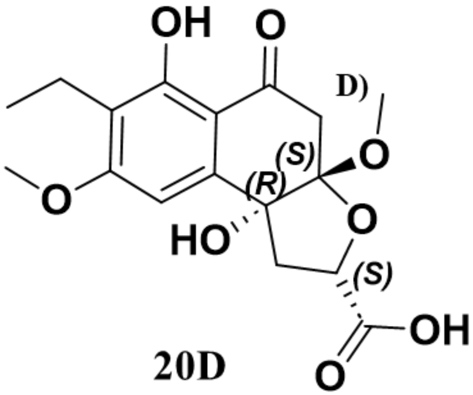

20D

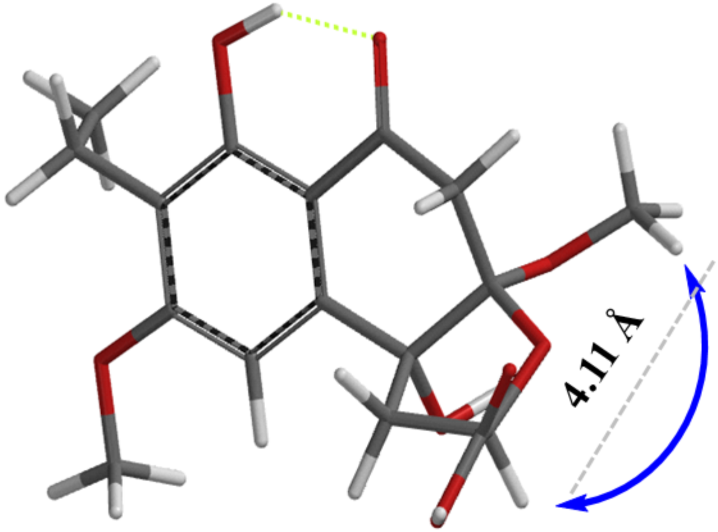

20A

Figure S53: Four possible conformers of **20** (**A**-**D**; left). Minimized structure of **20A**, showing the key NOESY correlation (blue arrow) and the distance between 3-OCH_3 and H-12, as they are on the same face (right).

Table S1: Calculated vs. experimental optical rotation of compound **20.**

OR experimental	Configuration	OR calculated	Configuration	OR calculated
**-23.5**	3*R*,4*R*,12*R*	-26.53	3*S*,4*S*,12*S*	26.53
	3*R*,4*R*,12*S*	-126.91	3*S*,4*S*,12*R*	126.91
	3*R*,4*S*,12*R*	-94.01	3*S*,4*R*,12*S*	94.01
	3*R*,4*S*,12*S*	-81.58	3*S*,4*R*,12*R*	81.58
Experimental data were collected in MeOH at a concentration of 0.1 g/100mL				

Table S2: Summary of absolute configuration determination (ECD_{calc}, OR_{calc} and $NOESY_{exp}$) of compound **20**.

#	Configuration	ECD	OR	NOESY (H-12 → 3-OCH_3)
**20A**	3*R*,4*R*,12*R*	✓	✓	✓
**20B**	3*R*,4*S*,12*R*	✗	✓	✓
**20C**	3*S*,4*S*,12*S*	✗	✗	✓
**20D**	3*S*,4*R*,12*S*	✗	✗	✓
**20E**	3*R*,4*R*,12*S*	✓	✓	✗
**20F**	3*R*,4*S*,12*S*	✗	✓	✗
**20G**	3*S*,4*S*,12*R*	✗	✗	✗
**20H**	3*S*,4*R*,12*R*	✗	✗	✗

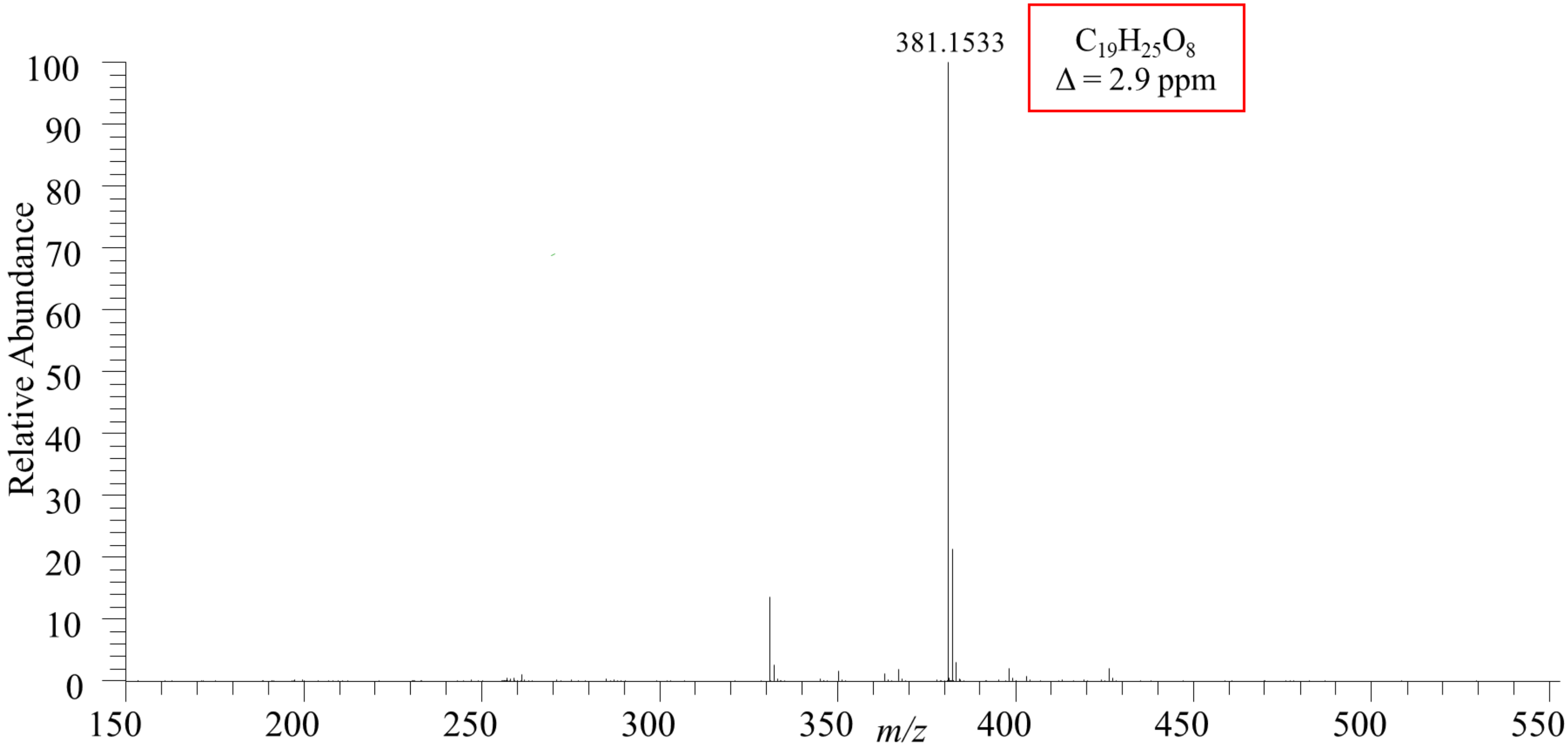

Figure S54: HRESIMS data for compound **21**.

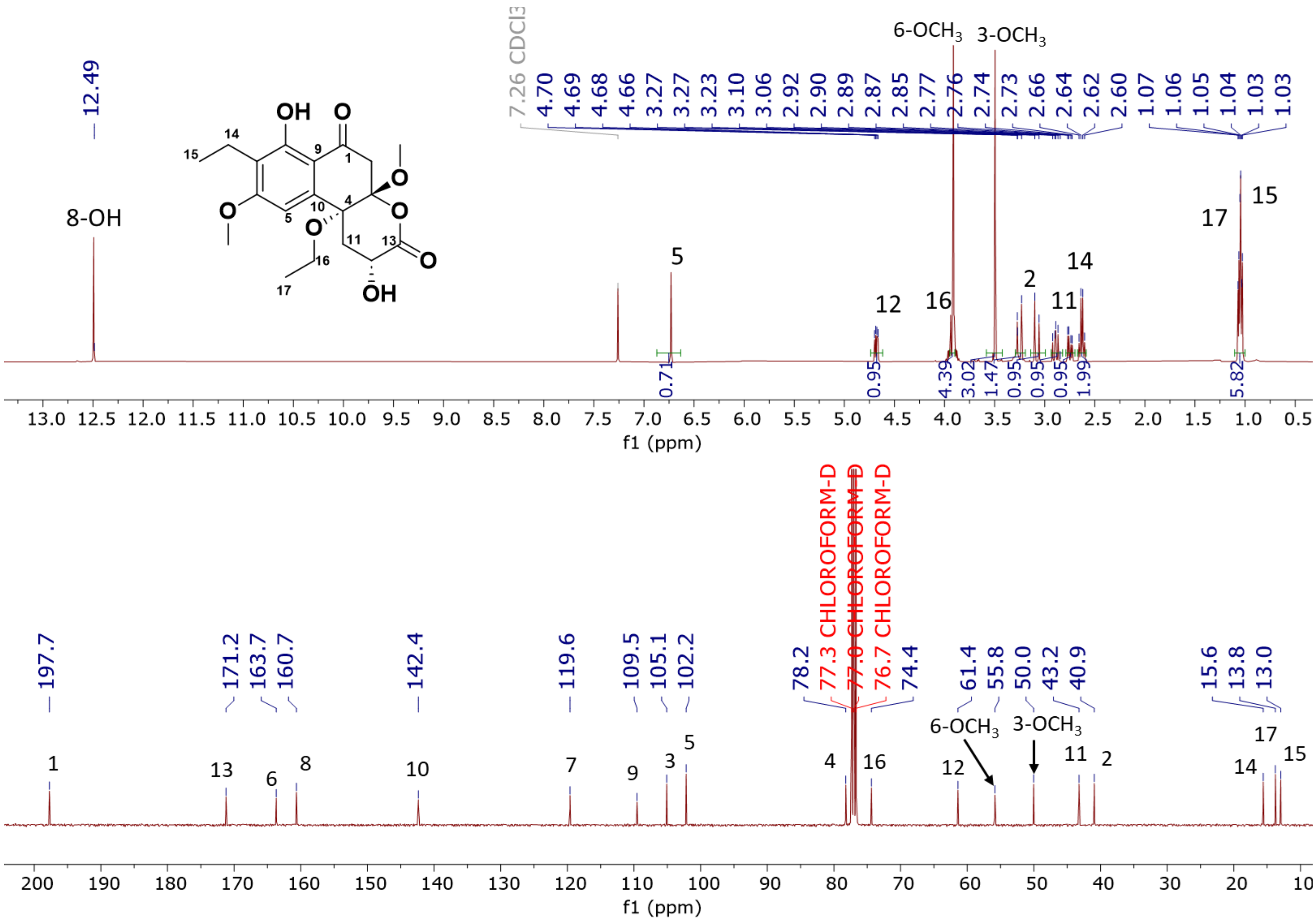

Figure S55: ^{1}H and ^{13}C NMR spectra for perenniporide H (**21**) ($CDCl_3$, 500 and 125 MHz, respectively).

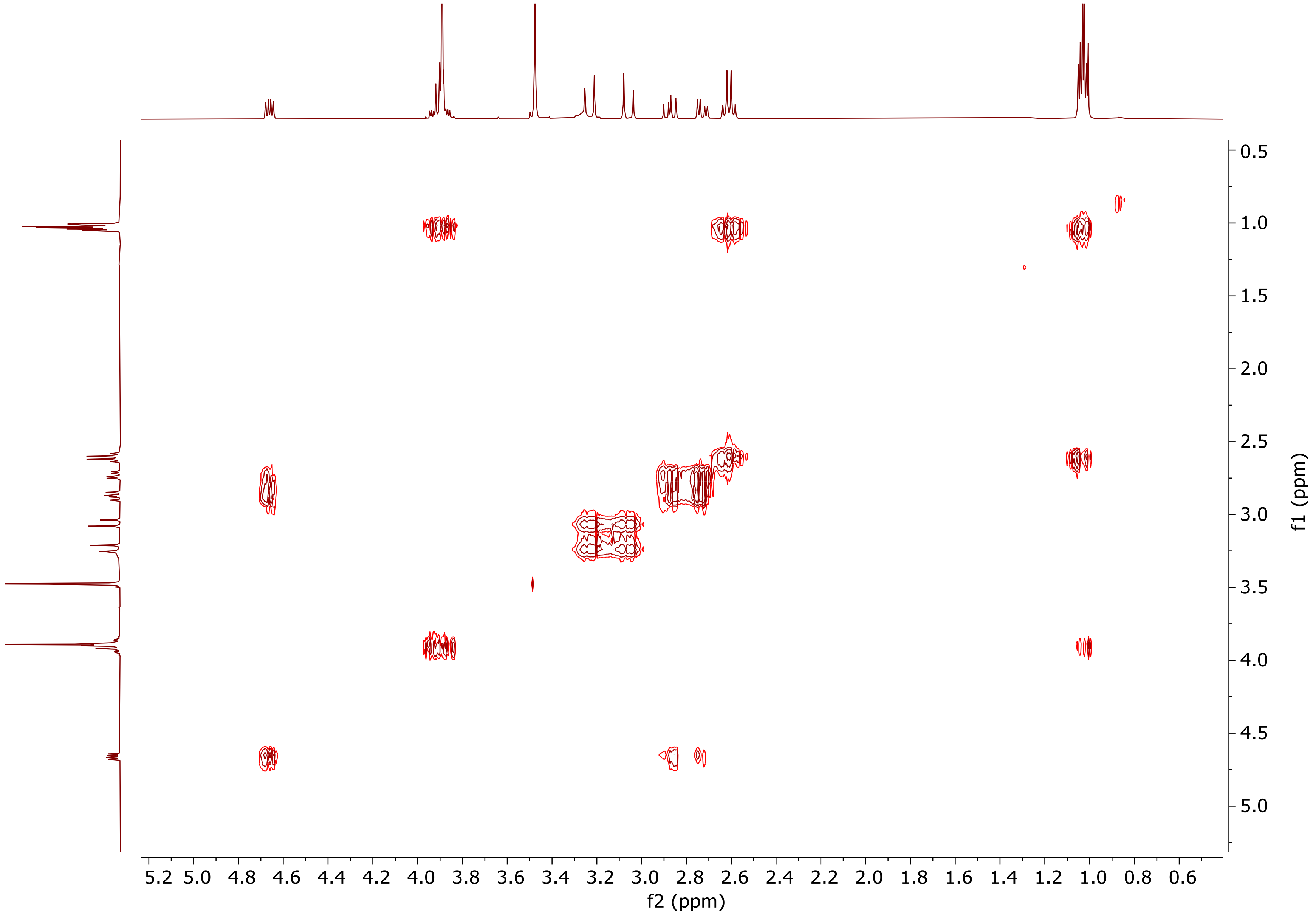

Figure S56: COSY spectrum for perenniporide H (**21**) ($CDCl_3$, 500 MHz).

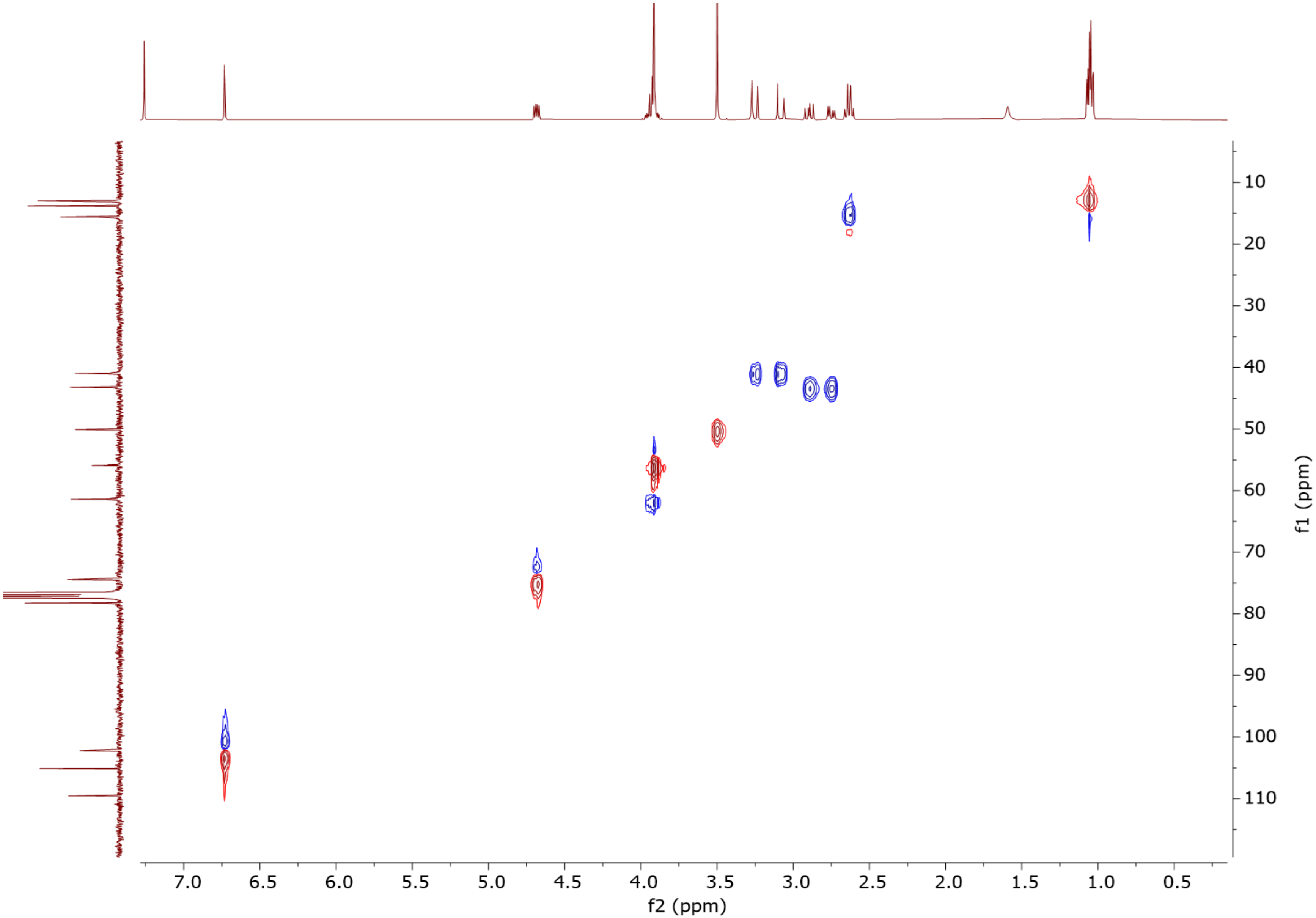

Figure S57: Edited HSQC spectrum for perenniporide H (**21**) ($CDCl_3$, 500 MHz).

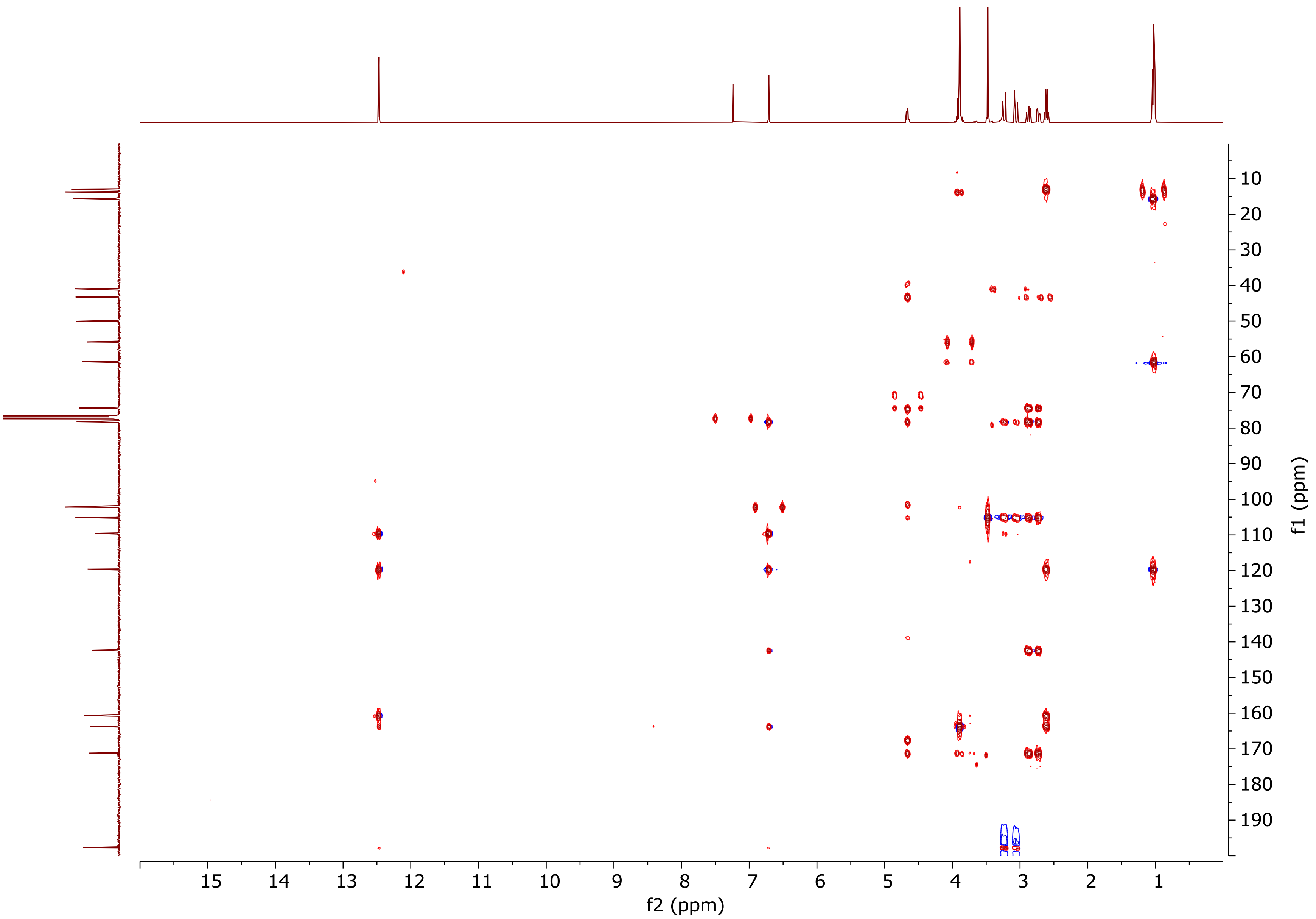

Figure S58: HMBC spectrum of perenniporide H (**21**) ($CDCl_3$, 500 MHz).

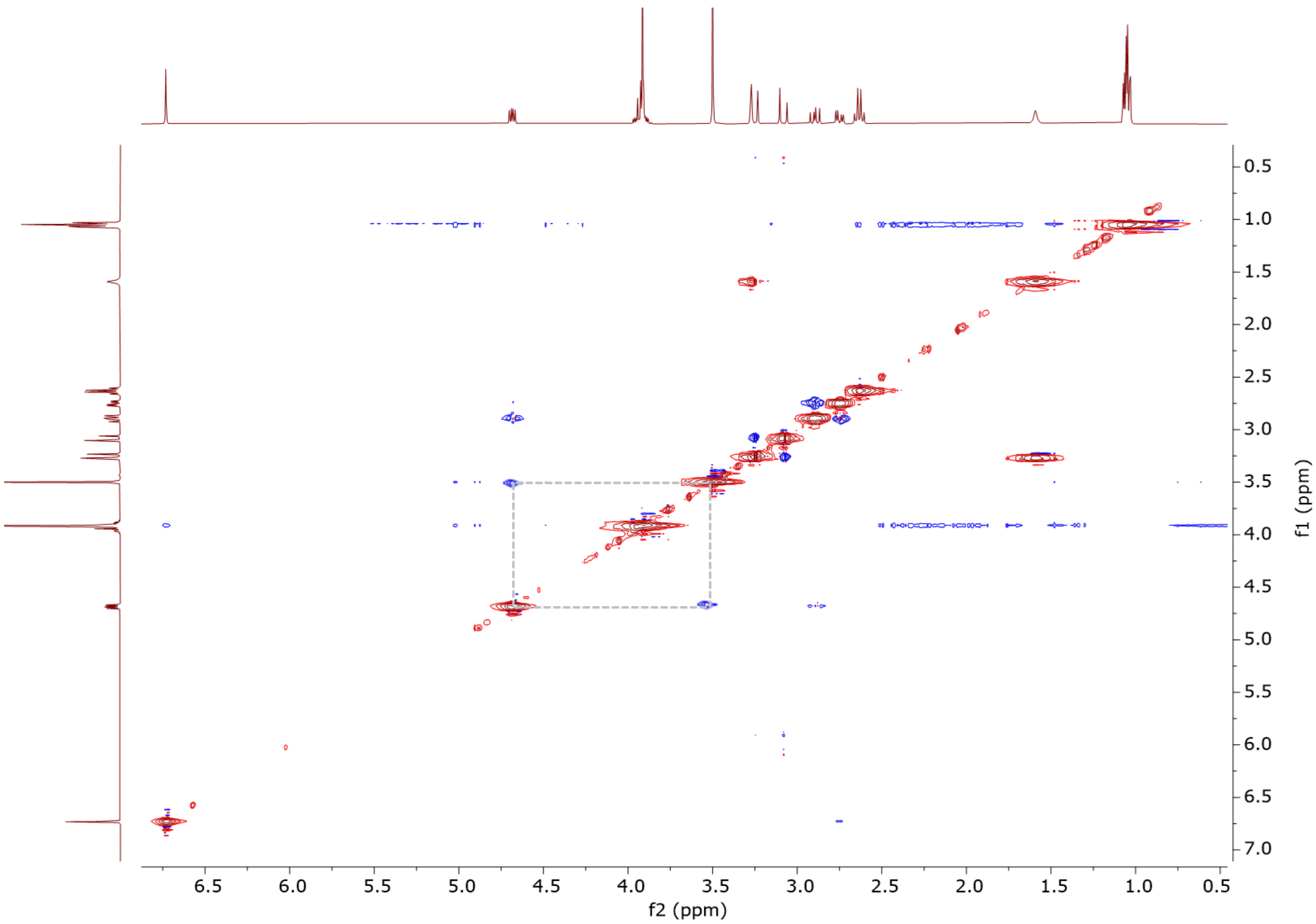

Figure S59: NOESY spectrum for perenniporide H (**21**) ($CDCl_3$, 500 MHz).

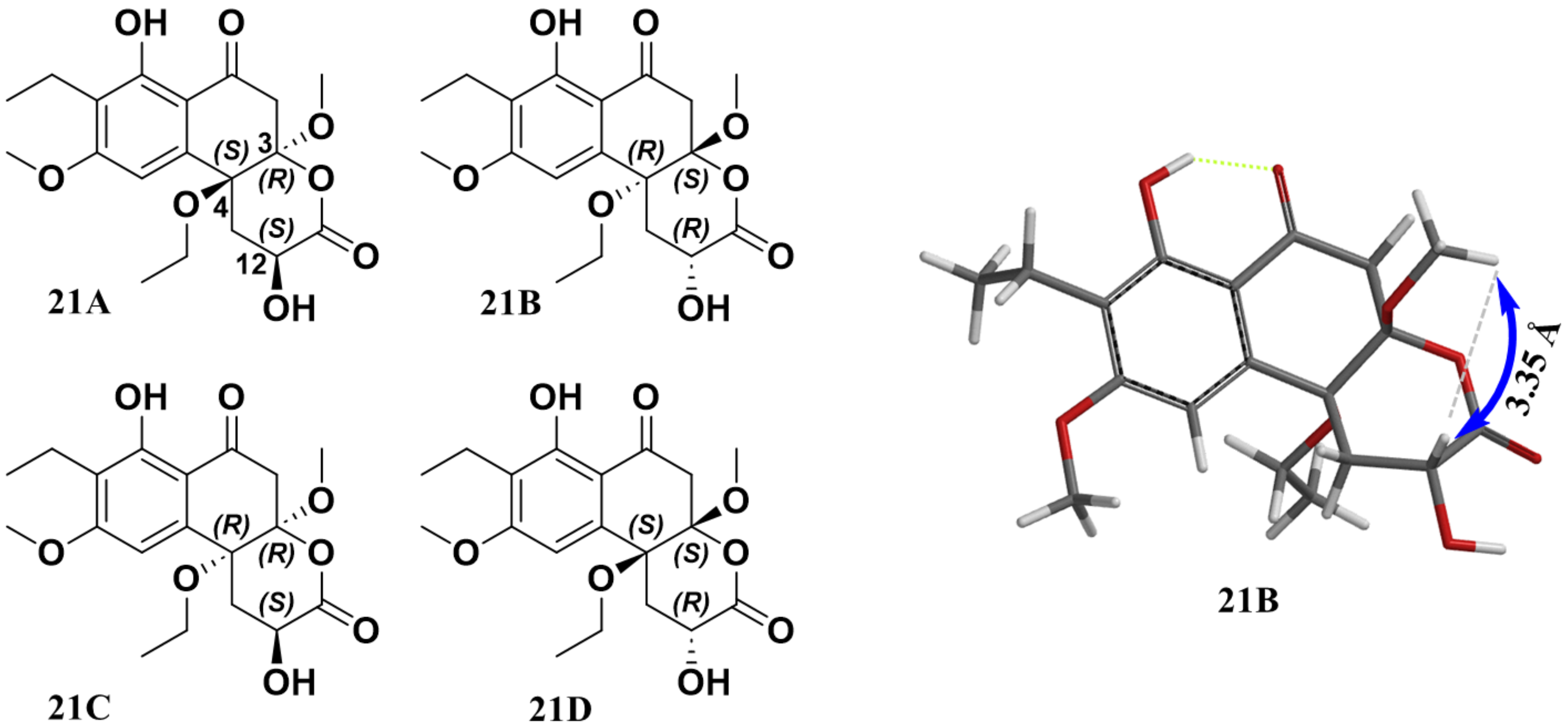

Figure S60: Four possible conformers of **21** (**A**-**D**; left). Minimized structure of **21B**, showing the key NOESY correlation (blue arrow) and the distance between 3-OCH_3 and H-12, as they are on the same face (right).

Table S3: Calculated vs. experimental optical rotation of compound **21.**

OR experimental	Configuration	OR calculated	Configuration	OR calculated
-56.8	3*S*,4*R*,12*R*	-33.27	3*R*,4*S*,12*S*	+33.27
	3*R*,4*R*,12*R*	-99.32	3*S*,4*S*,12*S*	+99.32
	3*R*,4*R*,12*S*	-77.77	3*S*,4*S*,12*R*	+77.77
	3*S*,4*R*,12*S*	-12.58	3*R*,4*S*,12*R*	+12.58
Experimental data were collected in MeOH at a concentration of 0.1 g/100mL				

Table S4: Summary of absolute configuration determination (ECD_{calc}, OR_{calc} and $NOESY_{exp}$) of compound **21**.

#	Configuration	ECD	OR	NOESY (H-12 → 3-OCH_3)
**21A**	3*R*,4*S*,12*S*	✗	✗	✓
**21B**	3*S*,4*R*,12*R*	✓	✓	✓
**21C**	3*R*,4*R*,12*S*	✗	✓	✓
**21D**	3*S*,4*S*,12*R*	✗	✗	✓
**21E**	3*S*,4*R*,12*S*	✓	✓	✗
**21F**	3*R*,4*R*,12*R*	✗	✓	✗
**21G**	3*R*,4*S*,12*R*	✗	✗	✗
**21H**	3*S*,4*S*,12*S*	✗	✗	✗

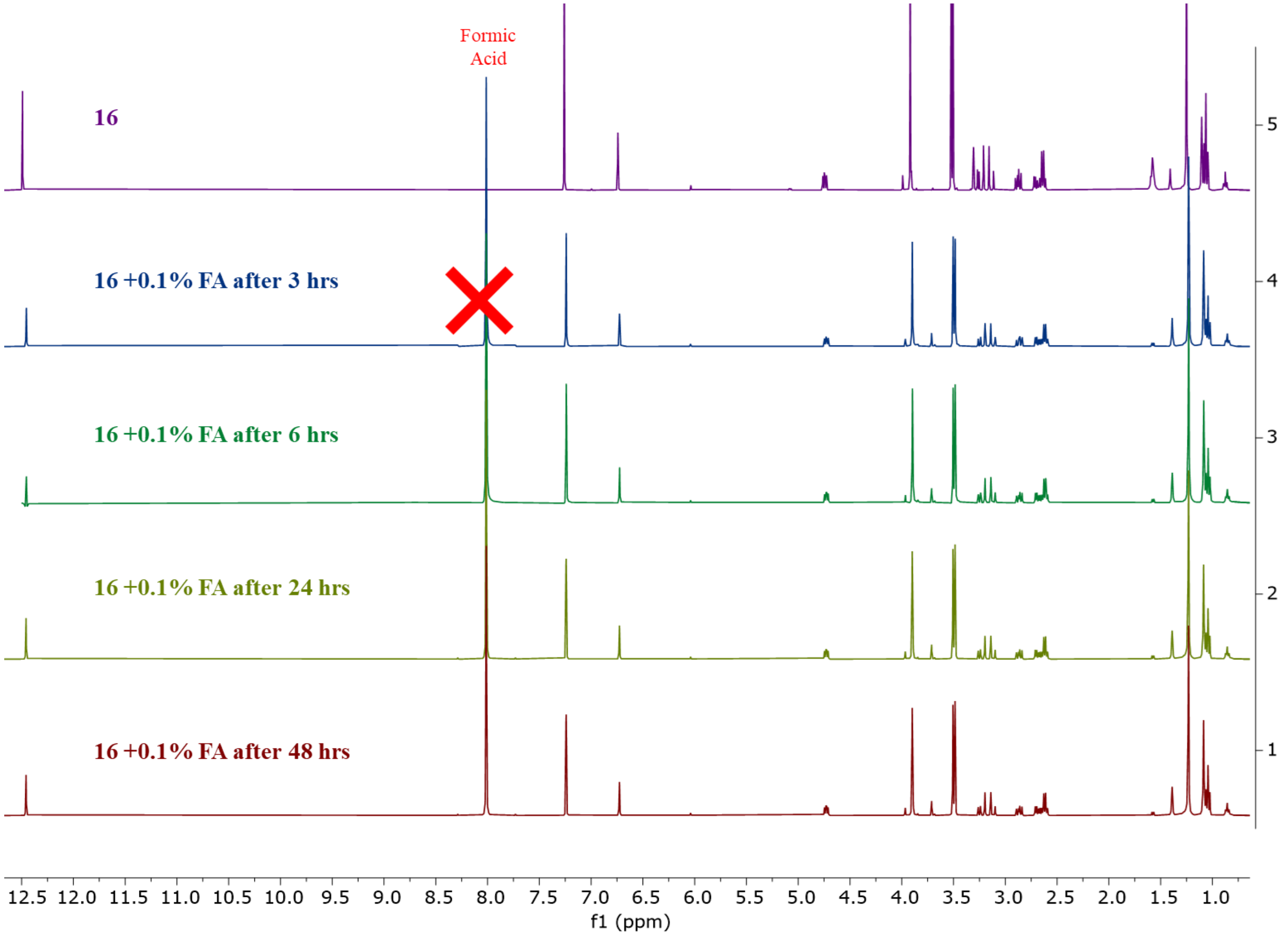

Figure S61: Stacked ^{1}H NMR spectra of **16** incubated with 0.1% formic acid after 3, 6, 24, and 48 hours ($CDCl_3$, 400 MHz).

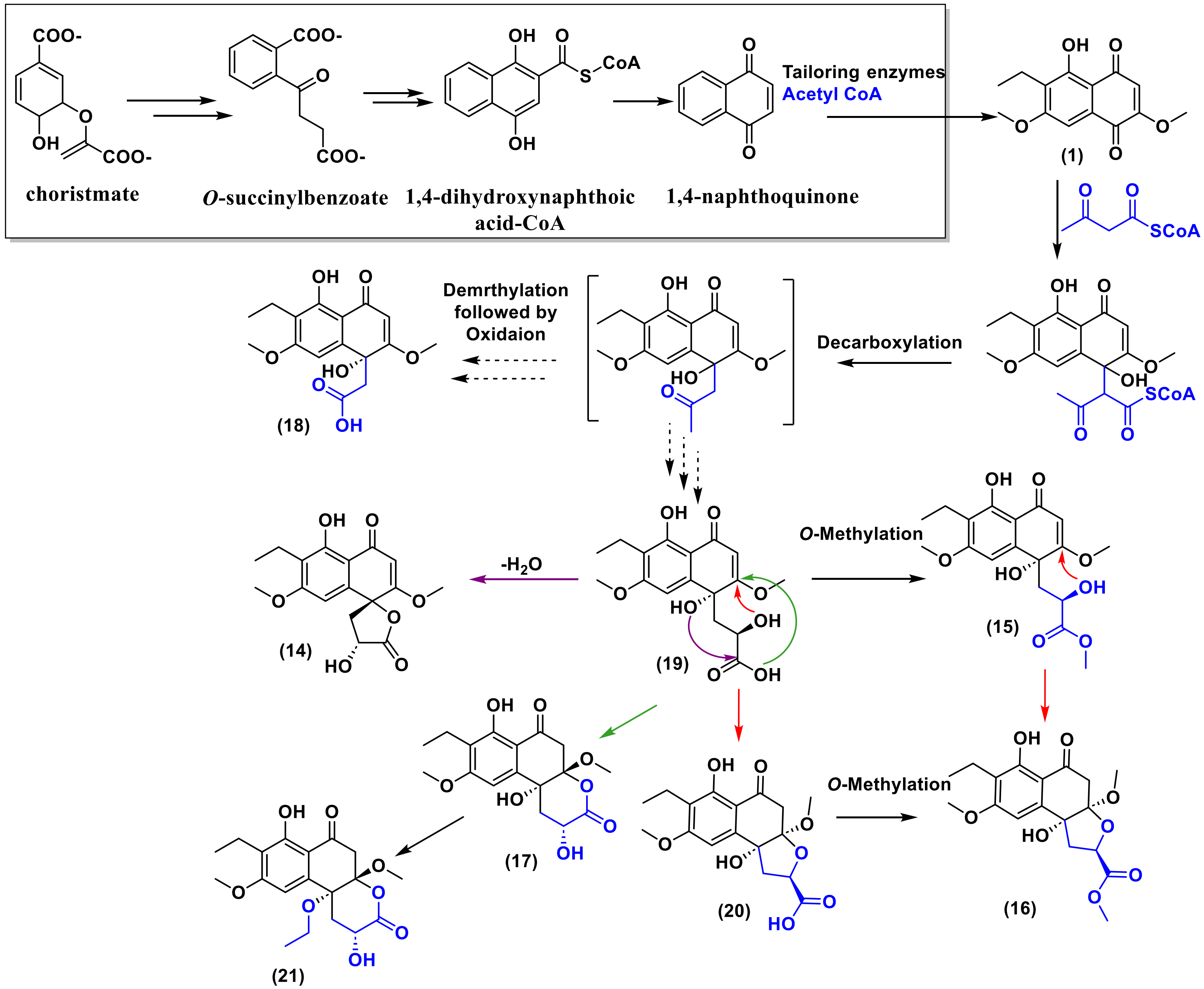

Figure S62: Proposed biosynthesis of perenniporides A-H (*i.e.,* compounds **14-21**).

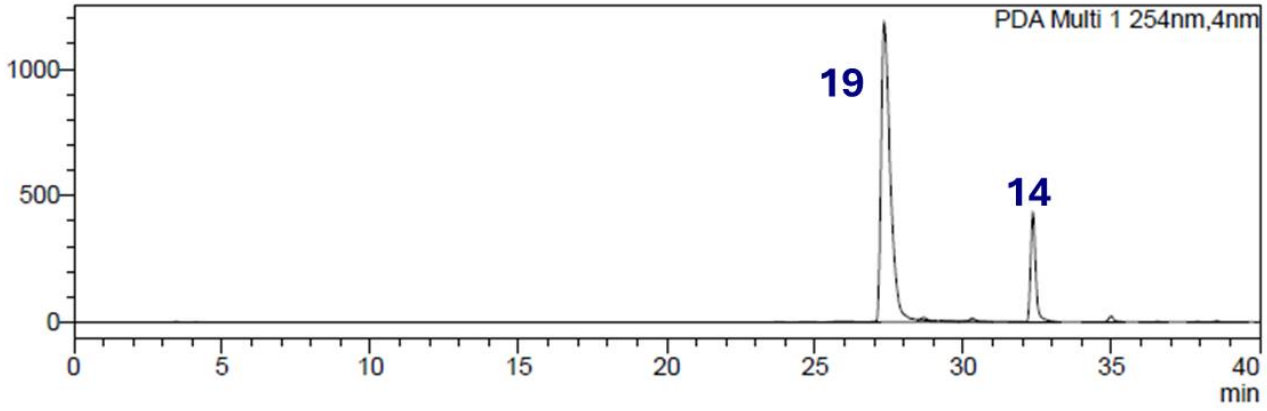

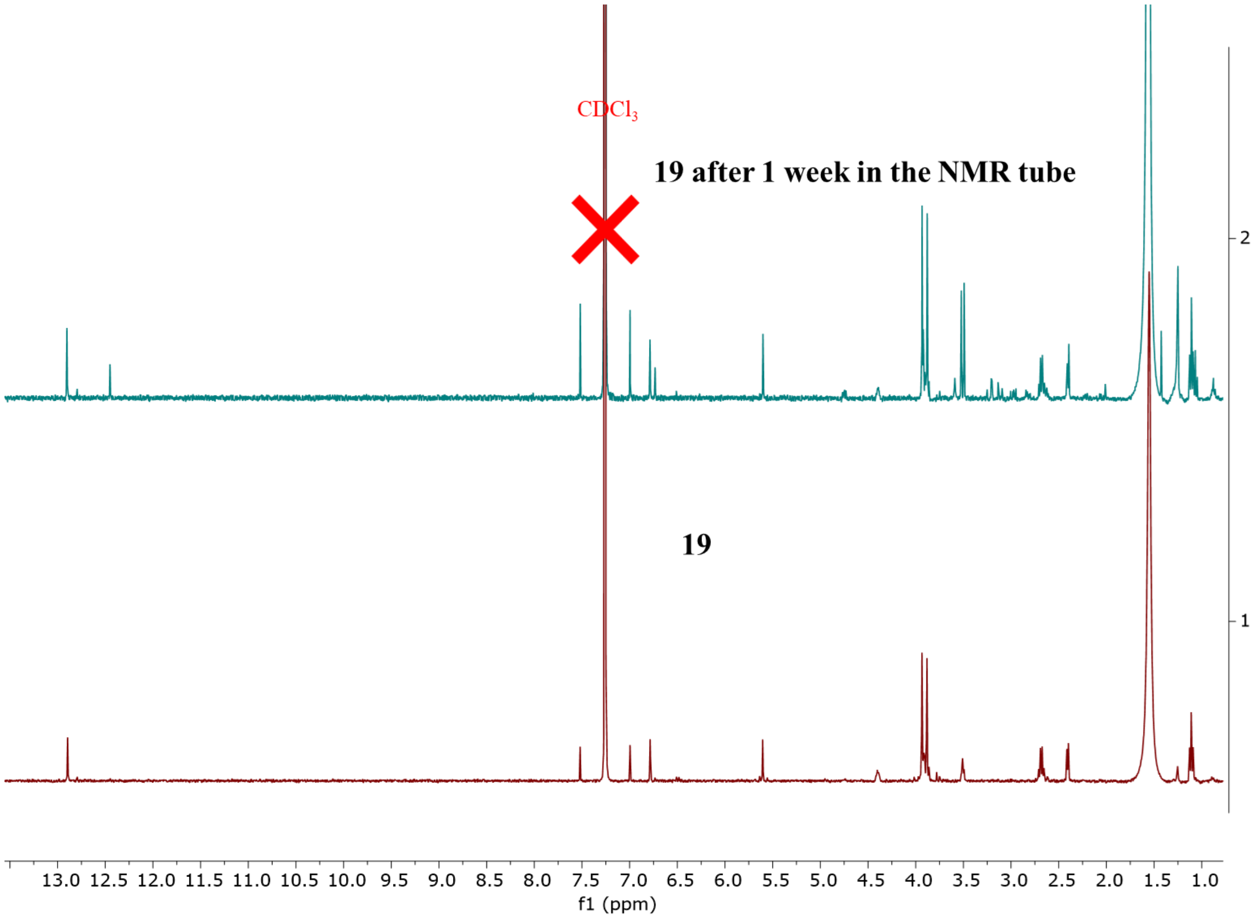

Figure S63: Chromatographic separation of compound **19** after one week in an NMR tube in $CDCl_3$, indicating partial conversion to compound **14** (top). Stacked ^1H NMR spectra of **19** before and after purification ($CDCl_3$, 400 MHz) (bottom).

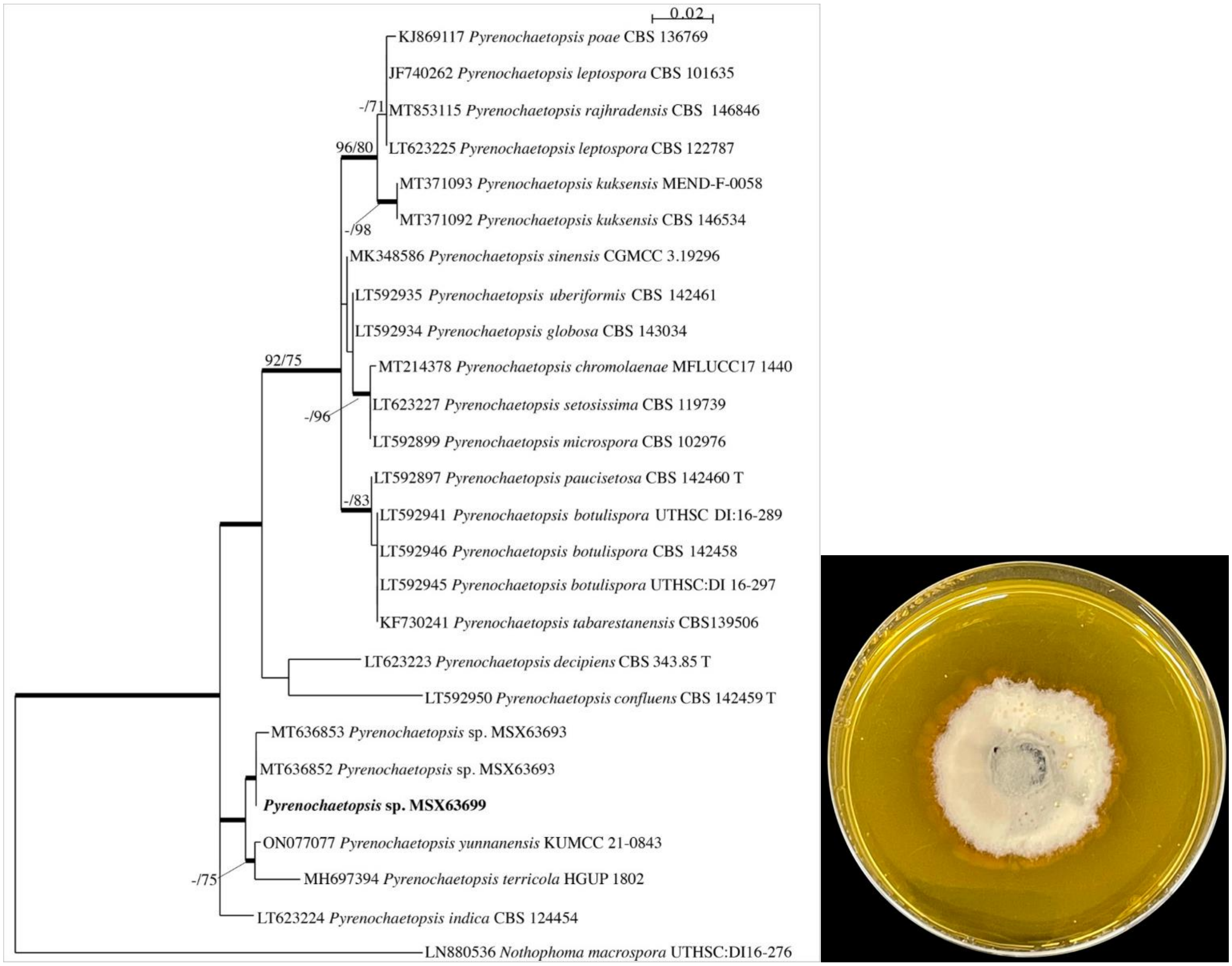

Figure S64: Phylogram of the most likely tree (-lnL = 1627.382) from an IQ-TREE analysis v2.2.0 of 26 taxa based on ITS data (547 bp) under Numbers refer to UFBoot support values ≥ 90% based on 5000 replicates followed by PHYML bootstrap values. For IQ tree nodes ≥ 95% are considered strongly supported, while nodes ≥70% are considered significant for PHYML bootstrap analysis. Thickened branches indicate significant Bayesian posterior probabilities ≥ 95%. Bayesian analysis was calculated using Mr. Bayes v. 3.2 under TIM2e+G4 for ITS region. The MSX63699 strain is highlighted in bold. A 30 days old culture on malt extract agar is shown on the right. Scale bar indicates the expected number of nucleotide substitutions per site.

Table S5:SMILES, InChI, and InChIkey descriptors of **1-21**.

Compound	SMILES	InChI	InChIkey
**1**	CCC(C(OC)=CC(C(C(OC)=C1)=O)=C2C1=O)=C2O	InChI=1S/C14H14O5/c1-4-7-10(18-2)5-8-12(14(7)17)9(15)6-11(19-3)13(8)16/h5-6,17H,4H2,1-3H3	KRSHMRFACGPISX-UHFFFAOYSA-N
**2**	OC1=C(C(O)C)C(OC)=CC2=C1C(C=C(OC)C2=O)=O	InChI=1S/C14H14O6/c1-6(15)11-9(19-2)4-7-12(14(11)18)8(16)5-10(20-3)13(7)17/h4-6,15,18H,1-3H3	DUFFAWHPHNGPDG-UHFFFAOYSA-N
**3**	O=C1C=C(OC)C(C2=CC(O)=C(CC)C(O)=C21)=O	InChI=1S/C13H12O5/c1-3-6-8(14)4-7-11(13(6)17)9(15)5-10(18-2)12(7)16/h4-5,14,17H,3H2,1-2H3	FNWDXGLBKMXTNQ-UHFFFAOYSA-N
**4**	O=C(OC(C(C(OC)=CC(C(C(OC)=C1)=O)=C2C1=O)=C2O)C)C	InChI=1S/C16H16O7/c1-7(23-8(2)17)13-11(21-3)5-9-14(16(13)20)10(18)6-12(22-4)15(9)19/h5-7,20H,1-4H3	SNIIQTSBSNAYGY-UHFFFAOYSA-N
**5**	O=C1C=C(OC)C(C2=CC(OC)=C(C(OC)C)C(O)=C21)=O	InChI=1S/C15H16O6/c1-7(19-2)12-10(20-3)5-8-13(15(12)18)9(16)6-11(21-4)14(8)17/h5-7,18H,1-4H3	MPVDQQCJVAESHC-UHFFFAOYSA-N
**6**	O=C1C=C(OC)C(C2=CC(OC)=C(C)C(O)=C21)=O	InChI=1S/C13H12O5/c1-6-9(17-2)4-7-11(12(6)15)8(14)5-10(18-3)13(7)16/h4-5,15H,1-3H3	GWPDLTIZFFIWSZ-UHFFFAOYSA-N
**7**	OC1=C(CC)C(OC)=CC([C@H](O)[C@H](OC)C2)=C1C2=O	InChI=1S/C14H18O5/c1-4-7-10(18-2)5-8-12(14(7)17)9(15)6-11(19-3)13(8)16/h5,11,13,16-17H,4,6H2,1-3H3/t11-,13+/m1/s1	YLSOOYDKHUEZQR-YPMHNXCESA-N
**8**	OC1=C(C(C)C(C(C2=C3O)=O)=C(O)C(C2=CC(OC)=C3)=O)C(OC)=CC4=C1C(C=C(OC)C4=O)=O	InChI=1S/C25H20O10/c1-9(18-24(31)19-11(22(29)25(18)32)5-10(33-2)6-13(19)26)17-15(34-3)7-12-20(23(17)30)14(27)8-16(35-4)21(12)28/h5-9,26,30,32H,1-4H3	XCPBYQWGHDPROP-UHFFFAOYSA-N
**9**	OC1=C(C(C)C(C(C2=C3O)=O)=C(O)C(C2=CC(O)=C3C(C)=O)=O)C(OC)=CC4=C1C(C=C(OC)C4=O)=O	InChI=1S/C26H20O11/c1-8(16-14(36-3)6-11-19(23(16)32)13(29)7-15(37-4)21(11)30)17-24(33)20-10(22(31)26(17)35)5-12(28)18(9(2)27)25(20)34/h5-8,28,32,34-35H,1-4H3	BBMLPMPNKPPBCD-UHFFFAOYSA-N
**10**	OC1=C(C(C)C(C(C2=C3O)=O)=C(O)C(C2=CC(OC)=C3CC)=O)C(OC)=CC4=C1C(C=C(OC)C4=O)=O	InChI=1S/C27H24O10/c1-6-11-15(35-3)7-13-21(23(11)30)26(33)19(27(34)24(13)31)10(2)18-16(36-4)8-12-20(25(18)32)14(28)9-17(37-5)22(12)29/h7-10,30,32,34H,6H2,1-5H3	AUGCJFLSEXRNSB-UHFFFAOYSA-N
**11**	OC1=C(C(C)C(C(C2=C3O)=O)=C(OC)C(C2=CC(OC)=C3CC)=O)C(OC)=CC4=C1C(C=C(OC)C4=O)=O	InChI=1S/C28H26O10/c1-7-12-16(35-3)8-14-22(24(12)31)27(34)20(28(38-6)25(14)32)11(2)19-17(36-4)9-13-21(26(19)33)15(29)10-18(37-5)23(13)30/h8-11,31,33H,7H2,1-6H3	IPHLUTXEZWZYON-UHFFFAOYSA-N

**12**	OC1=C(C(C)C(C(C2=C3O)=O)=C(O)C(C2=CC(OC)=C3CCO)=O)C(OC)=CC4=C1C(C=C(OC)C4=O)=O	InChI=1S/C27H24O11/c1-10(18-16(37-3)8-12-20(25(18)33)14(29)9-17(38-4)22(12)30)19-26(34)21-13(24(32)27(19)35)7-15(36-2)11(5-6-28)23(21)31/h7-10,28,31,33,35H,5-6H2,1-4H3	RVJVIUVVBWPIIY-UHFFFAOYSA-N
**13**	OC1=C(C(C)C(C(C2=C3O)=O)=C(O)C(C2=CC(OC)=C3CC)=O)C(OC)=CC4=C1C(C=C(O)C4=O)=O	InChI=1S/C26H22O10/c1-5-10-15(35-3)6-12-20(22(10)30)25(33)18(26(34)23(12)31)9(2)17-16(36-4)7-11-19(24(17)32)13(27)8-14(28)21(11)29/h6-9,28,30,32,34H,5H2,1-4H3	NIHYXLCAFOVNKA-UHFFFAOYSA-N
**14**	O=C1C=C(OC)[C@@]2(C[C@@H](O)C(O2)=O)C3=CC(OC)=C(CC)C(O)=C31	InChI=1S/C17H18O7/c1-4-8-12(22-2)5-9-14(15(8)20)10(18)6-13(23-3)17(9)7-11(19)16(21)24-17/h5-6,11,19-20H,4,7H2,1-3H3/t11-,17-/m1/s1	UKDUEMGCYIGYBD-PIGZYNQJSA-N
**15**	O=C1C=C(OC)[C@](C[C@@H](O)C(OC)=O)(O)C2=CC(OC)=C(CC)C(O)=C21	InChI=1S/C18H22O8/c1-5-9-13(24-2)6-10-15(16(9)21)11(19)7-14(25-3)18(10,23)8-12(20)17(22)26-4/h6-7,12,20-21,23H,5,8H2,1-4H3/t12-,18-/m1/s1	WZZOARZEYMJHRI-KZULUSFZSA-N
**16**	COC([C@H](O[C@@]1(C2)OC)C[C@@]1(O)C(C=C3OC)=C(C(O)=C3CC)C2=O)=O	InChI=1S/C18H22O8/c1-5-9-12(23-2)6-10-14(15(9)20)11(19)7-18(25-4)17(10,22)8-13(26-18)16(21)24-3/h6,13,20,22H,5,7-8H2,1-4H3/t13-,17-,18-/m1/s1	OQWJPFFTMZHPBR-FSPWUOQZSA-N
**17**	O=C1C[C@@](OC2=O)(OC)[C@](C[C@H]2O)(O)C3=CC(OC)=C(CC)C(O)=C31	InChI=1S/C17H20O8/c1-4-8-12(23-2)5-9-13(14(8)20)10(18)7-17(24-3)16(9,22)6-11(19)15(21)25-17/h5,11,19-20,22H,4,6-7H2,1-3H3/t11-,16-,17+/m1/s1	SEXRKUXABLFHSA-LQAWEQHXSA-N
**18**	O=C1C=C(OC)[C@](CC(O)=O)(O)C2=CC(OC)=C(CC)C(O)=C21	InChI=1S/C16H18O7/c1-4-8-11(22-2)5-9-14(15(8)20)10(17)6-12(23-3)16(9,21)7-13(18)19/h5-6,20-21H,4,7H2,1-3H3,(H,18,19)/t16-/m1/s1	UIAXRHPHQRCXEF-MRXNPFEDSA-N
**19**	O=C1C=C(OC)[C@](C[C@@H](O)C(O)=O)(O)C2=CC(OC)=C(CC)C(O)=C21	InChI=1S/C17H20O8/c1-4-8-12(24-2)5-9-14(15(8)20)10(18)6-13(25-3)17(9,23)7-11(19)16(21)22/h5-6,11,19-20,23H,4,7H2,1-3H3,(H,21,22)/t11-,17-/m1/s1	GYDHNRQGLDWRHK-PIGZYNQJSA-N
**20**	O=C(C1=C(O)C(CC)=C(OC)C=C1[C@]2(O)C[C@@H](O3)C(O)=O)C[C@]23OC	InChI=1S/C17H20O8/c1-4-8-11(23-2)5-9-13(14(8)19)10(18)6-17(24-3)16(9,22)7-12(25-17)15(20)21/h5,12,19,22H,4,6-7H2,1-3H3,(H,20,21)/t12-,16-,17-/m1/s1	SPMMWCLUYGKJAW-CSMYWGQOSA-N
**21**	O=C1C[C@@]2(OC)[C@](C[C@H](C(O2)=O)O)(OCC)C3=CC(OC)=C(CC)C(O)=C31	InChI=1S/C19H24O8/c1-5-10-14(24-3)7-11-15(16(10)22)12(20)9-19(25-4)18(11,26-6-2)8-13(21)17(23)27-19/h7,13,21-22H,5-6,8-9H2,1-4H3/t13-,18-,19+/m1/s1	DHXQUWHKCPWOEI-ZNOIYHFQSA-N

Table S6: Energies and Boltzmann distribution of compound **18** (4*R*).

Conformer	Hartrees	kcal/mol	ΔG (kcal/mol)	Boltzmann Distribution (%)
Conformer 6	-1146.93664	-719713.763	0	34.998673
Conformer 5	-1146.93632	-719713.56	0.2026856	24.0926292
Conformer 8	-1146.93539	-719712.976	0.78689705	8.21221993
Conformer 7	-1146.93528	-719712.907	0.85592311	7.23158159
Conformer 1	-1146.93528	-719712.905	0.85717812	7.21488076
Conformer 3	-1146.93515	-719712.824	0.93875437	6.20812643
Conformer 4	-1146.93478	-719712.595	1.16779538	4.07104959
Conformer 2	-1146.93471	-719712.548	1.2148586	3.7329436
Conformer 9	-1146.93391	-719712.046	1.71686629	1.48046612
Conformer 10	-1146.9337	-719711.918	1.84425074	1.17079009
Conformer 11	-1146.93346	-719711.764	1.9986181	0.88098954
Conformer 12	-1146.93327	-719711.644	2.11909995	0.70562514
Conformer 13	-1146.92413	-719705.911	7.85202773	1.8268E-05
Conformer 14	-1146.92326	-719705.368	8.39482354	6.7206E-06

Table S7: XYZ coordinates of compound **18** (4*R*).

		**Conformer 1**			**Conformer 2**			**Conformer 3**			**Conformer 4**		
Row	Symbol	X	Y	Z	X	Y	Z	X	Y	Z	X	Y	Z
1	C	-1.571517	1.369813	0.296705	-1.529399	1.468093	-0.203242	-1.605264	1.357176	0.276903	-1.568212	1.444815	-0.220935
2	C	-0.979038	-1.236515	-0.525170	-0.968866	-1.236405	-0.641277	-1.008911	-1.271037	-0.468385	-1.000499	-1.268507	-0.589122
3	C	-0.224309	1.009286	0.002370	-0.175566	1.030580	-0.290049	-0.259370	0.992994	-0.018784	-0.213573	1.010514	-0.311176
4	C	-2.623537	0.449701	0.186625	-2.603764	0.578088	-0.341852	-2.654288	0.430046	0.205199	-2.640057	0.546910	-0.321945
5	C	-2.303110	-0.852584	-0.242317	-2.297237	-0.777710	-0.566758	-2.332061	-0.883770	-0.185452	-2.329995	-0.813464	-0.511277
6	C	0.050872	-0.313171	-0.398104	0.081581	-0.338905	-0.498032	0.017876	-0.340129	-0.380484	0.047371	-0.363136	-0.484179
7	H	-0.756269	-2.242819	-0.851890	-0.757202	-2.282172	-0.816012	-0.784643	-2.286236	-0.765187	-0.786271	-2.317826	-0.737344
8	C	0.837098	2.015339	0.055163	0.918601	2.000405	-0.228737	0.798381	2.003418	-0.003991	0.876726	1.985529	-0.285199
9	C	2.177582	1.648454	-0.362294	2.279586	1.541750	-0.436754	2.136646	1.629362	-0.424757	2.237775	1.526863	-0.497446
10	H	2.913806	2.442505	-0.375109	3.048742	2.303796	-0.455771	2.869251	2.425710	-0.468692	3.003271	2.291386	-0.545165
11	C	2.475869	0.376645	-0.711337	2.556008	0.227667	-0.595174	2.434316	0.350928	-0.750066	2.517166	0.211002	-0.635913
12	C	1.479197	-0.773409	-0.662646	1.507769	-0.874603	-0.528003	1.444980	-0.802299	-0.647599	1.475254	-0.893752	-0.520400
13	O	-3.355017	-1.705603	-0.356739	-3.367651	-1.604029	-0.703179	-3.381121	-1.744225	-0.263300	-3.398177	-1.647767	-0.610650
14	C	-4.046928	0.856434	0.492510	-4.032356	1.062532	-0.245598	-4.076450	0.840303	0.512026	-4.069739	1.027552	-0.222637
15	H	-4.579668	-0.000865	0.914273	-4.075996	2.099312	-0.590940	-4.601932	-0.006181	0.963604	-4.121644	2.055034	-0.593603
16	H	-4.032348	1.639221	1.256163	-4.656287	0.467590	-0.918972	-4.057971	1.645150	1.252314	-4.698124	0.413158	-0.874094
17	C	-4.812444	1.369939	-0.743020	-4.608212	0.980426	1.182000	-4.855173	1.314751	-0.730824	-4.630189	0.979088	1.212589
18	H	-4.868480	0.600158	-1.519443	-4.024266	1.592221	1.877448	-4.915301	0.522505	-1.484032	-4.041458	1.610395	1.886178
19	H	-4.321829	2.250013	-1.171560	-4.603104	-0.050655	1.550054	-4.371844	2.183696	-1.189253	-4.616719	-0.042355	1.606306
20	H	-5.835421	1.651564	-0.470130	-5.642370	1.341611	1.199347	-5.876726	1.600552	-0.456905	-5.665638	1.336513	1.231829
21	O	-1.865285	2.629718	0.688786	-1.806594	2.774337	0.007001	-1.900867	2.627472	0.632822	-1.848830	2.755324	-0.043664
22	H	-0.997205	3.126630	0.692491	-0.922440	3.240224	0.049662	-1.034991	3.127353	0.615788	-0.966548	3.225652	-0.021066
23	O	0.598216	3.197279	0.424649	0.694289	3.225938	-0.032904	0.559939	3.194705	0.334038	0.650843	3.214790	-0.117042
24	O	3.668562	-0.067428	-1.133431	3.767131	-0.303198	-0.816961	3.621043	-0.098648	-1.182384	3.726112	-0.319520	-0.869071
25	C	4.760804	0.861803	-1.267785	4.908652	0.567648	-0.931330	4.706708	0.831445	-1.358751	4.861666	0.553160	-1.023526
26	H	5.005089	1.299459	-0.296080	5.060035	1.114976	0.002887	4.968113	1.291276	-0.401822	5.029891	1.118892	-0.103241

27	H	4.503864	1.645700	-1.985385	4.770724	1.263860	-1.763067	4.432417	1.598287	-2.088208	4.704079	1.232712	-1.865452
28	H	5.598954	0.273389	-1.636594	5.755825	-0.087738	-1.124935	5.539836	0.238047	-1.730877	5.707238	-0.102890	-1.221741
29	C	-3.125767	-3.061250	-0.759830	-3.156647	-2.999749	-0.948509	-3.149703	-3.110633	-0.626995	-3.183932	-3.049741	-0.814038
30	H	-2.476250	-3.579816	-0.047125	-2.618335	-3.468168	-0.118220	-2.723280	-3.183528	-1.632668	-2.649654	-3.234908	-1.751486
31	H	-2.690638	-3.105444	-1.763448	-2.611037	-3.157341	-1.884463	-4.130483	-3.585182	-0.611448	-4.178947	-3.490679	-0.867668
32	H	-4.108513	-3.531902	-0.767436	-4.152701	-3.434215	-1.028939	-2.491672	-3.604570	0.095290	-2.632885	-3.488978	0.023793
33	O	1.476792	-1.438177	-1.931928	1.622545	-1.695831	-1.696239	1.427886	-1.512805	-1.891291	1.574197	-1.748518	-1.665479
34	C	1.932594	-1.822471	0.414826	1.782543	-1.791097	0.717879	1.923868	-1.811344	0.459374	1.778390	-1.774267	0.746747
35	H	1.275935	-2.687297	0.319646	1.101552	-2.639577	0.650094	1.251306	-2.668319	0.419547	1.085223	-2.615240	0.721804
36	H	2.957557	-2.122415	0.180642	2.812664	-2.150653	0.649045	2.936560	-2.133098	0.203955	2.802372	-2.145923	0.659451
37	H	2.397051	-1.631510	-2.172053	2.554303	-1.948084	-1.796711	2.345786	-1.705861	-2.140525	2.505687	-1.997411	-1.776335
38	C	1.923354	-1.317682	1.841354	1.629936	-1.108361	2.059666	1.915049	-1.282229	1.877683	1.615450	-1.080146	2.082014
39	O	2.737675	-0.541970	2.308743	2.424054	-0.312517	2.528085	1.008199	-1.435608	2.675060	0.615293	-1.123263	2.774489
40	O	0.907778	-1.829231	2.565307	0.507850	-1.482135	2.706385	3.046402	-0.614130	2.183785	2.721625	-0.398361	2.444797
41	H	0.960265	-1.465270	3.467647	0.471110	-1.007663	3.556601	2.974086	-0.287588	3.098699	2.550025	0.036464	3.299449

		**Conformer 5**			**Conformer 6**			**Conformer 7**			**Conformer 8**		
Row	Symbol	X	Y	Z	X	Y	Z	X	Y	Z	X	Y	Z
1	C	-2.102016	1.204646	0.389307	-2.103781	1.288862	0.024785	-2.084274	1.215625	0.379282	-2.082522	1.298827	0.012707
2	C	-0.978791	-1.280097	-0.224904	-0.993862	-1.232117	-0.449617	-0.999406	-1.291462	-0.217798	-1.015978	-1.246293	-0.439942
3	C	-0.699982	1.098830	0.154825	-0.689211	1.144466	-0.075421	-0.684085	1.086873	0.146089	-0.670747	1.129527	-0.086059
4	C	-2.948351	0.089028	0.323649	-2.969984	0.195702	-0.116607	-2.947896	0.112790	0.318666	-2.967286	0.219534	-0.122238
5	C	-2.361980	-1.149090	-0.001680	-2.387917	-1.063270	-0.359420	-2.381109	-1.136344	0.001195	-2.407319	-1.051242	-0.354990
6	C	-0.154410	-0.165088	-0.141876	-0.151730	-0.137196	-0.302369	-0.159216	-0.188177	-0.142046	-0.156656	-0.163953	-0.302301
7	H	-0.548800	-2.238839	-0.479948	-0.567155	-2.205552	-0.648084	-0.583564	-2.259223	-0.461442	-0.605600	-2.229380	-0.624330
8	C	0.139829	2.295802	0.156690	0.177982	2.320048	-0.012137	0.174383	2.270982	0.139217	0.216706	2.290944	-0.035118
9	C	1.537020	2.173454	-0.209417	1.599934	2.156433	-0.241549	1.567793	2.125946	-0.237589	1.634484	2.102692	-0.278488
10	H	2.101600	3.095929	-0.263605	2.190856	3.063754	-0.256445	2.144158	3.039618	-0.313002	2.239534	2.999910	-0.318798
11	C	2.094200	0.967616	-0.475307	2.147937	0.932400	-0.432997	2.101070	0.909058	-0.498117	2.156230	0.866981	-0.462904
12	C	1.349815	-0.361248	-0.333005	1.358155	-0.374721	-0.344512	1.342486	-0.403413	-0.315872	1.348197	-0.421262	-0.325282
13	O	-3.220903	-2.200003	-0.079364	-3.262539	-2.094815	-0.498703	-3.256480	-2.173539	-0.072498	-3.299734	-2.067753	-0.489120
14	C	-4.434308	0.223976	0.566151	-4.467390	0.369432	-0.005613	-4.431896	0.272259	0.558381	-4.461691	0.419891	-0.015255
15	H	-4.801679	-0.688789	1.044453	-4.730126	1.377535	-0.338512	-4.813867	-0.631839	1.041668	-4.705774	1.431693	-0.351005
16	H	-4.602803	1.048462	1.264728	-4.959125	-0.335835	-0.681975	-4.588549	1.103415	1.251785	-4.963984	-0.278204	-0.691352
17	C	-5.241417	0.479657	-0.722036	-5.000615	0.154145	1.424602	-5.233130	0.532363	-0.732575	-5.002339	0.217039	1.413999
18	H	-5.117754	-0.343025	-1.433657	-4.550179	0.866751	2.123373	-5.120785	-0.296397	-1.438994	-4.540819	0.922610	2.112670
19	H	-4.917858	1.404103	-1.211498	-4.777445	-0.857146	1.779804	-4.894941	1.448853	-1.227065	-4.798671	-0.797492	1.771600
20	H	-6.308860	0.572938	-0.493590	-6.087117	0.291785	1.453739	-6.299364	0.643080	-0.506345	-6.086209	0.374571	1.439937
21	O	-2.650561	2.405561	0.680644	-2.644863	2.506722	0.253432	-2.614491	2.426331	0.663009	-2.602719	2.527179	0.232031
22	H	-1.895552	3.062502	0.669385	-1.875754	3.145516	0.296553	-1.849974	3.071703	0.649579	-1.823281	3.153208	0.272043
23	O	-0.337999	3.429374	0.438535	-0.294293	3.470010	0.205064	-0.284596	3.412963	0.416498	-0.234721	3.449815	0.176737
24	O	3.368144	0.762173	-0.830749	3.444554	0.689094	-0.659438	3.361229	0.671922	-0.883659	3.439891	0.589133	-0.723375
25	C	4.250927	1.889966	-0.978722	4.366882	1.791936	-0.735873	4.265500	1.778674	-1.063134	4.386236	1.668419	-0.842245
26	H	4.351151	2.421982	-0.028503	4.385200	2.338897	0.210996	4.399449	2.315325	-0.119873	4.445173	2.226020	0.096457
27	H	3.876740	2.564066	-1.754120	4.090271	2.460448	-1.555818	3.886595	2.453671	-1.835269	4.099840	2.332083	-1.662604
28	H	5.209794	1.469954	-1.277350	5.339588	1.343267	-0.929683	5.207329	1.335012	-1.380830	5.341657	1.193923	-1.058694
29	C	-2.714528	-3.505132	-0.383965	-2.764772	-3.411652	-0.764428	-2.770956	-3.488103	-0.370809	-2.825315	-3.394894	-0.746658
30	H	-2.243946	-3.524166	-1.372273	-2.210614	-3.440534	-1.708132	-2.299232	-3.518696	-1.358281	-2.268664	-3.438418	-1.688326
31	H	-3.583989	-4.161961	-0.381054	-3.647474	-4.046164	-0.839569	-3.651066	-4.130558	-0.366698	-3.719292	-4.013399	-0.821741
32	H	-2.000473	-3.838114	0.376225	-2.128166	-3.764407	0.053450	-2.063772	-3.829610	0.391972	-2.198174	-3.755195	0.075203
33	O	1.535105	-1.156448	-1.500234	1.632711	-1.192566	-1.478136	1.518963	-1.236503	-1.458500	1.610646	-1.286895	-1.426192
34	C	1.908736	-1.095585	0.934645	1.773267	-1.104226	0.979859	1.871523	-1.106824	0.986352	1.729974	-1.105276	1.037477
35	H	1.825402	-0.449529	1.810082	1.625230	-0.444088	1.836057	1.654106	-0.481056	1.852938	1.446442	-0.453765	1.865084
36	H	1.284923	-1.978345	1.109682	1.111197	-1.967385	1.105308	1.317576	-2.044954	1.089364	1.143862	-2.026828	1.106307
37	H	2.403692	-1.596559	-1.427374	2.478894	-1.652328	-1.316817	2.435971	-1.556109	-1.469779	2.515007	-1.630527	-1.341351
38	C	3.327081	-1.602469	0.786495	3.184185	-1.652013	0.975373	3.349751	-1.435680	1.017936	3.188989	-1.468870	1.219417
39	O	3.738575	-2.225433	-0.182608	3.671561	-2.295133	0.055954	4.136951	-1.043858	1.856329	3.902980	-1.072921	2.119173

40	O	4.088608	-1.348388	1.859497	3.842402	-1.410141	2.117399	3.709570	-2.275750	0.017491	3.619942	-2.344787	0.279682
41	H	4.969720	-1.740896	1.717545	4.721169	-1.829244	2.066724	4.667522	-2.442158	0.075825	4.562905	-2.533359	0.434693
		**Conformer 9**			**Conformer 10**			**Conformer 11**			**Conformer 12**		
Row	Symbol	X	Y	Z	X	Y	Z	X	Y	Z	X	Y	Z
1	C	1.676395	-1.456718	0.443401	1.594049	-1.631751	0.051497	1.547420	-1.632782	0.045540	-1.634204	-1.458179	-0.436711
2	C	0.782222	1.027292	-0.473263	0.796125	0.949639	-0.662493	0.775240	0.956556	-0.680084	-0.765569	1.031262	0.498543
3	C	0.284935	-1.239142	0.224203	0.207567	-1.305373	-0.000011	0.165284	-1.288786	0.012909	-0.243456	-1.222859	-0.236460
4	C	2.626399	-0.451263	0.215888	2.586690	-0.690018	-0.253797	2.546908	-0.707875	-0.290001	-2.595533	-0.470728	-0.176748
5	C	2.153249	0.785240	-0.260506	2.159849	0.600598	-0.616802	2.133725	0.585317	-0.661413	-2.136245	0.767055	0.311091
6	C	-0.148783	0.026580	-0.220995	-0.174953	0.007543	-0.344091	-0.197377	0.028608	-0.334752	0.170989	0.046883	0.215194
7	H	0.455355	1.986719	-0.846795	0.504337	1.947465	-0.955798	0.479758	1.955747	-0.967014	-0.433916	1.989812	0.871775
8	C	-0.661150	-2.344146	0.365791	-0.798451	-2.345819	0.203962	-0.854159	-2.316123	0.225435	0.717501	-2.314912	-0.390494
9	C	-2.040816	-2.142550	-0.030040	-2.193855	-2.028021	-0.025822	-2.247112	-1.984310	-0.019031	2.094909	-2.101293	0.018340
10	H	-2.680970	-3.014866	0.007551	-2.890333	-2.853642	0.048441	-2.955596	-2.801968	0.030242	2.745036	-2.967285	0.001390
11	C	-2.496585	-0.929250	-0.427787	-2.596855	-0.767802	-0.321804	-2.617926	-0.715991	-0.311035	2.520487	-0.879070	0.413574
12	C	-1.645565	0.339211	-0.371893	-1.651047	0.433086	-0.310477	-1.654313	0.468081	-0.267484	1.653684	0.375653	0.333206
13	O	3.107900	1.723817	-0.496713	3.151340	1.480208	-0.919272	3.132150	1.444679	-0.995829	-3.102398	1.684196	0.580104
14	C	4.099388	-0.704588	0.442326	4.052070	-1.055776	-0.195159	4.007851	-1.094146	-0.254503	-4.067876	-0.745234	-0.382447
15	H	4.571020	0.220705	0.786261	4.157857	-2.120167	-0.423447	4.093875	-2.163624	-0.466609	-4.560963	0.178164	-0.700211
16	H	4.210247	-1.443283	1.241216	4.589063	-0.500732	-0.969914	4.536700	-0.560090	-1.049373	-4.180284	-1.470279	-1.193523
17	C	4.832693	-1.207751	-0.816799	4.697519	-0.765788	1.174273	4.685103	-0.790171	1.096435	-4.770132	-1.285092	0.879202
18	H	4.764777	-0.477787	-1.629861	4.202295	-1.332015	1.969977	4.198485	-1.335628	1.911689	-4.699679	-0.570158	1.705288
19	H	4.404481	-2.151321	-1.170592	4.631583	0.298396	1.423073	4.638861	0.278829	1.328173	-4.320369	-2.228121	1.206647
20	H	5.893354	-1.377013	-0.600734	5.756284	-1.047318	1.166792	5.739460	-1.086872	1.072187	-5.831565	-1.467756	0.678446
21	O	2.113439	-2.660863	0.874794	1.983006	-2.881604	0.388212	1.925390	-2.884346	0.388925	-2.060420	-2.662884	-0.877554
22	H	1.297853	-3.235411	0.958175	1.139930	-3.401815	0.533885	1.079487	-3.392823	0.551384	-1.239680	-3.225282	-0.979530
23	O	-0.288985	-3.475718	0.784116	-0.469529	-3.519777	0.531895	-0.539417	-3.490629	0.561056	0.359369	-3.446141	-0.818504
24	O	-3.746976	-0.660920	-0.815137	-3.857783	-0.393272	-0.556231	-3.858689	-0.296376	-0.589670	3.750005	-0.567914	0.843748
25	C	-4.714290	-1.726046	-0.844863	-4.899658	-1.385486	-0.523612	-4.935063	-1.253656	-0.629396	4.745304	-1.604839	0.947799
26	H	-4.858606	-2.139314	0.157673	-4.966223	-1.836739	0.470632	-5.062642	-1.717863	0.352280	4.948932	-2.032000	-0.037912
27	H	-4.395448	-2.511344	-1.535933	-4.715631	-2.155709	-1.277843	-4.734608	-2.014655	-1.388275	4.408776	-2.382385	1.638724
28	H	-5.636981	-1.268832	-1.198136	-5.818047	-0.848047	-0.753600	-5.822516	-0.681747	-0.893871	5.635602	-1.114367	1.336906
29	C	2.724717	3.023493	-0.962149	2.817494	2.813074	-1.325133	2.808624	2.775218	-1.417880	-2.731703	2.982473	1.059910
30	H	2.070812	3.523921	-0.240742	2.218084	2.807767	-2.241286	2.187757	2.762165	-2.319541	-2.213291	2.913304	2.021673
31	H	2.229406	2.962836	-1.936629	3.770654	3.305596	-1.515688	3.764223	3.249811	-1.639237	-3.669214	3.522693	1.188886
32	H	3.654991	3.582375	-1.060507	2.282767	3.344658	-0.531479	2.300517	3.328512	-0.621701	-2.103370	3.506612	0.332790
33	O	-1.902164	1.079332	-1.558316	-1.979442	1.247591	-1.428522	-1.892013	1.337639	-1.367225	1.826678	1.167862	1.501510
34	C	-2.156139	1.110583	0.896953	-1.959638	1.174968	1.038416	-1.961897	1.183288	1.104969	2.164351	1.133636	-0.952983
35	H	-3.250709	1.158088	0.848490	-3.046102	1.304029	1.114814	-3.021690	1.455810	1.105339	3.233102	1.326587	-0.819424
36	H	-1.882604	0.567491	1.802688	-1.632686	0.569065	1.884547	-1.783045	0.474418	1.914994	2.032284	0.481398	-1.817760
37	H	-1.831438	2.028367	-1.342353	-1.813918	2.177333	-1.182454	-2.834032	1.566750	-1.379184	2.775785	1.317078	1.632418
38	C	-1.680032	2.540808	0.988950	-1.367006	2.560547	1.136140	-1.143734	2.419784	1.404246	1.472659	2.440917	-1.269955
39	O	-1.601599	3.304955	0.034946	-1.334668	3.364178	0.212360	-0.221351	2.463891	2.198327	0.656730	2.593778	-2.161151
40	O	-1.392760	2.916656	2.239813	-0.916574	2.851031	2.361358	-1.565012	3.503357	0.722014	1.881743	3.454405	-0.480851
41	H	-1.146202	3.860094	2.236911	-0.600099	3.773317	2.370418	-1.004883	4.259437	0.974011	1.408156	4.261555	-0.751362

		**Conformer 13**			**Conformer 14**		
Row	Symbol	X	Y	Z	X	Y	Z
1	C	1.795236	-1.264358	0.331256	1.695178	-1.327338	0.142441
2	C	0.788977	1.214968	-0.470440	0.841445	1.291028	-0.337354
3	C	0.403495	-1.116705	0.064158	0.314682	-1.062893	-0.091465
4	C	2.689366	-0.190965	0.212062	2.652739	-0.303499	0.146129
5	C	2.160939	1.045387	-0.203861	2.200968	1.005127	-0.110452
6	C	-0.086672	0.144365	-0.326874	-0.095919	0.264653	-0.319598
7	H	0.414482	2.173599	-0.802125	0.519014	2.305055	-0.529833
8	C	-0.491905	-2.264776	0.164535	-0.645932	-2.160994	-0.166855
9	C	-1.890908	-2.091804	-0.181237	-2.017438	-1.861145	-0.525940

10	H	-2.532303	-2.964426	-0.118423	-2.701112	-2.697308	-0.626816
11	C	-2.407558	-0.898684	-0.545168	-2.464951	-0.598667	-0.721774
12	C	-1.569593	0.377283	-0.639528	-1.569719	0.627917	-0.529042
13	O	3.062195	2.055456	-0.325344	3.163318	1.964188	-0.117274
14	C	4.163066	-0.369240	0.496458	4.113953	-0.604325	0.388584
15	H	4.558986	0.559271	0.918133	4.565408	0.234713	0.926162
16	H	4.282970	-1.148722	1.254222	4.189168	-1.483760	1.034232
17	C	4.983332	-0.747536	-0.752689	4.906865	-0.859159	-0.908599
18	H	4.906660	0.026610	-1.523022	4.874189	0.014365	-1.567640
19	H	4.632179	-1.691289	-1.182689	4.499322	-1.714605	-1.457200
20	H	6.041521	-0.865746	-0.494837	5.956815	-1.072448	-0.679944
21	O	2.287639	-2.464370	0.711360	2.112352	-2.593625	0.363576
22	H	1.506515	-3.088484	0.735949	1.295138	-3.167708	0.307314
23	O	-0.070120	-3.399331	0.519034	-0.294666	-3.355127	0.038813
24	O	-3.750957	-0.828345	-0.711995	-3.787245	-0.450760	-0.947647
25	C	-4.363828	-0.162099	-1.841525	-4.358402	0.562803	-1.808237
26	H	-3.962268	-0.562267	-2.775501	-3.929828	0.493873	-2.809850
27	H	-4.218378	0.917831	-1.812684	-4.213411	1.568234	-1.413920
28	H	-5.423915	-0.400370	-1.756811	-5.420745	0.321504	-1.836604
29	C	2.617277	3.357513	-0.724697	2.799519	3.330588	-0.349410
30	H	1.897001	3.764966	-0.007917	2.350226	3.455178	-1.339951
31	H	2.175724	3.332810	-1.726262	3.732107	3.891803	-0.298630
32	H	3.512177	3.979081	-0.736220	2.111599	3.691522	0.421854
33	O	-1.693682	0.930910	-1.967073	-1.697782	1.518860	-1.646967
34	C	-2.174900	1.483509	0.258936	-2.097913	1.480742	0.672932
35	H	-1.655935	2.422460	0.048553	-1.543491	2.419275	0.662785
36	H	-3.220512	1.650759	-0.015185	-3.157035	1.693854	0.515733
37	H	-1.181226	0.382162	-2.580032	-1.208567	1.148734	-2.397488
38	C	-2.132125	1.242092	1.750216	-1.920826	0.857999	2.042556
39	O	-1.763636	0.231197	2.314852	-1.030035	1.138719	2.822625
40	O	-2.581812	2.327439	2.416048	-2.882761	-0.041058	2.328443
41	H	-2.555877	2.134423	3.370170	-2.709744	-0.408884	3.213741

Table S8: Energies and Boltzmann distribution of compound **18** (4*S*).

Conformer	Hartrees	kcal/mol	ΔG (kcal/mol)	Boltzmann Distribution (%)
Conformer 6	-1146.936642	-719713.763	0	35.0242093
Conformer 5	-1146.936319	-719713.56	0.2026856	24.1102081
Conformer 8	-1146.935388	-719712.976	0.78689705	8.21821188
Conformer 7	-1146.935278	-719712.907	0.85592311	7.23685802
Conformer 1	-1146.935276	-719712.905	0.85717812	7.22014501
Conformer 3	-1146.935143	-719712.822	0.9406369	6.19114701
Conformer 4	-1146.93477	-719712.588	1.17469799	4.02254051
Conformer 2	-1146.934706	-719712.548	1.2148586	3.73566729
Conformer 9	-1146.933906	-719712.046	1.71686629	1.48154632
Conformer 10	-1146.933703	-719711.918	1.84425074	1.17164434
Conformer 11	-1146.933457	-719711.764	1.9986181	0.88163234
Conformer 12	-1146.933265	-719711.644	2.11909995	0.70613999

Conformer 14	-1146.924129	-719705.911	7.85202773	1.8282E-05
Conformer 13	-1146.924084	-719705.882	7.88026566	1.7355E-05
Conformer 15	-1146.923355	-719705.425	8.33772016	7.4716E-06
Conformer 16	-1146.923264	-719705.368	8.39482354	6.7255E-06

Table S9: XYZ coordinates of compound 18 (4*S*).

		Conformer 1			Conformer 2			Conformer 3			Conformer 4		
Row	Symbol	X	Y	Z	X	Y	Z	X	Y	Z	X	Y	Z
1	C	1.57152	1.36981	0.29671	1.52940	1.46810	-0.20324	1.60535	1.35725	0.27699	1.56818	1.44493	-0.22075
2	C	0.97904	-1.23652	-0.52517	0.96887	-1.23640	-0.64128	1.00886	-1.27088	-0.46858	1.00043	-1.26834	-0.58936
3	C	0.22431	1.00929	0.00237	0.17557	1.03058	-0.29005	0.25942	0.99313	-0.01872	0.21353	1.01066	-0.31108
4	C	2.62354	0.44970	0.18663	2.60377	0.57809	-0.34185	2.65431	0.43003	0.20520	2.64002	0.54703	-0.32188
5	C	2.30311	-0.85258	-0.24232	2.29724	-0.77771	-0.56676	2.33203	-0.88372	-0.18558	2.32991	-0.81331	-0.51143
6	C	-0.05087	-0.31317	-0.39810	-0.08158	-0.33890	-0.49803	-0.01790	-0.33994	-0.38058	-0.04743	-0.36296	-0.48430
7	H	0.75627	-2.24282	-0.85189	0.75720	-2.28217	-0.81602	0.78453	-2.28602	-0.76554	0.78622	-2.31764	-0.73770
8	C	-0.83710	2.01534	0.05516	-0.91860	2.00041	-0.22873	-0.79830	2.00356	-0.00363	-0.87676	1.98569	-0.28504
9	C	-2.17758	1.64845	-0.36229	-2.27959	1.54175	-0.43675	-2.13669	1.62957	-0.42413	-2.23782	1.52707	-0.49730
10	H	-2.91381	2.44251	-0.37511	-3.04874	2.30380	-0.45576	-2.86930	2.42594	-0.46766	-3.00330	2.29161	-0.54490
11	C	-2.47587	0.37665	-0.71134	-2.55601	0.22767	-0.59517	-2.43440	0.35125	-0.74979	-2.51722	0.21121	-0.63579
12	C	-1.47920	-0.77341	-0.66265	-1.50777	-0.87460	-0.52801	-1.44500	-0.80201	-0.64783	-1.47532	-0.89357	-0.52057
13	O	3.35502	-1.70560	-0.35674	3.36765	-1.60403	-0.70318	3.38107	-1.74420	-0.26353	3.39803	-1.64767	-0.61093
14	C	4.04693	0.85643	0.49251	4.03236	1.06253	-0.24559	4.07650	0.84018	0.51213	4.06970	1.02763	-0.22248
15	H	4.03235	1.63922	1.25616	4.65629	0.46760	-0.91897	4.05803	1.64496	1.25249	4.69806	0.41339	-0.87412
16	H	4.57967	-0.00087	0.91427	4.07600	2.09931	-0.59093	4.60190	-0.00638	0.96367	4.12162	2.05520	-0.59318
17	C	4.81244	1.36994	-0.74302	4.60821	0.98042	1.18201	4.85531	1.31469	-0.73065	4.63020	0.97877	1.21272
18	H	4.32183	2.25001	-1.17156	4.60310	-0.05067	1.55005	4.37205	2.18371	-1.18901	4.61672	-0.04278	1.60616
19	H	4.86848	0.60016	-1.51944	4.02427	1.59221	1.87746	4.91542	0.52252	-1.48393	4.04151	1.60991	1.88650
20	H	5.83542	1.65156	-0.47013	5.64237	1.34160	1.19936	5.87687	1.60041	-0.45668	5.66565	1.33616	1.23200
21	O	1.86529	2.62972	0.68879	1.80660	2.77434	0.00700	1.90099	2.62745	0.63306	1.84885	2.75542	-0.04322
22	H	0.99721	3.12663	0.69249	0.92244	3.24023	0.04967	1.03506	3.12735	0.61617	0.96667	3.22589	-0.02052
23	O	-0.59822	3.19728	0.42465	-0.69429	3.22594	-0.03290	-0.55984	3.19476	0.33471	-0.65082	3.21492	-0.11668
24	O	-3.66856	-0.06743	-1.13343	-3.76713	-0.30320	-0.81696	-3.62114	-0.09822	-1.18216	-3.72624	-0.31928	-0.86872
25	C	-4.76080	0.86180	-1.26779	-4.90865	0.56765	-0.93133	-4.70679	0.83199	-1.35832	-4.86181	0.55338	-1.02290
26	H	-5.59895	0.27339	-1.63659	-5.75582	-0.08773	-1.12494	-4.43238	1.59910	-2.08744	-5.70744	-0.10265	-1.22091
27	H	-4.50386	1.64570	-1.98539	-4.77072	1.26387	-1.76305	-4.96830	1.29144	-0.40124	-4.70445	1.23294	-1.86486
28	H	-5.00509	1.29946	-0.29608	-5.06004	1.11497	0.00290	-5.53987	0.23874	-1.73079	-5.02985	1.11913	-0.10258
29	C	3.12577	-3.06125	-0.75983	3.15665	-2.99975	-0.94851	3.14959	-3.11068	-0.62696	3.18360	-3.04955	-0.81463
30	H	4.10851	-3.53190	-0.76744	4.15270	-3.43422	-1.02893	2.49143	-3.60441	0.09534	4.17855	-3.49061	-0.86844
31	H	2.69064	-3.10544	-1.76345	2.61104	-3.15734	-1.88446	4.13033	-3.58529	-0.61120	2.64923	-3.23443	-1.75209
32	H	2.47625	-3.57982	-0.04713	2.61833	-3.46816	-0.11822	2.72330	-3.18377	-1.63268	2.63255	-3.48893	0.02313
33	O	-1.47679	-1.43818	-1.93193	-1.62254	-1.69582	-1.69625	-1.42787	-1.51196	-1.89186	-1.57428	-1.74796	-1.66593
34	H	-2.39705	-1.63151	-2.17205	-2.55430	-1.94807	-1.79673	-2.34576	-1.70505	-2.14111	-2.50568	-1.99726	-1.77656
35	C	-1.93259	-1.82247	0.41483	-1.78254	-1.79110	0.71787	-1.92389	-1.81155	0.45868	-1.77837	-1.77448	0.74630
36	H	-2.95756	-2.12242	0.18064	-2.81266	-2.15067	0.64903	-2.93656	-2.13320	0.20309	-2.80243	-2.14594	0.65909
37	H	-1.27594	-2.68730	0.31965	-1.10154	-2.63958	0.65008	-1.25132	-2.66851	0.41858	-1.08536	-2.61557	0.72089
38	C	-1.92335	-1.31768	1.84135	-1.62994	-1.10837	2.05966	-1.91505	-1.28297	1.87720	-1.61511	-1.08093	2.08182
39	O	-2.73768	-0.54197	2.30874	-2.42407	-0.31254	2.52808	-1.00802	-1.43641	2.67436	-0.61511	-1.12499	2.77447
40	O	-0.90778	-1.82923	2.56531	-0.50786	-1.48214	2.70638	-3.04659	-0.61538	2.18376	-2.72086	-0.39844	2.44461
41	H	-0.96027	-1.46527	3.46765	-0.47112	-1.00767	3.55660	-2.97423	-0.28918	3.09880	-2.54915	0.03600	3.29943
		**Conformer 5**			**Conformer 6**			**Conformer 7**			**Conformer 8**		
Row	Symbol	X	Y	Z	X	Y	Z	X	Y	Z	X	Y	Z
1	C	2.10202	1.20465	0.38931	2.10378	1.28886	0.02479	2.08427	1.21563	0.37928	2.08251	1.29888	0.01276
2	C	0.97879	-1.28010	-0.22490	0.99386	-1.23212	-0.44962	0.99941	-1.29146	-0.21780	1.01597	-1.24625	-0.43998

3	C	0.69998	1.09883	0.15483	0.68921	1.14447	-0.07542	0.68409	1.08687	0.14609	0.67073	1.12954	-0.08598
4	C	2.94835	0.08903	0.32365	2.96998	0.19570	-0.11661	2.94790	0.11279	0.31867	2.96726	0.21958	-0.12224
5	C	2.36198	-1.14909	-0.00168	2.38792	-1.06327	-0.35942	2.38111	-1.13634	0.00120	2.40732	-1.05119	-0.35504
6	C	0.15441	-0.16509	-0.14188	0.15173	-0.13720	-0.30237	0.15922	-0.18818	-0.14205	0.15663	-0.16393	-0.30229
7	H	0.54880	-2.23884	-0.47995	0.56716	-2.20555	-0.64808	0.58356	-2.25922	-0.46144	0.60557	-2.22932	-0.62438
8	C	-0.13983	2.29580	0.15669	-0.17798	2.32005	-0.01214	-0.17438	2.27098	0.13922	-0.21672	2.29096	-0.03500
9	C	-1.53702	2.17345	-0.20942	-1.59993	2.15643	-0.24155	-1.56779	2.12595	-0.23759	-1.63452	2.10272	-0.27828
10	H	-2.10160	3.09593	-0.26361	-2.19086	3.06375	-0.25645	-2.14416	3.03962	-0.31300	-2.23954	2.99995	-0.31855
11	C	-2.09420	0.96762	-0.47531	-2.14794	0.93240	-0.43300	-2.10107	0.90906	-0.49812	-2.15628	0.86702	-0.46276
12	C	-1.34982	-0.36125	-0.33301	-1.35816	-0.37472	-0.34451	-1.34249	-0.40341	-0.31587	-1.34821	-0.42122	-0.32521
13	O	3.22090	-2.20000	-0.07936	3.26254	-2.09482	-0.49870	3.25648	-2.17354	-0.07250	3.29976	-2.06767	-0.48913
14	C	4.43431	0.22398	0.56615	4.46739	0.36943	-0.00561	4.43190	0.27226	0.55838	4.46167	0.41992	-0.01525
15	H	4.60280	1.04846	1.26473	4.95913	-0.33584	-0.68198	4.58855	1.10342	1.25179	4.96396	-0.27803	-0.69151
16	H	4.80168	-0.68879	1.04445	4.73013	1.37754	-0.33851	4.81387	-0.63184	1.04167	4.70575	1.43179	-0.35079
17	C	5.24142	0.47966	-0.72204	5.00062	0.15415	1.42460	5.23313	0.53236	-0.73258	5.00233	0.21677	1.41396
18	H	4.91786	1.40410	-1.21150	4.77745	-0.85715	1.77980	4.89494	1.44885	-1.22707	4.79865	-0.79783	1.77135
19	H	5.11775	-0.34303	-1.43366	4.55018	0.86675	2.12337	5.12079	-0.29640	-1.43899	4.54081	0.92221	2.11277
20	H	6.30886	0.57294	-0.49359	6.08712	0.29179	1.45374	6.29936	0.64308	-0.50635	6.08620	0.37429	1.43994
21	O	2.65056	2.40556	0.68064	2.64486	2.50672	0.25343	2.61449	2.42633	0.66301	2.60267	2.52721	0.23208
22	H	1.89555	3.06250	0.66939	1.87575	3.14552	0.29655	1.84997	3.07170	0.64958	1.82314	3.15321	0.27206
23	O	0.33800	3.42937	0.43854	0.29429	3.47001	0.20506	0.28460	3.41296	0.41650	0.23472	3.44982	0.17694
24	O	-3.36814	0.76217	-0.83075	-3.44455	0.68909	-0.65944	-3.36123	0.67192	-0.88366	-3.43986	0.58911	-0.72354
25	C	-4.25093	1.88997	-0.97872	-4.36688	1.79194	-0.73587	-4.26550	1.77867	-1.06313	-4.38626	1.66835	-0.84278
26	H	-3.87674	2.56407	-1.75412	-5.33959	1.34327	-0.92968	-3.88660	2.45367	-1.83527	-4.09950	2.33208	-1.66295
27	H	-4.35115	2.42198	-0.02850	-4.09027	2.46045	-1.55582	-4.39945	2.31533	-0.11987	-4.44568	2.22584	0.09595
28	H	-5.20979	1.46995	-1.27735	-4.38520	2.33890	0.21100	-5.20733	1.33501	-1.38083	-5.34152	1.19377	-1.05974
29	C	2.71453	-3.50513	-0.38397	2.76477	-3.41165	-0.76443	2.77096	-3.48810	-0.37081	2.82545	-3.39478	-0.74705
30	H	2.24395	-3.52417	-1.37227	3.64747	-4.04616	-0.83957	2.06377	-3.82961	0.39197	2.19833	-3.75538	0.07470
31	H	2.00047	-3.83811	0.37623	2.21061	-3.44053	-1.70813	3.65107	-4.13056	-0.36670	3.71949	-4.01317	-0.82229
32	H	3.58399	-4.16196	-0.38105	2.12817	-3.76441	0.05345	2.29923	-3.51870	-1.35828	2.26882	-3.43807	-1.68874
33	O	-1.53511	-1.15645	-1.50023	-1.63271	-1.19257	-1.47814	-1.51896	-1.23650	-1.45850	-1.61077	-1.28686	-1.42610
34	H	-2.40369	-1.59656	-1.42737	-2.47889	-1.65233	-1.31682	-2.43597	-1.55611	-1.46978	-2.51535	-1.62995	-1.34152
35	C	-1.90874	-1.09559	0.93465	-1.77327	-1.10423	0.97986	-1.87152	-1.10682	0.98635	-1.72997	-1.10523	1.03754
36	H	-1.28492	-1.97835	1.10968	-1.11120	-1.96739	1.10531	-1.31758	-2.04495	1.08936	-1.14377	-2.02673	1.10641
37	H	-1.82540	-0.44953	1.81008	-1.62523	-0.44409	1.83606	-1.65411	-0.48106	1.85294	-1.44654	-0.45368	1.86515
38	C	-3.32708	-1.60247	0.78650	-3.18419	-1.65201	0.97537	-3.34975	-1.43568	1.01794	-3.18894	-1.46896	1.21952
39	O	-3.73858	-2.22543	-0.18261	-3.67156	-2.29513	0.05595	-4.13695	-1.04386	1.85633	-3.90275	-1.07335	2.11960
40	O	-4.08861	-1.34839	1.85950	-3.84240	-1.41014	2.11740	-3.70957	-2.27575	0.01749	-3.62000	-2.34460	0.27962
41	H	-4.96972	-1.74090	1.71755	-4.72117	-1.82924	2.06672	-4.66752	-2.44216	0.07583	-4.56289	-2.53332	0.43482
		**Conformer 9**			**Conformer 10**			**Conformer 11**			**Conformer 12**		
Row	Symbol	X	Y	Z	X	Y	Z	X	Y	Z	X	Y	Z
1	C	-1.67641	-1.45671	0.44341	-1.59405	-1.63175	0.05150	-1.54742	-1.63278	0.04554	1.63420	-1.45818	-0.43671
2	C	-0.78222	1.02729	-0.47325	-0.79613	0.94964	-0.66249	-0.77524	0.95656	-0.68008	0.76557	1.03126	0.49854
3	C	-0.28495	-1.23914	0.22420	-0.20757	-1.30537	-0.00001	-0.16528	-1.28879	0.01291	0.24346	-1.22286	-0.23646
4	C	-2.62641	-0.45125	0.21590	-2.58669	-0.69002	-0.25380	-2.54691	-0.70788	-0.29000	2.59553	-0.47073	-0.17675
5	C	-2.15325	0.78525	-0.26050	-2.15985	0.60060	-0.61680	-2.13373	0.58532	-0.66141	2.13625	0.76706	0.31109
6	C	0.14878	0.02658	-0.22099	0.17495	0.00754	-0.34409	0.19738	0.02861	-0.33475	-0.17099	0.04688	0.21519
7	H	-0.45535	1.98672	-0.84678	-0.50434	1.94746	-0.95580	-0.47976	1.95575	-0.96701	0.43392	1.98981	0.87178
8	C	0.66113	-2.34415	0.36579	0.79845	-2.34582	0.20396	0.85416	-2.31612	0.22544	-0.71750	-2.31491	-0.39049
9	C	2.04080	-2.14256	-0.03004	2.19386	-2.02802	-0.02582	2.24711	-1.98431	-0.01903	-2.09491	-2.10129	0.01834
10	H	2.68095	-3.01488	0.00755	2.89033	-2.85364	0.04844	2.95560	-2.80197	0.03024	-2.74504	-2.96728	0.00139
11	C	2.49658	-0.92927	-0.42779	2.59686	-0.76780	-0.32180	2.61793	-0.71599	-0.31104	-2.52049	-0.87907	0.41357
12	C	1.64556	0.33920	-0.37189	1.65105	0.43309	-0.31048	1.65431	0.46808	-0.26748	-1.65368	0.37565	0.33321
13	O	-3.10789	1.72383	-0.49669	-3.15134	1.48021	-0.91927	-3.13215	1.44468	-0.99583	3.10240	1.68420	0.58010
14	C	-4.09940	-0.70457	0.44234	-4.05207	-1.05578	-0.19516	-4.00785	-1.09415	-0.25450	4.06788	-0.74524	-0.38245
15	H	-4.21026	-1.44328	1.24122	-4.58906	-0.50073	-0.96991	-4.53670	-0.56009	-1.04937	4.18028	-1.47028	-1.19352

16	H	-4.57102	0.22072	0.78629	-4.15786	-2.12017	-0.42345	-4.09388	-2.16362	-0.46661	4.56096	0.17816	-0.70021
17	C	-4.83271	-1.20771	-0.81679	-4.69752	-0.76579	1.17427	-4.68510	-0.79017	1.09644	4.77013	-1.28509	0.87920
18	H	-4.40451	-2.15128	-1.17060	-4.63158	0.29840	1.42307	-4.63886	0.27883	1.32817	4.32037	-2.22812	1.20665
19	H	-4.76479	-0.47774	-1.62984	-4.20230	-1.33202	1.96998	-4.19849	-1.33563	1.91169	4.69968	-0.57016	1.70529
20	H	-5.89337	-1.37697	-0.60072	-5.75628	-1.04732	1.16679	-5.73946	-1.08687	1.07219	5.83157	-1.46776	0.67845
21	O	-2.11345	-2.66086	0.87480	-1.98301	-2.88160	0.38821	-1.92539	-2.88435	0.38893	2.06042	-2.66288	-0.87755
22	H	-1.29787	-3.23541	0.95819	-1.13993	-3.40182	0.53389	-1.07949	-3.39282	0.55138	1.23968	-3.22528	-0.97953
23	O	0.28896	-3.47573	0.78410	0.46953	-3.51978	0.53190	0.53942	-3.49063	0.56106	-0.35937	-3.44614	-0.81850
24	O	3.74697	-0.66094	-0.81514	3.85778	-0.39327	-0.55623	3.85869	-0.29638	-0.58967	-3.75001	-0.56791	0.84375
25	C	4.71428	-1.72607	-0.84486	4.89966	-1.38549	-0.52361	4.93506	-1.25366	-0.62940	-4.74530	-1.60484	0.94780
26	H	4.39544	-2.51136	-1.53594	4.71563	-2.15571	-1.27784	5.82252	-0.68175	-0.89387	-4.40878	-2.38238	1.63872
27	H	4.85859	-2.13934	0.15767	4.96622	-1.83674	0.47063	4.73461	-2.01466	-1.38828	-4.94893	-2.03200	-0.03791
28	H	5.63697	-1.26886	-1.19813	5.81805	-0.84805	-0.75360	5.06264	-1.71786	0.35228	-5.63560	-1.11437	1.33691
29	C	-2.72470	3.02350	-0.96216	-2.81749	2.81307	-1.32513	-2.80862	2.77522	-1.41788	2.73170	2.98247	1.05991
30	H	-2.07080	3.52394	-0.24076	-2.28277	3.34466	-0.53148	-3.76422	3.24981	-1.63924	2.10337	3.50661	0.33279
31	H	-3.65497	3.58238	-1.06053	-3.77065	3.30560	-1.51569	-2.18776	2.76217	-2.31954	3.66922	3.52269	1.18889
32	H	-2.22939	2.96281	-1.93664	-2.21808	2.80777	-2.24129	-2.30052	3.32851	-0.62170	2.21329	2.91330	2.02167
33	O	1.90217	1.07932	-1.55832	1.97944	1.24759	-1.42852	1.89201	1.33764	-1.36723	-1.82668	1.16786	1.50151
34	H	1.83141	2.02835	-1.34237	1.81392	2.17733	-1.18245	2.83403	1.56675	-1.37918	-2.77579	1.31708	1.63242
35	C	2.15614	1.11058	0.89695	1.95964	1.17497	1.03842	1.96190	1.18329	1.10497	-2.16435	1.13364	-0.95298
36	H	1.88260	0.56750	1.80269	1.63269	0.56907	1.88455	1.78305	0.47442	1.91499	-2.03229	0.48140	-1.81776
37	H	3.25071	1.15806	0.84849	3.04610	1.30403	1.11481	3.02169	1.45581	1.10534	-3.23310	1.32659	-0.81942
38	C	1.68006	2.54081	0.98894	1.36701	2.56055	1.13614	1.14373	2.41978	1.40425	-1.47266	2.44092	-1.26996
39	O	1.60158	3.30494	0.03492	1.33467	3.36418	0.21236	0.22135	2.46389	2.19833	-0.65673	2.59378	-2.16115
40	O	1.39287	2.91669	2.23981	0.91657	2.85103	2.36136	1.56501	3.50336	0.72201	-1.88174	3.45441	-0.48085
41	H	1.14633	3.86013	2.23691	0.60010	3.77332	2.37042	1.00488	4.25944	0.97401	-1.40815	4.26156	-0.75136
		**Conformer 13**			**Conformer 14**			**Conformer 15**			**Conformer 16**		
Row	Symbol	X	Y	Z	X	Y	Z	X	Y	Z	X	Y	Z
1	C	1.76293	1.38328	-0.09950	-1.79526	-1.26435	0.33128	1.67324	1.36215	-0.33676	-1.69514	-1.32734	0.14235
2	C	0.78478	-1.17252	-0.66026	-0.78897	1.21496	-0.47044	0.82511	-1.29751	-0.48323	-0.84145	1.29106	-0.33735
3	C	0.35750	1.16432	-0.18304	-0.40352	-1.11671	0.06419	0.28061	1.06243	-0.37096	-0.31465	-1.06287	-0.09155
4	C	2.68594	0.34584	-0.29377	-2.68938	-0.19094	0.21207	2.64667	0.35409	-0.37729	-2.65271	-0.30352	0.14609
5	C	2.16942	-0.93275	-0.57546	-2.16094	1.04540	-0.20386	2.19561	-0.97752	-0.45453	-2.20096	1.00512	-0.11046
6	C	-0.11650	-0.13387	-0.45402	0.08667	0.14435	-0.32686	-0.12540	-0.28450	-0.43178	0.09594	0.26469	-0.31963
7	H	0.41820	-2.16162	-0.89797	-0.41446	2.17358	-0.80214	0.50363	-2.32805	-0.54528	-0.51903	2.30510	-0.52977
8	C	-0.57242	2.27770	-0.02299	0.49188	-2.26478	0.16460	-0.70643	2.13850	-0.41666	0.64597	-2.16096	-0.16702
9	C	-1.99331	2.02967	-0.18366	1.89088	-2.09184	-0.18118	-2.10537	1.79258	-0.56891	2.01748	-1.86107	-0.52606
10	H	-2.66285	2.87705	-0.08136	2.53227	-2.96446	-0.11834	-2.81507	2.60879	-0.65187	2.70117	-2.69722	-0.62698
11	C	-2.49366	0.79905	-0.42500	2.40754	-0.89873	-0.54514	-2.54543	0.51266	-0.59731	2.46499	-0.59857	-0.72178
12	C	-1.61402	-0.44435	-0.56443	1.56959	0.37724	-0.63953	-1.60525	-0.68177	-0.41921	1.56974	0.62799	-0.52898
13	O	3.09583	-1.91035	-0.75845	-3.06218	2.05548	-0.32536	3.17035	-1.92313	-0.49699	-3.16332	1.96418	-0.11721
14	C	4.17302	0.59667	-0.19383	-4.16308	-0.36920	0.49647	4.11925	0.69152	-0.33452	-4.11392	-0.60437	0.38857
15	H	4.69257	-0.05369	-0.90364	-4.28299	-1.14867	1.25424	4.66870	-0.03418	-0.94128	-4.18911	-1.48383	1.03418
16	H	4.37591	1.62992	-0.48917	-4.55900	0.55932	0.91812	4.26782	1.67550	-0.78814	-4.56536	0.23465	0.92619
17	C	4.73710	0.35458	1.22021	-4.98334	-0.74752	-0.75268	4.70003	0.69968	1.09350	-4.90687	-0.85916	-0.90860
18	H	4.57430	-0.68070	1.53684	-4.63219	-1.69129	-1.18265	4.59108	-0.28063	1.56841	-4.49933	-1.71458	-1.45725
19	H	4.25851	1.01315	1.95240	-4.90665	0.02661	-1.52303	4.19159	1.43921	1.72074	-4.87422	0.01440	-1.56761
20	H	5.81479	0.55043	1.24156	-6.04153	-0.86572	-0.49484	5.76644	0.94962	1.07165	-5.95681	-1.07246	-0.67992
21	O	2.24009	2.61923	0.16862	-2.28767	-2.46436	0.71137	2.08593	2.64750	-0.27344	-2.11229	-2.59364	0.36346
22	H	1.43895	3.21166	0.25625	-1.50655	-3.08848	0.73595	1.25549	3.20514	-0.28208	-1.29507	-3.16770	0.30719
23	O	-0.16443	3.44531	0.22422	0.07007	-3.39933	0.51911	-0.35885	3.35010	-0.36248	0.29472	-3.35510	0.03860
24	O	-3.84239	0.66485	-0.41908	3.75094	-0.82840	-0.71197	-3.88189	0.33021	-0.63831	3.78729	-0.45064	-0.94762
25	C	-4.55796	-0.08534	-1.42924	4.36378	-0.16219	-1.84155	-4.53494	-0.76452	-1.32321	4.35844	0.56294	-1.80819
26	H	-4.35941	-1.15542	-1.36727	4.21835	0.91774	-1.81272	-4.31817	-1.72770	-0.86186	4.21343	1.56837	-1.41387
27	H	-4.29442	0.28062	-2.42438	3.96217	-0.56237	-2.77550	-4.23933	-0.78376	-2.37386	3.92989	0.49402	-2.80981
28	H	-5.60948	0.11361	-1.22314	5.42387	-0.40049	-1.75688	-5.59741	-0.53849	-1.23572	5.42079	0.32166	-1.83654

29	C	2.66828	-3.24352	-1.06284	-2.61724	3.35753	-0.72471	2.81070	-3.30678	-0.59418	-2.79954	3.33057	-0.34939
30	H	3.58374	-3.82567	-1.16469	-3.51213	3.97912	-0.73623	3.75450	-3.85066	-0.62106	-2.11161	3.69154	0.42186
31	H	2.10875	-3.27328	-2.00337	-2.17570	3.33283	-1.72627	2.24765	-3.50379	-1.51219	-3.73213	3.89178	-0.29861
32	H	2.05964	-3.65650	-0.25186	-1.89696	3.76497	-0.00793	2.22928	-3.62440	0.27734	-2.35026	3.45514	-1.33993
33	O	-1.87599	-1.07187	-1.83781	1.69368	0.93081	-1.96711	-1.85372	-1.66606	-1.43396	1.69785	1.51904	-1.64681
34	H	-1.47580	-0.53220	-2.53655	1.18120	0.38205	-2.58004	-1.47866	-1.35191	-2.27083	1.20860	1.14903	-2.39737
35	C	-2.04804	-1.52581	0.45535	2.17493	1.48351	0.25887	-1.95772	-1.43550	0.90679	2.09787	1.48070	0.67311
36	H	-3.11122	-1.74899	0.32678	3.22054	1.65074	-0.01528	-3.02146	-1.68057	0.89990	3.15700	1.69382	0.51599
37	H	-1.51657	-2.45278	0.22435	1.65597	2.42245	0.04846	-1.38176	-2.36104	0.90773	1.54344	2.41923	0.66301
38	C	-1.82972	-1.20289	1.91558	2.13219	1.24216	1.75017	-1.63394	-0.69201	2.18651	1.92069	0.85781	2.04265
39	O	-1.46471	-0.14106	2.38007	1.76368	0.23130	2.31486	-0.64478	-0.88308	2.86902	1.02983	1.13841	2.82269
40	O	-2.11271	-2.27688	2.68370	2.58191	2.32752	2.41595	-2.58325	0.20602	2.51504	2.88264	-0.04124	2.32851
41	H	-1.98137	-2.02982	3.61649	2.55598	2.13455	3.37009	-2.31510	0.65343	3.33776	2.70957	-0.40917	3.21375

Table S10: Energies and Boltzmann distribution of compound **19** (4*R*,12*R*).

Conformer	Hartrees	kcal/mol	ΔG (kcal/mol)	Boltzmann Distribution (%)
Conformer 19	-1261.44581	-791569.363	0	31.24047553
Conformer 16	-1261.44547	-791569.149	0.21335327	21.08701612
Conformer 13	-1261.4452	-791568.981	0.38152584	15.46896976
Conformer 20	-1261.4452	-791568.98	0.38215335	15.45109718
Conformer 7	-1261.44384	-791568.126	1.23619393	3.203666022
Conformer 15	-1261.44383	-791568.126	1.23682144	3.199964564
Conformer 11	-1261.44316	-791567.702	1.66101793	1.464699126
Conformer 17	-1261.44312	-791567.675	1.68800085	1.393668795
Conformer 8	-1261.44311	-791567.669	1.69364843	1.379243609
Conformer 23	-1261.44293	-791567.559	1.80409012	1.12532335
Conformer 9	-1261.44291	-791567.548	1.81475779	1.103423439
Conformer 22	-1261.44268	-791567.4	1.96285005	0.839952222
Conformer 4	-1261.44227	-791567.146	2.21699145	0.525917685
Conformer 18	-1261.44226	-791567.135	2.22765911	0.515682804
Conformer 5	-1261.44217	-791567.082	2.28099743	0.467419712
Conformer 6	-1261.44192	-791566.923	2.43975736	0.34888659
Conformer 14	-1261.44168	-791566.771	2.59161468	0.263745014
Conformer 12	-1261.44161	-791566.727	2.63554035	0.243242593
Conformer 25	-1261.44151	-791566.668	2.69452626	0.218195305
Conformer 3	-1261.44141	-791566.607	2.75539469	0.195049563
Conformer 24	-1261.44117	-791566.456	2.9066245	0.147620621
Conformer 10	-1261.44092	-791566.297	3.06601195	0.110058152
Conformer 1	-1261.43795	-791564.431	4.93159801	0.003539806
Conformer 2	-1261.43784	-791564.366	4.9962315	0.003142437

Table S11: XYZ coordinates of compound **19** (4*R*, 12*R*).

		Conformer 1			Conformer 2			Conformer 3			Conformer 4		
Row	Symbol	X	Y	Z	X	Y	Z	X	Y	Z	X	Y	Z
1	C	-1.599989	1.506256	-0.319231	-1.667085	1.383021	0.297499	-1.572870	1.582972	-0.134537	-1.645389	1.404394	0.402239
2	C	-1.038739	-1.168451	-0.906331	-1.081526	-1.095408	-0.855229	-1.009839	-1.002080	-1.033596	-1.049936	-0.981937	-0.924490
3	C	-0.246742	1.077664	-0.446662	-0.326228	1.072783	-0.072724	-0.219575	1.172206	-0.305874	-0.302322	1.123682	0.018779
4	C	-2.674117	0.622850	-0.494406	-2.716915	0.476004	0.097851	-2.646845	0.727085	-0.418469	-2.693195	0.512890	0.130488
5	C	-2.366772	-0.718436	-0.794135	-2.399925	-0.760167	-0.497540	-2.338346	-0.568228	-0.875469	-2.370963	-0.675448	-0.552068
6	C	0.013255	-0.278925	-0.726327	-0.051434	-0.188796	-0.637675	0.042255	-0.141788	-0.741906	-0.021542	-0.093277	-0.633515
7	H	-0.827940	-2.202973	-1.138854	-0.862802	-2.051769	-1.309279	-0.799189	-2.001440	-1.387962	-0.827829	-1.902580	-1.445614
8	C	0.845111	2.043922	-0.335689	0.731632	2.069070	0.089600	0.872054	2.121592	-0.082621	0.752752	2.110642	0.254592
9	C	2.205741	1.600911	-0.586255	2.064268	1.768371	-0.404469	2.230040	1.713135	-0.374993	2.084791	1.850577	-0.249998
10	H	2.970976	2.367423	-0.579635	2.792970	2.567228	-0.340880	2.996974	2.468933	-0.260315	2.815560	2.637773	-0.112085
11	C	2.479730	0.302881	-0.863774	2.350716	0.565399	-0.959271	2.511552	0.447445	-0.764923	2.387128	0.677887	-0.855446
12	C	1.439302	-0.809788	-0.830601	1.367863	-0.597483	-1.018427	1.468743	-0.653671	-0.912247	1.399140	-0.466701	-1.043277
13	O	-3.436840	-1.539299	-0.963590	-3.450461	-1.599938	-0.694064	-3.408186	-1.363176	-1.147982	-3.419668	-1.500688	-0.816157
14	C	-4.102319	1.098247	-0.356250	-4.134624	0.828418	0.486156	-4.075209	1.185377	-0.232721	-4.113943	0.834984	0.533926
15	H	-4.152380	2.150735	-0.649428	-4.648628	-0.078379	0.818249	-4.122547	2.264732	-0.403051	-4.627539	-0.093328	0.801216
16	H	-4.733587	0.535735	-1.050271	-4.105912	1.516703	1.335586	-4.704337	0.707458	-0.989247	-4.091428	1.464408	1.427983
17	C	-4.660064	0.944588	1.072677	-4.937513	1.473656	-0.660714	-4.639375	0.870195	1.166834	-4.913312	1.554077	-0.570781
18	H	-4.068662	1.522972	1.790062	-5.009305	0.798203	-1.519273	-4.048328	1.359289	1.948043	-4.978592	0.938324	-1.473585
19	H	-4.648477	-0.103246	1.389641	-4.464987	2.402282	-0.997236	-4.633401	-0.207430	1.360466	-4.442524	2.504937	-0.841140
20	H	-5.694569	1.301995	1.120614	-5.954552	1.711375	-0.330015	-5.672739	1.223947	1.252319	-5.932841	1.765731	-0.230075
21	O	-1.877571	2.798121	-0.032016	-1.956907	2.581088	0.853576	-1.852121	2.832283	0.301032	-1.940572	2.559471	1.040480
22	H	-0.994307	3.262611	0.030967	-1.090911	3.078369	0.904076	-0.966780	3.284959	0.419233	-1.073322	3.052048	1.129258
23	O	0.623368	3.255175	-0.064750	0.499881	3.191491	0.615054	0.642299	3.294243	0.325188	0.509287	3.192846	0.857745
24	O	3.679564	-0.193961	-1.188061	3.517030	0.215056	-1.514877	3.722731	-0.017394	-1.098705	3.570923	0.357575	-1.394481
25	C	4.808750	0.694101	-1.296664	4.588920	1.175410	-1.578693	4.855946	0.868155	-1.050515	4.647794	1.311037	-1.350056
26	H	4.618462	1.455792	-2.057601	4.274649	2.055675	-2.145858	4.707853	1.711422	-1.730904	4.912658	1.532637	-0.312598
27	H	5.645139	0.064335	-1.594106	5.403159	0.667147	-2.091826	5.706699	0.270196	-1.372559	4.364807	2.227731	-1.875006
28	H	5.014500	1.162761	-0.330558	4.899478	1.461846	-0.570181	5.011339	1.226198	-0.029139	5.483103	0.830525	-1.856438
29	C	-3.225336	-2.918740	-1.288142	-3.224801	-2.890764	-1.273595	-3.193893	-2.687824	-1.649351	-3.189729	-2.737877	-1.500746
30	H	-2.677136	-3.431123	-0.491029	-4.205056	-3.364652	-1.317314	-4.188890	-3.104794	-1.803096	-2.774697	-2.564277	-2.498910
31	H	-2.689604	-3.022450	-2.237250	-2.554538	-3.491031	-0.650019	-2.648173	-3.300534	-0.924510	-4.169239	-3.207098	-1.589619
32	H	-4.221293	-3.350780	-1.381986	-2.815327	-2.801855	-2.285055	-2.654100	-2.666186	-2.601610	-2.522318	-3.388815	-0.926746
33	O	1.505125	-1.524225	-2.079205	1.295237	-1.055824	-2.381672	1.546690	-1.154892	-2.263926	1.335331	-0.765100	-2.454426
34	H	2.427951	-1.786231	-2.225164	2.200060	-1.224872	-2.688681	2.470155	-1.396772	-2.438207	2.242370	-0.906546	-2.769128
35	C	1.778406	-1.838276	0.297982	1.893108	-1.797784	-0.164018	1.795591	-1.855457	0.030590	1.910271	-1.761284	-0.334178
36	H	1.030780	-2.633253	0.235962	1.174436	-2.612095	-0.287325	1.074579	-2.646246	-0.191042	1.214645	-2.565152	-0.587512
37	H	2.747281	-2.279511	0.045459	2.835442	-2.121931	-0.615843	2.782226	-2.227629	-0.261534	2.872842	-2.012859	-0.789544
38	C	1.891094	-1.401176	1.779262	2.186492	-1.641788	1.348518	1.851778	-1.659549	1.560394	2.144769	-1.779721	1.190979
39	C	0.560994	-0.948257	2.388098	0.934163	-1.394620	2.194410	0.527065	-1.301169	2.227343	0.896138	-1.619109	2.053200
40	O	0.564777	0.341973	2.763915	0.939325	-0.198560	2.807387	-0.409146	-2.242015	2.063645	-0.000038	-2.592343	1.858956
41	H	-0.309340	0.565004	3.131268	0.114587	-0.102796	3.316709	-1.225171	-1.961390	2.515321	-0.766349	-2.440491	2.440695
42	O	-0.391755	-1.689144	2.533831	0.038416	-2.207932	2.312704	0.370780	-0.287237	2.884669	0.768596	-0.724744	2.870903
43	O	2.965495	-0.512192	2.043783	3.252155	-0.752527	1.645382	2.841050	-0.718184	1.938798	3.117764	-0.829366	1.588723
44	H	2.717140	0.389909	1.794145	2.938656	0.162900	1.605367	2.400545	-0.022608	2.456412	2.709464	-0.256101	2.259954
45	H	2.137930	-2.317505	2.325201	2.538508	-2.627107	1.670813	2.126069	-2.641503	1.972002	2.523541	-2.786952	1.417054
		Conformer 5			Conformer 6			Conformer 7			Conformer 8		
Row	Symbol	X	Y	Z	X	Y	Z	X	Y	Z	X	Y	Z
1	C	-2.621047	0.957859	0.504063	-2.638391	1.075427	0.179073	-2.164111	-0.961689	-0.796908	-2.158791	-1.206548	-0.574637
2	C	-1.133418	-1.226596	-0.409825	-1.154972	-1.134441	-0.679090	-1.167491	0.921779	1.011497	-1.189311	0.674065	1.251844
3	C	-1.238506	1.118725	0.199100	-1.230551	1.194375	-0.005522	-0.836497	-1.093398	-0.296332	-0.787609	-1.235918	-0.187627
4	C	-3.271047	-0.275702	0.357254	-3.316792	-0.126458	-0.068553	-2.999514	0.094163	-0.407378	-3.053169	-0.260981	-0.054664

5	C	-2.504767	-1.356456	-0.119552	-2.549327	-1.222762	-0.506578	-2.480254	1.021924	0.516091	-2.542584	0.672527	0.867889
6	C	-0.505640	0.001544	-0.247918	-0.501460	0.064291	-0.425384	-0.349404	-0.122186	0.600048	-0.317261	-0.266797	0.719860
7	H	-0.559129	-2.067674	-0.771882	-0.580643	-1.986071	-1.015383	-0.789114	1.648337	1.716969	-0.819656	1.400672	1.962007
8	C	-0.616139	2.439722	0.270597	-0.571209	2.488841	0.159555	-0.015708	-2.240222	-0.678390	0.098785	-2.280750	-0.695368
9	C	0.737899	2.601039	-0.221496	0.824687	2.613690	-0.211604	1.287566	-2.412103	-0.059788	1.459715	-2.357269	-0.192566
10	H	1.112497	3.615877	-0.271459	1.237160	3.614935	-0.198686	1.823810	-3.319705	-0.308389	2.052942	-3.197374	-0.532443
11	C	1.468788	1.535439	-0.633434	1.550536	1.533450	-0.593248	1.776793	-1.490818	0.802197	1.933599	-1.436002	0.677948
12	C	1.006699	0.086316	-0.468147	1.026131	0.098640	-0.510106	1.080196	-0.175928	1.125866	1.150364	-0.209650	1.127175
13	O	-3.179135	-2.527626	-0.272670	-3.246907	-2.365317	-0.747183	-3.331248	2.016335	0.886926	-3.445501	1.565423	1.355669
14	C	-4.742203	-0.423508	0.672399	-4.811056	-0.233398	0.133578	-4.408709	0.208612	-0.942707	-4.504359	-0.247594	-0.477811
15	H	-4.922907	-1.427558	1.067661	-5.260359	0.748790	-0.038391	-4.675745	1.266557	-1.020354	-4.808201	-1.269035	-0.724165
16	H	-5.006345	0.288675	1.459370	-5.224208	-0.916409	-0.614571	-4.435777	-0.209037	-1.953350	-5.118623	0.080972	0.365630
17	C	-5.654394	-0.189585	-0.547976	-5.201797	-0.728509	1.540158	-5.453629	-0.514337	-0.070089	-4.777938	0.666465	-1.688537
18	H	-5.433364	-0.904680	-1.346932	-4.829013	-0.049063	2.313808	-5.473186	-0.101956	0.943937	-4.201221	0.344939	-2.562045
19	H	-5.522319	0.820394	-0.949767	-4.790126	-1.724046	1.735476	-5.231870	-1.584103	0.002512	-4.508325	1.704280	-1.467184
20	H	-6.707186	-0.307901	-0.268556	-6.291513	-0.787233	1.637600	-6.455325	-0.404344	-0.500162	-5.840152	0.641732	-1.956008
21	O	-3.343893	2.013588	0.942329	-3.358103	2.142918	0.593322	-2.653541	-1.873149	-1.669002	-2.633197	-2.114361	-1.458687
22	H	-2.710073	2.787951	0.967730	-2.703568	2.894273	0.688714	-1.918641	-2.535297	-1.815617	-1.857801	-2.700908	-1.692738
23	O	-1.254600	3.441328	0.699493	-1.209147	3.501132	0.563432	-0.432136	-3.099928	-1.502964	-0.306310	-3.137164	-1.528923
24	O	2.693708	1.599536	-1.171628	2.817826	1.565944	-1.025533	2.941502	-1.573434	1.463285	3.152058	-1.438759	1.239716
25	C	3.298368	2.888210	-1.391694	3.484114	2.836206	-1.156867	3.753986	-2.753624	1.317658	4.055241	-2.525657	0.962730
26	H	3.452267	3.405942	-0.440584	3.575917	3.322657	-0.181582	4.079396	-2.863624	0.279589	4.300562	-2.551926	-0.102486
27	H	2.671296	3.491027	-2.054190	2.936855	3.479339	-1.851391	3.196274	-3.638184	1.637354	3.611026	-3.474864	1.274632
28	H	4.256392	2.681705	-1.865622	4.470644	2.606043	-1.555445	4.614271	-2.595804	1.965608	4.948824	-2.317217	1.548314
29	C	-2.479785	-3.689251	-0.734556	-2.554945	-3.529719	-1.213262	-2.883989	3.023469	1.801989	-3.024399	2.542815	2.314241
30	H	-2.070383	-3.529470	-1.737274	-2.064311	-3.338941	-2.173190	-3.730849	3.697412	1.929187	-3.917650	3.119589	2.552956
31	H	-3.225136	-4.483575	-0.766886	-3.322764	-4.292464	-1.340643	-2.032513	3.577444	1.393527	-2.262159	3.205815	1.892208
32	H	-1.677145	-3.967178	-0.043740	-1.817960	-3.870610	-0.478783	-2.615507	2.586037	2.769094	-2.642565	2.065275	3.222447
33	O	1.313017	-0.664913	-1.633094	1.408410	-0.634325	-1.664079	0.986531	-0.042015	2.555589	1.164602	-0.153106	2.565032
34	H	2.257218	-0.921797	-1.603898	2.339754	-0.918174	-1.562936	1.875345	-0.161854	2.925499	2.088413	-0.206654	2.856321
35	C	1.661506	-0.524655	0.824634	1.542053	-0.568895	0.817070	1.952947	1.010042	0.603398	1.866883	1.076311	0.603560
36	H	1.258311	0.021968	1.681428	1.079726	-0.033564	1.650768	1.439675	1.939772	0.865176	1.296850	1.940717	0.955802
37	H	1.303339	-1.556649	0.901646	1.147466	-1.590386	0.829369	2.896668	0.978810	1.153002	2.852464	1.106799	1.074051
38	C	3.202260	-0.486227	1.019545	3.059352	-0.586608	1.149497	2.290016	1.033036	-0.891678	2.072538	1.202079	-0.910197
39	C	3.927592	-1.547147	0.189641	3.822337	-1.648267	0.355295	3.496548	1.949233	-1.143734	3.176901	2.227019	-1.207778
40	O	4.727721	-2.341617	0.902123	4.521503	-2.495495	1.111932	3.340461	2.718992	-2.234000	2.865337	3.036276	-2.234272
41	H	5.142161	-2.987478	0.300824	4.966571	-3.139187	0.530561	4.158102	3.229528	-2.373590	3.624161	3.620663	-2.411284
42	O	3.795470	-1.657023	-1.021903	3.801606	-1.715450	-0.866323	4.508904	1.943073	-0.469205	4.241868	2.270376	-0.621432
43	O	3.533786	-0.548359	2.397167	3.264132	-0.697304	2.548542	1.140669	1.414631	-1.628130	0.837674	1.523686	-1.527047
44	H	3.278050	-1.409631	2.759403	2.951396	-1.559565	2.860006	1.358698	1.430340	-2.570894	0.972050	1.608894	-2.481616
45	H	3.596865	0.472379	0.672376	3.512166	0.367673	0.867734	2.634685	0.035919	-1.209543	2.465173	0.253967	-1.311214
		**Conformer 9**			**Conformer 10**			**Conformer 11**			**Conformer 12**		
Row	Symbol	X	Y	Z	X	Y	Z	X	Y	Z	X	Y	Z
1	C	-2.171379	-0.994718	-0.761411	-1.912645	-1.310285	-0.384691	-2.150618	1.251478	0.520287	1.896138	-1.454481	-0.086813
2	C	-1.113813	0.921781	0.975785	-1.309802	1.213865	0.656686	-1.144676	-0.693184	-1.216897	1.314056	1.179674	-0.822809
3	C	-0.835837	-1.131633	-0.284066	-0.598871	-1.037861	0.096804	-0.773643	1.272414	0.154341	0.553866	-1.084378	-0.392747
4	C	-2.985088	0.082462	-0.383509	-2.926852	-0.343127	-0.351793	-3.033072	0.282443	0.022734	2.950007	-0.532382	-0.152844
5	C	-2.434998	1.027677	0.503180	-2.603642	0.916144	0.189088	-2.504341	-0.683519	-0.854609	2.633449	0.787245	-0.528787
6	C	-0.318275	-0.145393	0.577858	-0.315756	0.244246	0.608068	-0.284876	0.272154	-0.708103	0.282866	0.251121	-0.750932
7	H	-0.715330	1.658833	1.659141	-1.085010	2.187437	1.069537	-0.762934	-1.441499	-1.897645	1.095640	2.197038	-1.116355
8	C	-0.033376	-2.292068	-0.664442	0.415360	-2.091801	0.108358	0.103092	2.332860	0.646222	-0.508245	-2.090005	-0.384918
9	C	1.287127	-2.458657	-0.082623	1.709411	-1.818445	0.707664	1.476456	2.388531	0.175875	-1.844974	-1.707200	-0.803313
10	H	1.813735	-3.370790	-0.334956	2.401165	-2.650042	0.761633	2.065515	3.235108	0.506676	-2.580681	-2.500528	-0.851696
11	C	1.803755	-1.528187	0.752428	2.012532	-0.588585	1.182873	1.967635	1.441079	-0.655475	-2.135299	-0.425748	-1.125783
12	C	1.116193	-0.212390	1.090487	1.085265	0.615463	1.079096	1.187685	0.210491	-1.097941	-1.140200	0.723115	-1.024312
13	O	-3.264362	2.043653	0.863328	-3.621367	1.816092	0.222667	-3.395401	-1.598578	-1.321954	3.684463	1.646834	-0.585772

14	C	-4.403798	0.200485	-0.892115	-4.317399	-0.657284	-0.854456	-4.490708	0.278620	0.423218	4.366123	-0.945705	0.176655
15	H	-4.659324	1.259200	-0.993993	-4.752994	0.248099	-1.287082	-4.800864	1.306719	0.630606	4.492281	-2.001644	-0.078744
16	H	-4.457752	-0.245138	-1.889595	-4.242698	-1.398078	-1.655577	-5.090754	-0.079671	-0.418394	5.058682	-0.373981	-0.447869
17	C	-5.438237	-0.484143	0.022991	-5.254780	-1.194323	0.245191	-4.780734	-0.595447	1.659375	4.732058	-0.737407	1.659544
18	H	-5.430696	-0.042998	1.025001	-5.373065	-0.464375	1.052516	-4.217494	-0.243981	2.530121	4.076518	-1.323066	2.312329
19	H	-5.228147	-1.554199	0.121330	-4.862051	-2.119841	0.678799	-4.506436	-1.639491	1.476443	4.641236	0.316028	1.943616
20	H	-6.447539	-0.373433	-0.388632	-6.247020	-1.407670	-0.167358	-5.846807	-0.563959	1.910115	5.764825	-1.051223	1.847090
21	O	-2.690132	-1.920880	-1.599477	-2.211157	-2.529802	-0.885806	-2.642564	2.188982	1.361933	2.183457	-2.726245	0.269989
22	H	-1.965849	-2.594769	-1.746912	-1.372471	-3.069974	-0.814643	-1.872663	2.785737	1.589124	1.316898	-3.224572	0.236223
23	O	-0.478693	-3.165693	-1.458303	0.173700	-3.237267	-0.360024	-0.319524	3.217760	1.439996	-0.275533	-3.284557	-0.055027
24	O	2.987239	-1.605556	1.380412	3.154892	-0.241222	1.794253	3.200360	1.421769	-1.185350	-3.320067	0.025965	-1.563457
25	C	3.792227	-2.790109	1.227348	4.161924	-1.243107	2.031652	4.102268	2.511801	-0.915516	-4.402085	-0.904149	-1.759325
26	H	4.086715	-2.915444	0.181833	4.530890	-1.641256	1.082659	4.319776	2.566888	0.154594	-4.677565	-1.369423	-0.808988
27	H	3.242009	-3.668432	1.575925	3.755346	-2.047802	2.650424	3.671589	3.453874	-1.265603	-4.117030	-1.667058	-2.488895
28	H	4.671590	-2.625512	1.847392	4.963351	-0.729593	2.559698	5.009542	2.282325	-1.471396	-5.230601	-0.309554	-2.139884
29	C	-2.787082	3.066604	1.745144	-3.384652	3.133018	0.735215	-2.956335	-2.607939	-2.238458	3.464281	3.009056	-0.971773
30	H	-1.935050	3.597815	1.308530	-3.081282	3.097578	1.786550	-2.197630	-3.251847	-1.781797	3.051692	3.070064	-1.983952
31	H	-2.508296	2.649443	2.718230	-4.337696	3.654027	0.647932	-2.563450	-2.161667	-3.157745	4.446918	3.479442	-0.949112
32	H	-3.621924	3.755839	1.869526	-2.625544	3.655284	0.144013	-3.843591	-3.197128	-2.468931	2.798918	3.515777	-0.265203
33	O	1.033151	-0.091950	2.522779	0.941220	1.184475	2.392523	1.216725	0.138186	-2.535285	-1.111755	1.404364	-2.291256
34	H	1.923121	-0.223649	2.886246	1.830272	1.343790	2.747146	2.143096	0.192670	-2.818652	-2.024342	1.636977	-2.524988
35	C	1.989994	0.974243	0.573200	1.713021	1.710608	0.158429	1.895317	-1.070685	-0.551641	-1.603908	1.754140	0.053683
36	H	1.491715	1.902268	0.870464	1.125336	2.617359	0.324520	1.332661	-1.937189	-0.913416	-0.987069	2.644254	-0.095378
37	H	2.943479	0.926240	1.107900	2.734974	1.906633	0.501063	2.888400	-1.107176	-1.009723	-2.644016	2.028457	-0.153841
38	C	2.299231	1.014764	-0.938649	1.723761	1.490709	-1.364912	2.078322	-1.173045	0.977822	-1.465183	1.391282	1.542701
39	C	3.418746	2.035325	-1.171313	2.725145	0.427437	-1.825145	3.077258	-2.298393	1.268946	-2.476824	0.343199	2.015781
40	O	2.970136	3.195973	-1.677720	2.143880	-0.599424	-2.462434	2.486362	-3.417741	1.718944	-1.896170	-0.763484	2.502427
41	H	3.721945	3.808431	-1.769786	2.832392	-1.233193	-2.736055	3.168619	-4.099710	1.853888	-2.589834	-1.382654	2.795831
42	O	4.585802	1.827638	-0.897224	3.927545	0.531554	-1.665157	4.275536	-2.198293	1.083480	-3.680940	0.521068	1.991771
43	O	1.168024	1.241303	-1.758918	2.062603	2.710678	-2.027881	0.869419	-1.307259	1.701847	-1.654347	2.558647	2.345442
44	H	0.766491	2.092221	-1.529225	2.975151	2.944268	-1.795317	0.414948	-2.116927	1.426520	-2.566435	2.868214	2.228803
45	H	2.707442	0.051915	-1.259179	0.728316	1.226652	-1.724345	2.547009	-0.259639	1.355250	-0.455659	1.042446	1.764036
		**Conformer 13**			**Conformer 14**			**Conformer 15**			**Conformer 16**		
Row	Symbol	X	Y	Z	X	Y	Z	X	Y	Z	X	Y	Z
1	C	2.171153	0.978317	-0.773212	-2.622159	0.968759	0.483338	-2.164124	-0.961620	-0.796865	-2.148605	-1.230004	-0.547627
2	C	1.154426	-0.938206	0.988643	-1.108377	-1.215225	-0.388935	-1.167394	0.921853	1.011468	-1.178630	0.687275	1.240602
3	C	0.838109	1.101228	-0.284824	-1.233857	1.133177	0.207164	-0.836513	-1.093374	-0.296306	-0.774770	-1.244708	-0.168901
4	C	3.002226	-0.084499	-0.393680	-3.265026	-0.267281	0.326475	-2.999479	0.094274	-0.407348	-3.045057	-0.280860	-0.037943
5	C	2.472388	-1.029719	0.505767	-2.485491	-1.347884	-0.128575	-2.480163	1.022038	0.516081	-2.534179	0.671252	0.865316
6	C	0.341198	0.114093	0.588310	-0.488959	0.016381	-0.220346	-0.349361	-0.122161	0.600036	-0.304671	-0.257519	0.718867
7	H	0.768605	-1.677294	1.676851	-0.525220	-2.058463	-0.731293	-0.788977	1.648410	1.716920	-0.808508	1.428237	1.935510
8	C	0.022130	2.255623	-0.654320	-0.616932	2.456258	0.290287	-0.015770	-2.240218	-0.678375	0.114090	-2.293957	-0.662824
9	C	-1.286902	2.418086	-0.045251	0.748119	2.620848	-0.171856	1.287493	-2.412165	-0.059773	1.478237	-2.354614	-0.166096
10	H	-1.820228	3.330031	-0.283920	1.123372	3.635833	-0.213175	1.823672	-3.319810	-0.308354	2.073824	-3.197843	-0.493810
11	C	-1.785162	1.482596	0.795562	1.486923	1.556600	-0.568830	1.776768	-1.490899	0.802207	1.952511	-1.414570	0.683558
12	C	-1.092387	0.162157	1.104085	1.024381	0.109036	-0.415965	1.080239	-0.175961	1.125835	1.165451	-0.183545	1.112826
13	O	3.318992	-2.031394	0.867005	-3.152586	-2.521503	-0.290724	-3.331119	2.016478	0.886938	-3.439379	1.567192	1.343184
14	C	4.418077	-0.187874	-0.913344	-4.742253	-0.418969	0.609571	-4.408686	0.208752	-0.942642	-4.498871	-0.282613	-0.452174
15	H	4.686918	-1.243909	-1.008690	-4.927182	-1.419856	1.010920	-4.675687	1.266702	-1.020331	-4.797476	-1.309253	-0.682656
16	H	4.457758	0.249975	-1.914944	-5.027588	0.299862	1.382866	-4.435803	-0.208949	-1.953262	-5.110185	0.053527	0.390463
17	C	5.450952	0.518063	-0.012837	-5.627080	-0.202219	-0.633969	-5.453597	-0.514118	-0.069947	-4.785554	0.612910	-1.673675
18	H	5.457793	0.085206	0.992794	-5.384619	-0.924687	-1.419947	-5.473105	-0.101679	0.944057	-4.211467	0.283383	-2.545913
19	H	5.227159	1.585965	0.078601	-5.490372	0.804036	-1.043503	-5.231863	-1.583885	0.002704	-4.521854	1.655443	-1.468042
20	H	6.458230	0.417602	-0.432039	-6.685429	-0.322500	-0.377437	-6.455304	-0.404120	-0.499989	-5.849048	0.577257	-1.934730
21	O	2.670389	1.905724	-1.622425	-3.357575	2.023181	0.902746	-2.653578	-1.873069	-1.668958	-2.623080	-2.155536	-1.412775
22	H	1.936881	2.570038	-1.765953	-2.727716	2.799828	0.940508	-1.918664	-2.535208	-1.815572	-1.845729	-2.741516	-1.642000

23	O	0.447340	3.129500	-1.459027	-1.266743	3.456646	0.702762	-0.432246	-3.099916	-1.502939	-0.291114	-3.167234	-1.478445
24	O	-2.957166	1.553921	1.445663	2.721559	1.615086	-1.086016	2.941439	-1.573588	1.463341	3.174738	-1.399730	1.237502
25	C	-3.767727	2.736900	1.311957	3.337509	2.900068	-1.297990	3.753820	-2.753865	1.317786	4.081348	-2.487942	0.976596
26	H	-4.081366	2.865527	0.272401	2.724995	3.504757	-1.972141	4.079310	-2.863880	0.279746	4.320573	-2.534024	-0.089311
27	H	-3.213505	3.615379	1.653719	4.302481	2.687247	-1.754513	3.195982	-3.638365	1.637425	3.643132	-3.432744	1.309546
28	H	-4.635281	2.567905	1.947300	3.477080	3.417063	-0.344485	4.614057	-2.596132	1.965819	4.977261	-2.264181	1.552890
29	C	2.861065	-3.056094	1.756831	-2.439538	-3.684364	-0.728414	-2.883818	3.023561	1.802034	-3.018084	2.563832	2.281537
30	H	2.013754	-3.601033	1.328033	-2.007582	-3.530142	-1.722516	-3.730663	3.697514	1.929281	-2.627639	2.104732	3.195577
31	H	2.582235	-2.637766	2.729458	-3.181891	-4.480874	-0.772583	-2.032341	3.577534	1.393573	-3.913183	3.139564	2.515795
32	H	3.706002	-3.733212	1.879885	-1.652578	-3.955281	-0.017145	-2.615321	2.586079	2.769112	-2.262492	3.223707	1.842945
33	O	-1.009152	0.005672	2.532217	1.338507	-0.635668	-1.591181	0.986578	-0.041990	2.555558	1.192984	-0.093055	2.548365
34	H	-1.899505	0.126097	2.898260	2.302984	-0.653317	-1.703487	1.875412	-0.161679	2.925460	2.119473	-0.138573	2.832457
35	C	-1.963566	-1.014114	0.555551	1.667085	-0.518942	0.870507	1.953033	1.009948	0.603312	1.868093	1.094621	0.550269
36	H	-1.448810	-1.947990	0.797549	1.283711	0.034004	1.732816	1.439598	1.939690	0.864726	1.298937	1.963045	0.893104
37	H	-2.905711	-0.991489	1.110562	1.286836	-1.542327	0.952307	2.896619	0.978942	1.153160	2.859934	1.134400	1.009476
38	C	-2.294618	-0.995744	-0.949836	3.209915	-0.532709	1.068757	2.290516	1.032576	-0.891674	2.040857	1.180393	-0.979212
39	C	-3.368271	-2.032181	-1.273336	3.903442	-1.638278	0.276988	3.496351	1.949669	-1.143730	3.008515	2.302904	-1.346730
40	O	-4.526647	-1.808094	-0.643638	4.066206	-1.342413	-1.024978	3.339823	2.719122	-2.234151	4.238602	2.124090	-0.853287
41	H	-5.166551	-2.497794	-0.896231	4.478003	-2.101294	-1.477890	4.157090	3.230264	-2.373693	4.804519	2.867466	-1.129112
42	O	-3.176425	-2.958043	-2.041983	4.266149	-2.685401	0.776646	4.508607	1.944434	-0.469040	2.680097	3.253472	-2.034812
43	O	-1.156665	-1.244299	-1.755792	3.492844	-0.728880	2.447373	1.141076	1.412891	-1.628632	0.811141	1.394951	-1.649081
44	H	-1.332550	-2.061225	-2.254348	3.633252	-1.680030	2.589592	1.359352	1.428324	-2.571343	0.883600	2.247791	-2.112057
45	H	-2.715686	-0.017687	-1.221842	3.644341	0.426228	0.778837	2.636100	0.035594	-1.208982	2.490349	0.249235	-1.351684
		**Conformer 17**			**Conformer 18**			**Conformer 19**			**Conformer 20**		
Row	Symbol	X	Y	Z	X	Y	Z	X	Y	Z	X	Y	Z
1	C	-2.158754	1.206765	0.574828	-2.633124	1.086306	0.154950	-2.624935	0.953248	0.507833	2.171152	0.978312	-0.773215
2	C	-1.189495	-0.673952	-1.251758	-1.132561	-1.129034	-0.658975	-1.087822	-1.216369	-0.362809	1.154424	-0.938205	0.988648
3	C	-0.787580	1.236020	0.187676	-1.221732	1.204488	-0.000920	-1.241640	1.134021	0.220540	0.838108	1.101226	-0.284825
4	C	-3.053194	0.261209	0.054929	-3.306583	-0.116269	-0.102185	-3.252450	-0.292432	0.360856	3.002223	-0.084504	-0.393680
5	C	-2.542731	-0.672312	-0.867680	-2.530570	-1.215563	-0.516943	-2.462480	-1.365565	-0.093247	2.472385	-1.029721	0.505771
6	C	-0.317355	0.266879	-0.719853	-0.484970	0.071907	-0.400060	-0.485792	0.024892	-0.206126	0.341197	0.114094	0.588312
7	H	-0.819926	-1.400563	-1.961961	-0.553349	-1.985176	-0.974881	-0.495982	-2.055973	-0.700238	0.768602	-1.677292	1.676856
8	C	0.098922	2.280778	0.695333	-0.565928	2.499739	0.173562	-0.639557	2.463854	0.288268	0.022130	2.255621	-0.654326
9	C	1.459850	2.357187	0.192535	0.837825	2.623746	-0.170331	0.714370	2.642405	-0.204786	-1.286902	2.418087	-0.045256
10	H	2.053135	3.197241	0.532437	1.251789	3.624271	-0.151688	1.070105	3.663022	-0.267500	-1.820227	3.330031	-0.283929
11	C	1.933639	1.435920	-0.678044	1.567787	1.542368	-0.536413	1.466018	1.590562	-0.612004	-1.785162	1.482600	0.795561
12	C	1.150247	0.209638	-1.127258	1.041963	0.110286	-0.459816	1.027657	0.141338	-0.405483	-1.092388	0.162162	1.104088
13	O	-3.445758	-1.565122	-1.355408	-3.223163	-2.358873	-0.765884	-3.114191	-2.547684	-0.244884	3.318988	-2.031397	0.867009
14	C	-4.504368	0.247784	0.478150	-4.805109	-0.221196	0.066453	-4.725606	-0.461208	0.654303	4.418074	-0.187883	-0.913345
15	H	-4.807831	1.269011	0.725796	-5.249774	0.759931	-0.122828	-4.895691	-1.463776	1.057664	4.686915	-1.243918	-1.008683
16	H	-5.118804	-0.079499	-0.365681	-5.201076	-0.909314	-0.686287	-5.013838	0.255054	1.428837	4.457753	0.249959	-1.914949
17	C	-4.778218	-0.667695	1.687743	-5.228427	-0.706217	1.467070	-5.620755	-0.255667	-0.583806	5.450950	0.518061	-0.012846
18	H	-4.201445	-0.347385	2.561666	-4.873508	-0.021327	2.244290	-5.375497	-0.976200	-1.370667	5.457794	0.085212	0.992788
19	H	-4.508877	-1.705302	1.465139	-4.821782	-1.700432	1.678911	-5.498425	0.751734	-0.994977	5.227158	1.585964	0.078586
20	H	-5.840428	-0.642997	1.955226	-6.320138	-0.763943	1.539392	-6.675728	-0.387834	-0.319742	6.458228	0.417598	-0.432048
21	O	-2.632985	2.114601	1.458915	-3.361445	2.155215	0.549502	-3.370487	1.998956	0.928048	2.670389	1.905715	-1.622432
22	H	-1.857510	2.701161	1.692910	-2.709950	2.907015	0.656836	-2.752598	2.784152	0.961050	1.936882	2.570029	-1.765964
23	O	-0.306085	3.137246	1.528912	-1.211006	3.513075	0.561033	-1.292745	3.458265	0.705484	0.447339	3.129493	-1.459037
24	O	3.152014	1.438615	-1.239915	2.841636	1.566126	-0.950827	2.677215	1.672501	-1.177397	-2.957165	1.553929	1.445663
25	C	4.055420	2.525309	-0.962746	3.518408	2.831290	-1.080347	3.249564	2.973048	-1.420135	-3.767724	2.736908	1.311955
26	H	3.611442	3.474637	-1.274602	3.598749	3.321677	-0.106211	3.412136	3.499380	-0.475530	-4.081365	2.865534	0.272399
27	H	4.949008	2.316729	-1.548265	2.984839	3.473270	-1.786409	2.595549	3.556731	-2.073571	-3.213500	3.615388	1.653714
28	H	4.300635	2.551431	0.102495	4.509017	2.592184	-1.463064	4.201283	2.781737	-1.912405	-4.635278	2.567917	1.947298
29	C	-3.024824	-2.542450	-2.314129	-2.521875	-3.527718	-1.206686	-2.388928	-3.705398	-0.677857	2.861065	-3.056085	1.756851
30	H	-2.262627	-3.205576	-1.892223	-2.008718	-3.344784	-2.156329	-1.964241	-3.552904	-1.675289	2.582237	-2.637744	2.729473
31	H	-2.642987	-2.064849	-3.222299	-3.287607	-4.290399	-1.346220	-3.122029	-4.510753	-0.712498	3.706004	-3.733200	1.879912

32	H	-3.918143	-3.119102	-2.552876	-1.803015	-3.863722	-0.452301	-1.595677	-3.961369	0.031794	2.013754	-3.601033	1.328062
33	O	1.164426	0.153016	-2.565048	1.432646	-0.622024	-1.619284	1.451556	-0.673912	-1.512439	-1.009152	0.005683	2.532221
34	H	2.088253	0.205589	-2.856438	2.401929	-0.677884	-1.643322	1.146307	-0.308028	-2.356383	-1.899502	0.126123	2.898265
35	C	1.866738	-1.076354	-0.603562	1.543766	-0.567525	0.863494	1.709584	-0.464134	0.860607	-1.963568	-1.014112	0.555561
36	H	1.296513	-1.940744	-0.955538	1.094309	-0.026240	1.700825	1.386955	0.133252	1.716664	-1.448812	-1.947986	0.797565
37	H	2.852194	-1.107007	-1.074311	1.132431	-1.582073	0.880533	1.300319	-1.468760	1.004812	-2.905713	-0.991482	1.110569
38	C	2.072879	-1.201901	0.910136	3.059901	-0.627145	1.205445	3.255457	-0.506262	0.932911	-2.294617	-0.995750	-0.949825
39	C	3.176943	-2.227163	1.207505	3.797740	-1.734587	0.457974	3.916008	-1.742046	0.296582	-3.368269	-2.032183	-1.273342
40	O	2.865524	-3.035902	2.234486	4.073865	-1.424935	-0.821324	3.604167	-2.056016	-0.953786	-4.526619	-1.808160	-0.643576
41	H	3.624191	-3.620519	2.411397	4.509898	-2.184883	-1.249067	2.849679	-1.502366	-1.296831	-5.166530	-2.497841	-0.896205
42	O	4.241612	-2.271166	0.620677	4.099076	-2.794874	0.970586	4.747781	-2.384248	0.921230	-3.176448	-2.957981	-2.042074
43	O	0.838144	-1.522868	1.527581	3.206456	-0.857368	2.599856	3.617426	-0.463572	2.306730	-1.156654	-1.244307	-1.755765
44	H	0.973271	-1.609715	2.481896	3.319112	-1.813564	2.732849	4.328940	-1.119364	2.422767	-1.332614	-2.061128	-2.254468
45	H	2.466089	-0.253869	1.310799	3.543269	0.325403	0.978102	3.680506	0.362230	0.414287	-2.715694	-0.017694	-1.221833
		**Conformer 22**			**Conformer 23**			**Conformer 24**			**Conformer 25**		
Row	Symbol	X	Y	Z	X	Y	Z	X	Y	Z	X	Y	Z
1	C	-2.478254	-1.213691	-0.385275	2.490256	-1.285621	0.024457	-2.480703	-1.212946	-0.387385	-2.489365	1.286785	0.028684
2	C	-1.419847	1.298415	0.233389	1.436688	1.256464	-0.466757	-1.428693	1.300898	0.236174	-1.447793	-1.259349	-0.469129
3	C	-1.091635	-1.082616	-0.084031	1.086875	-1.123171	-0.162546	-1.094083	-1.078564	-0.087840	-1.086943	1.118201	-0.159115
4	C	-3.342746	-0.110086	-0.380549	3.374904	-0.199959	-0.041451	-3.348379	-0.111846	-0.378501	-3.379129	0.205483	-0.040316
5	C	-2.789517	1.141728	-0.049803	2.821192	1.069197	-0.296876	-2.798235	1.140819	-0.045698	-2.831482	-1.065588	-0.299486
6	C	-0.576380	0.194839	0.211294	0.575376	0.168900	-0.393950	-0.582366	0.199539	0.210370	-0.581795	-0.175724	-0.393496
7	H	-1.013211	2.267764	0.485314	1.030801	2.238189	-0.666729	-1.024181	2.270940	0.488775	-1.045922	-2.242620	-0.669530
8	C	-0.240334	-2.268099	-0.001032	0.208289	-2.290988	-0.198044	-0.238448	-2.261486	-0.010856	-0.202410	2.281788	-0.192044
9	C	1.119706	-2.117708	0.475699	-1.182683	-2.106069	-0.559340	1.121312	-2.108765	0.465769	1.186656	2.091502	-0.557748
10	H	1.678662	-3.032462	0.629242	-1.770511	-3.006952	-0.684293	1.682947	-3.022463	0.615282	1.777926	2.990072	-0.682494
11	C	1.645666	-0.894684	0.731051	-1.703529	-0.868691	-0.746735	1.642870	-0.885135	0.726988	1.700886	0.852020	-0.749124
12	C	0.920889	0.417762	0.437678	-0.930835	0.424125	-0.489816	0.914771	0.424984	0.431690	0.923233	-0.435927	-0.483508
13	O	-3.666801	2.180847	-0.029146	3.713666	2.093289	-0.365775	-3.678536	2.177418	-0.021724	-3.729112	-2.085010	-0.372007
14	C	-4.813755	-0.271556	-0.689376	4.860806	-0.393480	0.157576	-4.819325	-0.276634	-0.685925	-4.864043	0.405316	0.159930
15	H	-5.171234	0.626903	-1.201206	5.132869	-1.397589	-0.180164	-5.179427	0.621249	-1.196983	-5.131233	1.412397	-0.172790
16	H	-4.937867	-1.110783	-1.379745	5.400844	0.319762	-0.471969	-4.942105	-1.115787	-1.376603	-5.407759	-0.302167	-0.472991
17	C	-5.677143	-0.516084	0.563954	5.307901	-0.214652	1.621938	-5.681007	-0.523793	0.568054	-5.311689	0.221460	1.623504
18	H	-5.597946	0.320593	1.265547	4.806755	-0.935214	2.276672	-5.602948	0.312613	1.270097	-4.806727	0.936208	2.281681
19	H	-5.364692	-1.427116	1.084858	5.075881	0.792119	1.984354	-5.366121	-1.434488	1.088105	-5.084604	-0.788252	1.980841
20	H	-6.731414	-0.628459	0.287745	6.388837	-0.367482	1.714668	-6.735302	-0.638286	0.292782	-6.391827	0.379263	1.717331
21	O	-2.994528	-2.428782	-0.679180	3.002981	-2.514504	0.261287	-2.993676	-2.428853	-0.683765	-2.996039	2.517508	0.269263
22	H	-2.231307	-3.073319	-0.616536	2.225474	-3.144636	0.240829	-2.227960	-3.070948	-0.625119	-2.215173	3.143579	0.251228
23	O	-0.684243	-3.414541	-0.289427	0.651330	-3.451107	0.031004	-0.679361	-3.408212	-0.302988	-0.639830	3.443252	0.041291
24	O	2.872801	-0.668469	1.217550	-2.962307	-0.610778	-1.124671	2.866413	-0.657054	1.223278	2.955327	0.588246	-1.139308
25	C	3.699274	-1.785398	1.594648	-3.839604	-1.705827	-1.447444	3.697778	-1.776596	1.582012	3.836474	1.682826	-1.453499
26	H	3.919283	-2.413180	0.726651	-3.420474	-2.291620	-2.269964	4.603912	-1.342634	2.001121	3.410115	2.290687	-2.255971
27	H	3.201599	-2.374462	2.369751	-4.778043	-1.245651	-1.750926	3.938420	-2.372634	0.697122	4.765539	1.221867	-1.784257
28	H	4.617398	-1.348585	1.982927	-3.998533	-2.343125	-0.572966	3.194525	-2.394745	2.330241	4.013916	2.297225	-0.566232
29	C	-3.194373	3.497378	0.279733	3.246443	3.419182	-0.640539	-3.208835	3.495135	0.286036	-3.268337	-3.412260	-0.650876
30	H	-2.769711	3.536802	1.288059	4.138670	4.044698	-0.654181	-2.782622	3.535934	1.293668	-2.590438	-3.766849	0.132324
31	H	-4.072412	4.140743	0.228178	2.566359	3.767787	0.143453	-4.088506	4.136382	0.235721	-2.770681	-3.460209	-1.624885
32	H	-2.451261	3.831640	-0.451516	2.749134	3.467927	-1.614669	-2.467700	3.830982	-0.446503	-4.163642	-4.033319	-0.667122
33	O	1.062996	1.310204	1.542483	-1.147035	1.341872	-1.561314	1.056474	1.319922	1.534674	1.137605	-1.362749	-1.547499
34	H	1.924281	1.758478	1.441189	-1.988176	1.802852	-1.379772	1.934027	1.737861	1.456067	1.997351	-1.793459	-1.385878
35	C	1.486372	1.077748	-0.867171	-1.371548	1.067041	0.870384	1.472449	1.080115	-0.880617	1.355360	-1.065780	0.887054
36	H	1.333911	0.403513	-1.713976	-1.160906	0.373047	1.688186	1.307457	0.402937	-1.722429	1.131986	-0.364144	1.694644
37	H	0.864588	1.958767	-1.047355	-0.719872	1.933044	1.013837	0.859975	1.968238	-1.055641	0.713979	-1.939003	1.030872
38	C	2.947261	1.564239	-0.889506	-2.815360	1.579208	1.026531	2.939421	1.535381	-0.918022	2.805040	-1.543721	1.060720
39	C	4.051826	0.521261	-1.028562	-3.924459	0.554119	1.240991	3.956334	0.411772	-1.120520	3.821785	-0.432025	1.321490
40	O	3.757497	-0.447559	-1.902810	-3.575942	-0.435998	2.070315	5.129084	0.684786	-0.518710	5.037787	-0.742886	0.835063

41	H	4.535293	-1.023743	-2.013927	-4.352073	-1.000938	2.236525	5.775505	-0.007326	-0.745028	5.674942	-0.056099	1.100499
42	O	5.126561	0.632987	-0.466197	-5.040542	0.695574	0.773982	3.772426	-0.557758	-1.828805	3.594879	0.561315	1.982740
43	O	3.222682	2.388113	0.240749	-3.169862	2.430282	-0.060634	3.216122	2.352017	0.219230	3.162005	-2.394461	-0.028052
44	H	4.165160	2.276897	0.457334	-4.129544	2.341084	-0.197141	4.173670	2.465412	0.312047	4.121143	-2.529993	-0.033395
45	H	3.043101	2.166916	-1.807566	-2.820062	2.164973	1.960417	3.045698	2.141366	-1.832610	2.821448	-2.126942	1.995958

Table S12: Energies and Boltzmann distribution of compound **19** (4*R*,12*S*).

Conformer	Hartrees	kcal/mol	ΔG (kcal/mol)	Boltzmann Distribution (%)
Conformer 17	-1261.447001	-791570.1131	0	47.97333957
Conformer 7	-1261.446264	-791569.6507	0.462474581	20.46337051
Conformer 5	-1261.446188	-791569.603	0.510165311	18.74218140
Conformer 22	-1261.445123	-791568.9347	1.178463044	5.47166678
Conformer 19	-1261.444746	-791568.6981	1.415034166	3.53867507
Conformer 6	-1261.444194	-791568.3517	1.76141947	1.86939100
Conformer 8	-1261.443984	-791568.22	1.893196487	1.46644658
Conformer 2	-1261.441753	-791566.82	3.293170423	0.11121326
Conformer 1	-1261.441673	-791566.7698	3.343371192	0.10138910
Conformer 13	-1261.441135	-791566.4322	3.680971361	0.05443518
Conformer 14	-1261.440806	-791566.2257	3.887422022	0.03721345
Conformer 4	-1261.440619	-791566.1084	4.004766318	0.02997875
Conformer 15	-1261.440499	-791566.0331	4.080067471	0.02609550
Conformer 16	-1261.440369	-791565.9515	4.161643721	0.02245417
Conformer 24	-1261.440151	-791565.8147	4.298440815	0.01745205
Conformer 9	-1261.440135	-791565.8047	4.308480969	0.01713221
Conformer 3	-1261.439962	-791565.6961	4.417040131	0.01402672
Conformer 10	-1261.439917	-791565.6679	4.445278063	0.01331567
Conformer 25	-1261.439722	-791565.5455	4.567642437	0.01062821
Conformer 20	-1261.439444	-791565.3711	4.742090108	0.00770701
Conformer 18	-1261.439414	-791565.3522	4.760915396	0.00744430
Conformer 11	-1261.43841	-791564.7222	5.390935043	0.00233211
Conformer 12	-1261.438324	-791564.6682	5.444900869	0.00211141

Table S13: XYZ coordinates of compound **19** (4*R*,12*S*).

		**Conformer 1**			**Conformer 2**			**Conformer 3**			**Conformer 4**		
Row	Symbol	X	Y	Z	X	Y	Z	X	Y	Z	X	Y	Z
1	C	-1.709110	1.557052	0.140168	1.795584	-1.295230	0.523560	1.759530	-1.323698	0.558442	-1.659749	1.596097	-1.709110
2	C	-0.950306	-0.774880	-1.198907	0.985252	0.897397	-1.007406	0.964568	0.828919	-1.036567	-0.933378	-0.710376	-0.950306
3	C	-0.329026	1.293838	-0.096231	0.434118	-1.177634	0.120535	0.396913	-1.203168	0.159965	-0.282988	1.315582	-0.329026
4	C	-2.714172	0.680865	-0.293615	2.756674	-0.337171	0.170654	2.729150	-0.388250	0.169702	-2.677116	0.749985	-2.714172
5	C	-2.308151	-0.486301	-0.967686	2.327776	0.751637	-0.611753	2.307812	0.679965	-0.644279	-2.287251	-0.404422	-2.308151
6	C	0.032018	0.105712	-0.761526	0.044802	-0.057668	-0.640129	0.015856	-0.103142	-0.633405	0.060976	0.140498	0.032018
7	H	-0.665487	-1.672758	-1.729510	0.682457	1.739979	-1.613387	0.666860	1.655856	-1.666138	-0.659914	-1.598057	-0.665487
8	C	0.687189	2.252668	0.334042	-0.531989	-2.214726	0.478937	-0.577510	-2.220123	0.552218	0.747156	2.247224	0.687189
9	C	2.079609	1.996575	0.014334	-1.893328	-2.111863	-0.014038	-1.939958	-2.118048	0.061458	2.135909	1.978835	2.079609
10	H	2.788453	2.763373	0.301198	-2.556281	-2.933778	0.226424	-2.609216	-2.927485	0.325602	2.855689	2.728953	2.788453
11	C	2.452566	0.866867	-0.629958	-2.290342	-1.051561	-0.754601	-2.330071	-1.074151	-0.705491	2.492144	0.859285	2.452566
12	C	1.492260	-0.247736	-1.026769	-1.400039	0.138859	-1.088480	-1.430916	0.100789	-1.070735	1.516303	-0.236040	1.492260
13	O	-3.312887	-1.306631	-1.374570	3.294493	1.645265	-0.950635	3.282400	1.551849	-1.016455	-3.303619	-1.195583	-3.312887
14	C	-4.172518	0.980975	-0.033044	4.198610	-0.487171	0.598700	4.171781	-0.541660	0.594192	-4.131802	1.067445	-4.172518
15	H	-4.310023	2.065861	-0.014335	4.625398	0.505946	0.766829	4.611013	0.451135	0.728794	-4.250015	2.153312	-4.310023
16	H	-4.770066	0.590446	-0.861872	4.228493	-1.020939	1.552822	4.200784	-1.045430	1.564511	-4.729954	0.716631	-4.770066
17	C	-4.690729	0.383116	1.289953	5.063796	-1.241876	-0.429964	5.022483	-1.338289	-0.414807	-4.670794	0.433963	-4.690729
18	H	-4.133199	0.778958	2.145171	5.077566	-0.719080	-1.391764	5.035982	-0.846773	-1.392964	-4.111129	0.787797	-4.133199
19	H	-4.591934	-0.707243	1.294846	4.680907	-2.253667	-0.598644	4.627917	-2.350806	-0.548737	-4.593721	-0.657645	-4.591934
20	H	-5.748889	0.628267	1.432883	6.096667	-1.325295	-0.074328	6.056537	-1.421487	-0.062605	-5.724849	0.695171	-5.748889
21	O	-2.083145	2.679797	0.794122	2.194003	-2.352568	1.266332	2.150906	-2.361994	1.330480	-2.017936	2.707036	-2.083145
22	H	-1.236762	3.169239	1.006397	1.376193	-2.910071	1.412122	1.328337	-2.906018	1.498211	-1.164763	3.175859	-1.236762
23	O	0.372750	3.306945	0.951022	-0.196752	-3.206556	1.182353	-0.248670	-3.194829	1.281920	0.448005	3.289951	0.372750
24	O	3.701581	0.556703	-1.009749	-3.507065	-0.883693	-1.294758	-3.547244	-0.909693	-1.245654	3.735906	0.540023	3.701581
25	C	4.765963	1.498171	-0.774746	-4.495872	-1.919827	-1.142220	-4.544627	-1.932419	-1.062397	4.814440	1.459807	4.765963
26	H	4.892715	1.666108	0.298238	-4.132506	-2.855591	-1.575686	-4.784849	-2.040802	-0.001258	4.945176	1.600069	4.892715
27	H	4.556010	2.441854	-1.285698	-5.371297	-1.567691	-1.684842	-4.191041	-2.882678	-1.471791	4.617753	2.418421	4.556010
28	H	5.659396	1.035421	-1.190059	-4.738444	-2.058701	-0.085143	-5.418728	-1.587194	-1.611553	5.700319	0.994016	5.659396
29	C	-2.999845	-2.507951	-2.089922	2.951804	2.794580	-1.734565	2.948403	2.677748	-1.837096	-3.006625	-2.381517	-2.999845
30	H	-2.478916	-2.284269	-3.026494	2.223215	3.424263	-1.213855	2.228666	3.332509	-1.335287	-2.475171	-2.140927	-2.478916
31	H	-3.960293	-2.973823	-2.308837	2.560087	2.500467	-2.713723	2.549277	2.355171	-2.804206	-3.973459	-2.824097	-3.960293
32	H	-2.395366	-3.185133	-1.477890	3.883064	3.345236	-1.864733	3.884775	3.213853	-1.989482	-2.418279	-3.086364	-2.395366
33	O	1.594754	-0.444075	-2.451344	-1.349727	0.276612	-2.522494	-1.399626	0.217771	-2.506012	1.630868	-0.432605	1.594754
34	H	2.528732	-0.596212	-2.666233	-2.261674	0.325975	-2.850405	-2.315619	0.270884	-2.821692	2.565035	-0.596453	2.528732
35	C	1.924587	-1.580145	-0.341595	-2.035577	1.443945	-0.518280	-2.042393	1.420234	-0.506392	1.915140	-1.575943	1.924587
36	H	1.246482	-2.367587	-0.682945	-1.392775	2.281924	-0.803288	-1.423570	2.249383	-0.860504	1.255238	-2.357040	1.246482
37	H	2.924464	-1.821292	-0.713757	-2.996806	1.578197	-1.022754	-3.034661	1.531591	-0.953863	2.934125	-1.812487	2.924464
38	C	2.045834	-1.636943	1.196644	-2.359035	1.534516	0.988591	-2.262352	1.538477	1.014895	1.946658	-1.626949	2.045834
39	C	0.693017	-1.717792	1.907143	-1.120272	1.741785	1.862892	-0.984783	1.779499	1.824387	0.566826	-1.698750	0.693017
40	O	0.357297	-0.586044	2.539231	-0.787945	0.657385	2.575176	-0.453335	2.989050	1.558004	-0.050592	-2.864950	0.357297
41	H	-0.517623	-0.700753	2.953621	0.014719	0.850835	3.093450	0.331837	3.121940	2.118062	-0.903561	-2.891596	-0.517623
42	O	0.014045	-2.728476	1.916572	-0.524556	2.801957	1.921199	-0.518773	1.007591	2.638156	0.091569	-0.841795	0.014045
43	O	2.801538	-2.786655	1.573972	-3.242458	2.630105	1.221583	-3.193505	2.584598	1.301957	2.727935	-2.738554	2.801538
44	H	2.250282	-3.571809	1.425728	-2.739499	3.452677	1.109358	-2.829546	3.426198	0.987013	2.318678	-3.558709	2.250282
45	H	2.599457	-0.775694	1.575788	-2.891187	0.643312	1.326913	-2.715284	0.629301	1.414177	2.440619	-0.743373	2.599457
		**Conformer 5**			**Conformer 6**			**Conformer 7**			**Conformer 8**		
Row	Symbol	X	Y	Z	X	Y	Z	X	Y	Z	X	Y	Z
1	C	0.177800	-2.066464	-1.101391	-0.777563	-2.047952	-1.092325	-0.802330	-1.993361	-1.412444	-0.524690	-1.985765	-1.403846
2	C	-1.222936	-1.109215	0.850664	0.979055	-1.111500	0.819932	1.008516	-1.133741	0.599205	1.215652	-1.136362	0.569416
3	C	-0.058600	-0.732253	-1.186286	-0.283481	-0.717418	-1.184723	-0.299647	-0.619983	-1.338655	-0.150359	-0.614119	-1.336833
4	C	-0.286312	-2.926350	-0.057352	-0.408475	-2.914329	-0.060249	-0.415379	-2.942603	-0.504659	-0.034330	-2.938432	-0.508726
5	C	-0.991076	-2.426659	0.906529	0.487826	-2.425203	0.883158	0.508265	-2.487001	0.497235	0.843672	-2.487822	0.474212
6	C	-0.755316	-0.267554	-0.186335	0.593367	-0.262959	-0.204283	0.604398	-0.206905	-0.310307	0.719490	-0.206168	-0.327811

7	H	-1.776129	-0.749794	1.600795	1.669633	-0.759575	1.554923	1.718925	-0.809252	1.373648	1.896647	-0.815803	1.328929
8	C	0.397890	0.114382	-2.326937	-0.638563	0.136211	-2.313492	-0.675581	0.326962	-2.353324	-0.616762	0.336390	-2.338996
9	C	0.071155	1.427036	-2.443931	-0.037348	1.444723	-2.439920	-0.067685	1.691722	-2.318116	-0.132062	1.698750	-2.313309
10	H	0.374451	1.992252	-3.334692	-0.281767	2.015076	-3.322778	-0.328400	2.338769	-3.125225	-0.452179	2.348493	-3.111705
11	C	-0.599652	1.911273	-1.477161	0.776476	1.919271	-1.490341	0.772144	2.122330	-1.317887	0.671431	2.123859	-1.332016
12	C	-1.012527	1.165208	-0.193132	1.113986	1.166419	-0.217004	1.134636	1.257571	-0.143355	1.108704	1.255638	-0.169910
13	O	-1.426486	-3.299634	1.888793	0.836981	-3.304461	1.853612	0.874088	-3.440654	1.351924	1.300687	-3.444523	1.317442
14	C	-0.025746	-4.341110	0.007245	-0.936994	-4.324591	0.013470	-0.954700	-4.394479	-0.601177	-0.443456	-4.388632	-0.598724
15	H	0.029444	-4.634909	1.055655	-1.042349	-4.620078	1.063423	-1.037533	-4.632071	-1.648443	-0.650543	-4.624256	-1.642007
16	H	-0.871804	-4.364107	-0.439745	-1.934995	-4.337594	-0.409407	-1.963347	-5.022556	-0.283254	0.393878	-5.020160	-0.298157
17	C	1.272126	-5.361221	-0.716473	-0.036008	-5.351132	-0.733839	-0.080690	-4.735447	0.249266	-1.683055	-4.726401	0.275669
18	H	2.144091	-5.384362	-0.275506	0.965805	-5.384672	-0.316873	0.931047	-4.143346	-0.063887	-2.549225	-4.131430	-0.019793
19	H	1.239032	-5.111713	-1.777722	0.065450	-5.100039	-1.796661	-0.002156	-4.535072	1.309944	-1.499983	-4.527508	1.332685
20	H	1.416995	-6.368128	-0.644189	-0.461723	-6.354212	-0.653646	-0.513949	-5.795031	0.143914	-1.940875	-5.785089	0.174586
21	O	0.859537	-2.537000	-2.045318	-1.623466	-2.508166	-2.016959	-1.675042	-2.414484	-2.382370	-1.367258	-2.401970	-2.355124
22	H	1.089809	-1.786919	-2.695940	-1.752385	-1.754969	-2.662038	-1.812967	-1.605023	-2.932486	-1.578247	-1.590814	-2.899149
23	O	1.041226	-0.295012	-3.219415	-1.431983	-0.263760	-3.187958	-1.493768	-0.036856	-3.270925	-1.403566	-0.022726	-3.238118
24	O	-0.988554	3.094666	-1.501458	1.405415	3.098746	-1.524790	1.407396	3.349582	-1.209262	1.199218	3.348706	-1.234219
25	C	-0.733128	3.959304	-2.639996	1.238590	3.968636	-2.656129	1.220875	4.326308	-2.231589	0.929050	4.329496	-2.246163
26	H	0.343337	4.248532	-2.743416	0.189247	4.265319	-2.735222	0.171495	4.537250	-2.278613	-0.142757	4.542819	-2.266758
27	H	-1.220997	3.461386	-3.548980	1.587448	3.471773	-3.574431	1.546068	3.970104	-3.200651	1.289571	3.976256	-3.224930
28	H	-1.161032	4.835093	-2.432521	1.850955	4.839632	-2.459272	1.843485	5.219378	-1.932383	1.474855	5.220468	-1.956919
29	C	-2.173527	-2.874047	2.929798	1.724355	-2.889506	2.874781	1.789250	-3.077200	2.391239	2.217137	-3.085912	2.338085
30	H	-3.099940	-2.037589	3.493326	1.298356	-2.613604	2.446829	2.758465	-2.663391	1.974775	3.141222	-4.013799	2.861950
31	H	-2.411849	-2.592316	2.524018	2.701413	-3.755478	3.524933	1.911041	-4.003563	2.920855	2.438058	-2.366777	3.039193
32	H	-1.577063	-3.736636	3.586060	1.837093	-2.052074	3.451367	1.383221	-2.359025	3.082417	1.764220	-2.675293	1.903106
33	O	-2.434651	1.080866	-0.087965	2.548154	1.071083	-0.147318	2.570332	1.294077	-0.068001	2.546319	1.280941	-0.136829
34	H	-2.639645	1.978670	-0.176596	2.905349	1.967526	-0.232035	2.931667	2.225140	-0.054410	2.819086	2.209753	-0.121989
35	C	-0.320210	1.951770	1.052950	0.599227	1.950418	1.044559	0.656615	1.831311	1.198755	0.554622	1.832741	1.187460
36	H	-0.707651	1.439952	1.925087	1.012658	1.434978	1.907332	1.081325	1.248635	1.993214	1.026709	1.247045	1.972058
37	H	-0.641079	2.962320	1.022036	1.019438	2.958515	1.009996	1.083129	2.869715	1.296202	0.887617	2.867296	1.277385
38	C	1.220312	2.058493	1.210762	-0.929785	2.070231	1.248754	-0.855604	1.788691	1.380860	-0.975420	1.809533	1.422193
39	C	1.881018	2.570428	2.603948	-1.284000	2.389988	2.715876	-1.174765	2.044936	2.839710	-1.342282	1.894317	2.922956
40	O	1.606415	1.733464	3.581996	-0.921071	3.285254	2.842057	-2.169167	1.088772	3.667949	-0.907749	2.671006	3.181751
41	H	2.074978	2.120405	4.441221	-1.168328	3.400590	3.789233	-2.364546	1.313273	4.579785	-1.166812	2.635962	4.137185
42	O	2.598571	3.636370	2.789873	-1.844440	1.854237	3.664670	-0.633780	3.025893	3.197625	-1.970275	1.283491	3.782906
43	O	1.665170	2.917275	0.242945	-1.510345	3.041062	0.348558	-1.366248	2.738944	0.564599	-1.640661	2.858124	0.675057
44	H	1.349107	3.700172	0.717118	-1.840701	3.087252	0.439847	-2.328451	3.421426	1.156854	-2.001581	2.817522	0.778877
45	H	1.628507	1.061597	1.110987	-1.378553	1.091830	1.070162	-1.328917	0.787769	1.126874	-1.347440	0.834266	1.110590
		**Conformer 9**			**Conformer 10**			**Conformer 11**			**Conformer 12**		
Row	Symbol	X	Y	Z	X	Y	Z	X	Y	Z	X	Y	Z
1	C	1.839241	1.365340	-0.121514	-1.820607	1.362844	-0.497265	-1.797811	1.370464	0.057051	1.849210	-1.346094	-0.523651
2	C	1.347062	-1.334272	0.414818	-1.338121	-1.390565	-0.440193	-1.339427	-1.356691	-0.372834	1.370326	1.404517	-0.382812
3	C	0.530683	0.944157	0.253919	-0.489018	0.873428	-0.632042	-0.491048	0.917032	-0.292681	0.520465	-0.850397	-0.656489
4	C	2.903556	0.460000	-0.229789	-2.913289	0.498218	-0.346491	-2.873879	0.482761	0.196124	2.942024	-0.488714	-0.335163
5	C	2.634468	-0.891469	0.061402	-2.645542	-0.884467	-0.327055	-2.623104	-0.882597	-0.042655	2.676151	0.892886	-0.273700
6	C	0.301712	-0.424352	0.498023	-0.268008	-0.516898	-0.578280	-0.281982	-0.464397	-0.492028	0.300469	0.537276	-0.560403
7	H	1.161646	-2.378474	0.624058	-1.156003	-2.455851	-0.417106	-1.167076	-2.410233	-0.542487	1.189741	2.469067	-0.329695
8	C	-0.534292	1.927125	0.439186	0.614270	1.798893	-0.878583	0.583894	1.881860	-0.535546	-0.584946	-1.767188	-0.924010
9	C	-1.813578	1.487087	0.969828	1.935057	1.261297	-1.157809	1.856064	1.402488	-1.043496	-1.903375	-1.220657	-1.193832
10	H	-2.536988	2.262475	1.190256	2.698600	1.977232	-1.436190	2.590706	2.157628	-1.294303	-2.667657	-1.927876	-1.491810
11	C	-2.058321	0.176532	1.200765	2.171624	-0.069737	-1.101297	2.087874	0.078833	-1.210170	-2.134466	0.111136	-1.119164
12	C	-1.091678	-0.941345	0.834289	1.140999	-1.093199	-0.644723	1.107903	-1.011204	-0.795647	-1.107047	1.117974	-0.622690
13	O	3.700952	-1.731061	-0.028783	-3.734676	-1.687977	-0.190225	-3.699373	-1.702210	0.075475	3.765362	1.689973	-0.101164
14	C	4.288254	0.928819	-0.615041	-4.318635	1.037241	-0.205843	-4.253366	0.984244	0.557411	4.344971	-1.034517	-0.196469
15	H	4.783119	0.142275	-1.192375	-4.389984	1.981378	-0.753461	-4.759311	0.227721	1.164367	4.419607	-1.963476	-0.769000

16	H	4.194865	1.801757	-1.267439	-5.017615	0.336576	-0.671806	-4.150503	1.881685	1.173971	5.049760	-0.322977	-0.636627
17	C	5.167681	1.295599	0.596677	-4.737845	1.270415	1.259098	-5.123671	1.311874	-0.671998	4.750267	-1.308666	1.265317
18	H	5.302977	0.434880	1.259628	-4.074949	1.992338	1.747314	-5.268346	0.426220	-1.299004	4.081739	-2.042516	1.727405
19	H	4.716214	2.105430	1.179177	-4.703921	0.337686	1.831476	-4.659947	2.091096	-1.285640	4.712395	-0.392001	1.862862
20	H	6.157957	1.627781	0.265823	-5.760074	1.661643	1.308411	-6.110189	1.670134	-0.358023	5.771405	-1.702948	1.313418
21	O	2.083947	2.671566	-0.375199	-2.059367	2.694275	-0.526976	-2.026778	2.686970	0.254827	2.085873	-2.676928	-0.590270
22	H	1.214752	3.146088	-0.236764	-1.169625	3.130190	-0.662130	-1.154405	3.147440	0.090006	1.195139	-3.106795	-0.739126
23	O	-0.349215	3.150501	0.192700	0.432239	3.047182	-0.899807	0.406661	3.115865	-0.350478	-0.405234	-3.015584	-0.973149
24	O	-3.169929	-0.331110	1.753817	3.326984	-0.677610	-1.408990	3.198400	-0.466399	-1.722620	-3.279478	0.731102	-1.435077
25	C	-4.198251	0.562503	2.218806	4.429228	0.107962	-1.898807	4.270813	0.390612	-2.158915	-4.396566	-0.045984	-1.904548
26	H	-4.612917	1.133620	1.384043	4.141724	0.632090	-2.814423	4.663335	0.960261	-1.312572	-4.721745	-0.742014	-1.127122
27	H	-3.794933	1.238785	2.977582	5.225100	-0.605050	-2.106381	3.920106	1.064336	-2.945415	-4.124968	-0.587146	-2.815170
28	H	-4.965000	-0.076959	2.652490	4.753791	0.823246	-1.138471	5.035785	-0.278084	-2.549341	-5.183993	0.675242	-2.116195
29	C	3.522613	-3.128648	0.228852	-3.564914	-3.110035	-0.171242	-3.541614	-3.111964	-0.129323	3.597251	3.110822	-0.039168
30	H	2.813345	-3.572645	-0.477168	-2.949462	-3.422163	0.678787	-2.841494	-3.538967	0.595704	2.973347	3.397187	0.813794
31	H	3.183666	-3.299723	1.255780	-3.119440	-3.466679	-1.105624	-3.202729	-3.325590	-1.148074	3.161883	3.497521	-0.966356
32	H	4.505729	-3.577717	0.089647	-4.568545	-3.521193	-0.065182	-4.532057	-3.539637	0.023430	4.600223	3.516973	0.090128
33	O	-0.937793	-1.799762	1.979733	1.097042	-2.154801	-1.616169	0.952957	-1.928326	-1.890893	-1.050100	2.212381	-1.555697
34	H	-1.824154	-2.047122	2.287147	2.006073	-2.459753	-1.764454	1.837653	-2.211401	-2.171093	-1.957838	2.517557	-1.711153
35	C	-1.689454	-1.826275	-0.310496	1.582271	-1.743354	0.709278	1.688585	-1.830378	0.406768	-1.564887	1.725272	0.747932
36	H	-1.023846	-2.689253	-0.398435	0.889471	-2.571232	0.882961	1.033867	-2.697741	0.524398	-0.848536	2.516816	0.981649
37	H	-2.658585	-2.199585	0.034636	2.574544	-2.179026	0.556937	2.674031	-2.198507	0.105614	-2.536591	2.199492	0.584001
38	C	-1.879391	-1.279660	-1.741117	1.621533	-0.931787	2.020890	1.836618	-1.199006	1.812222	-1.692400	0.850454	2.005760
39	C	-2.846420	-0.109889	-1.902053	2.586502	0.249870	2.066288	2.863918	-0.061596	1.862249	-2.834099	-0.171491	1.948026
40	O	-4.057263	-0.354905	-1.387860	3.841838	-0.083621	1.744392	2.375942	1.082267	2.366851	-2.475429	-1.379105	2.418190
41	H	-4.638961	0.404886	-1.570338	4.413824	0.697975	1.848998	3.088905	1.746246	2.367191	-3.260054	-1.956278	2.424963
42	O	-2.552641	0.910084	-2.500295	2.247752	1.362887	2.428292	4.019416	-0.198654	1.504463	-3.966738	0.102333	1.599467
43	O	-0.647336	-0.958926	-2.358506	0.333498	-0.502371	2.419134	0.614634	-0.892270	2.458408	-0.436743	0.284597	2.334594
44	H	-0.735395	-0.070859	-2.744666	0.397720	0.439266	2.653016	0.160389	-0.178869	1.986524	-0.538661	-0.276267	3.116653
45	H	-2.352570	-2.104257	-2.297269	2.013250	-1.633145	2.774576	2.287813	-1.985706	2.427300	-2.015399	1.540507	2.802613
		**Conformer 13**			**Conformer 14**			**Conformer 15**			**Conformer 16**		
Row	Symbol	X	Y	Z	X	Y	Z	X	Y	Z	X	Y	Z
1	C	-2.490649	0.852233	0.562059	-2.477098	1.081377	0.231239	-2.591245	-1.059654	-0.418645	2.595288	-1.158174	0.070346
2	C	-0.794375	-1.021167	-0.634577	-0.820503	-0.882272	-0.872554	-1.105498	1.212366	0.257027	1.118992	1.148685	-0.498005
3	C	-1.133639	1.192545	0.296177	-1.079296	1.322155	0.104459	-1.185073	-1.166152	-0.211293	1.173793	-1.226214	-0.008684
4	C	-3.012275	-0.410884	0.244235	-3.061241	-0.123351	-0.188820	-3.265879	0.161542	-0.285480	3.289821	0.039319	-0.148560
5	C	-2.145684	-1.327854	-0.377762	-2.209451	-1.089669	-0.754180	-2.499281	1.287597	0.072145	2.525345	1.185611	-0.440590
6	C	-0.287279	0.222978	-0.281995	-0.257002	0.306661	-0.427624	-0.455289	-0.006354	0.116110	0.450301	-0.049250	-0.284174
7	H	-0.151874	-1.731023	-1.134700	-0.187783	-1.626833	-1.333204	-0.534673	2.092565	0.518203	0.551697	2.043028	-0.715196
8	C	-0.667241	2.564594	0.498783	-0.532700	2.647407	0.397475	-0.530941	-2.471279	-0.251651	0.489879	-2.511525	0.106265
9	C	0.642156	2.939064	0.002124	0.842416	2.932106	0.037747	0.856807	-2.566798	0.173965	-0.932207	-2.571309	-0.193613
10	H	0.894214	3.991120	0.053120	1.166700	3.959574	0.147439	1.260001	-3.566069	0.280220	-1.370820	-3.559647	-0.251592
11	C	1.488051	2.009580	-0.508853	1.661206	1.953710	-0.423854	1.574958	-1.460185	0.463794	-1.642027	-1.447327	-0.433263
12	C	1.204804	0.506198	-0.447716	1.261282	0.475752	-0.439715	1.071129	-0.041418	0.218478	-1.079533	-0.041338	-0.250548
13	O	-2.700447	-2.524795	-0.708260	-2.816971	-2.231392	-1.175217	-3.196119	2.445127	0.216586	3.237588	2.322318	-0.657746
14	C	-4.459506	-0.748508	0.521891	-4.547503	-0.359104	-0.045754	-4.760578	0.253482	-0.492243	4.798404	0.091316	-0.067871
15	H	-4.535574	-1.810846	0.772278	-5.064279	0.602650	-0.111020	-4.999734	1.225362	-0.934189	5.199440	-0.886321	-0.349909
16	H	-4.790549	-0.183295	1.397762	-4.893600	-0.972598	-0.882719	-5.063899	-0.514020	-1.209814	5.168901	0.818141	-0.796734
17	C	-5.396588	-0.440241	-0.662584	-4.929549	-1.049283	1.278647	-5.568342	0.077743	0.808840	5.321554	0.468013	1.332295
18	H	-5.108513	-1.011761	-1.550767	-4.624025	-0.445310	2.139426	-5.307449	0.848708	1.540907	4.994425	-0.258632	2.083276
19	H	-5.368659	0.623810	-0.919290	-4.449443	-2.029545	1.364675	-5.376280	-0.900257	1.262248	4.959328	1.455816	1.635038
20	H	-6.430747	-0.701207	-0.411929	-6.013706	-1.196554	1.335959	-6.642425	0.151941	0.606097	6.416848	0.491848	1.338715
21	O	-3.318572	1.761585	1.123621	-3.282757	2.034437	0.751000	-3.311707	-2.156209	-0.745234	3.310951	-2.270823	0.349656
22	H	-2.771555	2.591902	1.243546	-2.689666	2.818175	0.944256	-2.662741	-2.914753	-0.780957	2.647045	-3.010942	0.446613
23	O	-1.408106	3.432703	1.040361	-1.254776	3.555631	0.897084	-1.158129	-3.513126	-0.581576	1.115062	-3.567685	0.391648
24	O	2.700238	2.252154	-1.016204	2.931057	2.115027	-0.807164	2.823889	-1.453718	0.974953	-2.929770	-1.410112	-0.834313

25	C	3.181592	3.607891	-1.063785	3.514981	3.429917	-0.760448	3.422071	-2.702375	1.392440	-3.595148	-2.645776	-1.182307
26	H	3.261281	4.019307	-0.053461	3.529495	3.803568	0.267486	2.776394	-3.195957	2.122429	-3.700706	-3.280233	-0.298579
27	H	2.516602	4.226055	-1.673293	2.958627	4.116060	-1.405066	4.371633	-2.428075	1.848175	-3.030598	-3.162888	-1.961719
28	H	4.166425	3.550491	-1.524236	4.531797	3.309538	-1.130299	3.589589	-3.349472	0.527617	-4.574416	-2.350666	-1.554501
29	C	-1.889787	-3.535750	-1.318692	-2.035138	-3.264994	-1.785361	-2.500747	3.652560	0.550627	2.549280	3.539057	-0.972321
30	H	-1.499148	-3.198514	-2.284250	-1.545848	-2.904252	-2.695872	-1.768074	3.911533	-0.220477	1.891353	3.842382	-0.151550
31	H	-2.552821	-4.387040	-1.471083	-2.743393	-4.053253	-2.039412	-2.005357	3.565064	1.522940	1.971623	3.437198	-1.896674
32	H	-1.063362	-3.827460	-0.662369	-1.286301	-3.655876	-1.088801	-3.267704	4.425025	0.599904	3.329868	4.286814	-1.109779
33	O	1.649214	-0.135552	-1.632882	1.770851	-0.168915	-1.596593	1.454751	0.807295	1.305722	-1.534417	0.807270	-1.310183
34	H	2.579923	-0.409885	-1.522835	2.660232	-0.523179	-1.404530	2.330971	0.533836	1.619999	-2.438863	0.549484	-1.548571
35	C	1.994787	0.012159	0.820679	1.883778	-0.109711	0.881498	1.607759	0.536853	-1.141140	-1.479339	0.565016	1.143456
36	H	2.971647	0.503622	0.834001	2.892238	0.296781	0.998340	1.317411	-0.142245	-1.948687	-1.143704	-0.116866	1.930889
37	H	1.450365	0.343763	1.707646	1.287323	0.249095	1.723228	1.072730	1.476587	-1.302988	-0.902569	1.487827	1.249742
38	C	2.186518	-1.508271	0.959716	1.935009	-1.643067	0.995439	3.114721	0.839345	-1.321838	-2.955278	0.918017	1.446479
39	C	3.476456	-1.975978	0.279928	3.239041	-2.207079	0.424517	3.646168	1.853762	-0.305145	-3.541420	1.934288	0.462204
40	O	4.068944	-2.984586	0.925158	3.673591	-3.283101	1.086710	3.178967	3.090689	-0.542677	-3.014362	3.157881	0.633184
41	H	4.855999	-3.269169	0.425836	4.477161	-3.625264	0.654459	3.519686	3.693100	0.142750	-3.395567	3.760185	-0.030682
42	O	3.904137	-1.510305	-0.765904	3.806707	-1.754667	-0.558553	4.410835	1.585522	0.602719	-4.393619	1.678317	-0.367966
43	O	2.166909	-1.809904	2.348889	1.761075	-1.969371	2.368421	3.948632	-0.299843	-1.375721	-3.815930	-0.193331	1.590527
44	H	2.318760	-2.758537	2.471245	1.821734	-2.929814	2.477309	3.998941	-0.680694	-0.484483	-3.964058	-0.578248	0.712021
45	H	1.375003	-2.043271	0.446785	1.133136	-2.097482	0.396590	3.197583	1.315703	-2.304027	-2.939473	1.410581	2.424084
		**Conformer 17**			**Conformer 18**			**Conformer 19**			**Conformer 20**		
Row	Symbol	X	Y	Z	X	Y	Z	X	Y	Z	X	Y	Z
1	C	-2.556768	1.087936	0.373781	-2.462619	-1.161397	-0.512247	-2.543006	0.913123	0.675303	-2.479086	-1.276835	-0.178292
2	C	-1.203173	-1.006230	-0.892376	-1.422215	1.263996	0.413519	-1.195875	-1.164404	-0.624546	-1.443192	1.184590	0.651875
3	C	-1.164337	1.208053	0.096872	-1.089912	-1.075240	-0.138025	-1.187344	1.088564	0.273855	-1.084134	-1.146733	0.084518
4	C	-3.284470	-0.056450	0.017344	-3.321881	-0.057297	-0.426793	-3.235829	-0.281786	0.434301	-3.363913	-0.201495	-0.018844
5	C	-2.582398	-1.094343	-0.624899	-2.779117	1.148654	0.057051	-2.541122	-1.307411	-0.235098	-2.820084	1.026075	0.405821
6	C	-0.500088	0.135263	-0.529751	-0.585184	0.159990	0.314241	-0.524904	0.024336	-0.369091	-0.582956	0.106175	0.489543
7	H	-0.679733	-1.812178	-1.387164	-1.025625	2.201874	0.776957	-0.676187	-1.963361	-1.134473	-1.047465	2.137272	0.975536
8	C	-0.459867	2.457056	0.383168	-0.245215	-2.268248	-0.155230	-0.526213	2.380548	0.451867	-0.207390	-2.313713	0.004712
9	C	0.905557	2.609845	-0.077830	1.098606	-2.182883	0.388832	0.782756	2.581712	-0.137122	1.173177	-2.183223	0.436237
10	H	1.344765	3.592559	0.041196	1.652235	-3.110941	0.458170	1.177995	3.589083	-0.094600	1.758921	-3.093562	0.464469
11	C	1.570841	1.582301	-0.663162	1.606972	-1.001048	0.803197	1.445852	1.565284	-0.743540	1.676998	-0.983419	0.801700
12	C	1.014841	0.158734	-0.733665	0.898355	0.334627	0.631276	0.963192	0.113867	-0.709123	0.915401	0.328773	0.683646
13	O	-3.327033	-2.180783	-0.964323	-3.652215	2.186590	0.147347	-3.256912	-2.439400	-0.472041	-3.713508	2.039532	0.556227
14	C	-4.762086	-0.164297	0.317298	-4.778358	-0.171761	-0.815343	-4.679422	-0.442846	0.853134	-4.840630	-0.360199	-0.300226
15	H	-5.196777	0.839315	0.309208	-5.107754	0.778533	-1.245911	-4.855633	-1.486247	1.130864	-5.127235	-1.396529	-0.100086
16	H	-5.246760	-0.736038	-0.479593	-4.875440	-0.933318	-1.594284	-4.857048	0.165913	1.744320	-5.405060	0.273329	0.390300
17	C	-5.061312	-0.833522	1.673287	-5.698325	-0.536733	0.366470	-5.681950	-0.035982	-0.244866	-5.226883	0.000638	-1.748223
18	H	-4.615020	-0.267700	2.497743	-5.646104	0.221376	1.154571	-5.548850	-0.645506	-1.144498	-4.700711	-0.637347	-2.465954
19	H	-4.662458	-1.852637	1.705764	-5.414939	-1.500303	0.802535	-5.553600	1.014820	-0.524580	-4.978015	1.042655	-1.973909
20	H	-6.142075	-0.886723	1.845052	-6.739693	-0.608769	0.033722	-6.711147	-0.169763	0.106248	-6.303397	-0.132048	-1.902622
21	O	-3.213841	2.099038	0.986096	-2.970226	-2.332803	-0.957908	-3.198224	1.917393	1.300946	-2.983598	-2.464550	-0.582271
22	H	-2.533840	2.819318	1.131248	-2.214132	-2.986964	-0.936509	-2.543869	2.671937	1.367094	-2.209759	-3.097335	-0.611601
23	O	-1.037948	3.413546	0.971492	-0.677727	-3.372046	-0.585697	-1.098613	3.330216	1.056416	-0.639411	-3.433665	-0.382252
24	O	2.801615	1.654603	-1.183247	2.807548	-0.819586	1.380659	2.617515	1.678820	-1.381708	2.914819	-0.761372	1.277400
25	C	3.496444	2.915456	-1.173209	3.637543	-1.965009	1.661409	3.235573	2.974743	-1.492148	3.797783	-1.879700	1.499686
26	H	3.670158	3.247875	-0.145689	3.921745	-2.461053	0.729672	3.487933	3.363881	-0.501611	4.019525	-2.378392	0.552488
27	H	2.924038	3.667616	-1.723038	3.107670	-2.659492	2.318303	2.569885	3.668229	-2.013240	3.343259	-2.580880	2.204205
28	H	4.445712	2.727235	-1.671820	4.518807	-1.568627	2.162229	4.141613	2.815047	-2.074194	4.705084	-1.452946	1.923056
29	C	-2.704255	-3.279101	-1.640725	-3.188822	3.462588	0.605173	-2.627732	-3.548222	-1.125094	-3.258186	3.323855	0.998686
30	H	-1.922077	-3.730782	-1.021893	-2.415071	3.862111	-0.058311	-2.296449	-3.275740	-2.132371	-2.547827	3.756590	0.286810
31	H	-2.284094	-2.963242	-2.601122	-2.806772	3.398478	1.629161	-3.393855	-4.320548	-1.189299	-2.800611	3.259369	1.991203
32	H	-3.499526	-4.004521	-1.810571	-4.061964	4.114121	0.581652	-1.779592	-3.920246	-0.541210	-4.150692	3.947107	1.047895
33	O	1.300043	-0.425782	-2.000466	0.988332	1.082630	1.852994	1.162059	-0.504868	-1.977171	1.080581	1.085739	1.892110

34	H	2.207651	-0.782724	-1.958745	1.870027	0.945724	2.231943	2.089164	-0.804354	-2.014456	1.993653	0.977611	2.199227
35	C	1.639636	-0.702782	0.418910	1.529202	1.149643	-0.545981	1.728907	-0.667226	0.414385	1.422535	1.156563	-0.543187
36	H	1.347059	-0.273617	1.381279	1.366670	0.612374	-1.484665	1.501819	-0.215195	1.382264	1.204852	0.607712	-1.463903
37	H	1.184552	-1.695904	0.343947	0.967924	2.085605	-0.596087	1.324918	-1.684859	0.420317	0.827014	2.072471	-0.552627
38	C	3.175646	-0.854141	0.386181	3.001699	1.540566	-0.424797	3.253489	-0.746329	0.263395	2.885404	1.599338	-0.542719
39	C	3.628014	-1.798594	1.493268	3.969621	0.474719	-0.954442	3.873981	-1.419139	1.492077	3.845516	0.561761	-1.138517
40	O	3.436278	-1.294780	2.716505	5.230555	0.738023	-0.556584	4.810705	-2.326602	1.174320	5.122606	0.859292	-0.825668
41	H	3.721458	-1.947325	3.381505	5.834069	0.096168	-0.972057	5.196648	-2.673889	1.998579	5.713944	0.235400	-1.283815
42	O	4.099982	-2.898473	1.267596	3.682635	-0.450251	-1.687888	3.583198	-1.124618	2.635962	3.535220	-0.369212	-1.854777
43	O	3.631279	-1.360140	-0.866947	3.182239	2.711179	-1.239392	3.544105	-1.430300	-0.954228	2.957815	2.764447	-1.382015
44	H	3.842062	-2.302921	-0.745910	4.045923	3.101892	-1.040322	4.490038	-1.370208	-1.149846	3.818169	3.190727	-1.254916
45	H	3.658535	0.113453	0.548005	3.271176	1.770318	0.611106	3.683434	0.262888	0.238599	3.228010	1.852392	0.465726

		**Conformer 22**			**Conformer 24**			**Conformer 25**		
Row	Symbol	X	Y	Z	X	Y	Z	X	Y	Z
1	C	-2.573492	1.070471	0.382320	-2.373002	1.059105	0.496645	-2.328923	1.290071	0.132696
2	C	-1.217470	-1.015339	-0.894652	-0.734690	-0.989277	-0.478070	-0.767377	-0.876907	-0.701429
3	C	-1.182618	1.196430	0.100096	-0.990435	1.309340	0.260130	-0.913266	1.427567	0.047897
4	C	-3.298430	-0.075665	0.026211	-2.949745	-0.195058	0.249703	-2.976725	0.093256	-0.206236
5	C	-2.595080	-1.109618	-0.621024	-2.110231	-1.202932	-0.259969	-2.171096	-0.978445	-0.633947
6	C	-0.517233	0.128074	-0.532706	-0.178683	0.254718	-0.209079	-0.144119	0.314033	-0.351846
7	H	-0.693241	-1.818289	-1.393483	-0.108596	-1.783514	-0.859131	-0.170600	-1.717451	-1.025966
8	C	-0.481055	2.446359	0.388795	-0.457833	2.665959	0.389864	-0.289494	2.734340	0.257430
9	C	0.882525	2.605114	-0.076264	0.888945	2.937559	-0.080295	1.118216	2.897386	-0.059258
10	H	1.318648	3.588971	0.044591	1.198759	3.975173	-0.094262	1.510967	3.905831	-0.020576
11	C	1.548777	1.582003	-0.667741	1.690343	1.930828	-0.499299	1.876306	1.831603	-0.406646
12	C	0.997002	0.157115	-0.743250	1.325441	0.456398	-0.352728	1.378198	0.391162	-0.328828
13	O	-3.336718	-2.198602	-0.958678	-2.715409	-2.392314	-0.517015	-2.838646	-2.113429	-0.970537
14	C	-4.774197	-0.190021	0.332846	-4.422982	-0.435731	0.488862	-4.480583	-0.030923	-0.116617
15	H	-5.213429	0.811650	0.326654	-4.565780	-1.468136	0.821671	-4.925826	0.953239	-0.287878
16	H	-5.259941	-0.763990	-0.461770	-4.758904	0.216954	1.299526	-4.831391	-0.691024	-0.915330
17	C	-5.064153	-0.860418	1.690275	-5.292640	-0.182347	-0.758437	-4.966900	-0.576848	1.240439
18	H	-4.616682	-0.292432	2.512581	-4.999627	-0.840834	-1.582418	-4.657277	0.078077	2.061550
19	H	-4.660372	-1.877649	1.721050	-5.197260	0.853344	-1.100729	-4.561044	-1.575611	1.431303
20	H	-6.143867	-0.918649	1.867008	-6.348641	-0.369006	-0.534096	-6.060160	-0.646309	1.256483
21	O	-3.231765	2.077601	1.000034	-3.171108	2.048393	0.957326	-3.087865	2.335984	0.529834
22	H	-2.553641	2.799533	1.145097	-2.586972	2.855935	1.043679	-2.453128	3.093867	0.684255
23	O	-1.059971	3.399169	0.982073	-1.166210	3.607173	0.841838	-0.964042	3.727540	0.645569
24	O	2.777145	1.661921	-1.194673	2.925338	2.058993	-1.002828	3.165898	1.861101	-0.768961
25	C	3.463233	2.927867	-1.189257	3.485775	3.375769	-1.173172	3.848693	3.127556	-0.852054
26	H	3.644013	3.260319	-0.163024	3.586921	3.869888	-0.203178	3.878822	3.604274	0.131387
27	H	2.880338	3.676375	-1.732950	2.855346	3.969678	-1.840229	3.350324	3.777113	-1.576485
28	H	4.409165	2.747122	-1.696931	4.465659	3.218837	-1.620088	4.857033	2.892253	-1.187444
29	C	-2.711609	-3.295021	-1.636189	-1.940975	-3.483650	-1.030956	-2.106550	-3.248317	-1.451202
30	H	-2.296205	-2.978439	-2.598401	-1.144914	-3.765710	-0.335228	-1.406552	-3.616049	-0.694841
31	H	-3.504382	-4.024000	-1.802425	-1.511319	-3.234420	-2.006639	-1.565859	-3.005379	-2.371694
32	H	-1.925355	-3.742580	-1.019567	-2.642611	-4.310104	-1.141044	-2.857192	-4.010624	-1.658252
33	O	1.273480	-0.413766	-2.019290	1.762754	-0.276404	-1.500079	1.875984	-0.363052	-1.437007
34	H	2.185901	-0.756601	-1.994543	2.662767	0.015363	-1.714795	2.816064	-0.148973	-1.544864
35	C	1.625982	-0.711531	0.400459	2.089069	-0.034882	0.924319	1.955722	-0.175563	1.012780
36	H	1.338545	-0.292166	1.367039	3.143303	0.239733	0.805836	3.035381	0.012102	1.014947
37	H	1.174457	-1.705911	0.322933	1.704175	0.504257	1.795260	1.523140	0.383811	1.847854
38	C	3.152486	-0.864982	0.374287	2.005877	-1.532493	1.262251	1.716207	-1.664792	1.310449
39	C	3.624468	-1.633397	1.612877	2.599674	-2.462072	0.199994	2.337497	-2.629366	0.296068
40	O	4.518888	-2.592262	1.326205	3.910686	-2.217139	0.003760	3.677819	-2.495946	0.240941
41	H	4.810894	-3.001480	2.160715	4.263337	-2.865902	-0.630730	4.038819	-3.165686	-0.366506
42	O	3.260921	-1.368599	2.743235	1.996039	-3.357049	-0.358069	1.722060	-3.462840	-0.339049

43	O	3.513341	-1.502064	-0.849727	2.660213	-1.789243	2.508702	2.217001	-1.988194	2.611276
44	H	4.474326	-1.482504	-0.962504	3.590434	-1.524766	2.439034	3.167202	-1.797412	2.640455
45	H	3.633446	0.118893	0.441989	0.972047	-1.843465	1.409899	0.650764	-1.890224	1.346896

Table S14: Energies and Boltzmann distribution of compound **20** (3*R*,4*R*,12*R*).

Conformer	Hartrees	kcal/mol	ΔG (kcal/mol)	Boltzmann Distribution (%)
Conformer 1	-1261.4466	-791569.863	0	34.2214197
Conformer 3	-1261.44638	-791569.726	0.1374246	26.5671842
Conformer 2	-1261.44623	-791569.631	0.23280606	22.2859661
Conformer 4	-1261.44599	-791569.481	0.38215335	16.9254300

Table S15: XYZ coordinates of compound **20** (3*R*,4*R*,12*R*).

		**Conformer 1**			**Conformer 2**			**Conformer 3**			**Conformer 4**		
Row	Symbol	X	Y	Z	X	Y	Z	X	Y	Z	X	Y	Z
1	C	-1.869207	1.262485	0.541337	-1.814585	1.494749	0.146430	-1.817356	1.306988	0.458393	-1.756321	1.513096	-0.002682
2	C	-1.137191	-1.019055	-0.891596	-1.124288	-0.910062	-1.091899	-1.137601	-1.060115	-0.856820	-1.118709	-1.005343	-1.025407
3	C	-0.519292	1.110127	0.090794	-0.441432	1.215193	-0.144923	-0.470418	1.099914	0.021130	-0.388947	1.176711	-0.258930
4	C	-2.854604	0.304169	0.272915	-2.842278	0.606244	-0.194140	-2.825080	0.360418	0.234467	-2.803605	0.623381	-0.273395
5	C	-2.466809	-0.828980	-0.466178	-2.471573	-0.596335	-0.824148	-2.463315	-0.817306	-0.445834	-2.459362	-0.637395	-0.795630
6	C	-0.167164	-0.066726	-0.614174	-0.114195	-0.021890	-0.751990	-0.145400	-0.118669	-0.624205	-0.088876	-0.115628	-0.755799
7	H	-0.869640	-1.908198	-1.446021	-0.872968	-1.847072	-1.569721	-0.889982	-1.982402	-1.364205	-0.887483	-1.986164	-1.417764
8	C	0.442058	2.170831	0.332993	0.572665	2.209497	0.156533	0.515603	2.148540	0.213242	0.647457	2.170022	-0.040176
9	C	1.253128	-0.322724	-1.117747	1.326454	-0.414519	-1.078976	1.270220	-0.433838	-1.107425	1.343823	-0.570478	-1.034078
10	O	-3.453169	-1.722412	-0.725133	-3.493891	-1.428117	-1.142250	-3.470046	-1.698898	-0.662554	-3.499961	-1.468452	-1.048727
11	C	-4.278647	0.496372	0.741914	-4.285927	0.926984	0.118351	-4.244985	0.610874	0.688219	-4.240466	1.003994	0.000933
12	H	-4.710987	-0.480987	0.975199	-4.418704	2.012169	0.091602	-4.702809	-0.342805	0.966255	-4.345466	2.086411	-0.113986
13	H	-4.268526	1.077883	1.667971	-4.921786	0.501180	-0.663186	-4.222583	1.235752	1.585408	-4.882446	0.532506	-0.748686
14	C	-5.169264	1.209650	-0.294549	-4.744097	0.394413	1.490456	-5.113963	1.296275	-0.384799	-4.719745	0.595112	1.407845
15	H	-5.223282	0.638435	-1.226907	-4.145244	0.825221	2.299475	-5.179782	0.682771	-1.289080	-4.114525	1.074482	2.184175
16	H	-4.779983	2.205940	-0.528273	-4.650317	-0.695173	1.541316	-4.698536	2.270037	-0.663972	-4.654494	-0.488879	1.546615
17	H	-6.187804	1.325774	0.091647	-5.793234	0.653250	1.670365	-6.130318	1.456539	-0.008832	-5.762801	0.894034	1.558495
18	O	-2.231975	2.359688	1.238092	-2.157380	2.649737	0.753914	-2.155250	2.445801	1.098426	-2.073886	2.723735	0.501329
19	H	-1.412397	2.920501	1.330434	-1.309612	3.153678	0.903533	-1.322933	2.991457	1.164311	-1.215146	3.219289	0.610829
20	O	0.158551	3.189351	1.001731	0.302825	3.283484	0.738714	0.254288	3.206173	0.828149	0.400844	3.296779	0.444217
21	C	-3.155899	-2.920284	-1.455352	-3.222258	-2.669517	-1.806847	-3.200847	-2.937514	-1.333640	-3.255075	-2.769755	-1.599698
22	H	-2.787219	-2.686984	-2.459127	-2.738123	-2.499804	-2.773615	-2.826445	-2.761667	-2.346934	-2.759503	-2.697398	-2.572816
23	H	-4.101615	-3.456236	-1.528622	-4.195809	-3.133449	-1.961634	-4.158976	-3.453814	-1.382363	-4.238953	-3.221190	-1.722737
24	H	-2.425748	-3.535554	-0.920237	-2.600581	-3.320482	-1.184140	-2.485701	-3.543249	-0.768372	-2.654857	-3.378580	-0.916357
25	O	1.201883	-0.231237	-2.538140	1.441271	-0.390319	-2.498343	1.231395	-0.406992	-2.530488	1.472386	-0.674876	-2.448197
26	H	2.119468	-0.124424	-2.842115	2.392566	-0.374788	-2.699517	2.153792	-0.344309	-2.831920	2.425451	-0.706064	-2.638579
27	C	1.834427	-1.676375	-0.624810	1.733675	-1.782886	-0.466864	1.813222	-1.773344	-0.542554	1.710567	-1.884686	-0.294936
28	H	1.081566	-2.455713	-0.516027	0.911644	-2.495478	-0.416338	1.052077	-2.543005	-0.426542	0.884309	-2.588695	-0.211591
29	H	2.579075	-2.018629	-1.345621	2.525194	-2.217399	-1.079999	2.582845	-2.148368	-1.219596	2.524950	-2.370329	-0.835606
30	C	1.806833	2.084688	-0.306972	1.988489	1.980354	-0.314442	1.880618	1.995430	-0.413105	2.059242	1.865936	-0.479766
31	H	2.507989	2.659856	0.301566	2.662690	2.529701	0.346268	2.592841	2.584273	0.168905	2.743563	2.457401	0.132323
32	H	1.738642	2.571718	-1.288104	2.074548	2.417653	-1.317317	1.830203	2.431226	-1.419127	2.160470	2.206853	-1.517983
33	C	2.294498	0.659935	-0.508617	2.371607	0.512289	-0.394112	2.331521	0.549471	-0.534919	2.405472	0.387562	-0.420956
34	O	2.602542	0.113019	0.762794	2.482208	0.010000	0.927339	2.608649	0.060130	0.768163	2.488337	0.005289	0.943556
35	O	3.406007	0.581312	-1.385651	3.565553	0.298264	-1.128536	3.450939	0.397015	-1.391150	3.601781	0.079438	-1.115852

36	C	4.577277	1.330821	-1.030582	4.749243	0.965725	-0.666943	4.638964	1.129206	-1.056738	4.796400	0.757363	-0.700167
37	H	4.422065	2.404938	-1.176047	4.705422	2.040703	-0.870331	4.515634	2.199638	-1.251455	4.782277	1.810229	-1.000596
38	H	5.365655	0.988810	-1.701945	5.576318	0.528697	-1.227469	5.424075	0.735935	-1.703407	5.618676	0.251500	-1.207322
39	H	4.867319	1.131603	0.005285	4.900955	0.793224	0.402662	4.913052	0.968455	-0.009903	4.931189	0.678921	0.382660
40	C	2.523680	-1.317627	0.713517	2.292695	-1.411345	0.927733	2.446774	-1.368628	0.804237	2.206703	-1.398662	1.081779
41	C	1.775035	-1.816062	1.944503	1.370706	-1.789870	2.081325	1.631192	-1.811442	2.015059	1.216188	-1.664333	2.211534
42	O	1.191622	-1.132825	2.759334	0.754186	-1.025405	2.793051	1.311892	-2.974596	2.185079	0.774150	-2.777786	2.431829
43	O	1.856498	-3.160034	2.021572	1.335465	-3.130704	2.222469	1.344452	-0.832367	2.883227	0.920658	-0.591470	2.957363
44	H	1.364120	-3.458417	2.806772	0.734202	-3.351894	2.955626	0.858454	-1.225953	3.630489	0.316640	-0.875593	3.667213
45	H	3.532488	-1.742685	0.758994	3.251541	-1.909593	1.108284	3.429634	-1.838188	0.931285	3.125205	-1.921380	1.374748

Table S16: Energies and Boltzmann distribution of compound **20** (3*R*,4*R*,12*S*).

Conformer	Hartrees	kcal/mol	ΔG (kcal/mol)	Boltzmann Distribution (%)
Conformer 2	-1261.447652	-791570.522	0	39.1811379
Conformer 1	-1261.447538	-791570.45	0.0715361	34.3432585
Conformer 4	-1261.446751	-791569.956	0.56538616	13.8266002
Conformer 3	-1261.446674	-791569.908	0.6137044	12.64900336

Table S17: XYZ coordinates of compound **20** (3*R*,4*R*,12*S*).

		Conformer 1			Conformer 2			Conformer 3			Conformer 4		
Row	Symbol	X	Y	Z	X	Y	Z	X	Y	Z	X	Y	Z
1	C	-2.257268	-1.110630	-0.606726	-2.272114	-1.271946	-0.299862	-2.247616	-1.121106	-0.600874	-2.261717	1.279892	0.294631
2	C	-1.314910	1.212512	0.623263	-1.349960	1.094063	0.862039	-1.331542	1.217304	0.620254	-1.363853	-1.099354	-0.859486
3	C	-0.913668	-1.062836	-0.115705	-0.896684	-1.156576	0.079215	-0.905403	-1.057111	-0.107915	-0.887501	1.149116	-0.083898
4	C	-3.134076	-0.026127	-0.478475	-3.191511	-0.238013	-0.082817	-3.135740	-0.045137	-0.479173	-3.191661	0.254842	0.080708
5	C	-2.643147	1.127765	0.160613	-2.706226	0.941917	0.511758	-2.658018	1.116630	0.155746	-2.718456	-0.932167	-0.509624
6	C	-0.452378	0.134811	0.484208	-0.448197	0.062405	0.644644	-0.457667	0.148014	0.487218	-0.451721	-0.076100	-0.645642
7	H	-0.964265	2.119077	1.097243	-1.006770	2.017979	1.307169	-0.990903	2.129703	1.090319	-1.030080	-2.028313	-1.301241
8	C	-0.075338	-2.246014	-0.201350	-0.010141	-2.296226	-0.079755	-0.054814	-2.232037	-0.185687	0.010562	2.280230	0.070315
9	C	0.974087	0.284587	1.012055	1.008946	0.286296	1.047717	0.967329	0.316703	1.013321	1.003200	-0.317167	-1.046699
10	O	-3.528311	2.146921	0.284818	-3.627443	1.916419	0.710725	-3.553954	2.126958	0.273990	-3.649507	-1.897867	-0.705058
11	C	-4.554855	-0.107450	-0.988047	-4.641033	-0.385516	-0.485198	-4.554438	-0.143190	-0.991553	-4.639669	0.418686	0.482185
12	H	-4.865627	0.882569	-1.334101	-4.917809	-1.441562	-0.422139	-4.874401	0.842099	-1.342664	-4.905401	1.477351	0.415673
13	H	-4.580745	-0.777842	-1.851660	-5.265340	0.158486	0.229464	-4.571471	-0.817534	-1.852315	-5.269410	-0.121033	-0.230946
14	C	-5.553955	-0.609588	0.073061	-4.932853	0.130744	-1.908050	-5.550885	-0.650736	0.069459	-4.937232	-0.089950	1.906588
15	H	-5.572959	0.055835	0.942178	-4.345180	-0.415465	-2.653109	-5.578590	0.018307	0.935554	-4.343802	0.452224	2.650026
16	H	-5.288481	-1.613815	0.419183	-4.691480	1.194773	-1.998107	-5.276134	-1.650700	0.420644	-4.707367	-1.156229	2.000018
17	H	-6.565957	-0.651461	-0.344226	-5.993020	0.001698	-2.151800	-6.561429	-0.704611	-0.349962	-5.995989	0.051293	2.149695
18	O	-2.721269	-2.227403	-1.205368	-2.724949	-2.407770	-0.870663	-2.699066	-2.245427	-1.195050	-2.702831	2.422245	0.861561
19	H	-1.970278	-2.883706	-1.201930	-1.945628	-3.027891	-0.925263	-1.941535	-2.893980	-1.187454	-1.917118	3.034252	0.914624
20	O	-0.453633	-3.291637	-0.774450	-0.381501	-3.359724	-0.623760	-0.420912	-3.284280	-0.754489	-0.349122	3.349029	0.611705
21	C	-3.119703	3.375126	0.902314	-3.237099	3.150758	1.327726	-3.158780	3.362578	0.885452	-3.271132	-3.139701	-1.314578
22	H	-2.307398	3.846216	0.340026	-4.148684	3.744262	1.388717	-2.350729	3.838994	0.321561	-2.873972	-2.978374	-2.321577
23	H	-2.812592	3.210001	1.939720	-2.496577	3.677323	0.717526	-2.851216	3.206121	1.924060	-4.188505	-3.724537	-1.372337
24	H	-3.999920	4.016729	0.879505	-2.841893	2.979516	2.333856	-4.045533	3.994933	0.858262	-2.536014	-3.669773	-0.700919
25	O	0.884194	0.361029	2.429819	1.036860	0.363579	2.468075	0.878276	0.406113	2.430036	1.031450	-0.406899	-2.465944
26	H	1.778079	0.191707	2.773495	1.964146	0.240139	2.734017	1.773702	0.246729	2.774522	1.959477	-0.289641	-2.732314
27	C	1.713530	1.495481	0.385169	1.630152	1.531262	0.360738	1.692512	1.528839	0.373671	1.610073	-1.562318	-0.348791
28	H	1.078019	2.365938	0.230970	0.937825	2.365069	0.255984	1.051948	2.396385	0.223974	0.914575	-2.394351	-0.250929

29	H	2.538131	1.775575	1.045352	2.487897	1.860312	0.952590	2.525936	1.813722	1.020692	2.476865	-1.894008	-0.925989
30	C	1.267351	-2.243770	0.488866	1.382430	-2.226047	0.498927	1.285754	-2.212471	0.508403	1.401013	2.194698	-0.511455
31	H	1.914283	-2.963190	-0.017706	2.020480	-2.914613	-0.059121	1.940762	-2.929384	0.008733	2.046663	2.879775	0.042137
32	H	1.110573	-2.599539	1.515165	1.328236	-2.585049	1.534624	1.129087	-2.562552	1.536726	1.347093	2.550346	-1.548395
33	C	1.911117	-0.868128	0.545456	1.959104	-0.819790	0.502075	1.916661	-0.830396	0.556685	1.965461	0.783347	-0.509806
34	O	2.293561	-0.506389	-0.775086	2.207180	-0.444509	-0.845994	2.300806	-0.474440	-0.766124	2.218022	0.412752	0.840301
35	O	3.006693	-0.814266	1.440097	3.122839	-0.708080	1.299968	3.009433	-0.760446	1.453237	3.125194	0.657774	-1.310689
36	C	4.099925	-1.712992	1.198268	4.239553	-1.542952	0.956902	4.107071	-1.657681	1.227310	4.237558	1.509979	-0.999032
37	H	3.822904	-2.747546	1.426215	4.035802	-2.594223	1.185461	3.833818	-2.690186	1.468473	4.031073	2.549682	-1.273335
38	H	4.896838	-1.399734	1.873712	5.068934	-1.197501	1.575143	4.900382	-1.331815	1.901024	5.071170	1.141340	-1.597873
39	H	4.443527	-1.629862	0.164051	4.496178	-1.424767	-0.098914	4.454642	-1.587943	0.193417	4.491253	1.439699	0.061792
40	C	2.234123	0.916714	-0.944895	2.074417	0.974449	-1.006429	2.196032	0.946183	-0.960647	2.034166	-1.001408	1.022429
41	C	3.592980	1.470656	-1.350177	3.375428	1.584498	-1.509812	3.494401	1.591954	-1.418439	3.264428	-1.703827	1.575325
42	O	3.477901	2.768309	-1.699035	3.181252	2.875629	-1.847459	4.583682	0.827743	-1.262384	4.402423	-1.005616	1.462889
43	H	4.358553	3.107160	-1.939528	4.026928	3.250275	-2.151949	5.356359	1.337436	-1.566844	5.127014	-1.548608	1.822820
44	O	4.641985	0.861826	-1.366120	4.444728	1.019503	-1.603816	3.523866	2.722774	-1.871376	3.204364	-2.821401	2.057213
45	H	1.535465	1.128082	-1.762460	1.308947	1.154898	-1.769675	1.469108	1.122266	-1.760828	1.241419	-1.144023	1.764231

Table S18: Energies and Boltzmann distribution of compound **20** (3*R*,4*S*,12*R*).

Conformer	Hartrees	kcal/mol	ΔG (kcal/mol)	Boltzmann Distribution (%)
Conformer 1	-1261.441295	-791566.5326	0	31.67287064
Conformer 2	-1261.440843	-791566.2489	0.283634343	18.78250477
Conformer 9	-1261.440446	-791565.9998	0.532755657	11.86952198
Conformer 7	-1261.440233	-791565.8662	0.666415204	9.278828118
Conformer 10	-1261.440172	-791565.8279	0.70469329	8.647032516
Conformer 8	-1261.440129	-791565.8009	0.731676203	8.227696169
Conformer 4	-1261.439577	-791565.4545	1.078061507	4.346480216
Conformer 5	-1261.439413	-791565.3516	1.180973082	3.595827517
Conformer 6	-1261.439409	-791565.3491	1.183483121	3.579238075

Table S19: XYZ coordinates of compound **20** (3*R*,4*S*,12*R*).

		Conformer 1			Conformer 2			Conformer 4			Conformer 5		
Row	Symbol	X	Y	Z	X	Y	Z	X	Y	Z	X	Y	Z
1	C	-2.475349	-1.033996	-0.159822	-2.471614	-0.949035	-0.513273	-2.472069	-1.041757	-0.146308	-2.469514	-0.962302	-0.500921
2	C	-1.135271	1.396056	0.232552	-1.111915	1.451173	-0.017525	-1.140662	1.398451	0.211658	-1.119271	1.450228	-0.039577
3	C	-1.050473	-0.993169	-0.240542	-1.044740	-0.943787	-0.463935	-1.047741	-0.996034	-0.233269	-1.042456	-0.949497	-0.458783
4	C	-3.234804	0.110261	0.131893	-3.225063	0.218150	-0.310292	-3.235152	0.102919	0.134654	-3.227699	0.203614	-0.307705
5	C	-2.542950	1.319691	0.325009	-2.523679	1.409881	-0.052094	-2.547796	1.317401	0.310562	-2.531121	1.401693	-0.067186
6	C	-0.405132	0.256822	-0.045215	-0.388651	0.291798	-0.219478	-0.407121	0.259042	-0.055330	-0.391478	0.292107	-0.231079
7	H	-0.626663	2.341597	0.364744	-0.593504	2.384522	0.156604	-0.635553	2.347623	0.330295	-0.604627	2.388076	0.120815
8	C	-0.298337	-2.223205	-0.474446	-0.305453	-2.193897	-0.619014	-0.291613	-2.225583	-0.456211	-0.298070	-2.197619	-0.604427
9	C	1.089831	0.290813	-0.255781	1.119628	0.286958	-0.290656	1.086875	0.296955	-0.272300	1.116593	0.294094	-0.307865
10	O	-3.316659	2.399533	0.597182	-3.291687	2.509011	0.149584	-3.324751	2.397209	0.572364	-3.303391	2.499340	0.124597
11	C	-4.742832	0.039539	0.216619	-4.736184	0.182802	-0.351116	-4.742523	0.027109	0.225944	-4.738807	0.160409	-0.340074
12	H	-5.029390	-0.956064	0.566937	-5.103591	1.132094	-0.751534	-5.023768	-0.965692	0.588225	-5.112921	1.102433	-0.751280
13	H	-5.091094	0.759887	0.962401	-5.050604	-0.604962	-1.041408	-5.090734	0.754268	0.965098	-5.053037	-0.638134	-1.017899

14	C	-5.442389	0.323682	-1.127193	-5.377642	-0.067109	1.028051	-5.448120	0.293830	-1.118288	-5.371424	-0.073780	1.045954
15	H	-5.137778	-0.400363	-1.889919	-5.105842	0.721227	1.737443	-5.143441	-0.437274	-1.874209	-5.099567	0.725453	1.742979
16	H	-5.198693	1.326111	-1.493741	-5.053016	-1.026098	1.444928	-5.209796	1.293159	-1.496598	-5.039862	-1.025421	1.474040
17	H	-6.530130	0.259106	-1.013868	-6.469819	-0.086298	0.944575	-6.535144	0.226177	-1.000168	-6.463906	-0.099344	0.968786
18	O	-3.136556	-2.194392	-0.352815	-3.141593	-2.095861	-0.752057	-3.129305	-2.206844	-0.322765	-3.135108	-2.114780	-0.723072
19	H	-2.442435	-2.885284	-0.540668	-2.450553	-2.803737	-0.877594	-2.433526	-2.897233	-0.505730	-2.441703	-2.820929	-0.844689
20	O	-0.874725	-3.312563	-0.690105	-0.886878	-3.270653	-0.879726	-0.864056	-3.319632	-0.657165	-0.875232	-3.279827	-0.850872
21	C	-2.703071	3.675852	0.822706	-2.668860	3.776034	0.400980	-2.715615	3.678626	0.781129	-2.685803	3.772500	0.357661
22	H	-2.028178	3.640814	1.683652	-2.072488	3.745065	1.318263	-2.037676	3.656116	1.640065	-2.085420	3.755797	1.272649
23	H	-3.525595	4.359501	1.030382	-3.489340	4.482550	0.522240	-3.540264	4.361179	0.983821	-3.509286	4.476357	0.473717
24	H	-2.160986	4.013336	-0.066303	-2.043487	4.081092	-0.443849	-2.178060	4.008094	-0.113600	-2.065611	4.070180	-0.493562
25	C	1.226856	-2.214942	-0.453382	1.211192	-2.222785	-0.458343	1.233946	-2.211512	-0.441185	1.219726	-2.218134	-0.450871
26	H	1.577632	-2.174225	-1.488764	1.656129	-2.200078	-1.457491	1.580779	-2.186761	-1.478429	1.659891	-2.208592	-1.452365
27	H	1.571667	-3.158365	-0.024873	1.491151	-3.170613	0.006132	1.583656	-3.147216	0.000016	1.505584	-3.158553	0.024873
28	C	1.726668	-0.999777	0.306806	1.669846	-1.013552	0.336625	1.732209	-0.984632	0.300522	1.676735	-0.997950	0.327575
29	O	3.117156	-0.764125	0.148094	3.074560	-0.812543	0.304534	3.120221	-0.740984	0.130317	3.079640	-0.786449	0.283984
30	O	1.386959	-1.049927	1.684046	1.204780	-1.043862	1.677238	1.400834	-1.016816	1.679688	1.220185	-1.014220	1.670836
31	C	2.030382	-2.066095	2.465313	1.747500	-2.070288	2.519379	2.046679	-2.025133	2.469899	1.772588	-2.028804	2.521648
32	H	1.785456	-1.841940	3.504449	1.416393	-1.831041	3.530893	1.817465	-1.781129	3.508081	1.451583	-1.775696	3.532967
33	H	3.116076	-2.038070	2.331825	2.841154	-2.072329	2.483911	3.130672	-2.008598	2.321518	2.865805	-2.030704	2.474913
34	H	1.654070	-3.065181	2.219255	1.367655	-3.060966	2.246258	1.658588	-3.024700	2.245541	1.390616	-3.023142	2.265692
35	O	1.270231	0.337803	-1.679633	1.431419	0.316584	-1.692188	1.255277	0.327149	-1.699988	1.417801	0.311059	-1.713850
36	H	2.222823	0.416804	-1.885665	2.400708	0.367068	-1.810397	2.203450	0.353713	-1.915460	2.382270	0.310531	-1.840141
37	C	1.969881	1.346371	0.433719	1.958261	1.327119	0.469754	1.963853	1.368701	0.395844	1.952368	1.350194	0.433710
38	H	1.640977	1.536156	1.455082	1.542223	1.533334	1.455482	1.614224	1.604816	1.400547	1.517368	1.600987	1.400747
39	H	1.985900	2.288123	-0.118387	2.047499	2.263429	-0.084510	1.998463	2.287297	-0.193286	2.062455	2.263617	-0.154091
40	C	3.361095	0.629978	0.453455	3.323942	0.578054	0.620863	3.352702	0.644115	0.481414	3.310400	0.595127	0.649554
41	H	3.850325	0.717350	1.425158	3.724944	0.663171	1.632295	3.770510	0.709004	1.487822	3.635211	0.656615	1.689910
42	C	4.280615	1.169605	-0.635538	4.350730	1.087304	-0.383543	4.379137	1.245719	-0.466496	4.432329	1.169369	-0.202035
43	O	4.099572	0.983479	-1.827799	4.280051	0.886496	-1.584996	5.236307	2.033110	-0.121301	5.264723	1.948074	0.215941
44	O	5.283239	1.903282	-0.143633	5.313622	1.814021	0.191187	4.198977	0.856776	-1.745137	4.369481	0.768025	-1.487990
45	H	5.803959	2.271065	-0.881298	5.906410	2.164117	-0.499134	4.840200	1.318110	-2.315088	5.069793	1.213193	-1.998129
		**Conformer 6**			**Conformer 7**			**Conformer 8**			**Conformer 9**		
Row	Symbol	X	Y	Z	X	Y	Z	X	Y	Z	X	Y	Z
1	C	-2.469580	-0.962058	-0.500835	-2.475631	-1.045990	-0.167743	-2.473830	-0.962362	-0.515163	-2.439189	-1.077927	-0.203457
2	C	-1.119204	1.450319	-0.039530	-1.141510	1.386302	0.234950	-1.117983	1.441709	-0.025097	-1.172785	1.376458	0.276702
3	C	-1.042544	-0.949398	-0.458822	-1.051003	-1.004694	-0.234586	-1.048050	-0.956509	-0.460103	-1.015042	-1.001335	-0.240237
4	C	-3.227710	0.203868	-0.307601	-3.239439	0.099629	0.111626	-3.229174	0.206725	-0.323983	-3.236010	0.043085	0.083498
5	C	-2.531053	1.401918	-0.067090	-2.551057	1.309404	0.310872	-2.530285	1.399993	-0.069223	-2.581295	1.264430	0.322337
6	C	-0.391491	0.292146	-0.231095	-0.407795	0.246530	-0.034911	-0.393007	0.280860	-0.217591	-0.406636	0.260370	-0.002109
7	H	-0.604469	2.388113	0.120932	-0.634675	2.331311	0.378979	-0.601034	2.374964	0.155158	-0.691448	2.329655	0.451137
8	C	-0.298255	-2.197590	-0.604539	-0.293968	-2.233966	-0.458673	-0.305890	-2.206449	-0.606142	-0.223701	-2.207188	-0.472846
9	C	1.116593	0.294090	-0.307884	1.087352	0.282696	-0.245823	1.115490	0.277979	-0.292204	1.090556	0.336866	-0.178838
10	O	-3.303244	2.499613	0.124732	-3.327555	2.389175	0.573417	-3.299145	2.499965	0.121925	-3.388751	2.319465	0.591921
11	C	-4.738812	0.160694	-0.339926	-4.748276	0.028150	0.177826	-4.740008	0.170402	-0.371840	-4.743682	-0.066161	0.116804
12	H	-5.112946	1.102986	-0.750507	-5.038551	-0.966799	0.526803	-5.105715	1.115719	-0.783096	-5.016422	-1.074371	0.441100
13	H	-5.053070	-0.637423	-1.018261	-5.106239	0.750069	0.917415	-5.051009	-0.624108	-1.055858	-5.134566	0.632616	0.861927
14	C	-5.371384	-0.074376	1.045967	-5.430865	0.308653	-1.175513	-5.387629	-0.066112	1.006883	-5.404987	0.223135	-1.245260
15	H	-5.099531	0.724448	1.743482	-5.116384	-0.417155	-1.932534	-5.119140	0.729235	1.709625	-5.057085	-0.480337	-2.008705
16	H	-5.039775	-1.026267	1.473465	-5.183150	1.310336	-1.541422	-5.064799	-1.020881	1.434643	-5.174732	1.237285	-1.587523
17	H	-6.463873	-0.099921	0.968799	-6.519877	0.243792	-1.075739	-6.479369	-0.086289	0.918531	-6.493885	0.129856	-1.169637
18	O	-3.135288	-2.114525	-0.722894	-3.134004	-2.206998	-0.362930	-3.142344	-2.110555	-0.748343	-3.065203	-2.250027	-0.435792
19	H	-2.441883	-2.820652	-0.844439	-2.437294	-2.898423	-0.540218	-2.450422	-2.819346	-0.865243	-2.348502	-2.921003	-0.611807
20	O	-0.875526	-3.279781	-0.850764	-0.867817	-3.324875	-0.673245	-0.886460	-3.285299	-0.859888	-0.766380	-3.307055	-0.720358
21	C	-2.685560	3.772749	0.357754	-2.717116	3.665868	0.806533	-2.678186	3.768977	0.368801	-2.814065	3.604153	0.868283
22	H	-2.065331	4.070321	-0.493484	-2.163934	4.003269	-0.075632	-2.088958	3.744820	1.290827	-2.169523	3.564368	1.751961

23	H	-2.085208	3.756008	1.272767	-2.053472	3.631228	1.676149	-3.499586	4.476236	0.478569	-3.659654	4.263301	1.061867
24	H	-3.509017	4.476650	0.473752	-3.542480	4.349218	1.003437	-2.046519	4.067805	-0.473532	-2.249379	3.975052	0.007135
25	C	1.219547	-2.218093	-0.451428	1.230783	-2.221828	-0.430552	1.210625	-2.231070	-0.446422	1.299391	-2.158401	-0.414107
26	H	1.659377	-2.207980	-1.453078	1.587427	-2.190949	-1.464140	1.656548	-2.217670	-1.445239	1.674908	-2.096909	-1.439674
27	H	1.505705	-3.158681	0.023790	1.575363	-3.159430	0.010858	1.492050	-3.172931	0.029320	1.658197	-3.096798	0.014110
28	C	1.676674	-0.998112	0.327281	1.730677	-0.998398	0.318494	1.672857	-1.013790	0.335909	1.755381	-0.939447	0.371086
29	O	3.079630	-0.786683	0.283635	3.117066	-0.771592	0.174718	3.071642	-0.821738	0.315714	3.139359	-0.677106	0.259789
30	O	1.220217	-1.014628	1.670529	1.369221	-1.034772	1.694433	1.189387	-1.029228	1.674343	1.368105	-1.018292	1.737923
31	C	1.772639	-2.029410	2.521110	2.025719	-2.024411	2.496989	1.745050	-2.029575	2.537681	2.045998	-1.998493	2.533836
32	H	1.450814	-1.777015	3.532349	1.746454	-1.803534	3.528150	1.383556	-1.791748	3.539084	1.722665	-1.822102	3.560684
33	H	2.865892	-2.030650	2.475052	3.112933	-1.961244	2.391185	2.838733	-1.997863	2.526612	3.131515	-1.879322	2.466402
34	H	1.391384	-3.023805	2.264318	1.690064	-3.037317	2.247759	1.403497	-3.034856	2.266597	1.769410	-3.018854	2.245282
35	O	1.417800	0.311352	-1.713853	1.359233	0.256629	-1.663990	1.513566	0.232830	-1.679741	1.397073	0.347100	-1.591937
36	H	2.382287	0.310920	-1.840106	0.882859	0.993819	-2.072390	1.102147	0.983234	-2.132320	0.923229	1.089537	-1.993995
37	C	1.952437	1.349986	0.433908	1.974006	1.349890	0.403889	1.965478	1.328821	0.429693	1.941242	1.408191	0.508403
38	H	1.517464	1.600646	1.401003	1.657035	1.568633	1.423807	1.558582	1.568375	1.412255	1.629028	1.563980	1.541216
39	H	2.062583	2.263507	-0.153747	1.996043	2.277895	-0.168910	2.067277	2.249080	-0.146987	1.932984	2.363072	-0.018629
40	C	3.310442	0.594773	0.649608	3.359413	0.626304	0.418320	3.323471	0.570011	0.584029	3.344209	0.739374	0.461160
41	H	3.635177	0.655935	1.690009	3.835807	0.747311	1.398329	3.700055	0.680802	1.607642	3.876978	0.890288	1.408010
42	C	4.432402	1.169305	-0.201747	4.356635	1.141205	-0.611959	4.433139	1.057977	-0.338044	4.249259	1.321043	-0.619020
43	O	5.264778	1.947837	0.216580	4.899759	0.490616	-1.479612	5.041679	0.394036	-1.150339	4.379332	2.520823	-0.786055
44	O	4.369593	0.768563	-1.487853	4.608212	2.450361	-0.400565	4.699185	2.358867	-0.095784	4.939197	0.403579	-1.311083
45	H	5.069896	1.214018	-1.997779	5.271609	2.747537	-1.048135	5.431607	2.638865	-0.672797	5.522562	0.873868	-1.933539

		Conformer 10		
Row	Symbol	X	Y	Z
1	C	-2.435924	-0.981276	-0.554447
2	C	-1.147318	1.439426	0.026945
3	C	-1.012286	-0.944425	-0.468246
4	C	-3.222200	0.165018	-0.350774
5	C	-2.557071	1.366752	-0.049385
6	C	-0.391599	0.300906	-0.180581
7	H	-0.656249	2.378942	0.243639
8	C	-0.238112	-2.172931	-0.628723
9	C	1.117051	0.334787	-0.220497
10	O	-3.355455	2.443342	0.153442
11	C	-4.730263	0.095065	-0.434518
12	H	-5.109542	1.045993	-0.819499
13	H	-5.006696	-0.680903	-1.153912
14	C	-5.402293	-0.207550	0.919340
15	H	-5.168131	0.566811	1.656997
16	H	-5.066576	-1.170175	1.318589
17	H	-6.491059	-0.249143	0.805359
18	O	-3.072606	-2.137900	-0.831180
19	H	-2.362177	-2.827745	-0.950191
20	O	-0.787906	-3.258177	-0.921018
21	C	-2.769972	3.719813	0.444789
22	H	-2.198837	3.685943	1.377810
23	H	-3.610136	4.404550	0.555395
24	H	-2.129005	4.054693	-0.376716
25	C	1.274857	-2.166132	-0.438075
26	H	1.740000	-2.115880	-1.426913
27	H	1.569157	-3.113575	0.018456
28	C	1.693360	-0.959230	0.385493
29	O	3.088413	-0.736054	0.398431
30	O	1.184130	-1.024300	1.712524
31	C	1.754198	-2.028301	2.561746

32	H	1.359284	-1.832412	3.559629
33	H	2.845832	-1.956553	2.579736
34	H	1.456648	-3.037188	2.254320
35	O	1.550948	0.335012	-1.599840
36	H	1.140940	1.092909	-2.041319
37	C	1.930596	1.382320	0.544260
38	H	1.525813	1.550956	1.542297
39	H	2.000745	2.335392	0.018528
40	C	3.311593	0.673175	0.631980
41	H	3.749647	0.802953	1.629160
42	C	4.338561	1.233451	-0.345178
43	O	4.509162	2.429526	-0.501590
44	O	5.082347	0.299148	-0.953902
45	H	5.739011	0.754438	-1.511079

Table S20: Energies and Boltzmann distribution of compound **20** (3*R*,4*S*,12*S*).

Conformer	Hartrees	kcal/mol	ΔG (kcal/mol)	Boltzmann Distribution (%)
Conformer 5	-1261.44069	-791566.1529	0	17.3557605
Conformer 6	-1261.440689	-791566.1523	0.00062751	17.3357079
Conformer 4	-1261.440626	-791566.1128	0.04016061	16.1180097
Conformer 11	-1261.440497	-791566.0318	0.12110935	13.8849677
Conformer 14	-1261.440351	-791565.9402	0.21272576	11.7285199
Conformer 12	-1261.440177	-791565.831	0.32191243	9.59143727
Conformer 13	-1261.440176	-791565.8304	0.32253994	9.5803555
Conformer 1	-1261.438548	-791564.8088	1.34412558	1.45887426
Conformer 2	-1261.43851	-791564.785	1.36797095	1.39617336
Conformer 8	-1261.438028	-791564.4825	1.67043058	0.7997301
Conformer 9	-1261.437973	-791564.448	1.7049436	0.75046389

Table S21:XYZ coordinates of compound **20** (3*R*,4*S*,12*S*).

		**Conformer 1**			**Conformer 2**			**Conformer 4**			**Conformer 5**		
Row	Symbol	X	Y	Z	X	Y	Z	X	Y	Z	X	Y	Z
1	C	2.457793	-1.057872	-0.189732	-2.489174	-1.060212	-0.154100	2.490634	-1.043154	-0.144173	-2.519163	-1.025909	-0.198542
2	C	1.158364	1.397491	0.183738	-1.165421	1.395434	-0.425490	1.143170	1.402156	0.110467	-1.143136	1.411816	-0.354967
3	C	1.079934	-1.038726	0.183172	-1.084539	-1.040598	-0.408231	1.103483	-1.034846	0.189222	-1.108687	-1.025959	-0.412601
4	C	3.186515	0.124500	-0.397452	-3.236470	0.121760	-0.025749	3.207371	0.144271	-0.368783	-3.248417	0.166441	-0.055345
5	C	2.516017	1.345758	-0.205245	-2.551691	1.343301	-0.156003	2.512920	1.359772	-0.236902	-2.537880	1.377754	-0.125632
6	C	0.456487	0.223325	0.373055	-0.448136	0.221607	-0.547641	0.454535	0.222435	0.320135	-0.444801	0.227645	-0.496216
7	H	0.668874	2.348377	0.346698	-0.663952	2.346493	-0.544554	0.633298	2.350219	0.220273	-0.620366	2.356688	-0.423620
8	C	0.343947	-2.292646	0.317760	-0.338501	-2.294081	-0.475487	0.380563	-2.295306	0.342141	-0.381651	-2.290618	-0.494724
9	C	-0.965430	0.211764	0.878182	1.013340	0.211709	-0.923500	-0.975792	0.199323	0.803806	1.021815	0.194969	-0.854438
10	O	3.259431	2.460568	-0.410443	-3.310634	2.457692	-0.015704	3.244410	2.479294	-0.458870	-3.279226	2.501638	0.032066
11	C	4.643975	0.077299	-0.797281	-4.720270	0.074386	0.261153	4.676328	0.108379	-0.725208	-4.740902	0.137803	0.185218
12	H	4.818981	-0.829297	-1.383277	-5.205002	0.921998	-0.231865	4.883640	-0.816621	-1.270387	-5.194114	1.014144	-0.287014
13	H	4.862565	0.931068	-1.445132	-5.134283	-0.837800	-0.177388	4.898719	0.940541	-1.399439	-5.158415	-0.747412	-0.302666

14	C	5.606598	0.095520	0.406508	-5.050536	0.107552	1.766447	5.603609	0.190756	0.503383	-5.116968	0.116314	1.679930
15	H	5.434421	-0.765268	1.060869	-4.672530	1.023284	2.232377	5.427760	-0.647020	1.186057	-4.736871	1.004764	2.194445
16	H	5.476730	1.005685	1.000915	-4.604882	-0.747105	2.285858	5.441096	1.120808	1.057634	-4.703428	-0.767341	2.176824
17	H	6.646952	0.058929	0.065217	-6.134393	0.070641	1.921438	6.653784	0.160062	0.193027	-6.205606	0.094372	1.800385
18	O	3.100308	-2.230829	-0.366632	-3.142068	-2.233389	-0.021270	3.155421	-2.210561	-0.266058	-3.196949	-2.189507	-0.119503
19	H	2.432393	-2.947894	-0.182522	-2.461606	-2.950351	-0.151128	2.492256	-2.931913	-0.079718	-2.525182	-2.915032	-0.250359
20	O	0.909406	-3.401550	0.192932	-0.911388	-3.403311	-0.396133	0.966841	-3.398028	0.265979	-0.977912	-3.390168	-0.462766
21	C	2.658979	3.754284	-0.260018	-2.707641	3.751823	-0.152210	2.619273	3.767172	-0.373113	-2.648430	3.787545	-0.041508
22	H	1.828948	3.885684	-0.961203	-1.936316	3.907298	0.608579	1.809726	3.859936	-1.103750	-1.896207	3.901030	0.745331
23	H	3.449051	4.468229	-0.490328	-3.517447	4.465179	-0.002789	3.404808	4.485812	-0.604306	-3.448588	4.511091	0.110777
24	H	2.312384	3.909143	0.766534	-2.282060	3.883594	-1.151944	2.238228	3.952357	0.636087	-2.192321	3.946003	-1.023751
25	C	-1.153605	-2.285283	0.611008	1.178327	-2.285245	-0.641948	-1.123575	-2.300595	0.594833	1.138502	-2.304441	-0.617304
26	H	-1.285834	-2.460215	1.682840	1.400173	-2.457555	-1.699365	-1.284882	-2.441659	1.667541	1.391069	-2.454603	-1.671010
27	H	-1.613695	-3.125381	0.086889	1.593752	-3.125858	-0.082694	-1.558037	-3.159818	0.079636	1.522407	-3.161595	-0.060169
28	C	-1.752902	-0.944763	0.223265	1.741016	-0.944767	-0.202807	-1.732621	-0.978425	0.157943	1.714345	-0.981846	-0.138949
29	O	-3.069594	-0.745554	0.728810	3.096770	-0.744297	-0.591509	-3.068946	-0.799674	0.590459	3.083604	-0.812309	-0.456393
30	O	-1.710070	-0.711826	-1.171184	1.576311	-0.712838	1.182696	-1.624937	-0.779874	-1.243996	1.487692	-0.772474	1.247226
31	C	-2.434095	-1.632011	-2.003484	2.225557	-1.632443	2.075143	-2.388079	-1.665859	-2.075440	2.173418	-1.654861	2.147163
32	H	-1.912297	-2.591440	-2.085505	2.171464	-1.173170	3.063198	-2.300556	-1.268588	-3.087855	2.001728	-1.249548	3.145525
33	H	-2.477318	-1.167733	-2.989739	3.273370	-1.770978	1.798213	-3.439034	-1.671638	-1.775992	3.245905	-1.666465	1.938161
34	H	-3.449956	-1.779843	-1.629326	1.706185	-2.596258	2.103170	-1.983276	-2.683898	-2.059337	1.767169	-2.671487	2.104086
35	O	-0.855327	-0.020392	2.296178	1.030743	-0.018488	-2.346026	-0.983560	-0.068582	2.223220	1.148697	-0.081099	-2.266709
36	H	-1.735973	-0.201866	2.659805	1.941074	-0.196020	-2.629902	-0.442470	0.606720	2.657238	0.644442	0.590952	-2.747741
37	C	-1.921908	1.380992	0.607338	1.940964	1.381409	-0.567453	-1.953107	1.342947	0.513347	1.976504	1.336383	-0.488823
38	H	-1.784188	1.778178	-0.398619	1.715524	1.776164	0.423466	-1.853497	1.695348	-0.514607	1.788691	1.700314	0.522543
39	H	-1.801358	2.186632	1.332225	1.883200	2.188716	-1.298145	-1.836788	2.185970	1.196093	1.925176	2.172331	-1.187996
40	C	-3.309261	0.677767	0.752513	3.336265	0.679408	-0.593211	-3.310677	0.611338	0.739903	3.345257	0.596904	-0.587477
41	C	-4.326342	1.088588	-0.304798	4.258871	1.090553	0.547571	-4.411317	1.082317	-0.198640	4.358842	1.065425	0.445867
42	O	-4.949673	0.342751	-1.029773	4.817940	0.345295	1.323796	-4.987883	0.413197	-1.030290	4.861828	0.393271	1.321718
43	O	-4.485326	2.427807	-0.299082	4.417390	2.429793	0.555041	-4.689132	2.382902	0.031364	4.654765	2.366947	0.245754
44	H	-5.165655	2.664589	-0.954345	5.039416	2.666905	1.265766	-5.399530	2.661543	-0.573491	5.311232	2.642994	0.909823
45	H	-3.768658	0.908693	1.720385	3.876042	0.911589	-1.518331	-3.663278	0.776349	1.763291	3.791397	0.756226	-1.574631
		**Conformer 6**			**Conformer 8**			**Conformer 9**			**Conformer 11**		
Row	Symbol	X	Y	Z	X	Y	Z	X	Y	Z	X	Y	Z
1	C	-2.519168	-1.025896	-0.198551	2.420836	-1.091316	-0.220047	-2.452094	-1.107343	-0.124718	-2.493953	-1.065420	-0.172023
2	C	-1.143129	1.411823	-0.354958	1.198375	1.391396	0.224552	-1.209349	1.382234	-0.461031	-1.182363	1.403205	-0.383271
3	C	-1.108691	-1.025952	-0.412607	1.049737	-1.040798	0.174908	-1.052499	-1.049120	-0.400743	-1.086955	-1.033243	-0.406562
4	C	-3.248416	0.166456	-0.055346	3.180291	0.073694	-0.414012	-3.233858	0.053025	-0.006549	-3.251085	0.110054	-0.035269
5	C	-2.537874	1.377767	-0.125624	2.548190	1.309274	-0.185703	-2.589061	1.292456	-0.168919	-2.572424	1.337669	-0.133666
6	C	-0.444799	0.227650	-0.496213	0.465360	0.234229	0.400333	-0.457456	0.229232	-0.573391	-0.455946	0.235171	-0.517610
7	H	-0.620354	2.356693	-0.423603	0.738588	2.352112	0.413891	-0.739913	2.346060	-0.605156	-0.684730	2.359862	-0.472315
8	C	-0.381661	-2.290613	-0.494736	0.280687	-2.275742	0.295404	-0.269580	-2.279999	-0.457257	-0.329776	-2.280131	-0.481617
9	C	1.021817	0.194968	-0.854431	-0.949218	0.253856	0.925718	0.997690	0.257565	-0.972524	1.006485	0.235993	-0.894672
10	O	-3.279214	2.501653	0.032083	3.320473	2.406450	-0.378511	-3.379674	2.385478	-0.036258	-3.339517	2.444878	0.018219
11	C	-4.740902	0.137821	0.185214	4.629520	-0.006785	-0.837634	-4.711166	-0.035241	0.303414	-4.738545	0.047576	0.228891
12	H	-5.194107	1.014181	-0.286988	4.768090	-0.902355	-1.449814	-5.228918	0.790115	-0.193762	-5.220863	0.905538	-0.248221
13	H	-5.158421	-0.747374	-0.302702	4.864442	0.856935	-1.466226	-5.103425	-0.965741	-0.116373	-5.141256	-0.854730	-0.239818
14	C	-5.116970	0.116283	1.679924	5.609887	-0.049734	0.351089	-5.020204	0.008829	1.812918	-5.090582	0.038310	1.729578
15	H	-4.736869	1.004713	2.194471	5.421366	-0.922281	0.985067	-4.663627	0.942004	2.260783	-4.724123	0.942903	2.225552
16	H	-4.703437	-0.767392	2.176789	5.516220	0.847602	0.971243	-4.540988	-0.824535	2.337005	-4.647892	-0.827944	2.231974
17	H	-6.205609	0.094343	1.800377	6.643530	-0.108095	-0.007127	-6.100033	-0.058892	1.984709	-6.176348	-0.008123	1.867777
18	O	-3.196960	-2.189491	-0.119522	3.026235	-2.278156	-0.432281	-3.066015	-2.297462	0.039546	-3.140751	-2.244441	-0.066342
19	H	-2.525197	-2.915018	-0.250383	2.340908	-2.979750	-0.252924	-2.365690	-2.995452	-0.087897	-2.452811	-2.954756	-0.196221
20	O	-0.977926	-3.390161	-0.462787	0.811432	-3.397677	0.138253	-0.806021	-3.404912	-0.348643	-0.896844	-3.394017	-0.424225
21	C	-2.648415	3.787558	-0.041501	2.759365	3.713477	-0.194406	-2.818343	3.694920	-0.201928	-2.742133	3.744957	-0.082371
22	H	-3.448571	4.511107	0.110778	1.923701	3.882047	-0.880769	-2.411109	3.823339	-1.209690	-1.982152	3.888389	0.692045

23	H	-2.192305	3.946008	-1.023744	3.566271	4.409105	-0.422404	-2.041429	3.885874	0.544967	-3.558019	4.450466	0.071091
24	H	-1.896193	3.901048	0.745338	2.431803	3.857875	0.839877	-3.647370	4.385720	-0.051954	-2.303819	3.900480	-1.073163
25	C	1.138493	-2.304443	-0.617305	-1.211068	-2.231928	0.613207	1.243483	-2.228008	-0.647878	1.188053	-2.257049	-0.628710
26	H	1.391068	-2.454607	-1.671008	-1.330781	-2.421934	1.683910	1.453962	-2.412131	-1.705587	1.426784	-2.411706	-1.685008
27	H	1.522389	-3.161599	-0.060166	-1.703475	-3.048927	0.082103	1.693359	-3.045266	-0.080818	1.603429	-3.098098	-0.069706
28	C	1.714340	-0.981851	-0.138943	-1.778762	-0.868359	0.263213	1.772339	-0.863480	-0.244859	1.738623	-0.915024	-0.176965
29	O	3.083602	-0.812320	-0.456378	-3.078731	-0.639720	0.796440	3.112529	-0.626259	-0.662340	3.096844	-0.715613	-0.520734
30	O	1.487679	-0.772486	1.247231	-1.764507	-0.605328	-1.127916	1.636225	-0.606995	1.140988	1.537398	-0.688917	1.211162
31	C	2.173399	-1.654878	2.147167	-2.488633	-1.519697	-1.967084	2.293476	-1.519428	2.035674	2.220339	-1.576572	2.108129
32	H	2.001711	-1.249566	3.145530	-2.591536	-1.020246	-2.931529	2.303024	-1.026247	3.008740	2.118706	-1.132409	3.099519
33	H	3.245886	-1.666489	1.938166	-3.481605	-1.726719	-1.558955	3.321556	-1.711560	1.716990	3.280181	-1.654158	1.851413
34	H	1.767144	-2.671501	2.104088	-1.935267	-2.454040	-2.107999	1.741656	-2.461456	2.120208	1.761320	-2.571253	2.117533
35	O	1.148700	-0.081095	-2.266703	-0.827169	-0.012208	2.337175	1.000462	-0.003208	-2.390280	1.122752	-0.053697	-2.305472
36	H	0.644449	0.590959	-2.747734	-1.711313	-0.159490	2.708060	1.914367	-0.144257	-2.683285	0.597381	0.601123	-2.787655
37	C	1.976512	1.336377	-0.488813	-1.874013	1.457301	0.693124	1.892540	1.464481	-0.656177	1.932357	1.407145	-0.551897
38	H	1.788701	1.700302	0.522555	-1.716126	1.905194	-0.287692	1.647857	1.908195	0.308572	1.731478	1.797235	0.446468
39	H	1.925187	2.172328	-1.187982	-1.739351	2.221867	1.459146	1.820879	2.231032	-1.428692	1.861707	2.221986	-1.273922
40	C	3.345260	0.596891	-0.587473	-3.281770	0.796410	0.773816	3.305215	0.810977	-0.615528	3.321577	0.708394	-0.614960
41	C	4.358850	1.065416	0.445865	-4.217410	1.250300	-0.342316	4.136360	1.263639	0.581156	4.291385	1.239012	0.431001
42	O	4.861804	0.393279	1.321747	-4.211737	2.379743	-0.796134	4.077410	2.387196	1.045506	4.441219	2.430932	0.634573
43	O	4.654815	2.366922	0.245709	-5.097014	0.308782	-0.715008	4.994654	0.328863	1.015502	4.999434	0.289276	1.058777
44	H	5.311275	2.642976	0.909782	-5.688207	0.694093	-1.386680	5.523410	0.713427	1.737718	5.616621	0.728845	1.671371
45	H	3.791393	0.756206	-1.574631	-3.797353	1.048447	1.707710	3.900918	1.069749	-1.498602	3.799781	0.888864	-1.584395
		**Conformer 12**			**Conformer 13**			**Conformer 14**					
Row	Symbol	X	Y	Z	X	Y	Z	X	Y	Z			
1	C	2.466138	-1.070579	-0.170651	2.466218	-1.070425	-0.170634	-2.493581	-1.066058	-0.171340			
2	C	1.177604	1.399582	0.141778	1.177559	1.399661	0.141799	-1.182796	1.402867	-0.384004			
3	C	1.083625	-1.036676	0.180730	1.083700	-1.036599	0.180704	-1.086647	-1.033558	-0.406248			
4	C	3.206746	0.103966	-0.384672	3.206778	0.104168	-0.384601	-3.251040	0.109242	-0.034947			
5	C	2.541553	1.332366	-0.223950	2.541534	1.332541	-0.223875	-2.572779	1.337024	-0.134075			
6	C	0.464840	0.232449	0.341163	0.464848	0.232489	0.341130	-0.456043	0.234997	-0.517980			
7	H	0.690551	2.356867	0.273072	0.690422	2.356913	0.273056	-0.685468	2.359636	-0.473572			
8	C	0.334644	-2.282793	0.321676	0.334797	-2.282765	0.321617	-0.329123	-2.280248	-0.481008			
9	C	-0.960184	0.234834	0.841497	-0.960182	0.234779	0.841438	1.006314	0.236148	-0.895347			
10	O	3.295075	2.438788	-0.437241	3.295012	2.439006	-0.437043	-3.340184	2.444062	0.017449			
11	C	4.670028	0.040870	-0.760227	4.670074	0.041103	-0.760117	-4.738419	0.046458	0.229598			
12	H	4.848618	-0.877110	-1.327062	4.848602	-0.876632	-1.327378	-5.221133	0.903912	-0.248036			
13	H	4.904154	0.881510	-1.419734	4.904312	0.882039	-1.419202	-5.140935	-0.856328	-0.238351			
14	C	5.613663	0.075144	0.458157	5.613649	0.074677	0.458335	-5.090109	0.038191	1.730373			
15	H	5.426079	-0.772747	1.125033	5.425925	-0.773520	1.124780	-4.723819	0.943265	2.225593			
16	H	5.479983	0.996619	1.034033	5.480059	0.995895	1.034650	-4.647034	-0.827556	2.233306			
17	H	6.659030	0.026441	0.134162	6.659033	0.026008	0.134377	-6.175830	-0.008479	1.868855			
18	O	3.102542	-2.250403	-0.320778	3.102686	-2.250202	-0.320776	-3.139990	-2.245233	-0.064964			
19	H	2.425556	-2.959811	-0.138239	2.425734	-2.959665	-0.138286	-2.451868	-2.955404	-0.194686			
20	O	0.894158	-3.397277	0.218905	0.894376	-3.397199	0.218797	-0.895831	-3.394281	-0.422942			
21	C	2.699882	3.738704	-0.323368	2.699610	3.738883	-0.323696	-2.743260	3.744283	-0.084081			
22	H	1.883318	3.860760	-1.041762	3.497875	4.443577	-0.554177	-2.305214	3.899328	-1.075068			
23	H	3.498230	4.443367	-0.553651	2.335654	3.916657	0.693080	-1.983161	3.888452	0.690082			
24	H	2.336027	3.916164	0.693497	1.883084	3.860543	-1.042197	-3.559353	4.449610	0.069115			
25	C	-1.165677	-2.258262	0.594407	-1.165541	-2.258322	0.594326	1.188649	-2.256784	-0.628602			
26	H	-1.315674	-2.411945	1.667015	-1.315604	-2.412059	1.666916	1.427084	-2.411742	-1.684925			
27	H	-1.626829	-3.099457	0.072669	-1.626605	-3.099541	0.072539	1.604444	-3.097518	-0.069436			
28	C	-1.751222	-0.916140	0.188932	-1.751148	-0.916225	0.188826	1.738975	-0.914451	-0.177519			
29	O	-3.075588	-0.715572	0.645479	-3.075547	-0.715731	0.645261	3.096984	-0.714731	-0.521825			
30	O	-1.667144	-0.692022	-1.211435	-1.666971	-0.692149	-1.211550	1.538212	-0.687900	1.210631			
31	C	-2.425677	-1.578836	-2.046144	-2.425762	-1.578732	-2.046270	2.221731	-1.575069	2.107652			

32	H	-2.407650	-1.135926	-3.043121	-2.407103	-1.136108	-3.043363	2.120465	-1.130526	3.098910
33	H	-3.460060	-1.653776	-1.700145	-3.460300	-1.652957	-1.700580	3.281476	-1.652594	1.850481
34	H	-1.971228	-2.574547	-2.093284	-1.971913	-2.574740	-2.093002	1.762909	-2.569834	2.117658
35	O	-0.958000	-0.054591	2.257014	-0.957985	-0.054710	2.256945	1.122370	-0.053912	-2.306092
36	H	-0.404884	0.607914	2.695566	-0.405040	0.607916	2.695545	0.596590	0.600533	-2.788340
37	C	-1.911281	1.405810	0.576854	-1.911346	1.405715	0.576845	1.931848	1.407719	-0.553019
38	H	-1.799466	1.789336	-0.437960	-1.799571	1.789243	-0.437975	1.730620	1.798505	0.444990
39	H	-1.776659	2.225129	1.284598	-1.776768	2.225065	1.284570	1.861118	2.222044	-1.275626
40	C	-3.289224	0.708009	0.767234	-3.289223	0.707839	0.767264	3.321295	0.709470	-0.615045
41	C	-4.352817	1.244415	-0.179365	-4.352942	1.244300	-0.179165	4.290002	1.239436	0.432340
42	O	-4.528340	2.437924	-0.350841	-4.528554	2.437819	-0.350474	4.435385	2.430938	0.641439
43	O	-5.106899	0.297958	-0.756311	-5.107022	0.297869	-0.756159	5.002920	0.289515	1.054356
44	H	-5.778496	0.741168	-1.305824	-5.778713	0.741141	-1.305528	5.619447	0.728547	1.667998
45	H	-3.672876	0.884436	1.778514	-3.672763	0.884089	1.778616	3.800630	0.890485	-1.583846

Table S22: Energies and Boltzmann distribution of compound **21** (3*R*,4*R*,12*R*).

Conformer	Hartrees	kcal/mol	ΔG (kcal/mol)	Boltzmann Distribution (%)
Conformer 6	-1340.0038	-840865.258	0	23.4999251
Conformer 9	-1340.0038	-840865.257	0.00062751	23.4727737
Conformer 8	-1340.0034	-840865.011	0.24723879	14.9022690
Conformer 5	-1340.0034	-840865.005	0.25288637	14.7480229
Conformer 13	-1340.0034	-840865.005	0.25288637	14.7480229
Conformer 2	-1340.002	-840864.129	1.12888978	2.9366311
Conformer 7	-1340.002	-840864.129	1.12888978	2.9366311
Conformer 1	-1340.00194	-840864.095	1.16340281	2.7557242

Table S23: XYZ coordinates of compound **21** (3*R*,4*R*,12*R*).

		Conformer 1			Conformer 2			Conformer 5			Conformer 6		
Row	Symbol	X	Y	Z	X	Y	Z	X	Y	Z	X	Y	Z
1	C	-2.264061	-1.324625	-0.407797	-2.262134	-1.402306	0.211789	-2.155417	-1.311464	-0.617087	-2.182267	-1.431303	-0.065348
2	C	-1.258147	1.193963	0.250761	-1.253707	1.193712	0.39105	-1.168438	1.172787	0.183761	-1.188212	1.139322	0.369862
3	C	-0.870505	-1.167338	-0.121698	-0.848044	-1.190942	0.2783	-0.783412	-1.180847	-0.235624	-0.77675	-1.23242	0.107478
4	C	-3.15583	-0.245285	-0.374768	-3.174412	-0.340122	0.240694	-3.036714	-0.220955	-0.611207	-3.095694	-0.368481	-0.020694
5	C	-2.628157	1.011441	-0.028797	-2.642821	0.958631	0.332437	-2.518957	1.018139	-0.200107	-2.571953	0.916941	0.193489
6	C	-0.377719	0.123999	0.197078	-0.356189	0.137297	0.354746	-0.30017	0.091778	0.170637	-0.290195	0.083682	0.329461
7	H	-0.900563	2.176796	0.520839	-0.893283	2.208486	0.474497	-0.822142	2.145747	0.500311	-0.837653	2.146037	0.543646
8	C	-0.0024	-2.329195	-0.13937	0.044862	-2.333852	0.291838	0.086394	-2.339145	-0.295866	0.119338	-2.369158	0.02547
9	O	-3.523195	2.028212	0.011789	-3.554208	1.96116	0.359288	-3.399697	2.047523	-0.196592	-3.480578	1.921387	0.222589
10	C	-4.624784	-0.435322	-0.675201	-4.663706	-0.586134	0.165949	-4.483629	-0.383568	-1.015893	-4.575698	-0.602843	-0.216907
11	H	-5.005888	0.464751	-1.166299	-4.881765	-1.560129	0.612629	-4.821183	0.535847	-1.502851	-4.825999	-1.599928	0.155936
12	H	-4.734905	-1.263389	-1.380898	-5.178926	0.170328	0.76493	-4.554291	-1.188284	-1.752893	-5.132767	0.120403	0.385414
13	C	-5.470761	-0.723388	0.580811	-5.214371	-0.553726	-1.273485	-5.415145	-0.699678	0.171007	-5.01914	-0.485884	-1.688673
14	H	-5.404699	0.101395	1.297581	-4.740084	-1.321136	-1.893903	-5.389511	0.101893	0.916189	-4.499736	-1.218032	-2.315415
15	H	-5.133794	-1.636966	1.081318	-5.036529	0.419843	-1.74146	-5.121898	-1.632186	0.664085	-4.808013	0.512332	-2.085523
16	H	-6.524117	-0.854076	0.310085	-6.294219	-0.737768	-1.273511	-6.449014	-0.809299	-0.173885	-6.095897	-0.665827	-1.778528
17	O	-2.759877	-2.539652	-0.718013	-2.757301	-2.653701	0.127112	-2.641266	-2.506931	-1.004862	-2.671445	-2.667954	-0.282076
18	H	-1.992975	-3.17687	-0.684919	-1.973987	-3.271412	0.142411	-1.881545	-3.15275	-0.95191	-1.88913	-3.288268	-0.273612

19	O	-0.419328	-3.474943	-0.419188	-0.370585	-3.511853	0.224166	-0.325976	-3.469969	-0.639163	-0.293861	-3.536722	-0.15536
20	C	-3.08687	3.35408	0.341474	-3.118073	3.322743	0.473948	-2.968769	3.361018	0.186807	-3.052913	3.271171	0.452903
21	H	-2.354842	3.719248	-0.385525	-2.56604	3.481679	1.405534	-2.182522	3.727231	-0.480504	-2.570233	3.369464	1.430202
22	H	-2.665188	3.389049	1.350832	-4.0305	3.917935	0.483849	-2.619447	3.372696	1.223896	-3.963497	3.868903	0.433903
23	H	-3.983331	3.97168	0.298866	-2.501496	3.610851	-0.383154	-3.85135	3.992616	0.092255	-2.376319	3.609019	-0.338072
24	C	1.990012	-0.772478	-0.047529	1.983053	-0.755034	-0.097519	2.075368	-0.778703	-0.01498	2.067173	-0.739531	-0.116328
25	C	1.452621	-2.166816	0.230444	1.53072	-2.105252	0.434087	1.542101	-2.200046	0.081953	1.607285	-2.159379	0.178464
26	H	2.024354	-2.925588	-0.306762	2.04778	-2.923221	-0.070539	2.11814	-2.879197	-0.549773	2.114497	-2.878316	-0.468177
27	H	1.551201	-2.361509	1.304377	1.778437	-2.150634	1.500698	1.649574	-2.530717	1.120777	1.871883	-2.391855	1.215823
28	C	1.098098	0.341956	0.583173	1.151239	0.423298	0.498417	1.141243	0.248332	0.690277	1.202585	0.323297	0.623361
29	O	1.982743	-0.662976	-1.523261	1.773056	-0.845431	-1.559502	2.128646	-0.48762	-1.459311	1.906128	-0.57977	-1.573178
30	C	1.661695	1.694004	0.105199	1.602059	1.705347	-0.227483	1.751917	1.643332	0.453127	1.721412	1.706139	0.182901
31	H	1.102902	2.531972	0.526056	1.080156	2.58546	0.152714	1.162842	2.435118	0.91829	1.1736	2.521768	0.657455
32	H	2.696837	1.788472	0.436683	2.669258	1.856672	-0.058603	2.741915	1.656577	0.913093	2.764535	1.785745	0.494902
33	C	2.168201	0.533627	-2.119773	1.841097	0.262453	-2.327794	2.407445	0.769027	-1.873127	2.07746	0.640767	-2.129158
34	O	2.638298	0.586788	-3.239597	2.157507	0.171099	-3.497916	2.947542	0.946886	-2.946933	2.454846	0.73399	-3.280361
35	C	1.635251	1.781511	-1.417992	1.366315	1.585718	-1.73039	1.884955	1.938532	-1.038876	1.634561	1.868215	-1.332738
36	H	0.589195	1.846956	-1.758222	0.282218	1.588563	-1.928085	0.882712	2.121029	-1.459571	0.577565	1.988703	-1.620855
37	O	1.092348	0.18825	2.009816	1.345789	0.462971	1.919495	1.201993	-0.139419	2.069391	1.469462	0.063652	2.008046
38	C	2.092366	0.83343	2.829806	2.425387	1.228903	2.499567	0.5088	0.660076	3.043244	0.889094	0.931005	2.997412
39	H	3.093728	0.536647	2.507217	3.383234	0.905486	2.083801	0.910144	1.68101	3.047074	1.249437	1.957465	2.856387
40	H	1.998695	1.922002	2.739288	2.284104	2.293429	2.278538	-0.558992	0.710048	2.800026	-0.20321	0.933181	2.903897
41	C	1.833446	0.401368	4.263258	2.377124	0.993047	3.999709	0.717793	0.005584	4.3985	1.307581	0.407142	4.360944
42	H	1.93618	-0.683048	4.370109	2.529432	-0.064952	4.235017	1.781759	-0.042015	4.65036	2.397161	0.408765	4.46345
43	H	0.827867	0.690018	4.585027	1.41506	1.308064	4.415959	0.310325	-1.010121	4.408214	0.943374	-0.613293	4.515382
44	H	2.558906	0.88296	4.927194	3.169131	1.571441	4.486778	0.206622	0.589412	5.170806	0.886789	1.045592	5.144472
45	O	3.290064	-0.559425	0.395808	3.32404	-0.468322	0.129914	3.352005	-0.611793	0.508551	3.397212	-0.492929	0.202583
46	C	4.290273	-1.543531	0.071755	4.30094	-1.475885	-0.193256	4.369138	-1.55285	0.120406	4.384591	-1.440232	-0.242987
47	H	4.150076	-2.457127	0.656778	4.267798	-2.302261	0.522682	4.207568	-2.52657	0.592331	4.328089	-2.369583	0.331489
48	H	5.242698	-1.086668	0.340577	5.266628	-0.975569	-0.120809	5.306609	-1.12996	0.48179	5.346937	-0.962382	-0.059127
49	H	4.284342	-1.774	-0.996986	4.159133	-1.850408	-1.210658	4.414011	-1.663289	-0.966779	4.279633	-1.650243	-1.311171
50	O	2.352476	2.93147	-1.831608	1.981965	2.679714	-2.387709	2.700852	3.083835	-1.216491	2.376015	3.011486	-1.722933
51	H	2.518696	2.847107	-2.784182	2.026311	2.466792	-3.333722	2.919858	3.146941	-2.159759	2.457172	2.994538	-2.689832
		**Conformer 7**			**Conformer 8**			**Conformer 9**			**Conformer 13**		
Row	Symbol	X	Y	Z	X	Y	Z	X	Y	Z	X	Y	Z
1	C	-2.262134	1.402306	-0.211784	2.155318	1.311436	-0.617221	-2.182265	-1.431306	-0.065352	2.155417	1.311462	-0.617092
2	C	-1.253706	-1.193711	-0.391057	1.168255	-1.172665	0.184131	-1.188212	1.139316	0.369879	1.168437	-1.172787	0.183761
3	C	-0.848044	1.190942	-0.278295	0.783292	1.180899	-0.235783	-0.776748	-1.232423	0.107472	0.783411	1.180846	-0.235628
4	C	-3.174412	0.340122	-0.240696	3.036624	0.220913	-0.611013	-3.095692	-0.368486	-0.020688	3.036714	0.220953	-0.611209
5	C	-2.642821	-0.958631	-0.332444	2.518764	-1.018053	-0.19972	-2.571952	0.916934	0.193506	2.518957	-1.01814	-0.200108
6	C	-0.356189	-0.137296	-0.354748	0.300046	-0.0916	0.170837	-0.290193	0.083678	0.329467	0.30017	-0.091778	0.170635
7	H	-0.893281	-2.208485	-0.474508	0.821956	-2.145567	0.500816	-0.837654	2.146029	0.543677	0.822141	-2.145748	0.50031
8	C	0.044862	2.333853	-0.291825	-0.086569	2.339192	-0.296454	0.119343	-2.36916	0.025452	-0.086393	2.339145	-0.295871
9	O	-3.554207	-1.96116	-0.359301	3.399457	-2.047554	-0.196121	-3.480578	1.92138	0.222618	3.399696	-2.047525	-0.196592
10	C	-4.663706	0.586134	-0.165951	4.483558	0.383377	-1.015722	-4.575697	-0.602846	-0.216899	4.483629	0.383565	-1.015896
11	H	-4.881765	1.560127	-0.612633	4.821009	-0.536097	-1.502629	-4.825996	-1.599938	0.155926	4.821182	-0.53585	-1.502855
12	H	-5.178926	-0.17033	-0.764931	4.554292	1.188019	-1.752789	-5.132765	0.120388	0.385437	4.554291	1.188281	-1.752895
13	C	-5.21437	0.553728	1.273483	5.415141	0.699467	0.171139	-5.019145	-0.48586	-1.688661	5.415146	0.699673	0.171004
14	H	-4.740082	1.321138	1.8939	5.389466	-0.102046	0.916372	-4.499743	-1.217997	-2.315419	5.389512	-0.101898	0.916186
15	H	-5.036528	-0.419841	1.741459	5.122018	1.632027	0.664193	-4.808022	0.512362	-2.085494	5.1219	1.632182	0.664083
16	H	-6.294219	0.73777	1.273509	6.448998	0.808971	-0.173812	-6.095903	-0.665803	-1.778515	6.449015	0.809293	-0.173889
17	O	-2.757302	2.6537	-0.127102	2.6411	2.506786	-1.005453	-2.671443	-2.667957	-0.282086	2.641266	2.506928	-1.004869
18	H	-1.973987	3.271411	-0.142396	1.881392	3.152575	-0.952847	-1.889128	-3.288269	-0.273626	1.881546	3.152748	-0.951918
19	O	-0.370585	3.511854	-0.224146	0.325838	3.469918	-0.640022	-0.293855	-3.536722	-0.155389	0.325977	3.469968	-0.639169
20	C	-3.118072	-3.322742	-0.473964	2.968565	-3.360874	0.18763	-3.052912	3.271161	0.452938	2.968768	-3.361019	0.186809
21	H	-2.501496	-3.610853	0.383138	2.18228	-3.727339	-0.479508	-2.376319	3.609014	-0.338036	2.182521	-3.727233	-0.480502

22	H	-2.566038	-3.481676	-1.40555	2.619294	-3.372359	1.224744	-2.570232	3.369452	1.430237	2.619447	-3.372695	1.223898
23	H	-4.030499	-3.917935	-0.483867	3.8511	-3.99256	0.093224	-3.963496	3.868895	0.433941	3.85135	-3.992617	0.092258
24	C	1.983052	0.755033	0.097525	-2.075412	0.778638	-0.015369	2.067172	-0.739526	-0.116341	-2.075369	0.778703	-0.014981
25	C	1.53072	2.105253	-0.434076	-1.542349	2.200073	0.081061	1.60729	-2.159378	0.178441	-1.542101	2.200047	0.081949
26	H	2.047781	2.92322	0.070554	-2.118368	2.878858	-0.551076	2.1145	-2.878307	-0.46821	-2.11814	2.879195	-0.54978
27	H	1.778437	2.15064	-1.500687	-1.650066	2.53118	1.119714	1.871894	-2.391863	1.215796	-1.649574	2.530722	1.120771
28	C	1.151239	-0.423296	-0.498419	-1.141379	-0.248067	0.69041	1.202587	0.323294	0.623362	-1.141243	-0.24833	0.690278
29	O	1.773052	0.845423	1.559508	-2.128238	0.486886	-1.459683	1.906117	-0.579751	-1.57319	-2.128648	0.487617	-1.45931
30	C	1.602058	-1.705349	0.227476	-1.751947	-1.643171	0.453788	1.721413	1.70614	0.182908	-1.751918	-1.64333	0.453133
31	H	1.080156	-2.58546	-0.152728	-1.162944	-2.434648	0.919543	1.17361	2.521765	0.657479	-1.162842	-2.435115	0.918297
32	H	2.669258	-1.856673	0.058597	-2.742078	-1.656225	0.913476	2.76454	1.785737	0.494898	-2.741915	-1.656574	0.913101
33	C	1.841093	-0.262466	2.327793	-2.406652	-0.769932	-1.873074	2.07744	0.64079	-2.129161	-2.407447	-0.769031	-1.873123
34	O	2.157503	-0.171119	3.497917	-2.946126	-0.94827	-2.947172	2.454814	0.734024	-3.280368	-2.947545	-0.946892	-2.946928
35	C	1.36631	-1.585727	1.730382	-1.884445	-1.939077	-1.038086	1.634545	1.868231	-1.332727	-1.884959	-1.938535	-1.038869
36	H	0.282212	-1.58857	1.928074	-0.882107	-2.121798	-1.458408	0.577545	1.988716	-1.620833	-0.882717	-2.121036	-1.459565
37	O	1.345792	-0.462961	-1.919497	-1.202328	0.140161	2.069425	1.469488	0.063644	2.008042	-1.201986	0.139428	2.06939
38	C	2.425392	-1.228889	-2.499571	-0.509246	-0.658879	3.043652	0.889028	0.93091	2.997431	-0.508826	-0.660089	3.043249
39	H	3.383237	-0.90548	-2.083796	-0.910624	-1.679801	3.047991	1.249177	1.957433	2.856371	-0.910232	-1.680998	3.047106
40	H	2.284103	-2.293418	-2.278556	0.558565	-0.709065	2.800554	-0.203282	0.932891	2.903985	0.558959	-0.710131	2.800016
41	C	2.377139	-0.993015	-3.999711	-0.718169	-0.003805	4.398657	1.307693	0.407142	4.360945	-0.717756	-0.005556	4.398495
42	H	2.529451	0.064986	-4.235005	-1.782119	0.044007	4.650557	2.397278	0.408974	4.46339	-1.781716	0.042119	4.650368
43	H	1.415077	-1.308024	-4.415971	-0.310563	1.011848	4.408021	0.94369	-0.613361	4.515414	-0.310221	1.010122	4.408183
44	H	3.169147	-1.571406	-4.486782	-0.207043	-0.587467	5.171099	0.886822	1.04552	5.144489	-0.206614	-0.589403	5.170805
45	O	3.32404	0.468321	-0.129908	-3.352158	0.611848	0.507826	3.397212	-0.492924	0.202564	-3.352004	0.611795	0.508553
46	C	4.30094	1.475883	0.193266	-4.369256	1.552729	0.119236	4.384592	-1.440217	-0.243023	-4.369138	1.552851	0.120406
47	H	4.267805	2.302256	-0.522675	-4.207535	2.526777	0.590427	4.279635	-1.650209	-1.311211	-4.207567	2.526573	0.592329
48	H	5.266627	0.975564	0.120831	-5.306675	1.130176	0.481121	4.328093	-2.369578	0.331436	-5.306609	1.129962	0.481791
49	H	4.159125	1.850411	1.210667	-4.414329	1.662443	-0.968013	5.346937	-0.962368	-0.059153	-4.414012	1.663288	-0.966778
50	O	1.981955	-2.679728	2.387697	-2.700237	-3.084469	-1.215395	2.375988	3.011508	-1.722923	-2.70086	-3.083835	-1.216477
51	H	2.026301	-2.46681	3.333711	-2.919207	-3.147987	-2.158597	2.457159	2.994552	-2.68982	-2.919864	-3.146948	-2.159746

Table S24: Energies and Boltzmann distribution of compound **21** (3*R*,4*R*,12*S*).

Conformer	Hartrees	kcal/mol	ΔG (kcal/mol)	Boltzmann Distribution (%)
Conformer 2	-1340.003512	-840865.0786	0	52.19213357
Conformer 1	-1340.003394	-840865.0045	0.074046134	45.53666702
Conformer 4	-1339.999846	-840862.7781	2.300450223	0.75340828
Conformer 5	-1339.999789	-840862.7424	2.336218271	0.70536290
Conformer 8	-1339.999328	-840862.4531	2.6255002	0.41396164
Conformer 6	-1339.999295	-840862.4324	2.646208017	0.39846659

Table S25: XYZ coordinates of compound 21 (3*R*,4*R*,12*S*).

		**Conformer 1**			**Conformer 2**			**Conformer 4**			**Conformer 5**		
Row	Symbol	X	Y	Z	X	Y	Z	X	Y	Z	X	Y	Z
1	C	-2.070013	-1.460212	-0.241817	-2.072484	-1.259057	-0.726141	-2.109604	-1.501984	-0.133418	-2.146876	-1.270822	-0.650786
2	C	-1.099325	1.012018	0.619855	-1.086864	1.098255	0.399895	-1.177731	0.964311	0.786004	-1.191702	1.035155	0.601276
3	C	-0.662404	-1.257113	-0.105256	-0.689789	-1.159875	-0.380040	-0.703746	-1.247613	-0.080386	-0.747362	-1.127092	-0.396467
4	C	-2.998576	-0.445578	0.036352	-2.964987	-0.192769	-0.537343	-3.054208	-0.542701	0.258259	-3.071304	-0.274268	-0.306686
5	C	-2.486536	0.792753	0.453360	-2.446306	0.982704	0.027427	-2.561982	0.692995	0.708762	-2.567513	0.875596	0.322850

6	C	-0.189416	0.003207	0.353005	-0.211307	0.042937	0.208528	-0.251315	0.011261	0.399090	-0.284323	0.050457	0.250324
7	H	-0.761380	1.975906	0.967875	-0.745074	2.018775	0.847394	-0.852847	1.922187	1.161017	-0.859974	1.930282	1.103860
8	C	0.250474	-2.314518	-0.494217	0.202800	-2.258904	-0.693117	0.221810	-2.263116	-0.544295	0.165863	-2.166246	-0.831273
9	O	-3.408002	1.755774	0.695234	-3.334131	1.991592	0.197778	-3.501587	1.600639	1.068657	-3.488162	1.815602	0.646762
10	C	-4.482431	-0.677767	-0.131336	-4.422232	-0.318199	-0.917283	-4.536048	-0.828364	0.178332	-4.546061	-0.445611	-0.589085
11	H	-4.697344	-1.732087	0.063637	-4.786178	0.656913	-1.253388	-4.696264	-1.899086	0.332577	-4.979465	0.532339	-0.817651
12	H	-5.020348	-0.090731	0.618617	-4.507206	-1.007303	-1.762086	-5.043462	-0.301761	0.991678	-4.663553	-1.070482	-1.478752
13	C	-5.001311	-0.305603	-1.534372	-5.310757	-0.820489	0.237960	-5.163860	-0.410077	-1.166014	-5.319913	-1.081362	0.582814
14	H	-4.503751	-0.899306	-2.308023	-5.270662	-0.136601	1.091889	-4.697401	-0.943049	-2.000904	-5.244081	-0.465721	1.484891
15	H	-4.824702	0.752708	-1.751516	-4.990291	-1.810180	0.579293	-5.044075	0.664223	-1.339200	-4.930774	-2.077537	0.817180
16	H	-6.078529	-0.491415	-1.604714	-6.353918	-0.895674	-0.087649	-6.235316	-0.637778	-1.174669	-6.380814	-1.182945	0.329593
17	O	-2.548139	-2.648542	-0.658929	-2.558034	-2.393308	-1.266745	-2.569231	-2.690010	-0.573646	-2.618246	-2.383659	-1.247154
18	H	-1.755177	-3.237420	-0.805555	-1.788287	-3.025567	-1.337781	-1.766213	-3.240545	-0.794268	-1.828289	-2.969412	-1.419256
19	O	-0.149108	-3.445840	-0.851486	-0.209641	-3.342168	-1.166160	-0.159077	-3.392332	-0.927346	-0.230951	-3.228220	-1.362101
20	C	-2.990711	3.055541	1.136707	-2.901239	3.247047	0.740945	-3.104320	2.885246	1.568511	-3.075643	3.037558	1.274739
21	H	-3.909661	3.629486	1.250563	-2.527850	3.125207	1.762510	-2.519133	2.785144	2.488023	-3.989420	3.615547	1.408401
22	H	-2.348094	3.535347	0.392150	-3.789405	3.877859	0.750342	-4.034142	3.411025	1.782778	-2.380215	3.591785	0.637015
23	H	-2.473816	2.996508	2.099564	-2.132814	3.704091	0.110034	-2.533877	3.439772	0.817149	-2.619164	2.842819	2.250215
24	C	2.120867	-0.589008	-0.439427	2.147786	-0.670205	-0.282069	2.025991	-0.481755	-0.547555	2.062678	-0.529403	-0.440300
25	C	1.742190	-2.061664	-0.495200	1.691494	-2.109663	-0.468655	1.704982	-1.967279	-0.581822	1.656737	-1.978516	-0.655495
26	H	2.156230	-2.538793	-1.386695	2.200076	-2.575204	-1.316193	2.113228	-2.439160	-1.478249	2.148771	-2.399916	-1.534949
27	H	2.167978	-2.559287	0.381947	1.954176	-2.672370	0.432901	2.166814	-2.441950	0.290354	1.970136	-2.559487	0.218301
28	C	1.313160	0.176420	0.647077	1.241024	0.099517	0.720309	1.258154	0.250417	0.595522	1.197072	0.157430	0.660331
29	O	1.728827	-0.043514	-1.751803	1.981442	-0.041672	-1.605196	1.527719	0.037748	-1.838846	1.773887	0.134736	-1.729356
30	C	1.819623	1.632213	0.653579	1.829641	1.515757	0.875684	1.684599	1.727672	0.558395	1.718214	1.595826	0.818070
31	H	1.131426	2.306088	1.162382	1.117419	2.212361	1.316175	1.020770	2.350501	1.157340	1.023535	2.213157	1.387049
32	H	2.754394	1.639625	1.214677	2.671640	1.435074	1.563654	2.677108	1.810356	0.996409	2.648386	1.564521	1.381583
33	C	1.602874	1.288583	-1.900896	1.959938	1.301228	-1.698304	1.274815	1.351729	-1.974305	1.621760	1.470425	-1.769827
34	O	1.180253	1.742891	-2.947671	1.718424	1.830054	-2.767282	0.741772	1.764325	-2.987819	1.252401	2.006905	-2.798139
35	C	2.071248	2.194790	-0.761441	2.317997	2.120539	-0.457732	1.742037	2.304539	-0.872639	1.998573	2.288115	-0.533258
36	O	1.645363	-0.499597	1.866181	1.352765	-0.650776	1.936056	1.520900	-0.394766	1.851271	1.247713	-0.611001	1.872305
37	C	1.075074	0.006136	3.086272	0.643754	-0.162448	3.088659	2.704005	-0.083491	2.620871	2.336473	-0.459942	2.810847
38	H	1.377156	1.050065	3.237432	0.980316	0.852560	3.334407	3.594386	-0.141022	1.988619	3.296944	-0.532149	2.293093
39	H	-0.019551	-0.029293	3.030285	-0.431826	-0.125360	2.878053	2.623178	0.931462	3.028186	2.269653	0.519532	3.299484
40	C	1.583183	-0.863484	4.223394	0.933376	-1.110890	4.239333	2.774912	-1.096434	3.751435	2.189813	-1.568524	3.839869
41	H	2.674979	-0.824175	4.288673	2.004984	-1.144016	4.459249	2.875328	-2.113868	3.360896	2.275055	-2.553555	3.370586
42	H	1.278699	-1.905379	4.083435	0.593301	-2.124286	4.004355	1.877105	-1.049415	4.375790	1.221647	-1.506229	4.346688
43	H	1.168138	-0.507959	5.172002	0.407617	-0.770942	5.137416	3.643862	-0.881101	4.381976	2.978929	-1.475432	4.593296
44	O	3.470762	-0.349493	-0.219316	3.464414	-0.533264	0.137325	3.377173	-0.183284	-0.419085	3.400816	-0.344542	-0.114229
45	C	4.418323	-1.054078	-1.042580	4.475455	-1.263013	-0.581938	4.299439	-0.838862	-1.310893	4.389907	-0.991712	-0.937793
46	H	4.463939	-2.113647	-0.774387	4.410277	-2.335673	-0.377249	4.405734	-1.898459	-1.061376	4.395581	-2.072974	-0.772959
47	H	5.382736	-0.589608	-0.837013	5.426869	-0.883348	-0.209465	5.254683	-0.335935	-1.160917	5.346597	-0.574356	-0.623977
48	H	4.178918	-0.943424	-2.104207	4.405205	-1.079350	-1.658011	3.986025	-0.725512	-2.352464	4.224277	-0.771495	-1.996059
49	H	3.157299	2.280988	-0.914661	3.418092	2.134539	-0.452360	2.794171	2.506923	-1.125206	3.085199	2.433778	-0.632285
50	O	1.461397	3.469653	-0.884883	1.816765	3.440315	-0.596931	0.991795	3.506947	-0.933229	1.338386	3.543044	-0.573043
51	H	1.351881	3.649316	-1.833452	1.853712	3.667473	-1.540954	0.787025	3.673940	-1.868301	1.265928	3.802613	-1.506600

		**Conformer 6**			**Conformer 8**		
Row	Symbol	X	Y	Z	X	Y	Z
1	C	-2.076853	-1.457088	-0.229515	-2.098219	-1.213268	-0.750458
2	C	-1.075517	0.977745	0.698184	-1.076237	1.064000	0.499469
3	C	-0.664722	-1.244515	-0.174567	-0.698011	-1.118996	-0.480211
4	C	-2.994449	-0.474099	0.170987	-2.992453	-0.185063	-0.415596
5	C	-2.468283	0.745848	0.623113	-2.455700	0.950335	0.210854
6	C	-0.174341	-0.000724	0.312410	-0.198917	0.044449	0.169327
7	H	-0.724304	1.928179	1.069518	-0.718270	1.954266	0.993244
8	C	0.228954	-2.276003	-0.667463	0.184498	-2.181390	-0.924974

9	O	-3.380968	1.678194	0.987887	-3.345747	1.922845	0.523060
10	C	-4.483545	-0.719233	0.091894	-4.469506	-0.308458	-0.710312
11	H	-4.673085	-1.784766	0.248984	-4.868547	0.682563	-0.944840
12	H	-4.976125	-0.176711	0.903838	-4.599964	-0.931677	-1.599275
13	C	-5.099606	-0.287649	-1.253693	-5.273355	-0.915351	0.456755
14	H	-4.647722	-0.835455	-2.086926	-5.185459	-0.299718	1.357743
15	H	-4.950634	0.782540	-1.429663	-4.918698	-1.922908	0.697208
16	H	-6.176833	-0.486161	-1.261734	-6.334737	-0.983399	0.194629
17	O	-2.572041	-2.625168	-0.682609	-2.601702	-2.306308	-1.355871
18	H	-1.785906	-3.192869	-0.921369	-1.828966	-2.914013	-1.532267
19	O	-0.187319	-3.383042	-1.077361	-0.242871	-3.219461	-1.478853
20	C	-2.947653	2.953425	1.482284	-2.896199	3.137219	1.140708
21	H	-3.862389	3.503581	1.700076	-3.791254	3.745961	1.263830
22	H	-2.367329	3.490989	0.726227	-2.180261	3.661904	0.500721
23	H	-2.360478	2.840523	2.398979	-2.451108	2.937939	2.120489
24	C	2.086551	-0.553498	-0.660585	2.136440	-0.618985	-0.514378
25	C	1.721085	-2.028745	-0.696036	1.678242	-2.053276	-0.723910
26	H	2.134318	-2.501100	-1.591165	2.184197	-2.491022	-1.588555
27	H	2.144767	-2.528952	0.179383	1.933745	-2.648260	0.157383
28	C	1.343447	0.193360	0.489605	1.285513	0.100950	0.576892
29	O	1.605269	-0.006677	-1.920346	1.886041	0.054564	-1.779854
30	C	1.831684	1.654952	0.475277	1.871833	1.515145	0.753858
31	H	1.169611	2.318298	1.030433	1.183771	2.188679	1.263337
32	H	2.800350	1.670477	0.976054	2.755171	1.415445	1.385698
33	C	1.457816	1.325775	-2.058042	1.853377	1.400994	-1.825417
34	O	0.964968	1.773823	-3.075383	1.548524	1.960118	-2.861346
35	C	1.988587	2.231088	-0.947699	2.276211	2.177844	-0.579763
36	O	1.784988	-0.489188	1.672534	1.497916	-0.699493	1.749179
37	C	1.321718	0.004350	2.942585	0.890431	-0.262208	2.978438
38	H	1.633798	1.047715	3.075911	1.258746	0.736466	3.244310
39	H	0.226565	-0.033433	2.978921	-0.197612	-0.205271	2.855778
40	C	1.925600	-0.873888	4.024896	1.257744	-1.268238	4.055700
41	H	3.018920	-0.832237	3.999532	2.342728	-1.322520	4.188331
42	H	1.612313	-1.915294	3.901902	0.886164	-2.265695	3.801152
43	H	1.590730	-0.527700	5.008025	0.809403	-0.966688	5.007842
44	O	3.463846	-0.287950	-0.662129	3.514026	-0.466904	-0.298400
45	C	4.373693	-1.165685	0.025310	4.248909	-1.463116	0.435339
46	H	4.100962	-1.275894	1.076688	3.815851	-1.627852	1.423890
47	H	5.346833	-0.679739	-0.053318	5.253960	-1.052382	0.537870
48	H	4.424823	-2.144041	-0.462150	4.304753	-2.404073	-0.120523
49	H	3.061470	2.329097	-1.169732	3.374283	2.204831	-0.639004
50	O	1.357787	3.499486	-1.021554	1.753097	3.495138	-0.637091
51	H	1.202115	3.694637	-1.960392	1.750223	3.767916	-1.569509

Table S26: Energies and Boltzmann distribution of compound **21** (3*R*,4*S*,12*R*).

Conformer	Hartrees	kcal/mol	ΔG (kcal/mol)	Boltzmann Distribution (%)
Conformer 2	-1340.00669	-840867.075	0	21.5522789
Conformer 7	-1340.00666	-840867.051	0.02384537	20.6259843
Conformer 6	-1340.00661	-840867.019	0.05584836	19.4450609
Conformer 1	-1340.0066	-840867.014	0.06149594	19.2437945

Conformer 3	-1340.00659	-840867.011	0.06463349	19.1328814

Table S27: XYZ coordinates of compound **21** (3*R*,4*S*,12*R*).

		Conformer 1			Conformer 2			Conformer 3			Conformer 6		
Row	Symbol	X	Y	Z	X	Y	Z	X	Y	Z	X	Y	Z
1	C	2.529517	-1.048256	0.404447	-2.499275	1.181808	-0.006708	-2.529570	1.048204	0.404581	2.529476	-1.048244	0.404342
2	C	1.210080	1.319036	-0.280034	-1.207824	-1.249395	-0.492690	-1.210104	-1.319000	-0.280116	1.210007	1.319141	-0.279812
3	C	1.101699	-1.004750	0.408601	-1.082045	1.087850	0.145647	-1.101752	1.004722	0.408744	1.101663	-1.004750	0.408469
4	C	3.306113	0.067709	0.058341	-3.276752	0.087567	-0.415015	-3.306155	-0.067734	0.058384	3.306058	0.067772	0.058359
5	C	2.621479	1.242546	-0.295038	-2.605996	-1.122939	-0.657215	-2.621491	-1.242522	-0.295087	2.621395	1.242634	-0.294856
6	C	0.454307	0.214624	0.071786	-0.450925	-0.163025	-0.091081	-0.454342	-0.214608	0.071802	0.454252	0.214671	0.071890
7	H	0.726312	2.249471	-0.538568	-0.735906	-2.204179	-0.672267	-0.726346	-2.249408	-0.538772	0.726230	2.249612	-0.538203
8	C	0.347384	-2.213665	0.691248	-0.314269	2.277173	0.471498	-0.347440	2.213618	0.691519	0.347345	-2.213765	0.690795
9	O	3.403542	2.294169	-0.638926	-3.388900	-2.157030	-1.049082	-3.403528	-2.294142	-0.639095	3.403446	2.294318	-0.638659
10	C	4.815672	-0.007566	0.045412	-4.774635	0.211894	-0.573929	-4.815702	0.007541	0.045439	4.815616	-0.007481	0.045396
11	H	5.222544	0.967231	0.329200	-5.013427	1.238346	-0.865970	-5.222582	-0.967291	0.329097	5.222475	0.967260	0.329399
12	H	5.137949	-0.728185	0.802225	-5.101140	-0.442864	-1.386915	-5.137989	0.728071	0.802334	5.137898	-0.728262	0.802052
13	C	5.392578	-0.418630	-1.323690	-5.553098	-0.144390	0.707992	-5.392584	0.418766	-1.323619	5.392527	-0.418235	-1.323793
14	H	5.109599	0.298083	-2.101324	-5.269249	0.513287	1.536052	-5.109602	-0.297869	-2.101327	5.109534	0.298641	-2.101270
15	H	5.028916	-1.406931	-1.623213	-5.358620	-1.177916	1.012317	-5.028905	1.407094	-1.623034	5.028884	-1.406478	-1.623536
16	H	6.486443	-0.458640	-1.281024	-6.630683	-0.036367	0.543406	-6.486448	0.458781	-1.280950	6.486392	-0.458236	-1.281128
17	O	3.175637	-2.186216	0.727957	-3.131835	2.349142	0.226337	-3.175762	2.186121	0.728177	3.175617	-2.186249	0.727661
18	H	2.472146	-2.861075	0.940563	-2.427644	3.006255	0.486764	-2.472383	2.861064	0.940849	2.472176	-2.861156	0.940199
19	O	0.902765	-3.283708	1.027562	-0.859263	3.375813	0.721477	-0.902854	3.283636	1.027838	0.902712	-3.283790	1.027140
20	C	2.803857	3.547842	-0.994901	-2.798827	-3.429839	-1.348569	-2.803767	-3.547727	-0.995144	2.803725	3.547963	-0.994536
21	H	3.637290	4.211829	-1.221531	-2.079057	-3.345900	-2.168693	-3.637152	-4.211766	-1.221819	3.637119	4.212000	-1.221173
22	H	2.224909	3.957166	-0.161133	-3.628049	-4.067285	-1.653402	-2.224786	-3.957077	-0.161406	2.224795	3.957252	-0.160733
23	H	2.168068	3.441903	-1.879354	-2.314805	-3.856015	-0.464341	-2.167980	-3.441698	-1.879593	2.167892	3.442072	-1.878968
24	C	-1.666191	-1.049141	-0.292289	1.742919	1.010743	-0.268529	1.666152	1.049237	-0.292029	-1.666134	-1.049006	-0.292725
25	C	-1.162115	-2.198702	0.571501	1.198253	2.205798	0.504261	1.162067	2.198619	0.571975	-1.162126	-2.198906	0.570640
26	H	-1.576684	-2.081908	1.576845	1.506054	2.109372	1.549286	1.576477	2.081509	1.577353	-1.576971	-2.082717	1.575937
27	H	-1.496687	-3.166655	0.192722	1.605027	3.148144	0.131720	1.496773	3.166635	0.193482	-1.496461	-3.166710	0.191261
28	C	-1.070112	0.300870	0.193353	1.047848	-0.299786	0.193158	1.070077	-0.300877	0.193265	-1.070161	0.300822	0.193490
29	O	-3.121046	-1.077999	-0.162095	3.176899	0.991795	0.010861	3.121007	1.078055	-0.161849	-3.121023	-1.077980	-0.162742
30	C	-1.727677	1.427770	-0.617543	1.741275	-1.476950	-0.508941	1.727620	-1.427534	-0.617996	-1.727766	1.428052	-0.616885
31	H	-1.369005	2.412006	-0.310059	1.313649	-2.436549	-0.211857	1.368919	-2.411864	-0.310848	-1.369101	2.412139	-0.308886
32	H	-1.492061	1.302375	-1.676518	1.622709	-1.379362	-1.590161	1.492033	-1.301778	-1.676932	-1.492162	1.303223	-1.675931
33	C	-3.857747	-0.009837	-0.535396	3.905604	-0.114750	-0.248923	3.857714	0.009978	-0.535349	-3.857755	-0.009681	-0.535591
34	O	-5.024397	-0.161611	-0.842435	5.102812	-0.015811	-0.436984	5.024393	0.161797	-0.842281	-5.024373	-0.161375	-0.842801
35	C	-3.240839	1.383918	-0.419503	3.226005	-1.480740	-0.153916	3.240780	-1.383775	-0.419906	-3.240932	1.384046	-0.418895
36	H	-3.471635	1.682283	0.615123	3.336335	-1.750351	0.908115	3.471526	-1.682499	0.614628	-3.471804	1.681831	0.615875
37	O	-1.460530	0.364747	1.578230	1.289436	-0.328547	1.612681	1.460591	-0.365160	1.578109	-1.460605	0.364099	1.578404
38	C	-0.977037	1.465333	2.368552	0.688672	-1.385921	2.381077	0.976977	-1.465840	2.368208	-0.977420	1.464526	2.369092
39	H	0.118969	1.461418	2.381703	-0.401474	-1.353212	2.271785	-0.119022	-1.461399	2.381915	0.118601	1.461556	2.381486
40	H	-1.308903	2.418618	1.937340	1.037806	-2.362629	2.022009	1.308172	-2.419114	1.936457	-1.310400	2.417818	1.938746
41	C	-1.533476	1.297211	3.772171	1.091500	-1.180352	3.831438	1.534180	-1.298572	3.771627	-1.532719	1.295044	3.773000
42	H	-1.196626	0.354497	4.214288	0.735847	-0.213668	4.201117	1.198078	-0.355839	4.214269	-1.194607	0.352422	4.214340
43	H	-2.627805	1.308298	3.764289	2.179231	-1.219650	3.945686	2.628497	-1.310302	3.763248	-2.627065	1.305031	3.765873
44	H	-1.185290	2.119055	4.406022	0.651597	-1.969093	4.450142	1.185789	-2.120463	4.405312	-1.184935	2.116897	4.407058
45	O	-1.341682	-1.143649	-1.648079	1.563220	1.071009	-1.652895	1.341653	1.144097	-1.647790	-1.341507	-1.142948	-1.648510
46	C	-1.676284	-2.359930	-2.340851	2.014985	2.250142	-2.343468	1.676268	2.360610	-2.340140	-1.675931	-2.358970	-2.341772
47	H	-1.000598	-3.174081	-2.062080	1.345666	3.096690	-2.163114	1.000408	3.174583	-2.061265	-1.000162	-3.173158	-2.063296
48	H	-1.547540	-2.134827	-3.399662	1.989051	1.993120	-3.402541	1.547804	2.135784	-3.399045	-1.547181	-2.133439	-3.400491
49	H	-2.713475	-2.649677	-2.152619	3.037252	2.509722	-2.055773	2.713383	2.650421	-2.151578	-2.713095	-2.648932	-2.153700

50	O	-3.855049	2.275219	-1.334764	3.896294	-2.425405	-0.971001	3.854989	-2.274788	-1.335442	-3.855111	2.275811	-1.333703
51	H	-4.807975	2.091832	-1.329830	4.850036	-2.276958	-0.871047	4.807864	-2.091134	-1.330652	-4.808030	2.092449	-1.328898

			**Conformer 7**	
Row	Symbol	X	Y	Z
1	C	2.499444	-1.181994	-0.005532
2	C	1.208418	1.249336	-0.492028
3	C	1.082140	-1.087845	0.146296
4	C	3.277152	-0.087957	-0.413958
5	C	2.606613	1.122596	-0.656424
6	C	0.451188	0.163037	-0.090947
7	H	0.736840	2.204275	-0.671548
8	C	0.314172	-2.277051	0.472007
9	O	3.389732	2.156452	-1.048367
10	C	4.775045	-0.212445	-0.572685
11	H	5.013915	-1.239427	-0.862737
12	H	5.101385	0.440746	-1.387021
13	C	5.553577	0.146614	0.708421
14	H	5.270064	-0.509510	1.537830
15	H	5.358776	1.180669	1.010710
16	H	6.631172	0.038624	0.543887
17	O	3.131767	-2.349379	0.227954
18	H	2.427412	-3.006625	0.487836
19	O	0.859080	-3.375790	0.721939
20	C	2.799951	3.429305	-1.348574
21	H	2.316347	3.856176	-0.464465
22	H	2.079933	3.344971	-2.168426
23	H	3.629331	4.066210	-1.654084
24	C	-1.742728	-1.010930	-0.269009
25	C	-1.198323	-2.205474	0.504731
26	H	-1.506003	-2.108020	1.549713
27	H	-1.605348	-3.148034	0.133045
28	C	-1.047788	0.299815	0.192273
29	O	-3.176707	-0.991545	0.010149
30	C	-1.740862	1.476366	-0.511307
31	H	-1.312708	2.436329	-0.216377
32	H	-1.623017	1.376777	-1.592415
33	C	-3.905113	0.115122	-0.249901
34	O	-5.102477	0.016560	-0.437297
35	C	-3.224991	1.480875	-0.154766
36	H	-3.333970	1.749641	0.907722
37	O	-1.290693	0.329189	1.611433
38	C	-0.690606	1.386343	2.380414
39	H	0.399742	1.352911	2.273410
40	H	-1.038465	2.363240	2.020466
41	C	-1.096936	1.180831	3.829688
42	H	-0.743451	0.213407	4.199561
43	H	-2.184897	1.221440	3.941347
44	H	-0.657417	1.968622	4.449845
45	O	-1.562572	-1.072279	-1.653252
46	C	-2.014246	-2.251959	-2.343017
47	H	-1.345076	-3.098439	-2.161794
48	H	-1.987959	-1.995795	-3.402290
49	H	-3.036635	-2.511184	-2.055437
50	O	-3.895328	2.426328	-0.970566
51	H	-4.849050	2.279192	-0.868842

Table S28: Energies and Boltzmann distribution of compound **21** (3*R*,4*S*,12*S*).

Conformer	Hartrees	kcal/mol	ΔG (kcal/mol)	Boltzmann Distribution (%)
Conformer 11	-1340.00663	-840867.035	0	27.9746756
Conformer 1	-1340.00662	-840867.028	0.0062751	27.6531365
Conformer 2	-1340.00643	-840866.907	0.12738445	22.1230816
Conformer 4	-1340.00642	-840866.906	0.12863947	22.0719899
Conformer 14	-1340.00121	-840863.633	3.40172959	0.0530963
Conformer 13	-1340.00111	-840863.569	3.46573557	0.0471904
Conformer 10	-1340.00102	-840863.514	3.52095641	0.0426257
Conformer 9	-1340.00062	-840863.262	3.77258776	0.0268129
Conformer 7	-1339.99892	-840862.2	4.83496153	0.0037874
Conformer 6	-1339.99888	-840862.173	4.86194444	0.0036038

Table S29: XYZ coordinates of compound **21** (3*R*,4*S*,12*S*).

		**Conformer 1**			**Conformer 2**			**Conformer 4**			**Conformer 6**		
Row	Symbol	X	Y	Z	X	Y	Z	X	Y	Z	X	Y	Z
1	C	-2.526135	1.187586	0.076701	-2.548456	1.037103	0.472295	2.548464	-1.037115	0.472247	-2.610055	1.138348	0.037677
2	C	-1.227624	-1.206995	-0.540518	-1.221834	-1.291423	-0.314064	1.221848	1.291449	-0.314035	-1.300837	-1.291463	-0.397767
3	C	-1.102312	1.105517	0.177153	-1.119652	1.015171	0.425582	1.119658	-1.015158	0.425553	-1.185927	1.069240	0.147491
4	C	-3.306812	0.097472	-0.333331	-3.320834	-0.083779	0.136377	3.320845	0.083773	0.136347	-3.384685	0.018947	-0.295014
5	C	-2.632300	-1.097612	-0.636374	-2.632284	-1.241400	-0.264592	2.632297	1.241411	-0.264571	-2.705062	-1.194429	-0.502462
6	C	-0.464090	-0.123849	-0.137097	-0.466565	-0.181838	0.028209	0.466571	0.181853	0.028181	-0.544537	-0.176058	-0.082545
7	H	-0.751427	-2.147894	-0.775849	-0.734017	-2.206507	-0.617055	0.734047	2.206547	-0.617008	-0.819935	-2.247210	-0.548295
8	C	-0.341864	2.283237	0.554759	-0.374099	2.220355	0.740871	0.374107	-2.220332	0.740860	-0.433031	2.265634	0.479251
9	O	-3.418896	-2.130759	-1.022698	-3.410899	-2.300053	-0.593827	3.410917	2.300086	-0.593747	-3.488346	-2.257619	-0.806873
10	C	-4.810937	0.208439	-0.430815	-4.830614	-0.032203	0.182349	4.830625	0.032181	0.182304	-4.889319	0.115291	-0.402715
11	H	-5.071544	1.239693	-0.684747	-5.211592	-1.018822	0.461024	5.211615	1.018789	0.461002	-5.154719	1.123804	-0.732063
12	H	-5.161776	-0.427622	-1.248491	-5.134570	0.668051	0.965391	5.134583	-0.668098	0.965323	-5.235688	-0.580664	-1.172174
13	C	-5.535441	-0.191702	0.869691	-5.464795	0.397128	-1.155477	5.464788	-0.397116	-1.155541	-5.614740	-0.192013	0.922222
14	H	-5.227193	0.446705	1.704056	-5.201091	-0.299562	-1.957715	5.201080	0.299599	-1.957755	-5.311582	0.507647	1.707916
15	H	-5.318316	-1.230704	1.137787	-5.126963	1.396608	-1.448029	5.126947	-1.396586	-1.448119	-5.392433	-1.207173	1.266563
16	H	-6.619637	-0.092047	0.749237	-6.556708	0.419607	-1.070997	6.556702	-0.419605	-1.071075	-6.699290	-0.107529	0.793095
17	O	-3.162128	2.339322	0.369898	-3.198563	2.158603	0.841757	3.198576	-2.158604	0.841753	-3.252002	2.305231	0.251599
18	H	-2.456211	2.996719	0.625577	-2.497812	2.840476	1.041454	2.497882	-2.840516	1.041531	-2.551361	2.978131	0.476844
19	O	-0.890070	3.370938	0.842010	-0.934673	3.279330	1.102251	0.934702	-3.279313	1.102179	-0.985456	3.365645	0.703711
20	C	-2.827092	-3.387039	-1.382668	-2.806353	-3.535929	-1.000358	2.806379	3.535988	-1.000185	-2.891613	-3.538305	-1.054227
21	H	-2.148639	-3.271947	-2.233667	-2.181198	-3.948746	-0.202526	2.181211	3.948749	-0.202332	-2.363400	-3.903031	-0.167741
22	H	-3.661477	-4.028411	-1.664227	-2.215408	-3.402493	-1.911778	2.215442	3.402634	-1.911624	-2.208564	-3.495061	-1.908279
23	H	-2.295705	-3.828286	-0.533794	-3.637548	-4.211383	-1.199619	3.637571	4.211465	-1.199386	-3.722650	-4.204280	-1.283936
24	C	1.737081	1.089828	-0.249263	1.657473	1.126549	-0.293831	-1.657477	-1.126542	-0.293793	1.637132	1.043109	-0.252030
25	C	1.165312	2.197330	0.627294	1.134453	2.200547	0.652316	-1.134449	-2.200491	0.652401	1.075839	2.186381	0.578517
26	H	1.433433	1.976860	1.665220	1.520949	1.984631	1.653165	-1.520884	-1.984476	1.653255	1.331679	2.011389	1.628501
27	H	1.593080	3.171360	0.382295	1.490877	3.194576	0.375379	-1.490924	-3.194527	0.375559	1.506710	3.148350	0.294523
28	C	1.050433	-0.272981	0.042394	1.063401	-0.267011	0.050056	-1.063398	0.267028	0.050016	0.969567	-0.306836	0.138325
29	O	3.147432	1.015783	0.127383	3.101683	1.111535	-0.067597	-3.101690	-1.111501	-0.067527	3.066372	1.005305	0.053762
30	C	1.683582	-1.345518	-0.864462	1.644953	-1.304716	-0.928660	-1.644983	1.304716	-0.928717	1.625161	-1.440374	-0.684732

31	H	1.521076	-2.335020	-0.432561	1.565566	-2.303576	-0.494837	-1.565659	2.303573	-0.494881	1.621204	-2.361540	-0.097496
32	H	1.214355	-1.330395	-1.848110	1.079412	-1.300386	-1.860345	-1.079435	1.300433	-1.860399	1.035093	-1.624071	-1.581592
33	C	3.826454	-0.124561	-0.091954	3.802839	0.002928	-0.365536	-3.802823	-0.002905	-0.365510	3.788932	-0.063687	-0.337831
34	O	4.941341	-0.266180	0.375455	4.965477	-0.090880	-0.017587	-4.965432	0.090997	-0.017478	4.977369	-0.113732	-0.085163
35	C	3.205635	-1.159595	-1.024915	3.134591	-1.055473	-1.237701	-3.134606	1.055394	-1.237804	3.078619	-1.159058	-1.128227
36	O	1.399164	-0.519972	1.418404	1.561203	-0.508761	1.380340	-1.561181	0.508744	1.380310	1.046211	-0.517861	1.561295
37	C	0.729882	-1.580807	2.122061	1.014701	-1.604482	2.134558	-1.014780	1.604540	2.134489	2.261833	-0.939693	2.213612
38	H	-0.330713	-1.332167	2.243821	-0.038924	-1.406750	2.363320	0.038881	1.406934	2.363202	2.764233	-1.718593	1.628511
39	H	0.796235	-2.518737	1.556936	1.068009	-2.534254	1.554786	-1.068240	2.534304	1.554718	2.944590	-0.089524	2.321026
40	C	1.408203	-1.728732	3.473450	1.830093	-1.729515	3.410326	-1.830129	1.729483	3.410295	1.875160	-1.483858	3.578819
41	H	1.347484	-0.796656	4.043835	1.431011	-2.545027	4.022061	-1.782207	0.805723	3.995240	1.218939	-2.354081	3.482128
42	H	2.462149	-1.998750	3.356272	1.782292	-0.805754	3.995280	-2.878810	1.947147	3.185893	1.358864	-0.720081	4.168591
43	H	0.911530	-2.516821	4.048591	2.878743	-1.947287	3.185881	-1.431107	2.545034	4.022022	2.776258	-1.786125	4.122429
44	O	1.616984	1.298510	-1.623512	1.385243	1.341258	-1.645052	-1.385257	-1.341319	-1.645000	1.456687	1.175793	-1.631803
45	C	2.119658	2.534916	-2.163106	1.766632	2.606448	-2.216338	-1.766578	-2.606569	-2.216181	1.899176	2.395689	-2.257340
46	H	1.462681	3.373047	-1.913099	1.098945	3.407967	-1.886908	-1.098837	-3.408025	-1.886702	1.230420	3.227821	-2.019033
47	H	2.128982	2.397293	-3.244362	1.668661	2.479456	-3.294494	-1.668632	-2.479650	-3.294348	1.862565	2.201823	-3.329298
48	H	3.135671	2.734164	-1.811420	2.803020	2.853833	-1.970479	-2.802949	-2.854002	-1.970290	2.924101	2.641027	-1.965249
49	O	3.859007	-2.408331	-0.863123	3.851291	-2.276502	-1.148381	-3.851407	2.276385	-1.148726	3.822779	-2.364055	-1.057881
50	H	4.780366	-2.223109	-0.615973	4.785269	-2.053649	-0.999374	-4.785246	2.053452	-0.998899	4.764549	-2.127412	-1.038747
51	H	3.403115	-0.761360	-2.031858	3.215091	-0.648006	-2.257116	-3.215017	0.647773	-2.257164	3.061811	-0.784462	-2.161539
		**Conformer 7**			**Conformer 9**			**Conformer 10**			**Conformer 11**		
Row	Symbol	X	Y	Z	X	Y	Z	X	Y	Z	X	Y	Z
1	C	2.621821	-1.031979	0.432369	-2.498300	1.202657	-0.089807	-2.526543	1.103368	0.316522	-2.526253	1.187370	0.077070
2	C	1.293699	1.357733	-0.144844	-1.242551	-1.265721	-0.457822	-1.248876	-1.320532	-0.229230	-1.227589	-1.207027	-0.540678
3	C	1.192365	-1.003452	0.401084	-1.077404	1.101289	0.052277	-1.096347	1.040885	0.309755	-1.102402	1.105381	0.177337
4	C	3.393350	0.110490	0.180759	-3.293785	0.095457	-0.411774	-3.320732	-0.023307	0.066988	-3.306857	0.097260	-0.333166
5	C	2.703573	1.300910	-0.109992	-2.641799	-1.139843	-0.580287	-2.656217	-1.233628	-0.203520	-2.632280	-1.097706	-0.636442
6	C	0.541887	0.221729	0.099535	-0.461240	-0.161421	-0.155829	-0.470796	-0.199244	0.012659	-0.464107	-0.123883	-0.137150
7	H	0.803080	2.297956	-0.351285	-0.775672	-2.229853	-0.600396	-0.773382	-2.267390	-0.441966	-0.751358	-2.147861	-0.776174
8	C	0.444452	-2.219406	0.666830	-0.310694	2.276368	0.430589	-0.326919	2.230297	0.633758	-0.342018	2.283131	0.555050
9	O	3.482702	2.383625	-0.349296	-3.446655	-2.188900	-0.873744	-3.458176	-2.299079	-0.440377	-3.418813	-2.130860	-1.022935
10	C	4.903551	0.053622	0.207942	-4.793502	0.225524	-0.547348	-4.829286	0.068121	0.077748	-4.811002	0.208216	-0.430584
11	H	5.288843	1.009831	0.573464	-5.028832	1.234123	-0.898157	-5.239441	-0.874304	0.451888	-5.071605	1.239395	-0.684841
12	H	5.213299	-0.716163	0.920188	-5.142778	-0.475401	-1.310956	-5.128792	0.854527	0.775976	-5.161903	-0.428097	-1.248028
13	C	5.525775	-0.250482	-1.169176	-5.548549	-0.042337	0.769605	-5.429656	0.366825	-1.310264	-5.535417	-0.191478	0.870114
14	H	5.257169	0.518018	-1.901173	-5.243430	0.663795	1.548676	-5.170200	-0.417353	-2.028724	-5.227083	0.447191	1.704243
15	H	5.183150	-1.217912	-1.550606	-5.355811	-1.056207	1.134748	-5.061836	1.321245	-1.700814	-5.318305	-1.230400	1.138529
16	H	6.618362	-0.282567	-1.096619	-6.628307	0.064547	0.619294	-6.521902	0.423678	-1.249771	-6.619621	-0.091835	0.749724
17	O	3.272217	-2.180736	0.709970	-3.116818	2.386941	0.093015	-3.154560	2.267402	0.579589	-3.162305	2.338961	0.370516
18	H	2.571914	-2.870698	0.877165	-2.406210	3.048359	0.319618	-2.442706	2.943606	0.752113	-2.456432	2.996375	0.626272
19	O	1.002929	-3.301648	0.953905	-0.841108	3.394874	0.606673	-0.866037	3.329713	0.886626	-0.890289	3.370698	0.842637
20	C	2.877027	3.651926	-0.635860	-2.879134	-3.491007	-1.075741	-2.879698	-3.585839	-0.701499	-2.826985	-3.387129	-1.382757
21	H	2.259431	3.990630	0.201787	-2.186277	-3.490127	-1.922864	-2.273766	-3.565821	-1.612621	-2.295539	-3.828279	-0.533865
22	H	2.277583	3.602992	-1.550290	-3.724025	-4.142875	-1.294910	-3.724946	-4.259142	-0.839471	-2.148555	-3.272155	-2.233796
23	H	3.707557	4.342117	-0.780038	-2.369590	-3.840911	-0.172615	-2.275094	-3.923542	0.145865	-3.661334	-4.028593	-1.664216
24	C	-1.574338	-1.063774	-0.279705	1.786459	1.022283	-0.160437	1.731230	1.050929	-0.196400	1.736891	1.090028	-0.249652
25	C	-1.068429	-2.182736	0.616813	1.177928	2.141100	0.671751	1.181385	2.132314	0.721409	1.165214	2.197478	0.627026
26	H	-1.431792	-2.005313	1.634167	1.331792	1.945488	1.736111	1.446786	1.915776	1.759377	1.433761	1.977228	1.664879
27	H	-1.442406	-3.159172	0.302923	1.659819	3.098328	0.463674	1.614615	3.107550	0.491652	1.592674	3.171581	0.381757
28	C	-0.990052	0.308840	0.161719	1.049174	-0.347117	-0.006546	1.051547	-0.342877	-0.001303	1.050460	-0.272864	0.042149
29	O	-3.027991	-1.065662	-0.123807	3.152339	0.859209	0.311739	3.142597	0.918072	0.128503	3.147394	1.016166	0.126743
30	C	-1.589881	1.415677	-0.737230	1.663350	-1.300078	-1.050120	1.579494	-1.253710	-1.126694	1.683591	-1.345295	-0.864826
31	H	-1.677201	2.340224	-0.161833	1.429278	-2.324839	-0.757951	1.405875	-2.291246	-0.837517	1.520527	-2.334897	-0.433366
32	H	-0.914414	1.612957	-1.568524	1.242412	-1.109758	-2.036072	1.052741	-1.054605	-2.058621	1.214733	-1.329577	-1.848634
33	C	-3.736288	-0.022911	-0.603094	3.802039	-0.280122	-0.011401	3.785526	-0.194533	-0.288071	3.826430	-0.124249	-0.092087

34	O	-4.945570	-0.006164	-0.476626	4.879125	-0.523803	0.498150	4.915245	-0.418700	0.102473	4.941314	-0.265630	0.375420
35	C	-2.978116	1.084312	-1.329616	3.201891	-1.171542	-1.100414	3.100319	-1.077385	-1.332973	3.205751	-1.159832	-1.024616
36	O	-1.219056	0.529163	1.566691	1.390901	-0.994132	1.231990	1.538603	-1.006374	1.178566	1.399471	-0.519898	1.418047
37	C	-2.507151	0.919406	2.086168	0.897116	-0.538119	2.505519	1.165722	-0.595267	2.507348	0.730399	-1.580783	2.121737
38	H	-2.966893	1.681617	1.446469	1.552899	0.250266	2.894801	1.832406	0.207145	2.846245	-0.330170	-1.332179	2.243871
39	H	-3.173857	0.051021	2.128120	-0.115021	-0.133669	2.399231	0.135208	-0.224716	2.516526	0.796552	-2.518652	1.556468
40	C	-2.279522	1.479781	3.480368	0.899303	-1.726711	3.453130	1.305640	-1.803082	3.419605	1.409111	-1.728919	3.472919
41	H	-1.641017	2.367713	3.446493	1.904523	-2.150459	3.541466	2.328053	-2.192705	3.393197	1.348634	-0.796892	4.043416
42	H	-1.806564	0.733148	4.125831	0.219655	-2.508651	3.101290	0.619279	-2.600804	3.120102	2.463006	-1.998994	3.355385
43	H	-3.240215	1.758812	3.925406	0.574720	-1.404484	4.448027	1.075680	-1.514026	4.450426	0.912555	-2.517027	4.048117
44	O	-1.247668	-1.203429	-1.631656	1.776839	1.305393	-1.533753	1.571926	1.363929	-1.554012	1.616593	1.298661	-1.623846
45	C	-1.580456	-2.444917	-2.282193	2.372959	2.538836	-1.977534	2.083437	2.625070	-2.024892	2.119133	2.534982	-2.163653
46	H	-0.908379	-3.248662	-1.967852	1.720818	3.393356	-1.774404	1.433302	3.454406	-1.731280	1.462871	3.373379	-1.912654
47	H	-1.444727	-2.259845	-3.347721	2.492912	2.430336	-3.055657	2.091308	2.545880	-3.112160	2.127073	2.397667	-3.244955
48	H	-2.619508	-2.726323	-2.089661	3.352210	2.693412	-1.516053	3.101421	2.798222	-1.664846	3.135656	2.733681	-1.813125
49	O	-3.760521	2.266393	-1.358616	3.791324	-2.460650	-1.039656	3.728742	-2.348812	-1.368893	3.858823	-2.408530	-0.861589
50	H	-4.691010	2.001221	-1.443283	4.704629	-2.347997	-0.728065	4.665341	-2.216006	-1.147317	4.780050	-2.223318	-0.613974
51	H	-2.838331	0.697442	-2.348916	3.483148	-0.675209	-2.041015	3.267951	-0.550007	-2.283754	3.403889	-0.762354	-2.031737

		Conformer 13			Conformer 14		
Row	Symbol	X	Y	Z	X	Y	Z
1	C	2.495528	-1.229698	0.074514	2.529044	-1.075619	0.458857
2	C	1.189438	1.169457	-0.506899	1.182680	1.253262	-0.290467
3	C	1.074759	-1.144035	0.208806	1.099581	-1.052771	0.452792
4	C	3.269229	-0.141113	-0.353071	3.292702	0.044632	0.100787
5	C	2.590980	1.055754	-0.639516	2.594117	1.202030	-0.282462
6	C	0.432517	0.088395	-0.085157	0.435979	0.144953	0.074946
7	H	0.710456	2.111275	-0.731896	0.687370	2.167497	-0.583136
8	C	0.319774	-2.322975	0.594910	0.362788	-2.259257	0.785264
9	O	3.370286	2.086039	-1.046703	3.363546	2.259057	-0.636615
10	C	4.770086	-0.256224	-0.487364	4.803088	-0.008075	0.104647
11	H	5.021584	-1.287995	-0.748212	5.192183	0.978134	0.373381
12	H	5.102575	0.379585	-1.312825	5.128167	-0.709074	0.878461
13	C	5.526741	0.141087	0.795606	5.399639	-0.436656	-1.250632
14	H	5.236752	-0.496871	1.636811	5.113937	0.260689	-2.044692
15	H	5.319176	1.180571	1.069343	5.053747	-1.435891	-1.534413
16	H	6.607384	0.038277	0.648989	6.493437	-0.459303	-1.196305
17	O	3.135186	-2.383240	0.350520	3.188550	-2.197262	0.809554
18	H	2.433823	-3.039618	0.621320	2.493484	-2.879017	1.028879
19	O	0.872578	-3.411806	0.868221	0.933404	-3.317774	1.131445
20	C	2.773458	3.343439	-1.394485	2.748860	3.495174	-1.027064
21	H	2.072742	3.228404	-2.227225	2.150156	3.911204	-0.210859
22	H	3.602384	3.981164	-1.699422	2.129216	3.360757	-1.919061
23	H	2.266084	3.788702	-0.533182	3.574693	4.168400	-1.254127
24	C	-1.768566	-1.121232	-0.168818	-1.690608	-1.165339	-0.201824
25	C	-1.185752	-2.236802	0.689780	-1.147001	-2.240402	0.731029
26	H	-1.439745	-2.024751	1.733032	-1.511748	-2.025162	1.740204
27	H	-1.617235	-3.208715	0.442861	-1.508807	-3.234525	0.461488
28	C	-1.076072	0.240661	0.127104	-1.091582	0.231033	0.134225
29	O	-3.173463	-1.048918	0.230679	-3.130047	-1.151783	0.055916
30	C	-1.723396	1.316021	-0.769582	-1.693837	1.261803	-0.842417
31	H	-1.552888	2.301455	-0.336614	-1.605782	2.261093	-0.416796
32	H	-1.273484	1.296816	-1.762436	-1.150704	1.244283	-1.787527
33	C	-3.855347	0.092856	0.030481	-3.838432	-0.044184	-0.227428
34	O	-4.962373	0.232165	0.517487	-4.992543	0.050633	0.148253
35	C	-3.248987	1.132639	-0.905626	-3.190714	1.010745	-1.117892
36	O	-1.421123	0.475496	1.507838	-1.581020	0.469888	1.469813

37	C	-0.593893	1.276156	2.376050	-0.885776	1.329385	2.395742
38	H	-0.865596	0.924394	3.375411	-1.232345	0.978226	3.371879
39	H	0.462566	1.037893	2.216796	0.192536	1.149054	2.342424
40	C	-0.835582	2.779449	2.281207	-1.199203	2.815118	2.247663
41	H	-1.902841	3.008512	2.360881	-2.280070	2.984199	2.216675
42	H	-0.452265	3.211871	1.352969	-0.749469	3.255946	1.353765
43	H	-0.317951	3.272822	3.111596	-0.795285	3.348532	3.115555
44	O	-1.671099	-1.319001	-1.546412	-1.448282	-1.378812	-1.559030
45	C	-2.187546	-2.548456	-2.088605	-1.848518	-2.640580	-2.124727
46	H	-1.530210	-3.391466	-1.856304	-1.178562	-3.446443	-1.810792
47	H	-2.213697	-2.401655	-3.168403	-1.772612	-2.512193	-3.204520
48	H	-3.198594	-2.746736	-1.722338	-2.880805	-2.882955	-1.857475
49	O	-3.899665	2.380626	-0.728573	-3.907529	2.231086	-1.021311
50	H	-4.814877	2.193847	-0.460574	-4.835691	2.007371	-0.840380
51	H	-3.462987	0.739038	-1.911053	-3.292524	0.597380	-2.133069

Table S30: Summary of key structure-activity relationship trends for compounds **1–21**.

Compound	Type	Key Structural Features	Observed Trend
**1–6**	Monomers	Quinone core, intact C-4 carbonyl	High activity maintained
**7**	Monomer	Reduced C-4 (hydroxynaphthoquinone)	Decreased activity due to reduced redox potential
**8**	Heterodimer	No substituent at 6'	Moderate activity
**9**	Heterodimer	Methoxy at C-2; Acetate group C-6′	Enhanced activity
**10**	Heterodimer	Improved activity compared to **8**, due to ethyl group at C-6′	Slightly reduced potency
**11**	Heterodimer	Additional methoxy at C-2'	Most potent heterodimer
**12**	Heterodimer	Similar to **10** but different linkage at C-6' (Improved activity)	Potent
**13**	Heterodimer	Altered stereochemistry at linkage	Inactive; likely due to instability / off-target activity
**14**	Perenniporide	No substituent at C-4	Lacks critical hydrogen-bonding group (No OH at C-4)
**15–18**	Perenniporides	Hydroxy/ethoxy substitutions	Moderate to good activity
**19**	Perenniporide	Long aliphatic chain + terminal Carboxylic acid	Highly active
**20**	Perenniporide	Branched chain + Carboxylic acid	Highly active
**21**	Perenniporide	No Carboxylic acid functional group	Slightly less active
